# Non‐Covalent Reversibly Photoconvertible Fluorescent Tags for Wash‐Free Protein Labeling

**DOI:** 10.1002/advs.78041

**Published:** 2026-09-29

**Authors:** Mrinal Mandal, Yuriy Shpinov, Aliénor Lahlou, Fivos Pham, Lina El Hajji, Ian Coghill, Eve Laureau, Marie‐Aude Plamont, Franck Perez, Thomas Le Saux, Isabelle Aujard, Arnaud Gautier, Ludovic Jullien

**Affiliations:** ^1^ Chimie Physique et Chimie du Vivant Département de chimie École normale supérieure, PSL University, Sorbonne Université, CNRS Paris France; ^2^ Sony Computer Science Laboratories Paris France; ^3^ Institut Curie Université PSL CNRS UMR144 Paris France

**Keywords:** fluorogenic chemogenetic tag, reversibly convertible fluorophores, reversibly photoswitchable fluorophore

## Abstract

Reversibly photoswitchable fluorophores are widely used in advanced bioimaging, but their design remains demanding. Here, we introduce a new series spanning the whole visible range, which results from combining a large set of fluorogens with the **FAST** protein scaffold. We first demonstrate that these well‐established fluorescent protein labeling tags turn into negative reversible photoswitchers upon decreasing the fluorogen concentration and increasing light intensity. We then show that using not one but two fluorogens adds new responses to illumination. Thus, we obtain positive reversible photoswitchers that increase their brightness under illumination. We also generate a palette of non‐covalent reversibly photoconvertible fluorescent proteins changing their fluorescence color upon illumination, a reversible behavior that still remains absent in regular fluorescent proteins. This light‐induced color change opens the possibility to discriminate six spectrally similar **FAST** variants in live cells upon demonstrating the superiority of using multiple spectral channels for exploiting the time dependence of the fluorescence response to illumination.

## Graphical abstract

FAST proteins combined with one or two fluorogens undergo reversible fluorescence switching or photoconversion through photoisomerization. At low fluorogen concentration and high light intensity, they act as negative or positive photoswitchers and photoconvertible tags, which are promising for discriminating FAST variants.

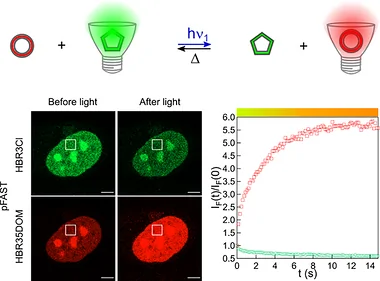

## 1 Introduction

Molecules experience multiple fates after light absorption [1]. Among them, fluorescence and photoisomerization acting in tandem have found numerous attractive applications in advanced fluorescence bioimaging [2] such as superresolution microscopy [3, 4, 5, 6] or dynamic contrast for multiplexed imaging [7, 8, 9, 10, 11, 12, 13, 14, 15, 16, 17]. However, fluorescence emission and photoisomerization are competitive relaxation processes, the former generally being notably slower than the latter [1]. Hence, to produce molecules that exhibit good performance with respect to both of them is highly demanding.

The reversibly photoisomerizable fluorophores generally involve a molecule engaged in the light‐triggered isomerization of a photochromic motif (e.g. (*Z*)‐(*E*) isomerization and/or ring opening‐closure reaction) that is possibly followed by a thermally‐driven reaction (e.g. protonation or aggregation) [18, 19]. In reversibly switchable fluorescent proteins (RSFPs) [20, 21, 22], a single chromophore covalently bound to the protein scaffold achieves to combine both photoisomerization and fluorescence emission. In contrast, in synthetic systems, the light‐triggered molecule is not necessarily fluorescent. Hence, most realizations necessitate that it be linked with one (or more) fluorophores and involve photoinduced energy or electron transfer to drive photoswitching or photoconversion of fluorescence. Therefore, the resulting multichromophoric system is rather large, and its design and accessible color changes are photo(physical)chemically constrained. Here, we introduce another strategy. Reversibly photoswitching fluorophores involve a host turning on the fluorescence of a photoisomerizable fluorogenic guest, which gets liberated and dark upon illumination. When the medium further contains another fluorogenic guest acting as a competitor, illumination can change the color of fluorescence emission, thereby opening a path towards unprecedented non‐covalent reversibly photoconvertible fluorescent tags.

Wash‐free chemogenetic fluorescent systems involving non‐covalent interaction between a biomolecular tag and a fluorogen in water are favorable for implementing the latter strategy. Combining synthetic fluorophores with genetically encoded tags for labeling of proteins [23, 24, 25, 26] and RNA [27], they are increasingly used in view of their labeling reversibility and continuous fluorogen replenishment [28]. Reported in a few non‐covalent chemogenetic systems [29, 30, 31, 32, 33], fluorogen photoisomerization has recently benefited from a thorough theoretical and experimental study [34] in the Fluorescence‐activating and Absorption‐Shifting Tag (**FAST**) series [35, 36]. A favorable kinetic regime of “photoejection” was identified at low fluorogen concentration and high light intensity, in which the limits of moderate fluorogen photoisomerization in the photostationary state are overcome to reach high fluorescence on/off ratios. Hence, it resulted in the generation of **RSpFAST,** combining **pFAST** [37] with acidic fluorogens as a series of green non‐covalent reversibly photoswitching fluorescent proteins (ncRSFPs). Here, we show that photoejection is not restricted to a few acidic fluorogens interacting with **pFAST**, but that it is a general dynamic behavior observed with multiple fluorogens when they interact with **FAST** variants such as **nirFAST** [38]. Hence, we deliver a large set of negative reversible photoswitchers, which turn off their fluorescence emission tailorable from the blue to the infrared wavelength range under illumination. We then expand the exploitation of photoejection. Overcoming the combination of the protein scaffold with a single photoisomerizable fluorogen [37, 39], we harness pairs of fluorogens to turn **FAST** variants into various types of photoswitchers. Thus, we generate a large palette of non‐covalent reversibly photoconvertible fluorescent proteins (ncRCFPs) changing the color of their fluorescence emission upon illumination, a reversible behavior that still remains absent in fluorescent proteins [20, 40, 41]. Further introducing a dark photoisomerizable fluorogen, we also produce positive reversible photoswitchers that increase their brightness under illumination. As an application of ncRCFPs, we eventually use a pair of fluorogens to open the possibility to discriminate up to six spectrally similar **FAST** variants in living cells by exploiting the distinct time dependence of their fluorescence response to illumination in two spectral channels.

## 2 Results

### 2.1 The Concept

When a fluorogen in its photoisomerized state **F’** exhibits a much lower affinity for the **FAST** scaffold **P** than in its initial thermodynamically stable state **F**, we recently demonstrated that illumination of the **FAST**‐**F** complex denoted **B** activates a photocycle, which involves continuous disruption of the **FAST**‐**F’** complex denoted **B’**, and **F** recombination with the photoliberated **FAST** (Figure 1) [34]. Combined with a fluorogen (path a), **FAST** behaves as **RSFAST**, a reversible negative photoswitcher that turns from bright to dark upon illumination and thermally recovers its initial fluorescence. Its fluorescence signal exhibits maximal decay when the exchange between its free and bound states is slower than fluorogen photoisomerization (typically at light intensity higher than 0.1 E·m^−2^·s^−1^ – a few W.cm^−2^ – and fluorogen concentration lower than 1 µm) [34]. Here, **FAST** is further combined not anymore with *one* but with *two* fluorogens, which considerably expands the repertoire of light‐driven fluorescence changes. Hence, thermally recovering reversibly photoconvertible fluorescent proteins (paths b,c) and reversible positive photoswitchers (path d) may become accessible as a consequence of the competition for occupying the tag complexing site (Section S1.2).

**FIGURE 1:**
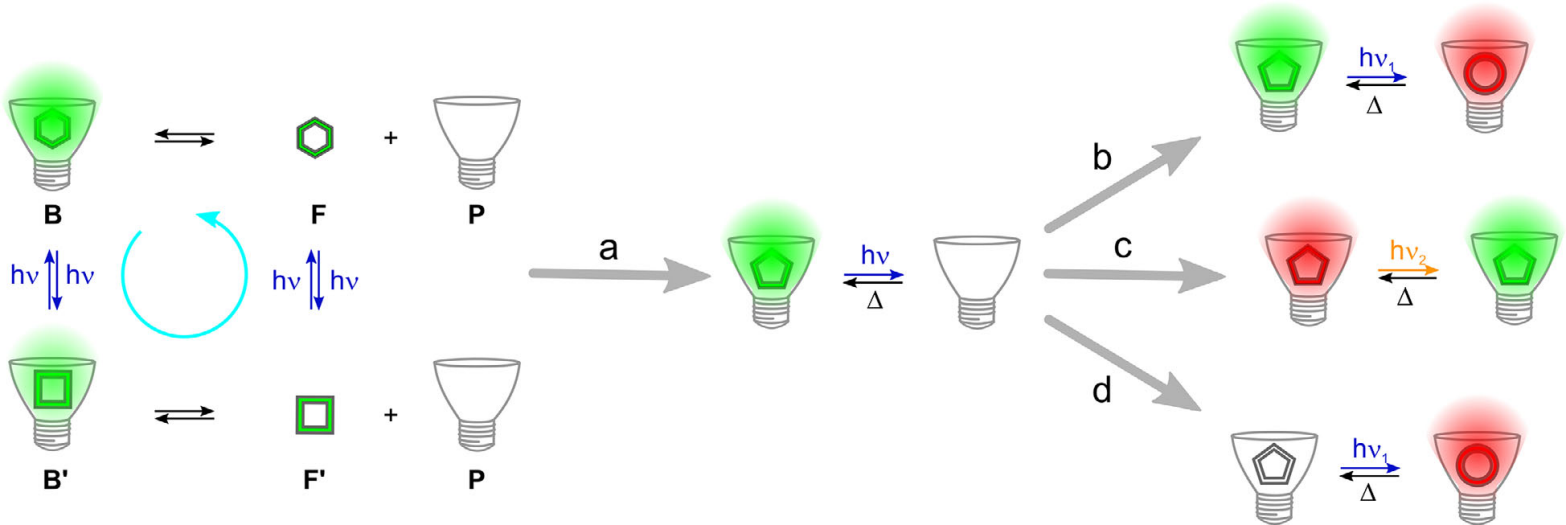
Non‐covalent reversibly photoswitchable and photoconvertible fluorescent tags with thermal recovery for wash‐free protein labeling. With one fluorogen, the four‐state photocycle of **FAST**‐based ncRSFPs reduces to a two‐state reversible negative photoswitcher in the kinetic regime of photoejection (path a) [34]. Further exploiting photoejection but with two fluorogens, the combination of a reversibly photoisomerizable fluorogen giving a bright complex and either a non‐photoisomerizable (path b) or a photoisomerizable (path c) one giving a bright complex of different color provides a reversibly photoconvertible fluorescent protein, whereas the combination of a reversibly photoisomerizable fluorogen giving a dark complex and a non‐photoisomerizable one giving a bright complex (path d) yields a reversible positive photoswitcher. See also Figure S1.

### 2.2 The Screening Step

To evaluate our concept, we submitted a large set of 15 reported [35, 37, 38, 39, 42] and 7 newly synthesized fluorogens absorbing light over the whole UV–vis wavelength range (Figure 2 and Section S2.1) to a preliminary series of experiments: (i) **HBO3M**, **HBO35DM**, **HBT35DM**, and **HBR3CN** absorb at the near UV–vis border and emit in the blue; (ii) **HBR3M**, **HBR25DM**, **HBR3Cl**, **HBR3Cl5F**, **HBR3F5OM**, **HBR35DF** absorb in the blue and emit in the green; (iii) **HBT35DOM** and **HBP35DOM** absorb in the cyan and emit in the yellow; (iv) **HBR35DOM** absorb in the green and emit in the orange; (v) **HPAR3OM** and **HPAR35DOM** absorb in the red and emit at the red‐near infra‐red border. We further produced fluorogens possessing lowered pK for enhanced **FAST** affinity (**HBT3CN**, **HBP3CN**, and **HBTH3CN** as blue emitters, and **HPAR3Cl**, **HPAR3F5OM** as red emitters) or low brightness (**HBIR3Cl**, **HBIR35DOM** as cyan and orange absorbers, respectively) [34, 37].

**FIGURE 2:**
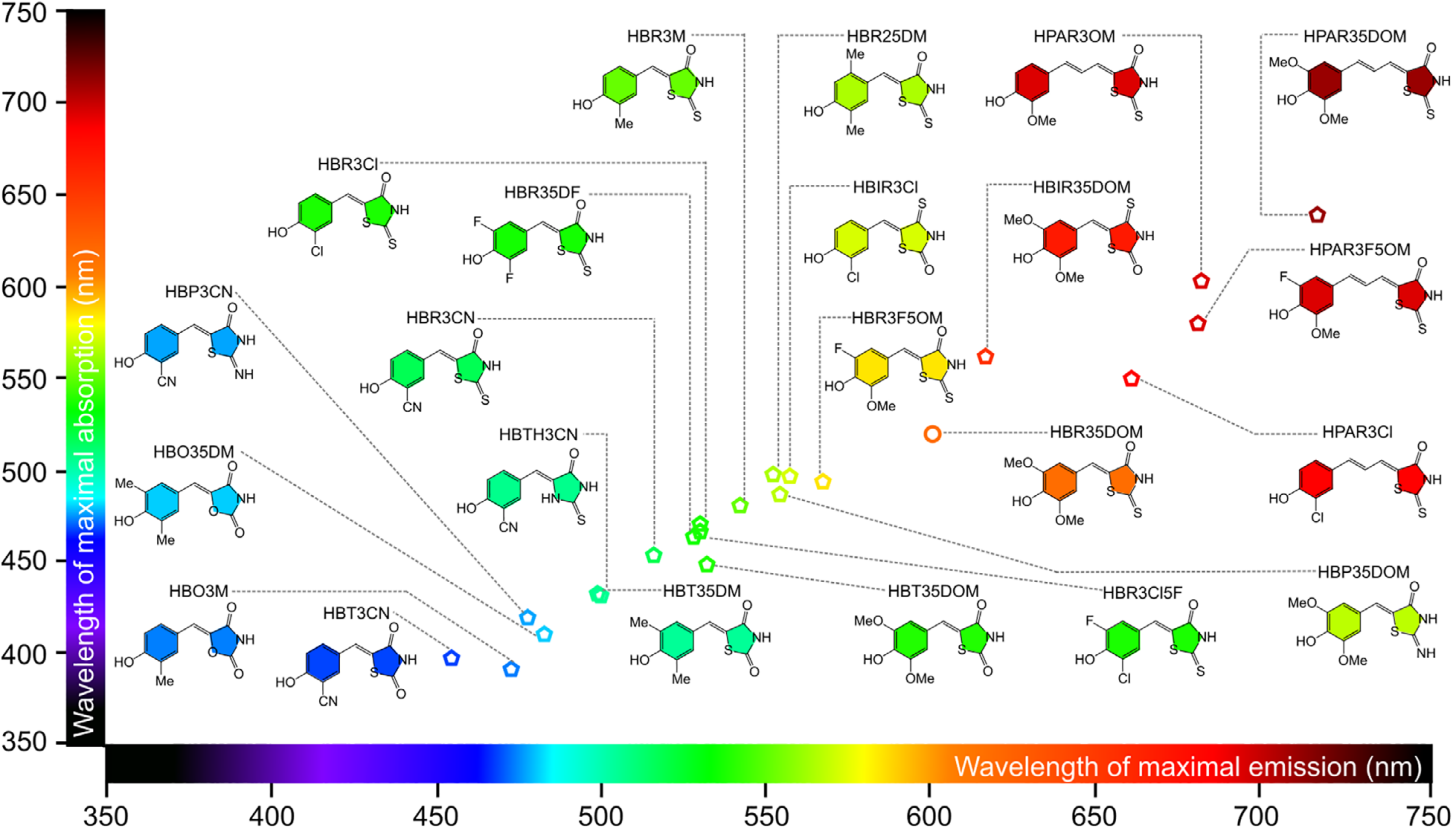
Palette of **FAST** fluorogens screened for reversible photoisomerization.

After recording their absorption and emission spectra (Section S3.1), these 22 fluorogens have been assayed for reversible photoisomerization (Section S3.2), thermal recovery (Section S3.3), and proton exchange (Section S3.4). Thus, we concluded that these visible range‐spanning fluorogens act as photoswitches upon photoisomerizing (except **HBR35DOM**) with similar effective cross sections (sum of the cross sections for the forward and inverse transitions; 10^3^ m^2^.mol^−1^ range at the absorption maximum), thermally recover on a time scale ranging from less than 1 s to more than 1 h, and exhibit a proton exchange constant between 5 and 9. We then restricted the next experiments to a subset of representative fluorogens giving **FAST** complexes spanning the whole absorption/emission wavelength range (Section S4.1) and measured 0.1–10 nm for the range of the dissociation constant of their complexes with **pFAST** and **nirFAST** (Section S4.2). The results are shown in Table 1.

**TABLE 1:** Photophysical and thermodynamic properties of the complex between **pFAST** and **nirFAST** and the (*Z*)‐stereoisomer of the fluorogens. Solvent: 1× pH = 7.4 PBS buffer. *T* = 293 K.

Fluorogen‐FAST	λ_abs_^max^	λ_em_^max^	ε	Φ	B	K_d_
	[nm]^a^	[nm]^b^	[mM^−1^cm^−1^]^c^	^d^	[mM^−1^cm^−1^]^e^	[nM]^f^
**HBT3CN‐pFAST**	398	455	33	—	—	—
**HBO3M‐pFAST**	392	473	14	0.08	1000	—
**HBP3CN‐pFAST**	420	478	34	—	—	—
**HBO35DM‐pFAST**	411	483	6	0.23	1300	—
**HBT35DM‐pFAST**	433	499	33	0.10	3300	14.0
**HBTH3CN‐pFAST**	432	500	39	—	—	—
**HBR3CN‐pFAST**	454	516	44	0.02	880	23
**HBR35DF‐pFAST**	464	528	56	0.06	3360	0.14
**HBR3Cl‐pFAST**	471	530	41	0.08	3280	0.10
**HBR3Cl5F‐pFAST**	467	530	51	0.05	2550	0.14
**HBT35DOM‐pFAST**	449	532	60	0.27	16200	—
**HBR3Cl‐nirFAST**	439	540	60	0.08	4800	0.14
**HBR3M‐pFAST**	481	542	54	0.23	12400	3.0
**HBR25DM‐pFAST**	498	552	45	0.29	13000	0.80
**HBP35DOM‐pFAST**	487	554	37	0.33	12200	—
**HBIR3Cl‐pFAST**	497	557	29	0.002	50	0.40
**HBR3F5OM‐pFAST**	494	567	30	0.42	12600	0.70
**HBR3F5OM‐nirFAST**	487	570	26	0.15	4000	0.90
**HBR35DOM‐pFAST**	520	600	44	0.35	15000	7.7
**HBR35DOM‐nirFAST**	521	600	26	0.24	6240	6.7
**HBIR35DOM‐pFAST**	562	616	40	0.10	4000	—
**HPAR3Cl‐nirFAST**	540	650	24	0.08	1920	0.70
**HPAR3F5OM‐nirFAST**	580	680	30	0.14	4200	0.70
**HPAR3OM‐nirFAST**	603	681	35	0.20	7000	0.70
**HPAR35DOM‐nirFAST**	639	716	43	0.07	2970	1.6

^a^ Wavelength of maximum absorption.

^b^ Wavelength of maximum emission.

^c^ Molar absorption coefficient at λ_abs_^max^.

^d^ Fluorescence quantum yield.

^e^ Molecular brightness = Φ×ε.

^f^ Dissociation constant. The photophysical data for **HBR3M**, **HBR25DM**, **HBR35DOM**, **HBIR35DOM**, **HBT35DM**, **HBT35DOM**, **HBO3M**, **HBO35DM**, **HBP35DOM**, **HPAR3OM**, and **HPAR35DOM** were taken from reference [37].

### 2.3 A Series of Negative FAST‐based Fluorescence Photoswitchers

As previously observed with acidic fluorogens interacting with **pFAST** [34], irradiating the present **FAST**‐fluorogen complexes in PBS pH 7.4 buffer with 480 or 640 nm light resulted in decays in the fluorescence intensity (Section S4.2 and Figures S24,S28 and S31) that could be reversed in the dark, indicating reversible photochemistry. Photoejection of the photoisomerized fluorogen from its **pFAST** tag in labeled cells was previously [34] observed at (i) fluorogen concentrations in the 0.1–1 µm range and (ii) light intensities higher than 0.1 Ein.m^−2^.s^−1^ (a few W.m^−2^ range), which is widely encountered in optical microscopies. Anticipating a similar behavior for all the photoisomerizable fluorogens [34], we led a first series of experiments devoted to evidence photoejection and delimit the experimental conditions of its observation.

Confocal microscopy was used to image live HeLa cells expressing **H2B‐pFAST** or **H2B‐nirFAST** at the nucleus that were conditioned with fluorogens at 0.1–10 µm concentration (Table S9 for the acquisition conditions) [34]. As displayed in Figure 3 and Figures S33 and S34, the fluorescence level at the nucleus exhibited a fast decrease of up to 80% upon applying repetitive scans in the presence of 0.1 µm of fluorogen for **HBT35DM**, **HBR3M**, and **HBR25DM** with **pFAST**, and for **HPAR3Cl**, **HPAR3OM**, and **HPAR35DOM** with **nirFAST**. These light‐induced fluorescence drops were successfully fitted by using a monoexponential function (upon adding a slow linear drift to account for photobleaching). Such a functional dependence is in line with the photoejection behavior predicted when **RSFAST** reversibly generates a darker state upon applying illumination in a scanning mode like in confocal microscopy (see Section S1.3).

**FIGURE 3:**
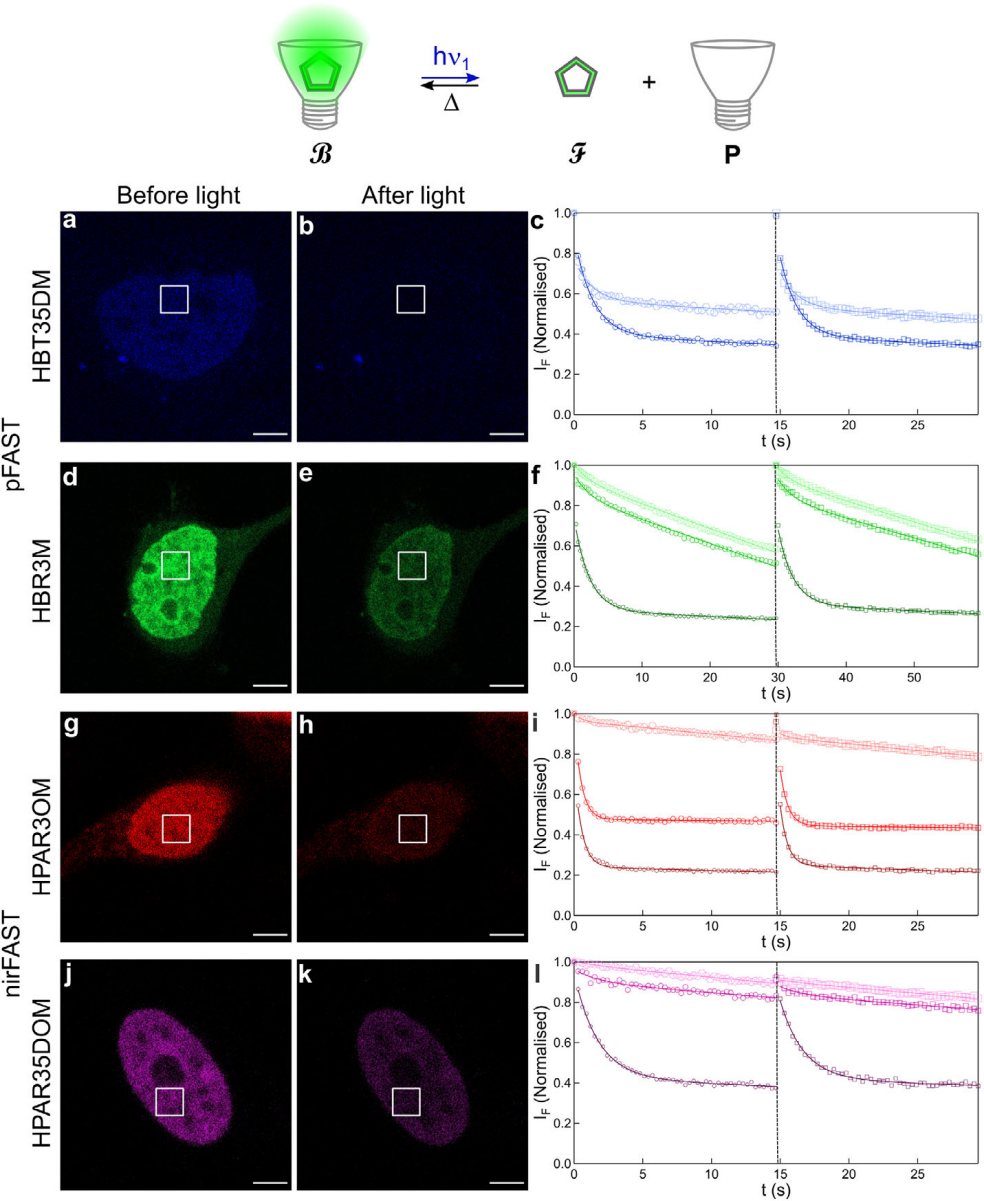
**FAST** to generate a palette of negative ncRSFPs. 405 (**a**–**c**), 488 (**d**–**f**), and 639 (**g**–**l**) nm light‐induced fluorescence emission from **HBT35DM** (**a**–**c**), **HBR3M** (**d**–**f**), **HPAR3OM** (**g**–**i**), and **HPAR35DOM** (**j**–**l**) in **H2B‐pFAST** (**a**–**f**) or **nirFAST** (**g**–**l**)‐labeled live HeLa cells observed in confocal microscopy in the presence of the fluorogen in DMEM pH 7.4 (see also Figures S33–S40). Photoejection is evidenced at 0.1 µm concentration from the contrast at the nuclei between the initial (**a**,**d**,**g**,**j**) and photosteady state (**b**,**e**,**h**,**k**) images, its reversibility by the similar loss of fluorescence normalized by its initial value after two consecutive series of frame acquisition (circles and squares markers after the first and second series respectively) in the square region of interest at nuclei, and its concentration‐dependence by the distinct decays observed at 0.1 (small colored markers), 1 (medium colored markers), or 10 (large colored markers) µm fluorogen concentration (**c**,**f**,**i**,**l**). Colored lines: exponential fits of the observed fluorescence drops with Equation (S33) by further adding a slow linear drift to account for photobleaching. Scale bar: 5 µm. *T* = 310 K. See Tables S9 and S10 for the conditions of image acquisition.

As previously observed [34], photoejection reversibility was evidenced by essentially recovering initial fluorescence upon introducing a sufficient delay (typically 120 s) before applying a new series of repetitive scans. Moreover, the fluorescence drop originating from photoejection could be abolished by increasing the fluorogen concentration above 1 µm, where we only observed slow photobleaching, rejoining well‐established experimental conditions for **FAST** labeling [35, 37]. Under such concentration conditions, the rate of **FAST**‐Fluorogen recombination efficiently counterbalances the rate of light‐induced disruption, and one only observes a fluorescence decay caused by photobleaching [34].

Light‐induced fluorogen ejection followed by thermal rebinding was further observed in three distinct compartments (nucleus, mitochondria, and membrane) of live HeLa cells and U2OS cells (Section S5.1 and Figures S33–S40), which suggests this phenomenon to be biologically robust.

### 2.4 Optimizing the Light‐Induced FAST Fluorescence Change in the Presence of Two Fluorogens

Photoejection can further be exploited when **FAST** interacts with two fluorogens. Indeed, illuminating a first reversibly photoisomerizable fluorogen F_1_ drives photoconversion of its thermodynamically stable (*Z*) to its (*E*) stereoisomer, which becomes less complexed by the tag in the 0.1–1 µm total fluorogen concentration range [34]. It results in a backward shift of the extent of F_1_ complexation, thereby liberating free tag sites now made available for complexation of a second fluorogen F_2_, reversibly photoisomerizable or not. Depending on the brightness, emission color, and thermokinetic properties of F_1_ and F_2_, illumination can generate various time evolutions of the **FAST** fluorescence (Figure 1).

The concentrations of F_1_ and F_2_ must be properly fixed to benefit from important fluorescence changes. Rational optimization would require collecting multiple thermokinetic constants for both fluorogens, which is demanding [34]. In the absence of extensive kinetic information, we first used the respective dissociation constants *K*_d,1_ and *K*_d,2_ of the **FAST‐**(*Z*)‐Fluorogen complexes as guides for tuning the range of the concentrations of F_1_ and F_2_ (Tables S1,S5 and S6). Indeed, fixing the fluorogen concentration to *K*_d_ optimizes both the complexation extent (governing the fluorescence level) and its change upon modifying the total fluorogen concentration (governing fluorescence sensitivity to illumination).

Yet, the latter guideline is not sufficient to address biological applications since endogenous cellular components interact with the **FAST** fluorogens. Hence, we previously concluded that the intracellular concentration of the free state of even rather hydrophilic fluorogens is lowered from the applied external fluorogen concentration by a factor of 10 to 10^2^ [34]. Hence, we eventually ended up by exploring extracellularly applied fluorogen concentrations ranging between 0.1 and a few µm for optimization.

### 2.5 A Palette of Reversibly Photoconvertible Fluorescent Proteins

To get reversibly photoconvertible fluorescent tags, we first used the strategy displayed in path b of Figure 1 and retained **HBR3Cl** (yielding a bright green **pFAST** complex) for the reversibly photoisomerizable fluorogen F_1_, and **HBR35DOM** (giving a bright red complex with **pFAST**) for the non‐photoisomerizable fluorogen F_2_. Figure 4a1–a10 shows the time‐lapse images of the nucleus of a 1:0.5 µm **HBR3Cl**:**HBR35DOM**‐conditioned live HeLa cell expressing **H2B‐pFAST**, which result from applying repetitive illumination scans in confocal microscopy. One observes a drop and a rise of the fluorescence emission of F_1_ and F_2,_ respectively, which evidences successful implementation of photoconvertibility in the **FAST** system. As displayed in Figure 4b, the reversibility of photoconvertibility in **pFAST**‐**HBR3Cl**:**HBR35DOM** was further evaluated by observing only 4% and 9% fluorescence loss in the green and red channels after 4 cycles of photoejection‐90 s‐long thermal recovery (see also Figures S41 and S46). The robustness of photoconvertibility was further supported by the observation of a similar behavior in a variety of cellular compartments (beyond the nucleus, mitochondria, and membrane) of live HeLa and U2OS cells (Section S5.2 and Figures S41–S47).

**FIGURE 4:**
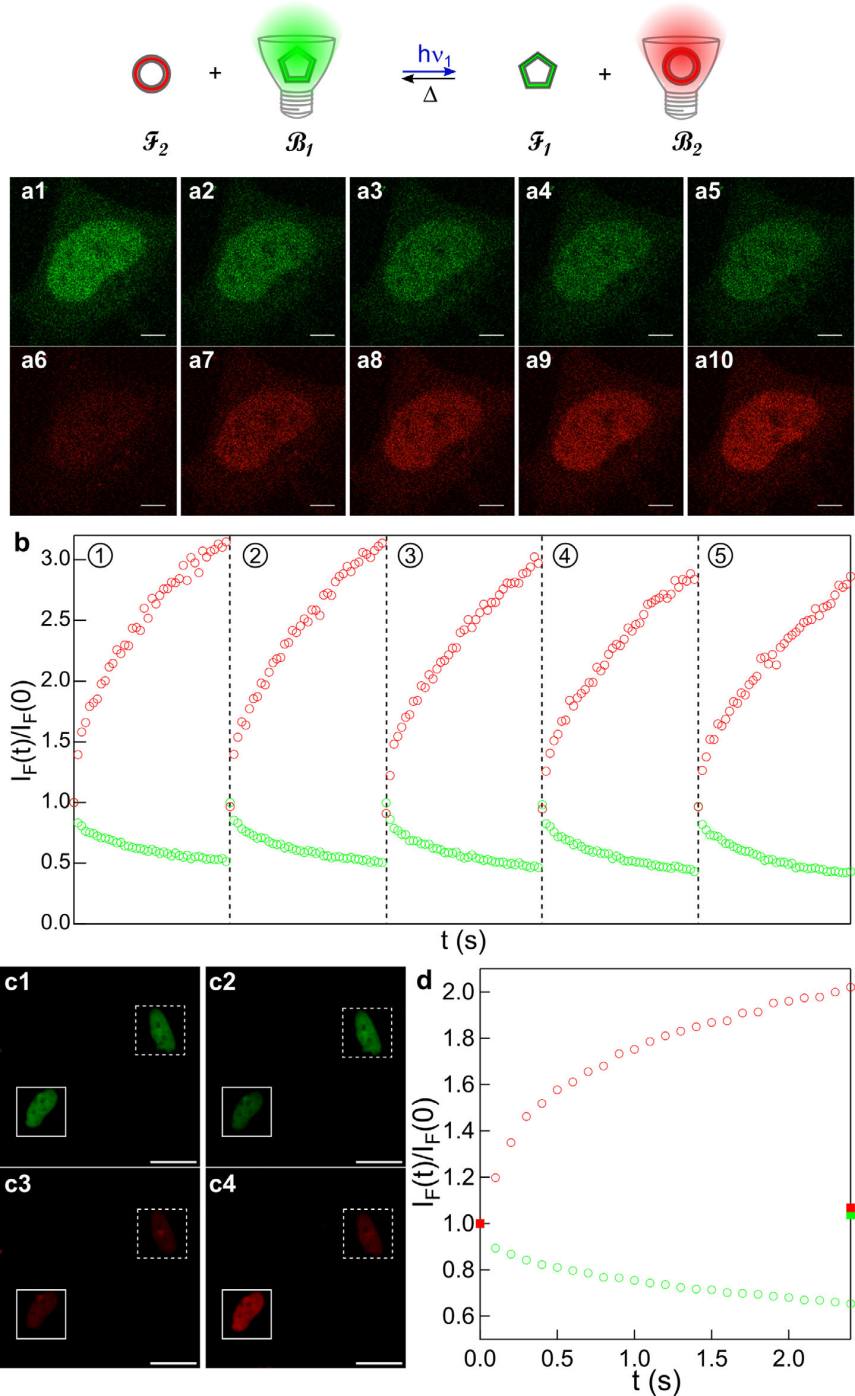
*The* ***pFAST***‐***HBR3Cl***: ***HBR35DOM*** *: ncRCFP*. **a1**–**a10**: Time‐lapse of 488 nm light‐induced change of fluorescence emission in the green (**a1–a5**) and red (**a6–a10**) channels of confocal microscopy from **H2B‐pFAST**‐labeled live HeLa cells conditioned with the **HBR3Cl**:**HBR35DOM** mixture of fluorogens in DMEM pH 7.4; **b**: Benchmarking of photostability of **pFAST**‐**HBR3Cl:HBR35DOM**. Evolution of the fluorescence intensity normalized by its initial value in the labeled nucleus shown in **a** over the course of five photoejection‐thermal recovery cycles. Each vertical dashed line represents a 90 s thermal recovery period in the dark (not included in the time axis for compactness). The duration of each illumination period is 5.9 s; **c1**–**c4**: Selective photoconversion at the nucleus of a targeted **H2B‐pFAST**‐labeled live HeLa cell under local (solid white square) 470 nm illumination within a population. Local photoconversion is evidenced from comparing the initial (**c1**, **c3**) and final (**c2**, **c4**) fluorescence images in the green (**c1**, **c2**) and red (**c3**,**c4**) channels. The local drop and rise of the fluorescence level, normalized by its initial value at the targeted nucleus in the green (green circles) and red (red circles) channels (**d**) contrasts with the constant normalized fluorescence levels at the non‐illuminated nucleus (green and red squares). Scale bar: 5 µm (**a1–a10**,**b**) or 28 µm (**c1–c4**). *T* = 298 (**c**) or 310 (**a**, **b**) K. See Tables S9 and S10 for the conditions of image acquisition.

The 1:2 µm **HBR3Cl**:**HBR35DOM** mixture was subsequently implemented in live HeLa cells expressing **H2B‐pFAST** to selectively drive photoconversion at a targeted cell within a cell population by using a recently developed home‐built microscope enabling us for patterned illumination [43]. After recording the first image of two cells in the green (Figure 4c1) and red (Figure 4c3) fluorescence channels under uniform 470 nm illumination, we went on to selectively illuminate the nucleus of a single cell. We observed its fluorescence change with a drop and a rise in the green and red channels, respectively (Figure 4d), ending up in a final spectral signature contrasting with the unchanged one of the non‐illuminated nucleus (Figure 4c2–c4).

The strategy shown in path b of Figure 1 was then extended to three other mixtures involving **HBR35DOM** as the non‐photoisomerizable fluorogen F_2_ (Figure 5). Thus, we retained **HBT35DM** (yielding a blue **pFAST** complex), and **HPAR3OM** and **HPAR3F5OM** (giving both a bright near‐infrared **nirFAST** complex) for the reversibly photoisomerizable fluorogen F_1_. Then, we imaged in confocal microscopy live HeLa cells expressing **H2B‐pFAST** that were conditioned with 10:0.25 µm **HBT35DM**:**HBR35DOM**, or **H2B‐nirFAST** with 0.5:0.25 µm **HPAR3OM**:**HBR35DOM** and with 0.5:0.5 µm **HPAR3F5OM**:**HBR35DOM**. Similar changes in fluorescence, normalized by its initial value, were observed for all the investigated fluorogen pairs after a consecutive series of frame acquisitions separated by 90 s thermal recovery period in the dark, thereby validating the generation of four reversibly photoconvertible fluorescent tags (Figure 5a‐c5 and Figure S42).

**FIGURE 5:**
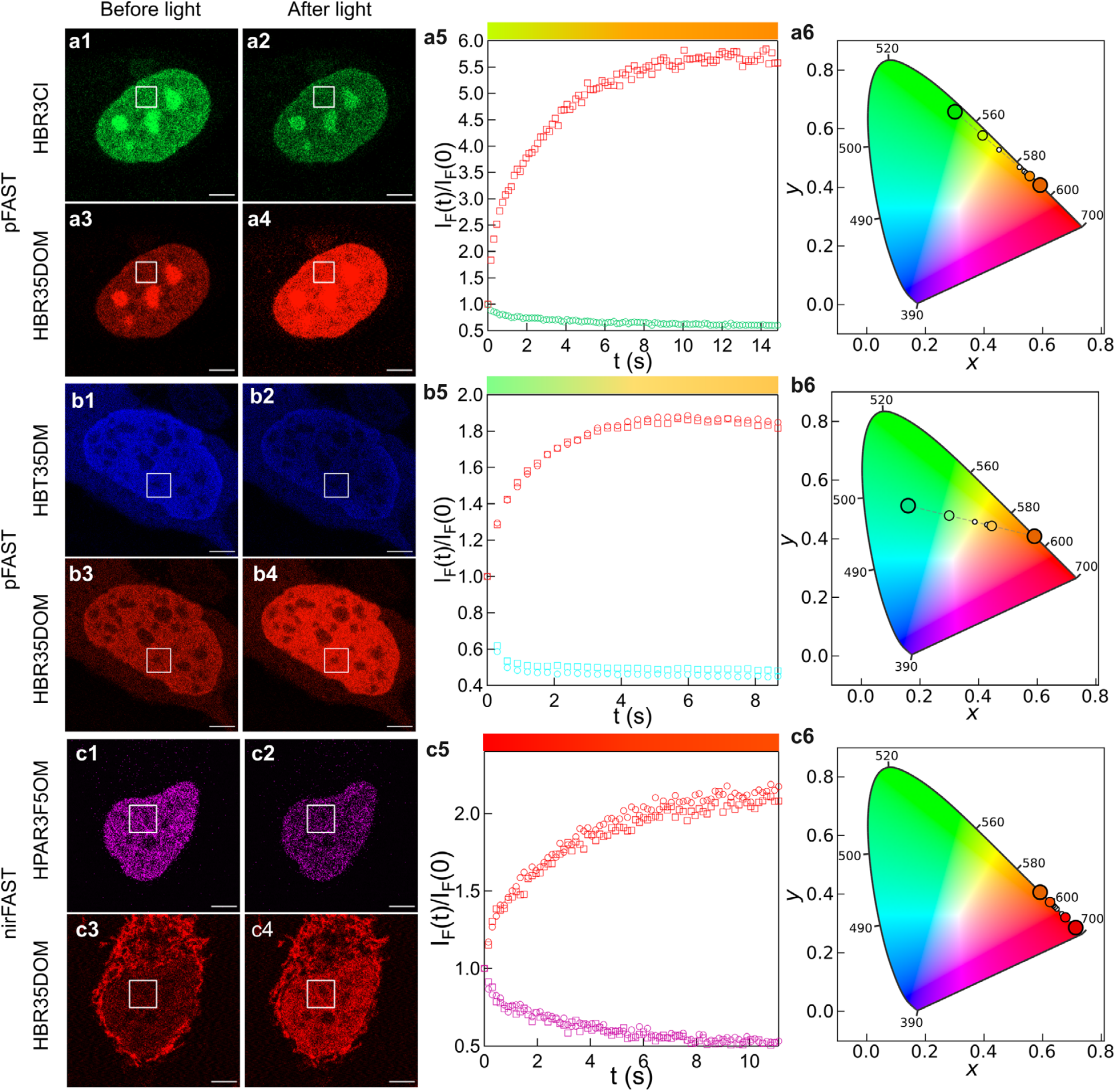
***FAST*** *to generate a palette of ncRCFPs*. **a1‐**‐**c6**: 488 (**a1**–**a6**), 445 (**b1**–**b6**), and 561 (**c1**–**c6**) nm light‐induced changes of fluorescence emission in two wavelength channels of confocal microscopy from **H2B‐pFAST** (**a1**–**b6**) or **nirFAST** (**c1**–**c6**)‐labeled live HeLa cells conditioned with the **HBR3Cl**:**HBR35DOM** (**a1**–**a6**), **HBT35DM**:**HBR35DOM** (**b1**–**b6**), and **HPAR3F5OM**:**HBR35DOM** (**c1**–**c6**) mixtures of fluorogens in DMEM pH 7.4. The fluorescence change is evidenced from the contrast between the initial (**a1**,**a3**,**b1**,**b3**,**c1**,**c3**) and photostationary state (**a2**,**a4**,**b2**,**b4**,**c2**,**c4**) images of the nuclei and the time trace of the fluorescence normalized by its initial value in the region of interest at nuclei (**a5**,**b5**,**c5**), and its reversibility by the similarity of the time traces after two consecutive series of frame acquisition (circles and squares markers after the first and second series respectively in **b5**,**c5**) separated by 90 s thermal recovery period in the dark. In the CIE 1931 color space (**a6**,**b6**,**c6**), the coordinates (x,y; Equation S42) of the fluorescence emission of **HBT35DM**, **HBR3Cl**, **HBR35DOM**, and **HPAR3F5OM** are displayed as large circles, whereas those of the FAST labels conditioned with the fluorogen mixtures at initial and photosteady states are shown as medium‐sized circles. The light‐induced drifts of fluorescence color are linearly sampled with small circles along a dashed line. Scale bar: 5 µm. *T* = 310 K. See Tables S9 and S10 for the conditions of image acquisition.

In fact, the time evolutions of the fluorescence level in two wavelength channels displayed in Figure 5a‐c5 do not easily reflect the real change of fluorescence color at the cell nucleus. Hence, to make its light‐driven change objective, we harnessed the CIE 1931 color space linking the visible spectrum and human color vision [44]. To establish a reference, we first titrated **pFAST‐HBT35DM** by **HBR35DOM** and evidenced that the progressive replacement of one fluorogen by another one delivered a linear trajectory in the color space (Section S4.4 in SI). We then exploited the time evolution of the fluorescence level in both recorded wavelength channels of Figure 5a‐c5 to compute the light‐driven color shift at the labeled nucleus (Section S1.4). Figure 5a‐c6 together with the colored ribbons in Figure 5a‐c5 demonstrate that the **FAST**‐labeled nuclei encounter considerable changes in color under illumination.

To further enlarge the series of ncRCFPs, we then implemented the strategy involving a cocktail of two photoisomerizable fluorogens (path c of Figure 1). We first conditioned live HeLa cells expressing **H2B‐nirFAST** with 1 µm **HBR3F5OM** as F_1_ (yielding a bright green complex) and 1 µm **HPAR3OM** as F_2_ (giving a bright red/infra‐red fluorescent complex) and applied the following sequence of illuminations: (i) recording the initial frame at λ_1_ = 488 nm to image both F_1_ and F_2_; (ii) applying a set of repetitive scans at λ_2_ = 639 nm driving decay of F_2_ fluorescence up to the photostationary state; (iii) recording the final frame at λ_1_ = 488 nm for imaging F_1_ and F_2_. In Figure S43, similar changes in fluorescence color were observed between the initial and photostationary states for two consecutive series of frame acquisition separated by a 90 s thermal recovery period in the dark: the **HBR3F5OM** fluorescence signal increased by 10 and 12%, and the **HPAR3OM** one decreased by 24 and 29% during the first and second series of frame acquisition, respectively.

### 2.6 A Series of Positive Fluorescence Photoswitchers

When F_1_ and F_2_ give a dark photoisomerizable and a bright non‐photoisomerizable **FAST** complex, respectively, F_1_ photoisomerization increases F_2_ fluorescence, thereby generating a positive reversible photoswitcher (path d in Figure 1). For illustration, we adopted F_1_ **HBIR3Cl** (giving a dark **pFAST** complex), and first retained **HBR35DOM** for F_2_. Figure 6a1–a5 show that applying in confocal microscopy sets of 514 nm light scans targeting **HBIR3Cl** photoisomerization, separated by 90 s thermal recovery period in the dark, reversibly doubled red fluorescence at the cell nucleus of live **H2B‐pFAST**‐expressing HeLa cells conditioned with 1:0.25 µm **HBIR3Cl**:**HBR35DOM**. We then again chose **HBIR3Cl** for F_1_ (giving a dark **nirFAST** complex) and used **HPAR35DOM** for F_2_ (yielding a bright red **nirFAST** complex with low absorbance at λ_1_ = 488 nm). Here, the sets of 488 nm light scans reversibly tripled the level of red/infra‐red fluorescence at the cell nucleus of live **H2B‐nirFAST**‐expressing HeLa cells conditioned with 1:0.5 µm **HBIR3Cl**:**HPAR35DOM,** as concluded from comparing the initial (Figure 6b1–b3) and final (Figure 6b2–b4) images recorded at 639 nm.

**FIGURE 6:**
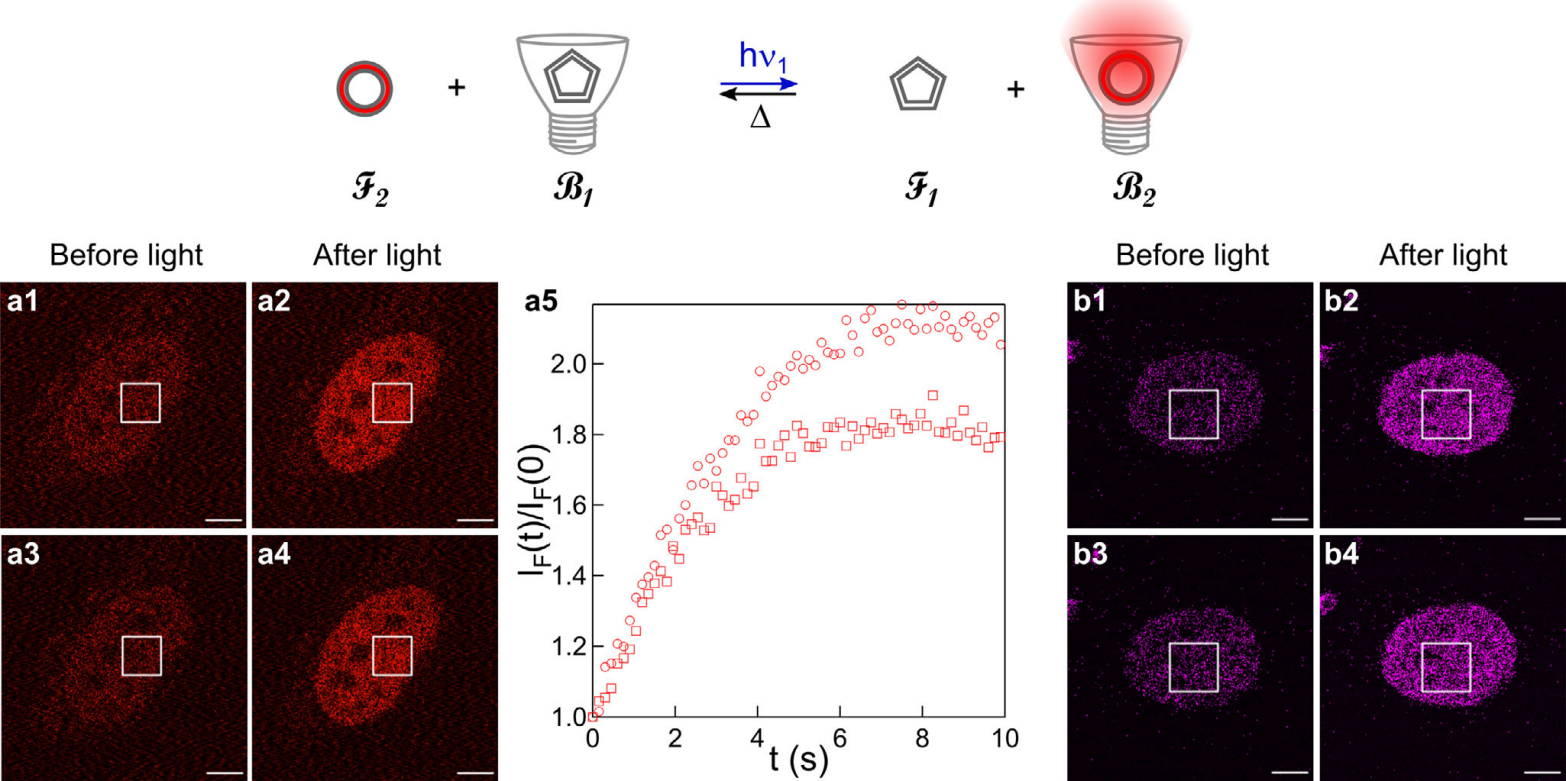
***FAST*** *to generate positive ncRSFPs*. 514 (**a1**–**a5**) and 488 (**b1**–**b4**) nm light‐induced changes of fluorescence images in confocal microscopy from **H2B‐pFAST‐** (**a1**–**a5**) and **H2B‐nirFAST‐** (**b1‐**‐**b4**) labeled live HeLa cells respectively, conditioned with the **HBIR3Cl**:**HBR35DOM** and **HBIR3Cl**:**HPAR35DOM** mixtures of fluorogens in DMEM pH 7.4. The fluorescence change is evidenced by the fluorescence contrast at the nuclei between the initial (**a1**,**a3**,**b1**,**b3**) and photosteady state (**a2**,**a4**,**b2**,**b4**) images, and its reversibility by the similar changes of fluorescence normalized by its initial value in the region of interest after two consecutive series of frame acquisition (circles and squares markers after the first and second series, respectively, in **a5**) separated by a 90 s thermal recovery period in the dark. Scale bar: 5 µm. *T* = 310 K. See Tables S9 and S10 for the conditions of image acquisition.

### 2.7 Application Towards Multiplexed Fluorescence Imaging

When conditioned with a cocktail of two differently colored fluorogens, **FAST** variants are prone to deliver specific kinetic responses to illumination as time traces of the fluorescence level in the spectral channels used to follow the substitution of a fluorogen by the other within the tag cavity. Indeed, these traces report on a rich set of photophysical (i.e., absorption and emission spectra, quantum yields of fluorescence of the **FAST‐Fluorogen** complexes), photochemical (i.e., cross sections of photoisomerization in the free and bound fluorogen states), and thermokinetic parameters (i.e., thermal rate constants of exchange between the four photocycle states) that are anticipated to depend on the **FAST** scaffold.

We first implemented this strategy by discriminating **pFAST** and **nirFAST**, labeling either two different cells or two different compartments of the same cell. For illustration, we retained (i) a mixture of live U2OS cells expressing **H2B‐nirFAST** at the nucleus and **Mito‐pFAST** at mitochondria, and live U2OS cells expressing both (ii) **H2B‐nirFAST** at the nucleus and **Mito‐pFAST** at mitochondria or (iii) **H2B‐pFAST** at the nucleus and **Mito‐nirFAST** at mitochondria. After conditioning with 1:0.5 µm **HBR3Cl**:**HBR35DOM**, these samples were submitted for acquisition of a sequence of confocal microscopy frames at 488 nm. The images were then processed to retrieve the time evolution of the fluorescence response at each pixel in the green and red spectral channels, mostly reporting on the fluorescence emission from **FAST‐HBR3Cl** and **FAST‐HBR35DOM,** respectively (Figure 7a–c,g–i,m–o). We performed the following unsupervised classification to predict the class of each pixel. The videos were downscaled with local averaging, and an intensity mask was applied to remove the background pixels. The red channel and green channel time series of the remaining pixels were normalized to their respective first point and concatenated. Then, a Principal Component Analysis (PCA) was applied to reduce the data to dimension 8, followed by a non‐linear t‐SNE reduction to dimension 2 [45]. We clustered the point cloud into three classes (**nirFAST**, **pFAST,** and noise) using HDBSCAN (Figure 7d,j,p) [46]. By coloring the corresponding points in the original mask, we obtained a segmented mask colored with the predicted class (Figure 7e,k,q). Hence, we were able to discriminate **pFAST** and **nirFAST**, which were found at the location expected for their constructs (Figure 7f,l,r). We further present in the Supporting Information an alternative of this classification method (Section S5.3.1 and Figures S48–S50): We use manually identified Regions Of Interest (ROIs) containing pixels only belonging to class **nirFAST** or class **pFAST** to internally train a classifier and apply the classification to the whole image to retrieve the segmentation mask.

**FIGURE 7:**
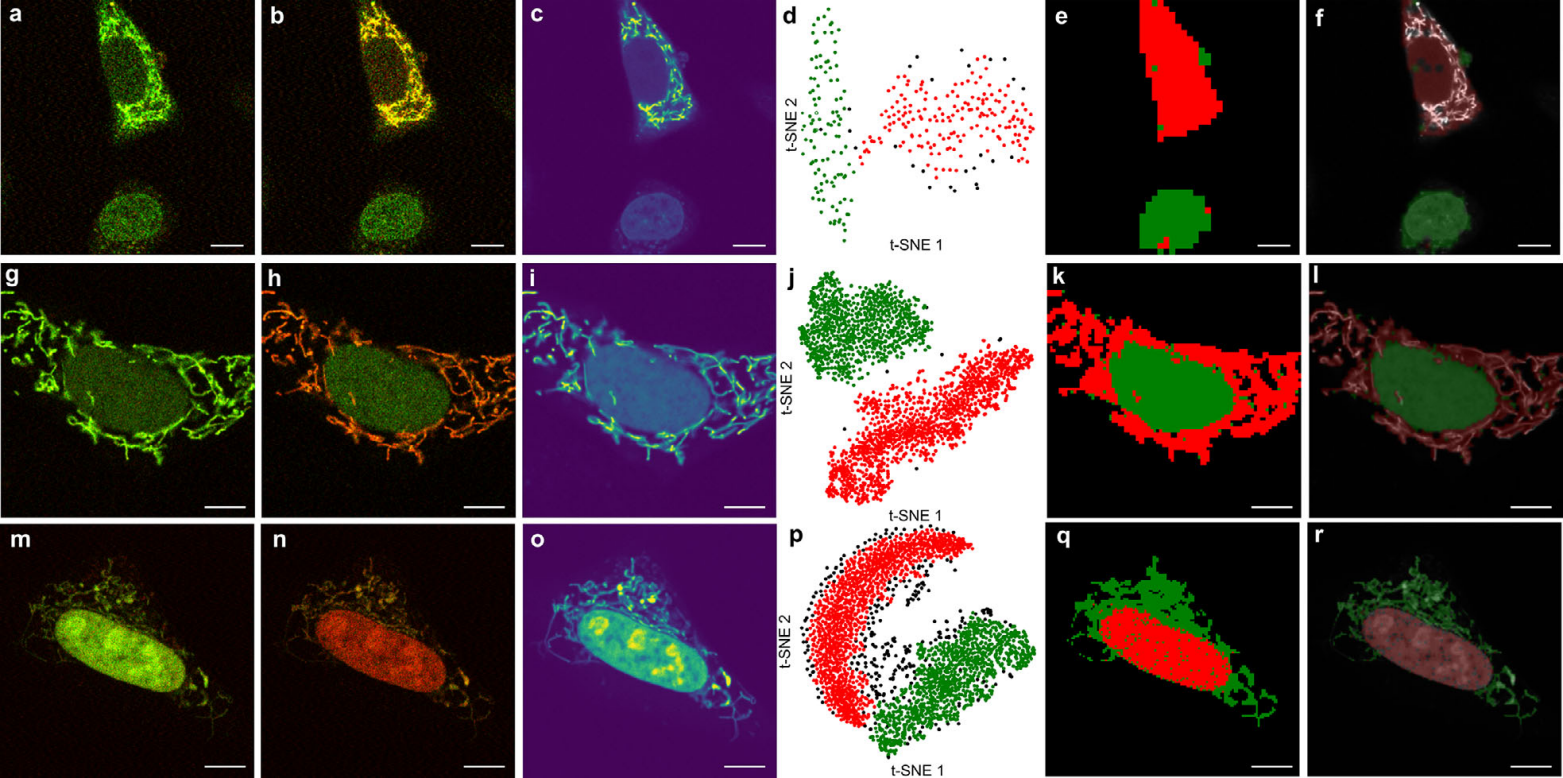
*rcFASTs* for dual‐color dynamic contrast in live U2OS cells conditioned with 1:0.5 µm **HBR3Cl**:**HBR35DOM** and expressing **H2B‐nirFAST** or **Mito‐pFAST** (**a**–**f**), or both **H2B‐nirFAST** and **Mito‐pFAST** (**g**–**l**) or **H2B‐pFAST** and **Mito‐nirFAST** (**m**–**r**). Combined images of fluorescence emission in the green and red channels before illumination (**a**,**g**,**m**) and at the photostationary state (**b**,**h**,**n**) of 488 nm light in confocal microscopy. In **a**,**b**,**g**,**h**,**m**, and **n**, the green (resp. red) channel intensity was rescaled to the maximal pixel intensity reached within the first in **a**,**g**,**m** (resp. the last in **b**,**h**,**n**) frame. The average images of the red and green channels combined are displayed in **c**,**i**,**o**; **d**,**j**,**p**: Dimension reduction from 150 dimensions to 2 dimensions to cluster the data and attribute a class to each pixel (red: **pFAST**, green: **nirFAST**, black: noise). The videos are downscaled with local averaging, a mask is applied to distinguish the background pixels, and the combined green and red channels time series are normalized to their respective first point and concatenated to obtain a time series of 150 data points. An unsupervised linear reduction of the data dimension (Principal Component Analysis, dimension 150 reduced to dimension 8) is followed by a non‐linear dimension reduction technique (t‐SNE, dimension 8 reduced to dimension 2). Then, HDBSCAN clustering is applied with the two classes of interest and the noise class; **e**,**k**,**q**: The illumination mask is colored with the class predicted for each pixel. The noise pixels are identified as background; **f**,**l**,**r**: The class mask in **e**,**k**,**q** is overlaid in transparency with the respective average image in **c**,**i**,**o**. Scale bar: 10 µm. T = 310 K. See Tables S9 and S10 for the conditions of image acquisition.

In order to further evaluate the potential of dual‐color dynamic contrast for multiplexed fluorescence imaging, we examined discrimination among a pool of live HeLa cells labeled at the nucleus with nine distinct **H2B‐xFAST** (x = Y [34], p [36], nir [37], fr [41], t [36], green [47], i [48], o [36], Tsi [49]). After conditioning with 1:0.5 µm **HBR3Cl**:**HBR35DOM**, we acquired at 488 nm a sequence of confocal microscopy frames imaging the nuclei of ten to fifteen single cells from each **H2B‐xFAST**‐labeled sample. The images were then processed to retrieve the time evolution of the fluorescence response at the nucleus in the green and red spectral channels. Figure 8a displays the sets of the time traces of the fluorescence levels in both color channels normalized to their first level and stacked sequentially. Except for **nirFAST**, fluorescence monotonously drops in the green channel, whereas initial increases followed by slow decays are observed in the red channel. Notably, the traces differ by their amplitude as well as by their time dependence.

**FIGURE 8:**
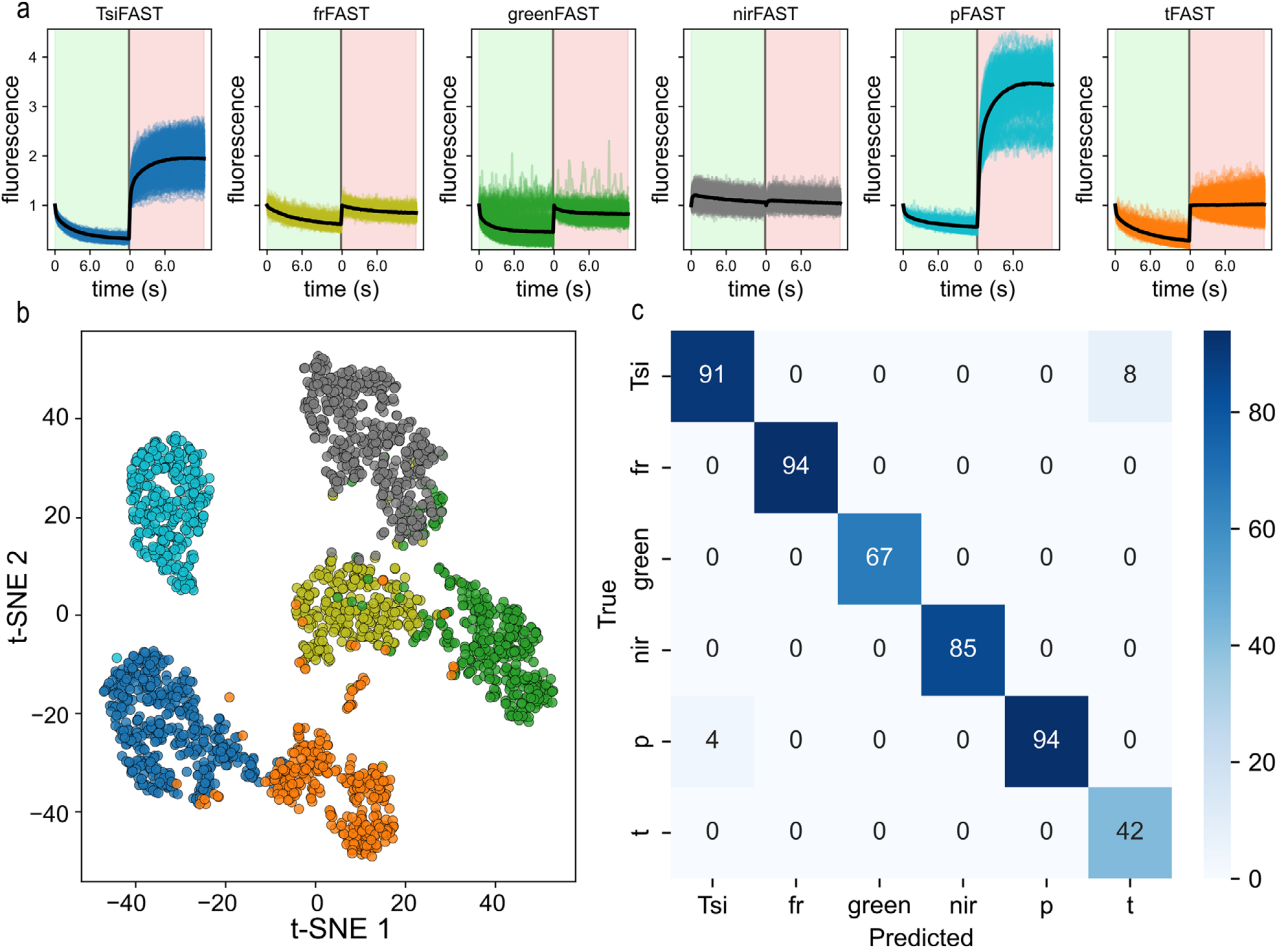
***rcFASTs*** *for multiplexed imaging*. (**a)**: Combined response to 488 nm light in confocal microscopy of the levels of fluorescence emission normalized by its initial value in the green channel from the nuclei of live HeLa cells expressing **H2B‐FAST** mutants (**TsiFAST**, **frFAST**, **greenFAST**, **nirFAST**, **pFAST**, **tFAST**) conditioned with 1:0.5 µm **HBR3Cl**:**HBR35DOM** in the 499–544 (green) and 588–694 (red) nm spectral channels; (**b)**: Dimension reduction from 150‐dimension to 2‐dimension to visualize the separability of the data. A supervised linear reduction of dimension of the whole dataset (Linear Discriminant Analysis, dimension 150 reduced to dimension 5) is followed by a non‐linear dimension reduction technique (t‐SNE, dimension 5 reduced to dimension 2); (**c)**: Confusion matrix associated with the 6 discriminated types of **FAST**‐labeled. *T* = 298 K. See Tables S9 and S10 for the conditions of image acquisition.

To refine this appreciation and conclude about its relevance for discriminating the **FAST** mutants, we evaluated the capacity of a trained machine learning model (Linear Discriminant Analysis) to distinguish the mutants (Section S2.5.5). To artificially increase the dataset size, we split the cells into micro‐regions and considered each as a data sample (2335 fluorescence traces in total). We divided the dataset into a training set and a test set. The test set corresponds to all the micro‐regions retrieved from three cells out of the 10–15 cells for each protein. We tested different numbers of proteins, different normalization methods, and data obtained from the green or red channel only, or combined channels (Section S5.4). We concluded that we can discriminate up to six **FAST** mutants with an accuracy of 98% when considering the combined channels normalized at their first fluorescence level, respectively.

We further demonstrated that the relative amplitude of the signal evolution, and not only its time evolution, is a discriminant trait. Hence, to discriminate six **FAST** mutants, normalizing to the first point yields better classification accuracy than min‐max (0‐1 scaling) normalization (88%). We also showed that combining the two channels presents an improvement in the classification. Combined channels allow discrimination of six **FAST** mutants, whereas using a single channel limits the separability to four different **FAST** mutants for the green channel (accuracy 97%) and three different **FAST** mutants for the red channel (accuracy 100%, Section S5.4). Figure 8b shows a two‐dimensional representation of the separability of the labelled proteins, while Figure 8c displays the confusion matrix on a test dataset, which highlights predictions proposed by the trained algorithm, as well as errors.

## 3 Discussion

### 3.1 From Chemogenetic Fluorescent Labels to Reversible Negative Photoswitchers

We previously evidenced that acidic fluorogens exhibit photoejection from **pFAST** in live cells at low extracellularly applied concentration of fluorogen and high light intensity [34]. Here, we showed that the photoejection scope expands to less acidic fluorogens. In fact, it concerns essentially all the investigated fluorogens and **FAST** mutants. Hence, **RSFASTs** exhibit a dual behavior: the stable and wash‐free fluorescent labeling tag turns into a negative reversible photoswitcher by lowering the extracellularly applied fluorogen concentration and increasing light intensity [34]. In live cells in a regime of high light intensity typically below 1 µm, **FAST** acts as a reversible fluorescent photoswitcher. In contrast, photoejection is essentially abolished above typically 1 µm and **FAST** then acts as a fluorescent label, now well‐established [35, 37].

Therefore, **RSFASTs** are prone to expand the collection of regular RSFPs. With respect to the latter, **RSFASTs** exhibit essentially similar photophysical performances, rather lower effective photoisomerization cross sections, and are currently restricted to thermally‐driven fluorescence recovery (see Section S5.1.3 and Table S7). Eventually note that photoejection and shift of the labeling behavior may manifest themselves beyond **FAST** since other non‐covalent chemogenetic systems (e.g., Spinach and Broccoli) exploit photoisomerizable donor‐acceptor fluorogens [29, 30, 32, 33].

### 3.2 A Labeling Mixture Strategy to Generate Reversibly Photoswitchable and Photoconvertible Fluorescent Protein Tags

Fluorogen mixtures have never simultaneously been used for labeling a chemogenetic system. Here, we benefited from the **FAST** promiscuity [34, 37, 38] and harnessed a mixture of two fluorogens emitting fluorescence in distinct wavelength ranges to tailor the color of their fluorescent complexes by implementing the principles of additive color synthesis. Interestingly, the collection of **FAST‐**Fluorogen complexes delimits a large portion of the CIE 1931 color space, thereby establishing high potential to generate a variety of labeling colors.

We jumped one step further by exploiting such fluorogen cocktails to expand the state‐of‐the‐art of reversibly photoswitchable fluorescent protein tags. Hence, we illustrated that a proper choice of the fluorogen pair converts at will the same labeling tag into reversible negative or positive photoswitchers, as well as fluorescent proteins reversibly photoconverting from one color to another within a large palette.

Eventually, these reversible photoswitching or photoconversion of the **FAST** fluorescence are notably transient, with thermal recovery of the initial state occurring on a few tens‐of‐seconds time scale, a range shorter than most regular RSFPs [11]. Whereas such a short range may be limiting for labeling a target within a population, it may be favorable for other applications (e.g., to apply multiplexed imaging protocols like OPIOM [50]).

### 3.3 Rich Photocycles for Dynamic Contrast

Any photocycle can be harnessed to kinetically discriminate reversibly photoactivatable fluorophores with protocols of dynamic contrast [7, 8, 9, 10, 11, 12, 13, 14, 15, 16, 17]. However, its efficiency depends on the number of its light‐driven and thermal steps and the span of its thermokinetic constants. Regular RSFPs exhibit rich photocycles involving multiple steps of light‐driven and thermal isomerization, proton exchange, and formation of triplet states [20, 21, 22]. Such a network of reactions is further expanded in **RCFASTs** by adding steps of association and dissociation of the fluorogen with the labeling tag, where the fluorogen concentration acts as a control parameter for tuning the rate of fluorogen recombination. For the purpose of multiplexed imaging, beyond their promiscuity and availability of a large collection of spectrally distinct fluorogens, **RCFASTs** will benefit from the small size of the **FAST** tag (14 kDa), which should limit the metabolic cost associated with expanding the proteins to be imaged with labeling fluorescent tags.

### 3.4 Enriching Dynamic Contrast With Colors

Discrimination of fluorophores is generally achieved either in the spectral or in the temporal domain. Here, we illustrated that both discriminative dimensions can be fruitfully combined. When using two differently colored fluorogens, **RCFASTs** offer multiple control parameters (e.g. fluorogen concentrations, wavelengths of excitation and fluorescence collection) and observables (e.g. fluorescence levels in several wavelength channels) to exploit a rich kinetic set and generate discriminative fingerprints for highly multiplexed imaging [39, 51].

### 3.5 Optimizing the Fluorogen Mixture

Yet, this richness has also counterparts for optimizing dynamic contrast with a pair of fluorogens. In the regime of photoejection that is used in the present manuscript, we previously concluded that: [34] (i) fluorogen photoisomerization essentially occurs within its bound state without involving any reactive process external to the **FAST** cavity (see Section S4.4.3 of the reference [34]); (ii) in contrast, thermal recovery after photoisomerization mostly involves fluorogen back isomerization external to the **FAST** cavity (see Section S4.5.2 of the reference [34]). This conclusion is important for optimizing the amplitudes of the fluorescence changes upon illumination. When using one fluorogen only, the rule of thumb is simple: the amplitude of photoejection drops when fluorogen concentration is increased, as observed in Figure 3.

Optimization is more delicate when using two fluorogens. We here report quantitative information on absorption and fluorescence emission spectra of the free fluorogens and of their **FAST** complexes. Hence, choosing the ranges of excitation and emission wavelengths for driving photoisomerization and reading out the time evolution of the fluorescence levels is easy. We also report the dissociation constant of the complexes of the fluorogens with **pFAST** and **nirFAST**. In vitro, such information is sufficient for maximizing the amplitude of the fluorescence change under illumination: the concentrations of both fluorogens should be fixed to their respective dissociation constant. However, for biological applications, we have evidenced interaction with endogenous cellular components, which makes this rule hazardous. Capitalizing on an intracellular concentration of the free fluorogen state previously estimated 10 to 10^2^ times lower than the extracellular one [34], it is reasonable to explore extracellularly applied fluorogen concentrations ranging between a tenth and a few µm for optimization.

## 4 Conclusion

We demonstrated that **FAST**‐based chemogenetic labels can be robustly converted into a broad family of reversible photoswitching and photoconvertible tags by controlling fluorogen concentration, illumination intensity, and by using one or two fluorogens. At submicromolar extracellular fluorogen concentration and illumination encountered in most fluorescence microscopies, the **FAST**‐fluorogen complexes undergo photoejection of the photoisomerized fluorogen, thereby delivering reversible negative photoswitchers emitting fluorescence across a wide spectral range. Combining two fluorogens further expands their scope by enabling reversible positive photoswitching and photoconversion. In particular, we used the latter configuration to implement dual‐color dynamic contrast. Hence, we generated distinct, time‑dependent spectral signatures to discriminate spectrally similar **FAST** variants in live cells, demonstrating that combining two spectral channels improves classification performance.

## Author Contributions

Conceptualization: M. M., Y. S., A. L., I. A., A. G., L. J.; Methodology: M. M., Y. S., A. L., I. A., A. G., L. J.; Software: Y. S., A. L.; Validation: M. M., Y. S., A. L., I. A., T. L. S., A. G., L. J.; Formal analysis: M. M., Y. S., A. L., F. P., I. C., T. L. S., L. J.; Investigation: M. M., Y. S., E. L.; Resources: M. M., Y. S., A. L., F. P., L. E. H., I. C., M.‐A. P., I. A.; Data Curation: M. M., Y. S., A. L.; Writing – original draft: M. M., Y. S., A. L., L. J.; Writing – review & Editing: M. M., Y. S., I. A., A. G., L. J.; Visualization: M. M., Y. S., A. L., A. G., L. J.; Supervision: F. P., I. A., A. G., L. J.; Project administration: L. J.; Funding acquisition: L. J.

## Funding

This work was supported by the ANR (ANR‐22‐CE11‐0011).

## Conflicts of Interest

The authors declare competing interests. A patent application has been filed relating to aspects of the work described in this manuscript. Authors listed on the patent: M. M., Y. S., I. C., L. E. H., F. P., I. A., T. L. S., A. G., L. J.; F. P., A.G., and L. J. are co‐founders and hold equity in Twinkle Bioscience/The Twinkle Factory, a company commercializing the FAST technology.

## Supporting information




**Supporting File**: advs78041‐sup‐0001‐SuppMat.pdf.

## Data Availability

The datasets generated during and/or analyzed during the current study and that are not in the Supporting Information are available from the corresponding author on request.
