# Supplementary material for: Non‐Covalent Reversibly Photoconvertible Fluorescent Tags for Wash‐Free Protein Labeling
Source: Adv Sci (Weinh). 2026 Sep 29:e78041. Online ahead of print. doi: 10.1002/advs.78041 (PMC13624302; doi:10.1002/advs.78041)
Supplement: Supplementary file 1 [file ADVS-9999-e78041-s001.pdf]

Non-covalent reversibly photoconvertible fluorescent tags for wash-free protein labeling

Mrinal Mandal,[1,†,*], Yuriy Shpinov,[1,†,*] Aliénor Lahlou,[1,2,*] Fivos Pham,[1]
Lina El Hajji,[1] Ian Coghill,[1] Eve Laureau,[1] Marie-Aude Plamont,[1] Franck Perez,[3]
Thomas Le Saux,[1] Isabelle Aujard,[1,*] Arnaud Gautier,[1,*] Ludovic Jullien[1,*]
[1]Chimie Physique et Chimie du Vivant, Département de chimie, École normale supérieure,
PSL University, Sorbonne Université, CNRS, Paris, France
[2]Sony Computer Science Laboratories, Paris, France
[3]Institut Curie, Université PSL, CNRS UMR144, Paris, France
[†] Co-first authors.
[*]To whom correspondence should be addressed; E-mail:
Mrinal.Mandal@ens.psl.eu,
Yuriy.Shpinov@ens.psl.eu,
Alienor.Lahlou@ens.psl.eu,
Isabelle.Aujard@ens.psl.eu.
Arnaud.Gautier@sorbonne-universite.fr,
Ludovic.Jullien@ens.psl.eu.

September 21, 2026

Contents

1 Theory **5**
1.1 Concept for non-covalent reversibly photoswitchable and photoconvertible fluorescent tags . . . 5
1.2 Thermodynamic equilibrium in the absence of illumination . . . 5
1.2.1 Interaction between the protein scaffold and a single fluorogen . . . 5
1.2.2 Interaction between the protein scaffold and two competing fluorogens . . . 6
1.3 Response of a non-covalent reversibly photoswitchable fluorescent protein to light scanning in confocal fluorescence microscopy . . . 7
1.3.1 Point spread function . . . 7
1.3.2 Photon exposure in scanning microscopy . . . 7
1.3.2.1 Non-overlapping scan lines . . . 8
1.3.2.2 Overlapping scan lines . . . 8
1.3.3 Photoisomerization in the protein cavity . . . 8
1.3.4 Photoejection and recombination . . . 9
1.3.5 Repetition of the illumination sequence . . . 10
1.3.6 Fluorescence signal . . . 11
1.4 Retrieving information on the fluorescence hue from the detector signals . . . 11
1.4.1 Detector signals . . . 11
1.4.2 CIE coordinates . . . 11

2 Materials and Methods **12**
2.1 Syntheses of the fluorogens . . . 12
2.1.1 General information . . . 12

2.1.2 Synthesis protocols . . . 12
2.1.2.1 **(E)-5-(3-chloro-4-hydroxybenzylidene)-4-thioxothiazolidin-2-one 3a [HBIR3Cl]** . . 13
2.1.2.2 **(Z)-5-(3-cyano-4-hydroxybenzylidene)thiazolidine-2,4-one 3b [HBT3CN]** . . . 13
2.1.2.3 **(Z)-5-(3-cyano-4-hydroxybenzylidene)-2-iminothiazolidin-4-one 3c [HBP3CN]** . . 13
2.1.2.4 **(Z)-5-(3-cyano-4-hydroxybenzylidene)-2-thioxoimidazolidin-4-one 3d [HBTH3CN]** 14
2.1.2.5 **((Z)-5-((E)-3-(4-hydroxy-3-chlorophenyl)allylidene)-2 thioxothiazolidin-4-one 6a [HPAR3Cl].** . . . 15
2.1.2.6 **((Z)-5-((E)-3-(4-hydroxy-3-fluoro-5-methoxyphenyl)allylidene)-2 thioxothiazolidin-4-one 6b [HPAR3F5OM].** . . . 18
2.2 Production and purification of fluorescent proteins . . . 19
2.2.1 Plasmids . . . 19
2.2.2 Production and purification of **Dronpa-2** . . . 19
2.2.3 Production and purification of **pFAST** . . . 19
2.2.4 **nirFAST** expression in bacteria and purification . . . 21
2.2.5 Preparation and storage of the solutions . . . 21
2.3 Production of labeled mammalian cells . . . 21
2.4 Instruments . . . 21
2.4.1 UV/Vis absorption and fluorescence spectrometers . . . 21
2.4.2 Light sources . . . 22
2.4.3 pH measurements . . . 22
2.4.4 Confocal microscopes . . . 22
2.4.5 Home-built microscope for patterned illumination . . . 22
2.5 Methods . . . 22
2.5.1 Measurement of light intensity . . . 22
2.5.1.1 Cuvette experiments . . . 22
2.5.1.2 Confocal microscopes . . . 23
2.5.2 Calibration of the detector sensitivity in confocal microscopy . . . 23
2.5.3 Methods for acquisition of the thermokinetic and photophysical parameters of the free fluorogens 24
2.5.3.1 Measurement of $K_{\mathbf{FH}}$ . . . 24
2.5.3.2 Measurement of $k^{\Delta}_{FF'} + k^{\Delta}_{F'F}$ . . . 24
2.5.3.3 Measurement of $\sigma_{FF'} + \sigma_{F'F}$ . . . 24
2.5.4 Methods for acquisition of the thermokinetic and photophysical parameters of the complexes between **pFAST** or **nirFAST** and the fluorogens . . . 24
2.5.4.1 Measurement of the quantum yield of fluorescence emission . . . 24
2.5.4.2 Measurement of the thermodynamic dissociation constant of the complex between **pFAST** or **nirFAST** and the thermodynamically stable state of the fluorogen (K_d) . . . 25
2.5.4.3 Measurement of k_{on}, k_{off} . . . 25
2.5.4.4 Measurement of $k^{\Delta}_{BB'} + k^{\Delta}_{B'B}$ . . . 26
2.5.4.5 Measurement of $k^{\Delta}_{BB'}$ and $k^{\Delta}_{B'B}$ . . . 26
2.5.4.6 Measurement of $\sigma_{BB'} + \sigma_{B'B}$ . . . 27
2.5.4.7 Measurement of $Q'_B/Q_{B'}, \sigma_{BB'}$, and $\sigma_{B'B}$ . . . 27
2.5.4.8 Measurement of K'_d . . . 28
2.5.5 Image segmentation and extraction of single-cell kinetic profiles . . . 28
2.5.5.1 Primary cell segmentation . . . 28
2.5.5.2 Sub-segmentation of cells into spatial micro-regions . . . 28
2.5.5.3 Extraction and normalization of fluorescence kinetics . . . 28
2.5.5.4 Train–test partitioning based on whole cells . . . 29
2.5.5.5 Cross-validation and model selection . . . 29
2.5.5.6 Final model training and evaluation . . . 29

3 Investigation of the free fluorogens 29
3.1 Absorption and emission spectra of the free fluorogens . . . 29
3.2 Measurement of the effective photoisomerization cross section of the free fluorogens . . . 33
3.2.1 Measurements at 365 nm . . . 33
3.2.2 Measurements at 405 nm . . . 36
3.2.3 Measurements at 480 nm . . . 40
3.3 Kinetics of thermal recovery from the photoisomerized state of the free fluorogen . . . 43
3.4 Measurement of the proton exchange constants of the free fluorogens . . . 50

4 Investigation of the pFAST-fluorogen and nirFAST-fluorogen complexes 53
4.1 Absorption and emission spectra . . . 53
4.2 Measurement of the thermodynamic dissociation constant of the complex between **pFAST** or **nirFAST** and the thermodynamically stable state of the fluorogen (K_d) . . . 59
4.2.1 Complexes with **pFAST** . . . 59
4.2.1.1 **HBR3Cl**, **HBR3Cl5F**, **HBR35DF**, **HBR3M**, **HBR25DM** . . . 59
4.2.1.2 **HBR3F5OM**, **HBR35DOM**, **HBT35DM** . . . 59
4.2.1.3 **HBR3CN** . . . 63
4.2.1.4 **HBIR3Cl** . . . 64
4.2.2 Complexes with **nirFAST** . . . 65
4.2.2.1 **HPAR3Cl**, **HBR3F5OM** . . . 65
4.2.2.2 **HBR3Cl**, **HBR35DOM**, **HPAR3F5OM**, **HPAR3OM**, **HPAR35DOM** . . . 66
4.2.2.3 **HBIR3Cl** . . . 66
4.3 Evaluation of the photoswitching robustness of **pFAST-HBR3Cl** . . . 70
4.4 Reference trajectory associated to replacing **HBT35DM** by **HBR35DOM** in **pFAST** in the CIE 1931 color space . . . 72

5 Confocal microscopy experiments 73
5.1 Experiments involving one fluorogen . . . 73
5.1.1 Labeling of live Hela cells . . . 73
5.1.1.1 At the nucleus . . . 73
Photoejection of **HBR25DM** . . . 73
Photoejection of **HPAR3Cl** . . . 73
5.1.1.2 At mitochondria . . . 73
Photoejection in **pFAST-HBR3F5OM** . . . 73
Photoejection in **nirFAST-HPAR3Cl** . . . 74
5.1.1.3 At the membrane . . . 74
Photoejection in **pFAST-HBR3F5OM** . . . 75
5.1.2 Labeling of live U2OS cells . . . 76
5.1.2.1 At the nucleus . . . 76
5.1.2.2 At mitochondria . . . 76
5.1.2.3 At the membrane . . . 76
5.1.3 Benchmarking of **RSpFAST** for photoejection . . . 76
5.2 Experiments involving two fluorogens . . . 78
5.2.1 Labeling of live Hela cells . . . 78
5.2.1.1 At the nucleus . . . 78
HBR3Cl:HBR35DOM . . . 78
HPAR3OM:HBR35DOM . . . 78
HBR3F5OM:HPAR3OM . . . 78
5.2.2 Labeling of live U2OS cells . . . 78
5.2.2.1 At the nucleus . . . 79

5.2.2.2 At mitochondria 79
5.2.2.3 At the membrane 80
5.3 Multiplexed imaging experiments 80
5.3.1 Mixture of living Hela cells labeled at the nucleus and at mitochondria 81
5.3.2 Living U2OS cells doubly labeled at the nucleus and at mitochondria 83
5.4 Comparative analyses in relation to multiplexed imaging 85
5.5 Experimental conditions for acquiring the images of the Main Text and the Supporting Information 88

1 Theory

1.1 Concept for non-covalent reversibly photoswitchable and photoconvertible fluorescent tags

In the following, we address several theoretical issues necessitated for the design and the implementation of our concept for accessing non-covalent reversibly photoswitchable and photoconvertible fluorescent tags with thermal recovery for wash-free protein labeling (Figure S1).

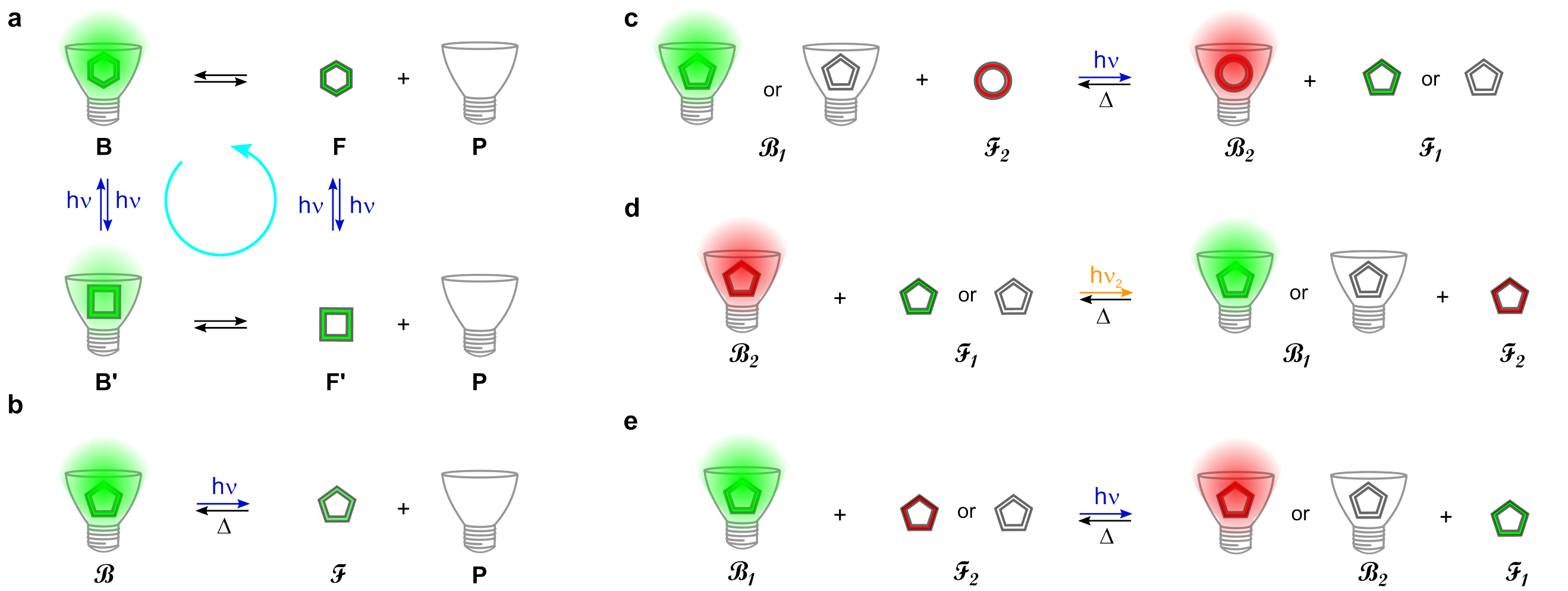

Figure S1: *Principle of non-covalent reversibly photoswitchable and photoconvertible fluorescent tags with thermal recovery for wash-free protein labeling.* **a**,**b**: The four-state photocycle of **FAST**-based ncRSFPs (**a**) yields a two-state reversible negative photoswitcher in the kinetic regime of photoejection (**b**); **c**–**e**: Combining a reversibly photoisomerizable fluorogen giving either a bright or dark complex and a non-photoisomerizable fluorogen (**c**), or a photoisomerizable fluorogen giving a bright complex with another photoisomerizable one giving either a bright or a dark complex of different color (**d**,**e**), provides a reversibly photoconvertible fluorescent protein (**c**,**d**,**e**), a reversible positive photoswitcher (**c**), or an enhanced reversible negative photoswitcher (**d**). In **c**–**e**, hν_1 and hν_2 refer to illumination at λ_1 and λ_2 in the absorption band of the complexes $\mathcal{B}_1$ and $\mathcal{B}_2$ respectively, where $\lambda_1 \leq \lambda_2$.

1.2 Thermodynamic equilibrium in the absence of illumination

1.2.1 Interaction between the protein scaffold and a single fluorogen

In this subsection, we consider that the fluorogen and the **FAST** protein scaffold interact to provide a fluorescent complex in the absence of illumination. One correspondingly adopts the thermodynamic model displayed in Figure S2 to analyze the thermodynamics of the interaction where the thermodynamic constant of association of **F** and **P** is denoted K and

F+P=B

Figure S2: *Thermodynamic model of the* ***FAST-Fluorogen*** *interaction.*

the dissociation constant of the complex **B** is $K_d = 1/K$. Noting F_{tot} and P_{tot} the total concentrations of **F** and **P**, the equilibrium concentrations $F^{\Delta}(\infty)$, $P^{\Delta}(\infty)$, and $B^{\Delta}(\infty)$ of the three species **F**, **P**, and **B** are

$$B^{\Delta}(\infty) = \frac{[K(F_{tot}+P_{tot})+1] - \sqrt{[K(F_{tot}+P_{tot})+1]^2 - 4K^2 F_{tot} P_{tot}}}{2K} \quad \text{(S1)}$$

$$F^{\Delta}(\infty) = F_{tot} - B(\infty) \quad \text{(S2)}$$

$$P^{\Delta}(\infty) = P_{tot} - B(\infty) \quad \text{(S3)}$$

The latter expression can be simplified when one component is in excess over the other:

- When $F_{tot} \gg P_{tot}$

$$B^{\Delta}(\infty) = \frac{KF_{tot}}{1+KF_{tot}} P_{tot} \tag{S4}$$

$$F^{\Delta}(\infty) = F_{tot} \tag{S5}$$

$$P^{\Delta}(\infty) = \frac{1}{1+KF_{tot}} P_{tot} \tag{S6}$$

- When $F_{tot} \ll P_{tot}$

$$B^{\Delta}(\infty) = \frac{KP_{tot}}{1+KP_{tot}} F_{tot} \tag{S7}$$

$$F^{\Delta}(\infty) = \frac{1}{1+KP_{tot}} F_{tot} \tag{S8}$$

$$P^{\Delta}(\infty) = P_{tot} \tag{S9}$$

Neglecting the brightness of the empty protein scaffold **P**, fluorescence intensity I_F given in Eq.(S10) results from the contributions of the fluorogen **F** and its protein complex **B** with respective brightnesses Q_F and Q_B

$$I_F = Q_F F^{\Delta}(\infty) + Q_B B^{\Delta}(\infty) \tag{S10}$$

1.2.2 Interaction between the protein scaffold and two competing fluorogens

We now consider a mixture that involves a protein scaffold interacting with two competing fluorogens. We correspondingly adopted the thermodynamic model displayed in Figure S3 to analyze the thermodynamics of the interaction where the thermodynamic constant of association of $\mathbf{F}_i$ and **P** is denoted K_i and the dissociation constant of the complex **B** is $K_{d,i} = 1/K_i$.

$\mathbf{F}_1+\mathbf{P}=\mathbf{B}_1$

$\mathbf{F}_2+\mathbf{P}=\mathbf{B}_2$

Figure S3: *Thermodynamic model of the interaction of **FAST** with two competing fluorogens.*

In the most general case, there is no analytic expression of the concentrations $F_1(\infty)$, $B_1(\infty)$, $F_2(\infty)$, and $B_2(\infty)$ at thermodynamic equilibrium. In order to overcome this limitation, we hypothesize that the experimental conditions are chosen such that $P \ll B_1 + B_2$. Then the system displayed in Figure S3 can be simplified to the thermodynamic model shown in Figure S4.

$\mathbf{F}_2+\mathbf{B}_1=\mathbf{B}_2+\mathbf{F}_1$

Figure S4: *Simplified thermodynamic model of the interaction of **pFAST** with two competing fluorogens.*

Denoting $F_{1,tot}$, $F_{2,tot}$, and P_{tot} the total concentrations of $\mathbf{F}_1$, $\mathbf{F}_2$, and **P**, one then obtains the expressions (S11–S14) of the concentrations $F_1^{\Delta}(\infty)$, $B_1^{\Delta}(\infty)$, $F_2^{\Delta}(\infty)$, and $B_2^{\Delta}(\infty)$ at thermodynamic equilibrium.

$$B_1^{\Delta}(\infty) = \frac{-[(1-K)P_{tot} + KF_{2,tot} + F_{1,tot}] + \sqrt{[(1-K)P_{tot} + KF_{2,tot} + F_{1,tot}]^2 + 4(K-1)P_{tot}F_{1,tot}}}{2(K-1)} \tag{S11}$$

$$F_1^{\Delta}(\infty) = F_{1,tot} - B_1^{\Delta}(\infty) \tag{S12}$$

$$B_2^{\Delta}(\infty) = P_{tot} - B_1^{\Delta}(\infty) \tag{S13}$$

$$F_2^{\Delta}(\infty) = F_{2,tot} - P_{tot} + B_1^{\Delta}(\infty) \tag{S14}$$

where $K = \frac{K_2}{K_1}$.

Neglecting the brightness of the free fluorogens with respect to the brightness of their complexes with the protein, the fluorescence intensity from the system is given in Eq.(S15)

$$I_F(\infty) = Q_{B_1} B_1^{\Delta}(\infty) + Q_{B_2} B_2^{\Delta}(\infty) = Q_{B_2} P_{tot} + (Q_{B_1} - Q_{B_2}) B_1^{\Delta}(\infty) \tag{S15}$$

1.3 Response of a non-covalent reversibly photoswitchable fluorescent protein to light scanning in confocal fluorescence microscopy

Confocal fluorescence microscopy works by rapidly scanning a focused laser beam across the imaging area. Therefore, the illumination can no longer be considered constant and homogeneous. Then, the scanning parameters need to be considered for an accurate model of the evolution of the fluorescence signal from a non-covalent reversibly photoswitchable fluorescent protein at each pixel as a function of the number of scans/illumination time.

1.3.1 Point spread function

In an inverted fluorescence microscope with a laser light source, the excitation and emission point spread functions (PSF) have non-trivial spatial distributions, but near the focus they can be approximated by Gaussian functions $\phi(r, w)$ with radial widths $w(z)$ dependent on the axial distance z from the beam waist.

$$\begin{aligned} PSF_{ex}(r,z) &= \frac{w_{ex}^2(0)}{w_{ex}^2(z)}\,\phi(r, w_{ex}(z)) \\ PSF_{em}(r,z) &= \frac{w_{em}^2(0)}{w_{em}^2(z)}\,\phi(r, w_{em}(z)) \\ \phi(r,w) &= e^{-2r^2/w^2} \\ w(z) &= w_0\sqrt{1+\left(\frac{\lambda z}{\pi w_0^2}\right)} \end{aligned} \tag{S16}$$

where λ is the light wavelength, r is the radial distance from the axis of illumination in a given z plane and w_0 is the radial beam waist (minimal beam radius obtained at the focus, defined at the radial distance at which light intensity is $1/e^2$ of the peak). The PSF as defined here are normalized by the peak light intensity I_0 at the focal point such that $I(r, z) = I_0 PSF(r, z)$.

In a confocal microscope, the emitted light is additionally passed through a pinhole before reaching the detector. Therefore, when a pinhole is employed, the detection PSF is narrower than the emission PSF, especially in the axial direction. The axial intensity follows a sinc function, but for small pinhole diameters (≤ 1 Airy), it can be roughly approximated by a Gaussian with beam radius w_{ax}.

$$\begin{aligned} PSF_{det}(r,z) &\approx \phi(r, w_0)\phi(z, w_{ax}) \\ w_{ax} &\approx \frac{3.47n}{NA} w_0 \end{aligned} \tag{S17}$$

where n is the refractive index of the imaging medium and NA is the numerical aperture of the objective.

The final confocal point spread function $PSF_{conf}(r, z)$ is the product of the excitation and detection PSF. However, if the pinhole is not used (“widefield” conditions), the point spread function is $PSF_{wf}(r, z)$

$$\begin{aligned} PSF_{conf}(r,z) &= PSF_{ex}(r,z)\cdot PSF_{det}(r,z) \\ PSF_{wf}(r,z) &= PSF_{ex}(r,z)\cdot PSF_{em}(r,z) \end{aligned} \tag{S18}$$

1.3.2 Photon exposure in scanning microscopy

In order to image an area, the laser beam is scanned across the sample row by row at a constant speed v, which can be calculated from the dwell time t_d and the horizontal pixel size Δx given by the instrument.

$$v = \Delta x / t_d \tag{S19}$$

We first consider the illumination from a single horizontal scanline at $y = 0$, with the focal plane at $z = 0$. To calculate the total photon exposure H_0 (in E·m^{-2})[a] received at a given position (x, y, z), we need to integrate the illumination PSF as it travels across the width l of the sample. The total width is much larger than the width of the PSF, so we can consider it infinite if we discard the pixels at the edge of the image.

$$
\begin{aligned}
H_0(y,z) &= \int_{-l/2}^{l/2} \frac{I_0 PSF_{ex}(r,z)dx}{v} \approx \frac{I_0}{v}\int_{-\infty}^{+\infty} \frac{w_{ex}^2(0)\phi\left(\sqrt{x^2+y^2}, w_{ex}(z)\right)dx}{w_{ex}^2(z)} \\
&= \frac{I_0 w_{ex}^2(0)}{w_{ex}^2(z)v}\int_{-\infty}^{+\infty} e^{-2(x^2+y^2)/w_{ex}^2(z)}dx = \frac{\sqrt{\pi} I_0 w_{ex}^2(0) e^{-2y^2/w_{ex}^2(z)}}{\sqrt{2}w_{ex}(z)v} \\
&= \frac{\sqrt{\pi} w_{ex}^2(0)}{\sqrt{2}\Delta x w_{ex}(z)} I_0 t_d \phi(y, w_{ex}(z)) = \frac{\sqrt{\pi} w_{ex}(z)}{\sqrt{2}\Delta x} I_0 t_d PSF_{ex}(y,z)
\end{aligned}
\tag{S20}
$$

The resulting equation shows that the amount of light received is homogeneous along the x axis. Moreover a scaling factor $\frac{\sqrt{\pi} w_{ex}(z)}{\sqrt{2}\Delta x}$ dependent on the beam width and pixel size appears in addition to the "naive" expression of photon exposure $H_0 = I_0 t_d PSF_{ex}$.

An important parameter is the vertical inter-line distance Δy. Small values of Δy cause the illumination PSF to partially overlap, exposing again the same area of the sample to the laser.

1.3.2.1 Non-overlapping scan lines We consider that the scan lines do not overlap, when $\Delta y \geq 3w_{ex}$. In that case, the total photon exposure H is equal to H_0.

1.3.2.2 Overlapping scan lines On the other hand, if $\Delta y < w_{ex}$, the scan lines lie close to one another and the cumulative photon exposure H can be calculated by adding up the contributions of all N_y scan lines. Since $N_y \gg 1$, we can approximate the number of lines as being infinite if the lines near the edges of the image are excluded.

$$
H = \sum_{n=1}^{N_y} H_0(y - n\Delta y, z) \approx \sum_{n\in\mathbb{Z}} H_0(y + n\Delta y, z) \tag{S21}
$$

If $\Delta y \ll w_{ex}$, we can approximate further by considering that the overlapping lines form a continuum with a line density of $1/\Delta y$.

$$
\begin{aligned}
H &\approx \int_{-\infty}^{+\infty} \frac{H_0(y,z)}{\Delta y} dy = \frac{\sqrt{\pi} I_0 w_{ex}^2(0)}{\sqrt{2}\Delta x \Delta y w_{ex}(z)} \int_{-\infty}^{+\infty} e^{-2y^2/w_{ex}^2(z)} dy \\
&= \frac{\pi w_{ex}^2(0)}{2\Delta x \Delta y} I_0 t_d
\end{aligned}
\tag{S22}
$$

In this case, the photon exposure is homogeneous in space within the limits of the image area.

1.3.3 Photoisomerization in the protein cavity

The photoswitching and complexation of a fluorogen in the presence of **FAST** can be described with the 4-state model shown in Figure S5, which includes thermal and light-driven processes.

Illumination drives activation of a photocycle: (i) since **FAST P** has been selectively evolved to yield a complex **B** with the thermodynamically stable fluorogen configuration **F**, the resulting less stable complex **B'** between the photoisomerized fluorogen **F'** and **FAST P** would disrupt, thereby liberating free **FAST P** and the photoisomerized fluorogen **F'**; (ii) the latter would subsequently experience back photoisomerization to its thermodynamically stable state **F** and (iii) eventually regenerate the initial **FAST**-fluorogen complex **B**.

[a] In this manuscript, we provide the values of the light intensities in E.m^{-2}.s^{-1} (or mol. of photons.m^{-2}.s^{-1}). This unit is currently used in actinometry. However, it is not often used in other fields such as optical microscopy, in which the researchers prefer to adopt W.m^{-2}. We provide the conversion in Eq.(S43).

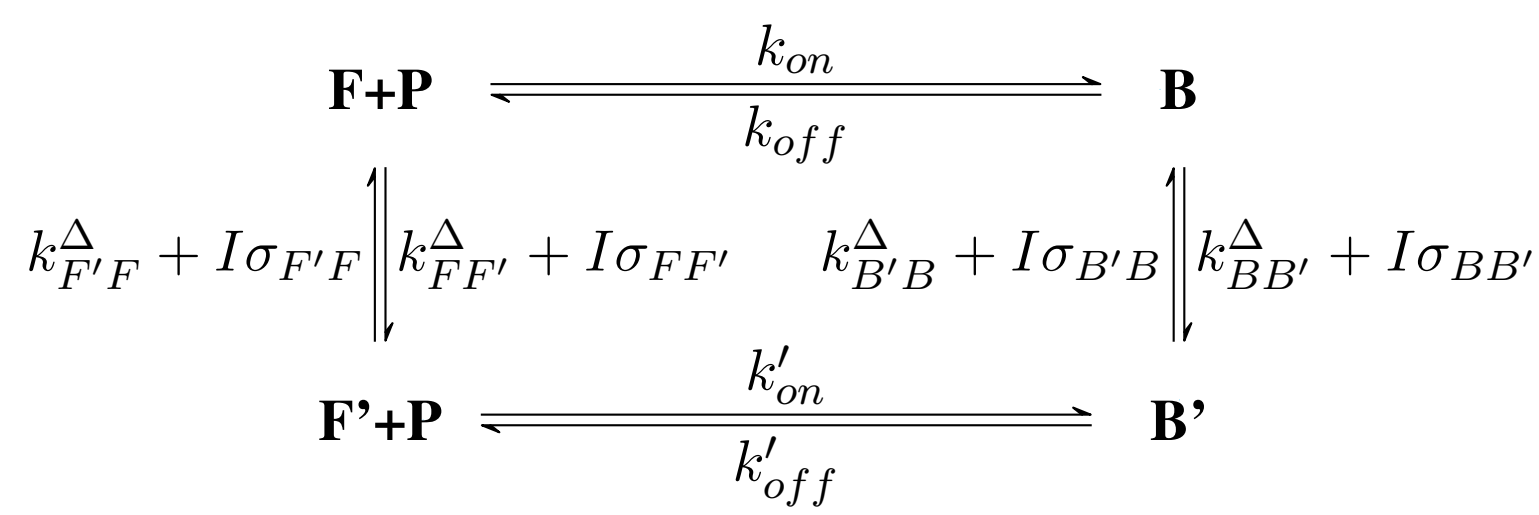

Figure S5: *Four-state model of the **FAST-Fluorogen** photocycle*. **F**, **F'**, **P**, **B** and **B'** represent free (*Z*)-**fluorogen**, free (*E*)-**fluorogen**, free **FAST** protein, (*Z*)-**fluorogen**:**FAST** complex, and (*E*)-**fluorogen**:**FAST** complex respectively. The thermal rate constants are denoted as k^{Δ} while σ denotes a cross-section of photoswitching. k_{on}, k_{off}, k'_{on} and k'_{off} represent the on- and off-rate constants associated with the formation of the complexes **B** and **B'** respectively. Light intensity is denoted as I.

Considering the dwell time in confocal microscopy to be on the order of the microsecond, photoisomerization is the only reaction that can take place at this timescale in the **FAST**:**Fluorogen** system. The concentrations of **B** and **B'** at a given pixel after they were illuminated by the beam, B_{1a} and B'_{1a} respectively, can be calculated upon considering that only the thermodynamically stable isomer **B** is initially present. Then

$$\begin{aligned} B_{1a} &= B_0(a + (1-a)\alpha) = B_0 A \\ B'_{1a} &= B_0((1-a) - (1-a)\alpha) = B_0(1-A) \end{aligned} \tag{S23}$$

where B_0 is the initial concentration of **B** and

$$\begin{aligned} \alpha &= e^{-H\sigma} \\ \sigma &= \sigma_{BB'} + \sigma_{B'B} \\ a &= \frac{\sigma_{B'B}}{\sigma_{BB'} + \sigma_{B'B}} \\ A &= a + (1-a)\alpha \end{aligned} \tag{S24}$$

1.3.4 Photoejection and recombination

After one full image is scanned, the process is repeated. During the time t_f for frame acquisition, we chose experimental conditions such that photoejection and recombination of the fluorogen have enough time to take place. Then the concentrations of **B** and **B'** at a given pixel after this delay in the dark, B_1 and B'_1 respectively, can be calculated

$$\begin{aligned} B_1 &= b + (B_{1a} - b)\beta = B_{1a}\beta + b(1-\beta) \\ B'_1 &= b' + (B'_{1a} - b')\beta' \approx 0 \end{aligned} \tag{S25}$$

with

$$\begin{aligned} \beta &= e^{-(k_{on}F + k_{off})t_f} \\ \beta' &= e^{-(k'_{on}F' + k'_{off})t_f} \approx 0 \\ b &= \frac{FP_{tot}}{F + K_D} \approx P_{tot} = B_0 \\ b' &= \frac{F'P_{tot}}{F' + K'_D} \approx 0 \end{aligned} \tag{S26}$$

where K_D and K'_D refer to the dissociation constant of **B** and **B'**, and P_{tot} designates the total concentration in **FAST**. Considering that t_f is on the timescale of hundred of milliseconds for all the investigated fluorogens, $k'_{off}t_f \gg 1$, and consequently $\beta' \approx 0$. Moreover, under the concentration conditions for photoejection ($F' \ll K'_D$ and $F \gg K_D$), $b' \approx 0$ and $b \approx P_{tot} = B_0$. In other words, **B'** is completely ejected during the time between frames. Meanwhile $k_{on}F + k_{off}$ is small and **B** cannot reach its equilibrium concentration at the same time scale under those conditions.

1.3.5 Repetition of the illumination sequence

The sequence of illumination and dark delay is repeated until a stationary state is reached, which can be described using a recursive definition of B_n, the concentration of **B** after the nth cycle.

$$\begin{aligned} B_n &\approx B_{n,a}\beta + B_0(1-\beta) = B_{n-1}A\beta + B_0(1-\beta) \\ &= B_0\left[(A\beta)^n + (1-\beta)\sum_{k=0}^{n-1}(A\beta)^n\right] \\ &= B_0\left[(A\beta)^n + \frac{(1-\beta)(1-(A\beta)^n)}{1-A\beta}\right] \\ &= B_0[B^* + (1-B^*)(A\beta)^n] \end{aligned} \tag{S27}$$

$$B^* = \frac{1-\beta}{1-A\beta}$$

The concentration of **B** at the photostationary state B^* depends on light intensity and a first-order approximation can be made if $H\sigma \ll 1$.

$$\begin{aligned} B^* &= \frac{1-\beta}{1-\beta(a+(1-a)\alpha)} = \frac{1-\beta}{(1-a\beta)-\beta(1-a)e^{-H\sigma}} \\ &\approx \frac{1-\beta}{(1-a\beta)-\beta(1-a)(1-H\sigma)} = \frac{1}{1+\beta\frac{1-a}{1-\beta}\,H\sigma} \\ &\approx 1-\beta\frac{1-a}{1-\beta}\,H\sigma \end{aligned} \tag{S28}$$

At first order, the amplitude of photoejection is proportional to the photon exposure.
Transforming the equation for B_n, it becomes apparent that the function B_n obeys an exponential decay law with apparent kinetic rate constant of photoejection k_{PE}.

$$B_n = B_0[B^* + (1-B^*)(A\beta)^n] = B_0[B^* + (1-B^*)e^{-k_{PE}n}] \tag{S29}$$

with

$$k_{PE} = -\ln A\beta > 0 \tag{S30}$$

The rate of photoejection depends on photon exposure according to the relationship:

$$k_{PE} = -\ln A\beta = -\ln[\beta(a+(1-a)\alpha)] = -\ln\left[\beta(a+(1-a)e^{-H\sigma})\right] \tag{S31}$$

If $H\sigma \ll 1$, or in other words if the extent of photoisomerization during each illumination cycle is small, we can perform a first-order approximation of k_{PE}.

$$\begin{aligned} k_{PE} &= -\ln\beta - \ln A \\ &\approx -\ln\beta - \ln(a+(1-a)(1-H\sigma)) \\ &= -\ln\beta - \ln(1+(a-1)H\sigma) \\ &\approx (1-a)H\sigma - \ln\beta \\ &= (1-a)H\sigma + (k_{on}F + k_{off})t_f \end{aligned} \tag{S32}$$

It becomes apparent that the rate of photoejection depends at first order on the photon exposure (and thus on light intensity, dwell time and pixel size) as well as concentration of free fluorogen.

1.3.6 Fluorescence signal

Finally, we express the fluorescence signal I_F originating from a point at (x, y, z) and collected at a pixel with coordinates $(0, 0, 0)$ at frame n. The concentration of **B** and **B'** changes as the laser beam moves across the scanned line. However, if the light intensity and dwell time are such that that the extent of photoisomerization during one scan is small, $B_n(t)$ and $B'_n(t)$ remain approximately constant during the line scan. Furthermore, at the start of the scan **B** is the only fluorescent species present, so any contribution from **B'** can be ignored.

$$\begin{aligned} I_F &\approx I_0 Q_B B_n(t) PSF_{conf}(r, z) \\ &= I_0 Q_B B_0 \left[B^* + (1 - B^*) e^{-k_{PE} n} \right] PSF_{conf}(r, z) \end{aligned} \tag{S33}$$

1.4 Retrieving information on the fluorescence hue from the detector signals

1.4.1 Detector signals

We consider two fluorescent **FAST** complexes $\mathbf{B}_1$ and $\mathbf{B}_2$ with brightnesses $b_1(\lambda)$ and $b_2(\lambda)$ and concentrations C_1 and C_2 respectively, where λ is the emission wavelength and the brightnesses are taken with respect to the same excitation wavelength λ_{exc}. Upon excitation with light intensity I, the total fluorescence spectral intensity $I_F(\lambda)$ (in units of Ein·m^{-2}·s^{-1}·nm^{-1}) is the sum of the contribution from $\mathbf{B}_1$ and $\mathbf{B}_2$:

$$I_F(\lambda) = (C_1 b_1(\lambda) + C_2 b_2(\lambda)) I \tag{S34}$$

We consider the case when the fluorescence from the sample is detected in two channels. The detected fluorescence signals S_1 and S_2 depend on the fluorescence spectral intensity $I_F(\lambda)$ as well as the respective spectral sensitivities $s_1(\lambda)$ and $s_2(\lambda)$ of the two detection channels:

$$\begin{aligned} S_1 &= \int_\lambda I_F(\lambda) s_1(\lambda) d\lambda = I \int_\lambda (C_1 b_1(\lambda) + C_2 b_2(\lambda)) s_1(\lambda) d\lambda \\ S_2 &= \int_\lambda I_F(\lambda) s_2(\lambda) d\lambda = I \int_\lambda (C_1 b_1(\lambda) + C_2 b_2(\lambda)) s_2(\lambda) d\lambda \end{aligned} \tag{S35}$$

Rewriting in matrix form using the inner product notation $\langle \cdot, \cdot \rangle$

$$\langle f, g \rangle := \int_\lambda f(\lambda) g(\lambda) d\lambda \tag{S36}$$

$$\begin{bmatrix} S_1 \\ S_2 \end{bmatrix} = I \begin{bmatrix} \langle b_1, s_1 \rangle & \langle b_2, s_1 \rangle \\ \langle b_1, s_2 \rangle & \langle b_2, s_2 \rangle \end{bmatrix} \cdot \begin{bmatrix} C_1 \\ C_2 \end{bmatrix} \tag{S37}$$

1.4.2 CIE coordinates

The tristimulus coordinates X, Y, Z of a fluorescent (emissive) source with spectral power intensity E(λ) (in units of W·m^{-2}·nm^{-1}) in CIE 1983 space are:

$$\begin{bmatrix} X \\ Y \\ Z \end{bmatrix} = \begin{bmatrix} \langle E, \bar{x} \rangle \\ \langle E, \bar{y} \rangle \\ \langle E, \bar{z} \rangle \end{bmatrix} \tag{S38}$$

Where $\bar{x}(\lambda), \bar{y}(\lambda), \bar{z}(\lambda)$ are the standard 2° color matching functions. Since this definition expects units of spectral power, we have to adjust it to use the spectral intensity $I_F(\lambda)$:

$$\begin{bmatrix} X \\ Y \\ Z \end{bmatrix} = \begin{bmatrix} \langle I_F, \bar{x}' \rangle \\ \langle I_F, \bar{y}' \rangle \\ \langle I_F, \bar{z}' \rangle \end{bmatrix}$$

$$\begin{aligned} \bar{x}'(\lambda) &= \bar{x}(\lambda) hcN_A/\lambda \\ \bar{y}'(\lambda) &= \bar{y}(\lambda) hcN_A/\lambda \\ \bar{z}'(\lambda) &= \bar{z}(\lambda) hcN_A/\lambda \end{aligned} \tag{S39}$$

Expanding $I_F(\lambda)$ and rearranging:

$$\begin{bmatrix} X \\ Y \\ Z \end{bmatrix} = \begin{bmatrix} \langle b_1, \bar{x}' \rangle & \langle b_2, \bar{x}' \rangle \\ \langle b_1, \bar{y}' \rangle & \langle b_2, \bar{y}' \rangle \\ \langle b_1, \bar{z}' \rangle & \langle b_2, \bar{z}' \rangle \end{bmatrix} \cdot \begin{bmatrix} C_1 \\ C_2 \end{bmatrix} \tag{S40}$$

Combining with Eq.(S37), we derive an equation for the CIE X, Y, Z coordinates as a function of the observed detector signals S_1, S_2:

$$\begin{bmatrix} X \\ Y \\ Z \end{bmatrix} = \begin{bmatrix} \langle b_1, \bar{x}' \rangle & \langle b_2, \bar{x}' \rangle \\ \langle b_1, \bar{y}' \rangle & \langle b_2, \bar{y}' \rangle \\ \langle b_1, \bar{z}' \rangle & \langle b_2, \bar{z}' \rangle \end{bmatrix} \cdot \begin{bmatrix} \langle b_1, s_1 \rangle & \langle b_2, s_1 \rangle \\ \langle b_1, s_2 \rangle & \langle b_2, s_2 \rangle \end{bmatrix}^{-1} \cdot \begin{bmatrix} S_1 \\ S_2 \end{bmatrix} \tag{S41}$$

Finally, the CIE chromacity coordinates x, y can be calculated from the X, Y, Z coordinates:

$$\begin{bmatrix} x \\ y \end{bmatrix} = \frac{1}{X + Y + Z} \begin{bmatrix} X \\ Y \end{bmatrix} \tag{S42}$$

2 Materials and Methods

2.1 Syntheses of the fluorogens

2.1.1 General information

3-Chloro-4-hydroxybenzaldehyde, pseudothiohydantoin was purchased from Sigma Aldrich. 5-formyl-2- hydroxybenzonitrile was purchased from BLDpharm. 3-fluoro-4-hydroxy-5-methoxybenzaldehyde were purchased from abcr GmbH. Rhodanine, isorhodanine, 2,4-thiazolidinedione and thiohydantoin were purchased from Acros Organics. Commercially available reagents were used as obtained. ^{1}H and ^{13}C NMR spectra were recorded at 300 K on a Bruker AM 300 spectrometer; chemical shifts are reported in ppm with protonated solvent as internal reference (^{1}H, CHD_2SOCD_3 in CD_3SOCD_3 2.50 ppm; ^{13}C, $^{13}CD_3SOCD_3$ in CD_3SOCD_3 39.5 ppm, ^{19}F, $C_7H_5F_3$ in CHD_2SOCD_3 -63.7 ppm). Mass spectra high resolution were performed by the Service de Spectrométrie de Masse de Chimie ParisTech and the Service de Spectrométrie de Masse de l'Institut de Chimie Organique et Analytique (Orléans). Analytical thin-layer chromatography (TLC) was conducted on Merck silica gel 60 F254 pre-coated plates.

2.1.2 Synthesis protocols

The synthesis pathways are displayed in Scheme 1.

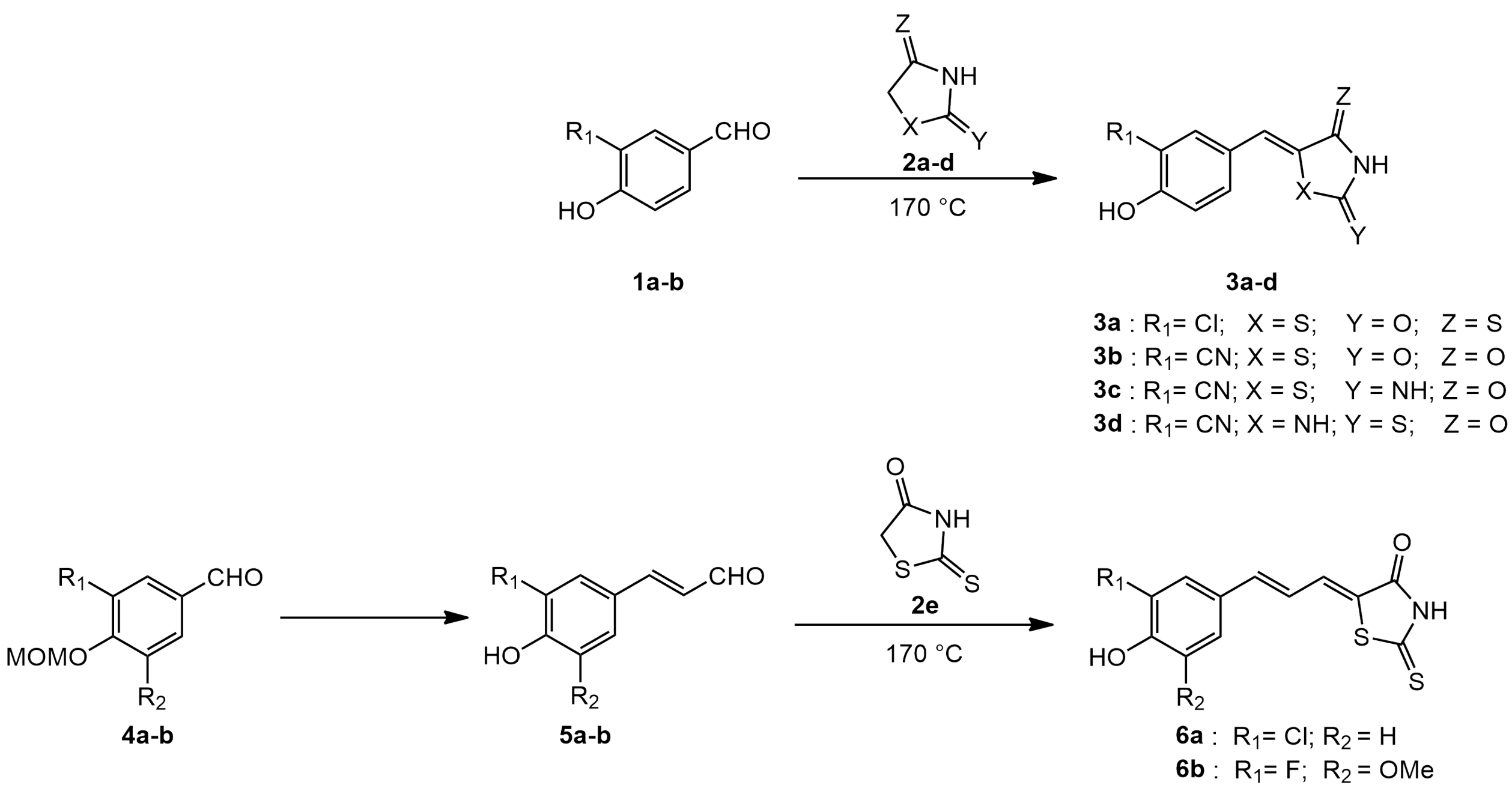

Scheme 1: Syntheses of the new fluorogens **3a-d** and **6a-b**.

2.1.2.1 (E)-5-(3-chloro-4-hydroxybenzylidene)-4-thioxothiazolidin-2-one 3a [HBIR3Cl] 4-Hydroxy-3-chloro benzaldehyde **1a** (140 mg, 0.9 mmol, 1.2 eq) and isorhodanine **2a** (100 mg, 0.75 mmol) were heated together at 170 °C. The mixture first fused and then solidified. After being cooled to 50–60 °C, the solid was stirred in ethanol at reflux until complete dissolution. Then an excess of water ($V_{H_2O} = 2\times V_{EtOH}$) was slowly added and a precipitate appeared. It was filtered, washed with water, and dried over P_2O_5. **HBIR3Cl** was obtained as an orange powder (58 %, 118 mg). **^{1}H NMR (300 MHz, DMSO-d$_6$, δ)**: 13.82 (br, 1H), 11.33 (br, 1H), 8.00 (s, 1H), 7.72 (d, J=3 Hz, 1H), 7.51(dd, J= 3, 9 Hz, 1H), 7.12 (d, J=9 Hz, 1H). **^{13}C NMR (75 MHz, DMSO-d$_6$, δ):** 195.1, 170.5, 155.9, 135.1, 133.0, 130.7, 127.5, 125.7, 120.9, 117.4. **HRMS (ESI):** m/z calc. for $C_{10}H_7ClNO_2S_2$: 271.9601, found: 271.9601 $[M+H]^+$.

2.1.2.2 (Z)-5-(3-cyano-4-hydroxybenzylidene)thiazolidine-2,4-one 3b [HBT3CN] Same as **HBIR3Cl** using 5-formyl-2-hydroxybenzonitrile **1b** (50 mg, 0.34 mmol) and 2,4-thiazolidinedione **2b** (47 mg, 0.40 mmol, 1.2 eq). **HBT3CN** was obtained as a dark red powder (65%, 54 mg). **^{1}H NMR (300 MHz, DMSO-d$_6$, δ):** 12.30 (s, br, 2H), 7.91 (d, J= 2.0 Hz, 1H), 7.72 (s, 1H), 7.69 (dd, J = 2.0, 8.8, 1H), 7.16 (d, J = 8.8 Hz, 1H). **^{13}C NMR (75 MHz, DMSO-d$_6$, δ):** 167.8, 167.4, 161.6, 136.3, 135.3, 130.1, 124.7, 121.9, 117.3, 116.2, 100.1. **HRMS (ESI):** m/z calc. for $C_{11}H_5N_2O_3S$: 245.0023, found: 245.0031 $[M-H]^-$.

2.1.2.3 (Z)-5-(3-cyano-4-hydroxybenzylidene)-2-iminothiazolidin-4-one 3c [HBP3CN] Same as **HBIR3Cl** using 5-formyl-2-hydroxybenzonitrile **1b** (50 mg, 0.34 mmol) and pseudothiohydantoin **2c** (46 mg, 0.40 mmol, 1.2 eq). **HBP3CN** was obtained as a dark red powder (36%, 30 mg). **^{1}H NMR (300 MHz, DMSO-d$_6$, δ):** 7.47 (d, J= 2.0, 1H), 7.36 (s, 1H), 7.31 (dd, J= 2.0, 9.0 Hz, 1H), 6.46 (d, J = 9.0 Hz, 1H). **^{13}C NMR (75 MHz, DMSO-d$_6$, δ):** 181.0, 175.0, 171.3, 136.3, 134.7, 129.7, 121.1, 120.8, 119.9, 117.1, 100.4. **HRMS (ESI):** m/z calc. for $C_{11}H_6N_3O_2S$: 244.0184, found: 244.0191 $[M-H]^-$.

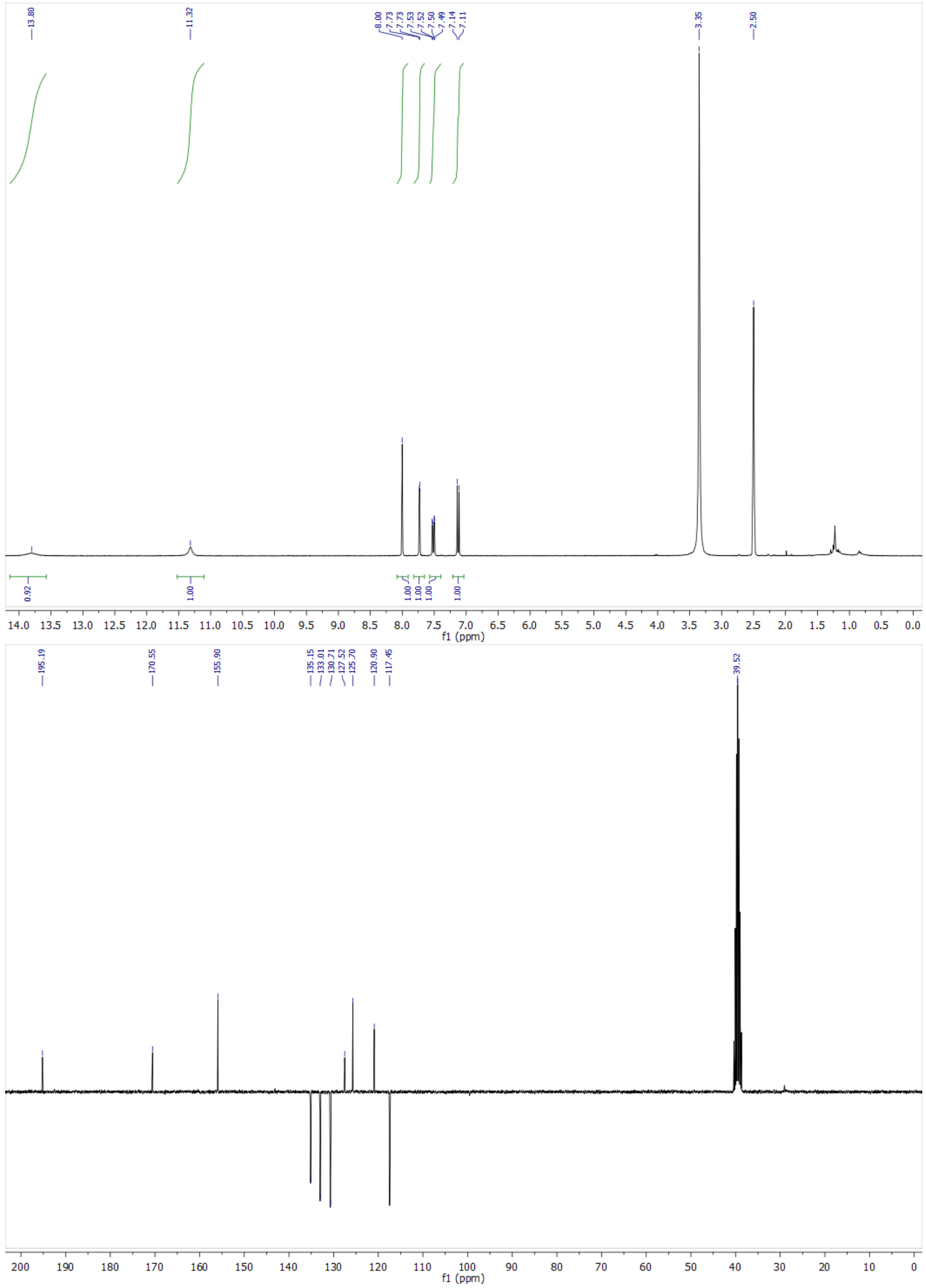

Figure S6: ^{1}H-NMR and ^{13}C-NMR spectra of **HBIR3Cl**.

2.1.2.4 (Z)-5-(3-cyano-4-hydroxybenzylidene)-2-thioxoimidazolidin-4-one 3d [HBTH3CN] Same as **HBIR3Cl** using 5-formyl-2-hydroxybenzonitrile **1b** (50 mg, 0.34 mmol) and thiohydantoin **2d** (46 mg, 0.40 mmol, 1.2 eq). **HBTH3CN** was obtained as a dark red powder (30%, 25 mg). **^{1}H NMR (300 MHz, DMSO-d$_6$, δ):** 12.3 (s, br, 3H), 8.1 (d, J = 2.0 Hz, 1H), 7.80 (dd, J= 2.0, 8.7 Hz, 1H), 7.02 (d, J = 8.7 Hz, 1H), 6.42 (s, 1H). **^{13}C NMR (75 MHz, DMSO-d$_6$, δ):** 178.9, 165.7, 161.2, 137.2, 134.5, 126.7, 123.8, 116.7, 116.6, 110.1, 99.8. **HRMS (ESI):** m/z calc. for $C_{11}H_6N_3O_2S$: 244.0181, found: 244.0188 [M-H]$^{-}$.

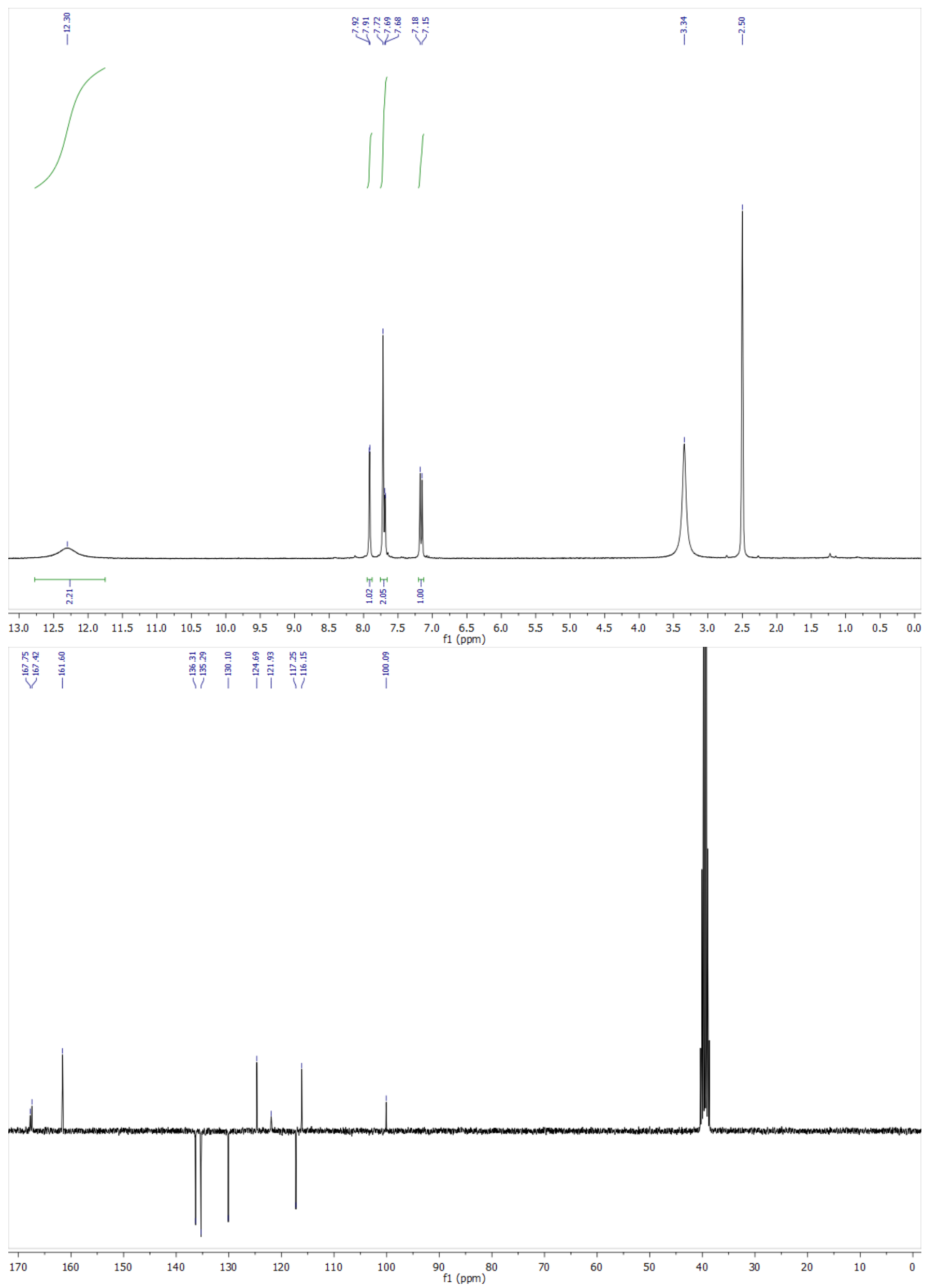

Figure S7: ^{1}H-NMR and ^{13}C-NMR spectra of **HBT3CN**.

2.1.2.5 ((Z)-5-((E)-3-(4-hydroxy-3-chlorophenyl)allylidene)-2 thioxothiazolidin-4-one 6a [HPAR3Cl]. 3-Chloro-4-(methoxymethoxy)benzaldehyde 4a To a stirred solution of 3-chloro-4-hydroxybenzaldehyde (500 mg, 3.2 mmol) in anhydrous dichloromethane (5 mL) at 0 °C was added diisopropylethylamine (1.1 mL, 6.4 mmol, 2 eq) and chloromethyl methyl ether (0.36 mL, 4.8 mmol, 1.5 eq). The mixture was stirred overnight at room temperature. The reaction was quenched with a saturated aqueous solution of ammonium chloride, and the aqueous layer was extracted with dichloromethane. The combined organic layers were washed with brine, dried over magnesium sulfate and concentrated in vacuo. The desired compound **4a** was obtained quantitatively as a pale yellow solid and used without further purification for the next step. **^{1}H NMR (300 MHz, acetone-d$_6$, δ):** 9.92 (s, 1H), 7.95 (d, J = 3 Hz, 1H), 7.87 (dd, J = 3 and 9 Hz, 1H), 7.43 (d, J = 9 Hz, 1H), 5.44 (s, 2H), 3.50 (s, 3H).

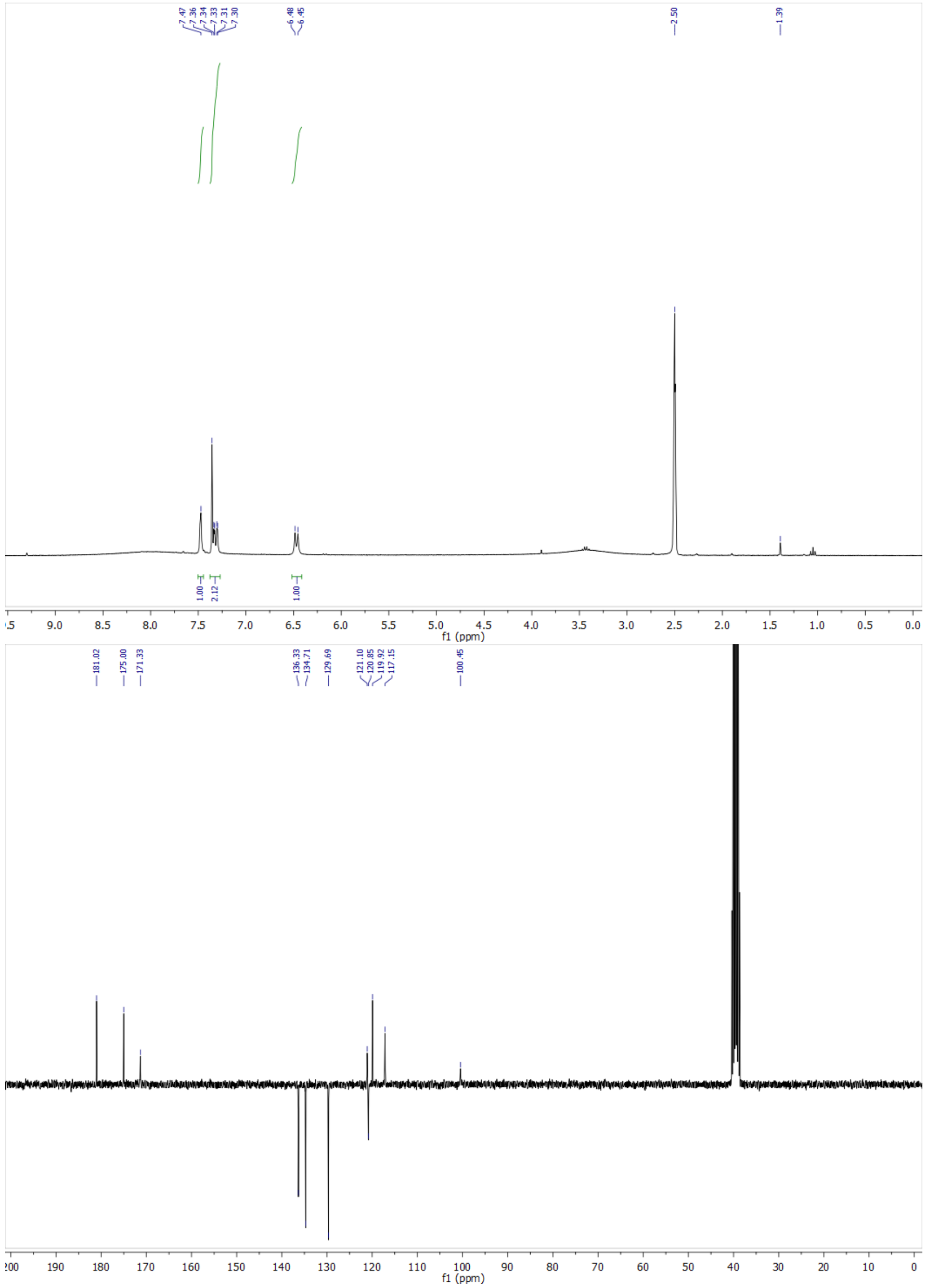

Figure S8: ^{1}H-NMR and ^{13}C-NMR spectra of **HBP3CN**.

3-Chloro-4-hydroxycinnamaldehyde 5a To a stirred solution of 3-chloro-4-(methoxymethoxy)benzaldehyde **4a** (250 mg, 1.2 mmol) and tributyl (1,3-dioxolan-2-ylmethyl) phosphonium bromide (915 mg, 2.4 mmol, 2 eq) in anhydrous tetrahydrofurane (5 mL) was added sodium hydride (60% dispersion in mineral oil) (100 mg, 2.4 mmol, 2 eq) and a catalytic amount of 18-crown-6 (64 mg, 0.24 mml, 0.2 eq). The reaction mixture was stirred at room temperature for the night. After addition of water, the mixture was extracted with ethyl acetate. The combined organic layers were concentrated in vacuo and the resulting residue was dissolved in THF/H_2O (v/v : 5/1). TFA was added (2 mL) and the solution was stirred at room temperature until complete hydrolysis. The solution was extracted with ethyl acetate. The combined organic layers were dried over magnesium sulfate and concentrated. The crude was purified by flash chromatography on silica gel with cyclohexane/ethyl acetate (v/v : 8/2) as eluant. **5a** was obtained as a pale yellow solid (175 mg, 77% yield). **^{1}H NMR (300 MHz, acetone-d$_6$, δ):** 9.67 (d, J = 9Hz, 1H), 7.78 (d, J = 3 Hz, 1H), 7.58 (d, J = 15 Hz, 1H), 7.58 (dd, J = 3 and 9 Hz, 1H), 7.11 (d, J = 9 Hz, 1H), 6.68 (dd, J = 9 and 15 Hz, 1H) ; **^{13}C NMR (75 MHz, DMSO-d$_6$, δ):** 193.9, 155.8, 152.1, 130.4, 129.1, 126.6, 126.4, 120.5, 116.9. **HRMS (ESI):** m/z calc. for $C_9H_6ClO_2$: 181.0136, found: 181.0064 $[M\text{-}H]^-$.

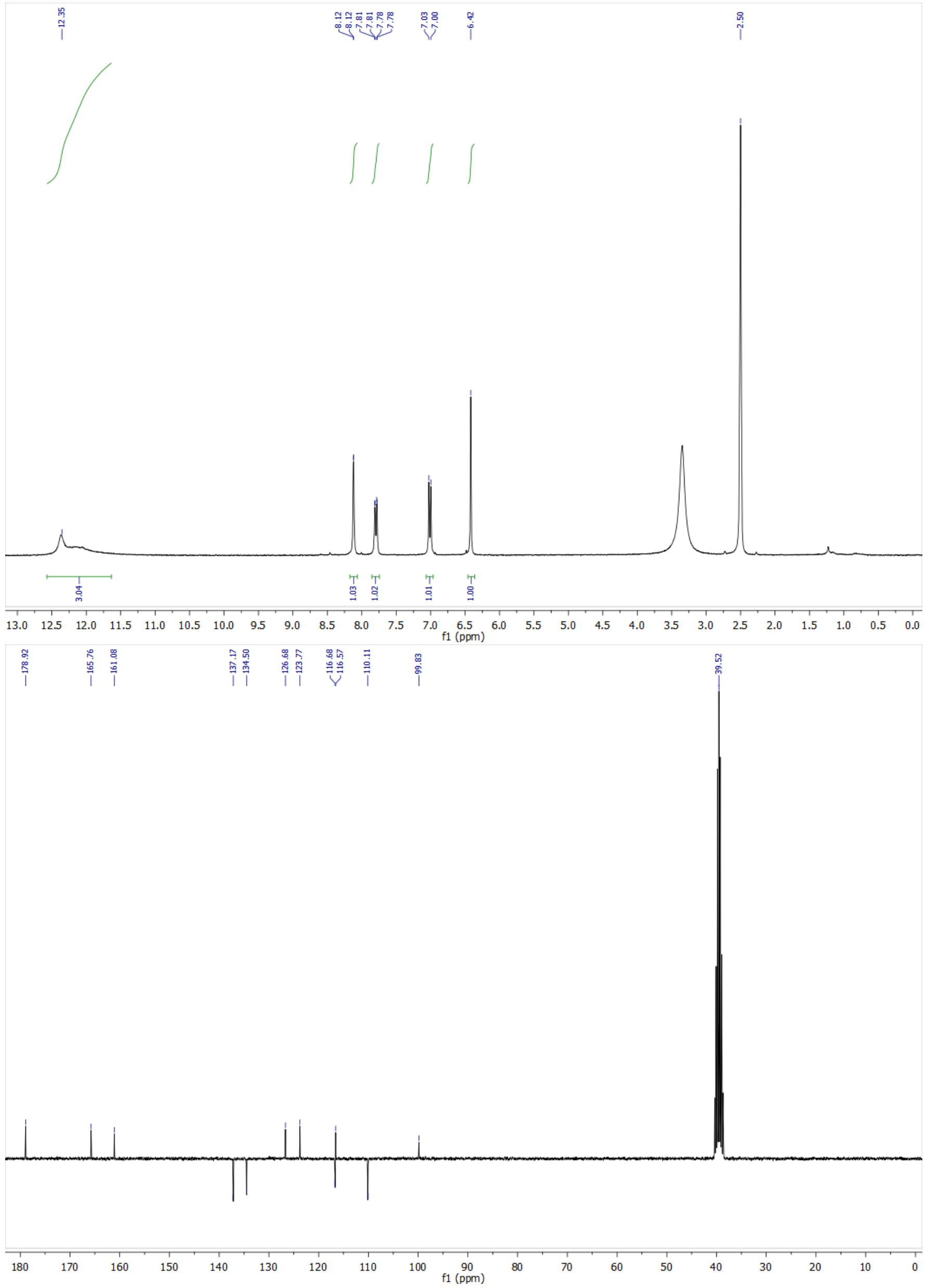

Figure S9: ^{1}H-NMR and ^{13}C-NMR spectra of **HBTH3CN**.

To a stirred solution of rhodanine **2e** (150 mg, 1.1 mmol, 1.5 eq) and 4-hydroxy 3-chlorocinnamaldehyde (135 mg, 0.74 mmol) in ethanol (8 mL) was added 4-dimethylaminopyridine (14 mg, 0.11 mmol, 0.15 eq). The solution was stirred at reflux for 24 h. The mixture was neutralized by adding 1 M HCl (16 mL). After cooling to 4 °C and standing overnight, the resulting precipitate was filtered, washed with a cold solution EtOH/H_2O (v/v : 1/5). After vacuum drying over P_2O_5, **HPAR3Cl** was obtained as a red powder (195 mg, 88% yield). **^{1}H NMR (300 MHz, acetone-d_6, δ):** 7.77 (d, J = 3 Hz, 1H), 7.52 (dd, J = 3 and 9 Hz, 1H), 7.34 (dd, J = 1 and 12 Hz, 1H), 7.23 (bd, J = 15 Hz, 1H), 7.07 (d, J = 9 Hz, 1H), 6.93 (dd, J = 12 and 15 Hz, 1H)) ; **^{13}C NMR (75 MHz, DMSO-d_6, δ):** 195.2, 168.6, 154.8, 143.8, 132.5, 129.3, 128.7, 128.1, 125.7, 121.9, 120.5, 116.8. **HRMS (ESI):** m/z calc. for $C_{12}H_7ClNO_2S_2$: 295.9687, found: 295.9615 $[M\text{-}H]^-$.

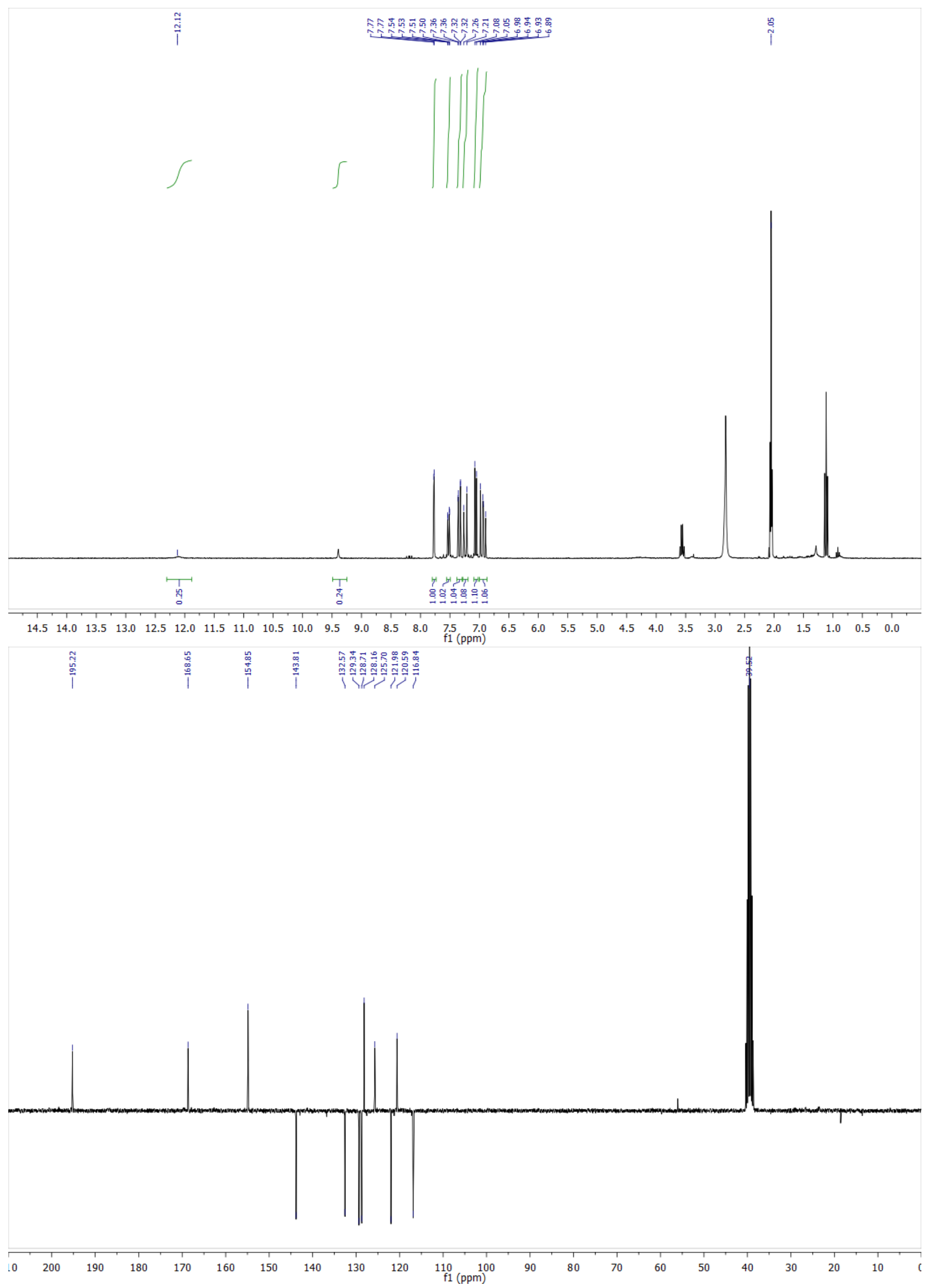

Figure S10: ^{1}H-NMR and ^{13}C-NMR spectra of **HPAR3Cl**.

2.1.2.6 ((Z)-5-((E)-3-(4-hydroxy-3-fluoro-5-methoxyphenyl)allylidene)-2 thioxothiazolidin-4-one 6b [HPAR3F5OM].

3-Fluoro-4-(methoxymethoxy)-5-methoxybenzaldehyde 4b Same as **4a** using 3-fluoro-4-hydroxy-5-methoxybenzaldehyde (544 mg, 3.2 mmol). The desired compound **4b** was obtained quantitatively as a pale yellow viscous oil and used without further purification for the next step. **^{1}H NMR (300 MHz, acetone-d$_6$, δ):** 9.92 (d, 1H, J= 3 Hz), 7.45 (dd, J = 3 Hz, 1H), 7.38 (dd, J = 3 and 12 Hz, 1H), 7.43 (d, J = 9 Hz, 1H), 5.22 (s, 2H), 3.99 (s, 3H), 3.54 (s, 3H).

3-Fluoro-4-hydroxy-5-methoxycinnamaldehyde 5b Same as **5a** using 3-Fluoro-4-(methoxymethoxy)-5-methoxybenz aldehyde **4b** (600 mg, 2.8 mmol), tributyl (1,3-dioxolan-2-ylmethyl) phosphonium bromide (2 g, 5.6 mmol, 2 eq), sodium hydride (60% dispersion in mineral oil) (224 mg, 5.6 mmol, 2 eq) and 18-crown-6 (148 mg, 0.56 mml, 0.2 eq) in dry tetrahydrofurane (15 mL). After purification by flash chromatography on silica gel with cyclohexane/ethyl acetate (v/v : 8/2) as eluant. **5b** was obtained as a pale yellow solid (350 mg, 64% yield). **^{1}H NMR (300 MHz, acetone-d$_6$, δ):** 9.65

(d, J = 9Hz, 1H), 7.56 (d, J = 15 Hz, 1H), 7.58 (d, J = 15 Hz, 1H), 7.58 (dd, J = 3 and 9 Hz, 1H), 7.11 (d, J = 9 Hz, 1H), 6.68 (dd, J = 9 and 15 Hz, 1H). **^{13}C NMR (75 MHz, DMSO-d$_6$, δ):** 194.0, 152.8 (d, JCF = 2 Hz), 151.2 (d, JCF = 237 Hz), 149.6 (d, JCF = 7 Hz), 137.5 (d, JCF = 14 Hz), 127.1, 124.7 (d, JCF = 9 Hz), 109.8 (d, JCF = 19 Hz), 108.2, 56.4. **^{19}F NMR (282 MHz, DMSO-d$_6$, δ):** -137.95 (s, 1F). **HRMS (ESI):** m/z calc. for $C_{10}H_8FO_3$: 195.0537, found: 195.0464 $[M-H]^-$.
Same as **HPAR3Cl** using rhodanine **2e** (180 mg, 1.33 mmol, 1.5 eq), 3-Fluoro-4-hydroxy-5-methoxycinnamaldehyde (175 mg, 0.89 mmol) and 4-dimethylaminopyridine (16 mg, 0.13 mmol, 0.15 eq) in ethanol (8 mL). **HPAR3F5OM** was obtained as a red powder (240 mg, 88% yield). **^{1}H NMR (300 MHz, DMSO-d$_6$, δ):** 13.56 (br, 1H), 9.84 (s, 1H), 7.30 (d, J = 12 Hz, 1H), 7.21 (dd, J = 3Hz and JHF = 12 Hz, 1 H), 7.19 (d, J = 15Hz, 1H), 7.14 bs, 1H), 6.93 (dd, J = 12 and 15 Hz, 1H), 3.87 (s, 3H) ; **^{13}C NMR (75 MHz, DMSO-d$_6$, δ):** 195.1, 168.6, 151.2 (d, JCF = 248 Hz), 149.7 (d, JCF = 5 Hz), 144.5 (d, JCF = 2 Hz), 136.7 (d, JCF =14 Hz), 132.4, 126.4 (d, JCF =9 Hz), 125.7, 122.3, 108.9 (d, JCF = 19 Hz), 107.6, 56.4 ; **^{19}F NMR (282 MHz, DMSO-d$_6$, δ):** -137.97 (s, 1F). **HRMS (ESI):** m/z calc. for $C_{13}H_9FNO_3S_2$: 310.0087, found: 310.0015 $[M-H]^-$.

2.2 Production and purification of fluorescent proteins

2.2.1 Plasmids

The plasmids for bacterial expression of **Dronpa-2**[1] and mammalian expression of **H2B-Dronpa-2**[2] were previously described. The plasmids for mamalian expression of H2B-xFAST (x=Y,[3] p,[4] nir,[5] fr,[6] t,[4] green,[7] i,[8] o,[4] Tsi[9]) have been reported in the original articles.

2.2.2 Production and purification of Dronpa-2

The **Dronpa-2** plasmid with an N-terminal hexahistidine tag was transformed in E. coli TOP10 strain. Cells were grown in Terrific Broth at 37 °C. The expression was induced at 16 °C by addition of IPTG to a final concentration of 1 mM at optical density at 600 nm of 0.6. The cells were collected after 16 h of expression and lysed by sonication in lysis buffer (50 mM PBS with 150 mM NaCl at pH 7.4, 1 mg.ml^{-1} DNAse, 5 mM $MgCl_2$ and 1 mM phenyl-methylsulfonyl fluoride, and a cocktail of protease inhibitors (Roche, ref 04693132001). After lysis, the mixture was incubated on ice for 2 h for DNA digestion. The insoluble material was removed by centrifugation and the supernatant was incubated overnight with Ni-NTA agarose beads (ThermoFisher) at 4 °C in a rotator-mixer. The protein loaded Ni-NTA column was washed with 20 column volumes of N1 buffer (50 mM PBS, 150 mM NaCl, 20 mM imidazole, pH 7.4) and 5 column volumes of N2 buffer (50 mM PBS, 150 mM NaCl, 40 mM imidazole, pH 7.4). The bound protein was eluted with N3 buffer (50 mM PBS, 150 mM NaCl, 0,5 M imidazole, pH 7.4). Imidazole was removed from protein fractions using an PD-10 column (Amersham -Cytiva) and replaced with 50 mM PBS, 150 mM NaCl pH 7.4 buffer.

2.2.3 Production and purification of pFAST

Expression. Plasmids were transformed in Rosetta (DE3) pLysS E. coli (Merck). Cells were grown at 37 °C in a lysogen broth (LB) medium supplemented with 50 μg/mL kanamycin and 34 μg/mL of chloramphenicol to OD_{600nm} 0.6. Expression was induced overnight at 16 °C by adding isopropyl-β-D-1-thiogalactopyranoside (IPTG) to a final concentration of 1 mM. Cells were collected by centrifugation (4300×g for 20 min at 4 °C) and frozen.
Purification. The cell pellet was resuspended in lysis buffer (PBS supplemented with 2.5 mM $MgCl_2$, 1 mM of protease inhibitor phenylmethanesulfonyl fluoride PMSF, and 0.025 mg/mL DNAse, pH 7.4) and sonicated (5 min, 20% of amplitude) on ice. The lysate was incubated for 2 h on ice to allow DNA digestion by DNAse. Cellular fragments were removed by centrifugation (9000×g for 1 h at 4 °C). The supernatant was incubated overnight at 4 °C by gentle agitation with prewashed Ni-NTA agarose beads in PBS buffer complemented with 20 mM of imidazole. Beads were washed with

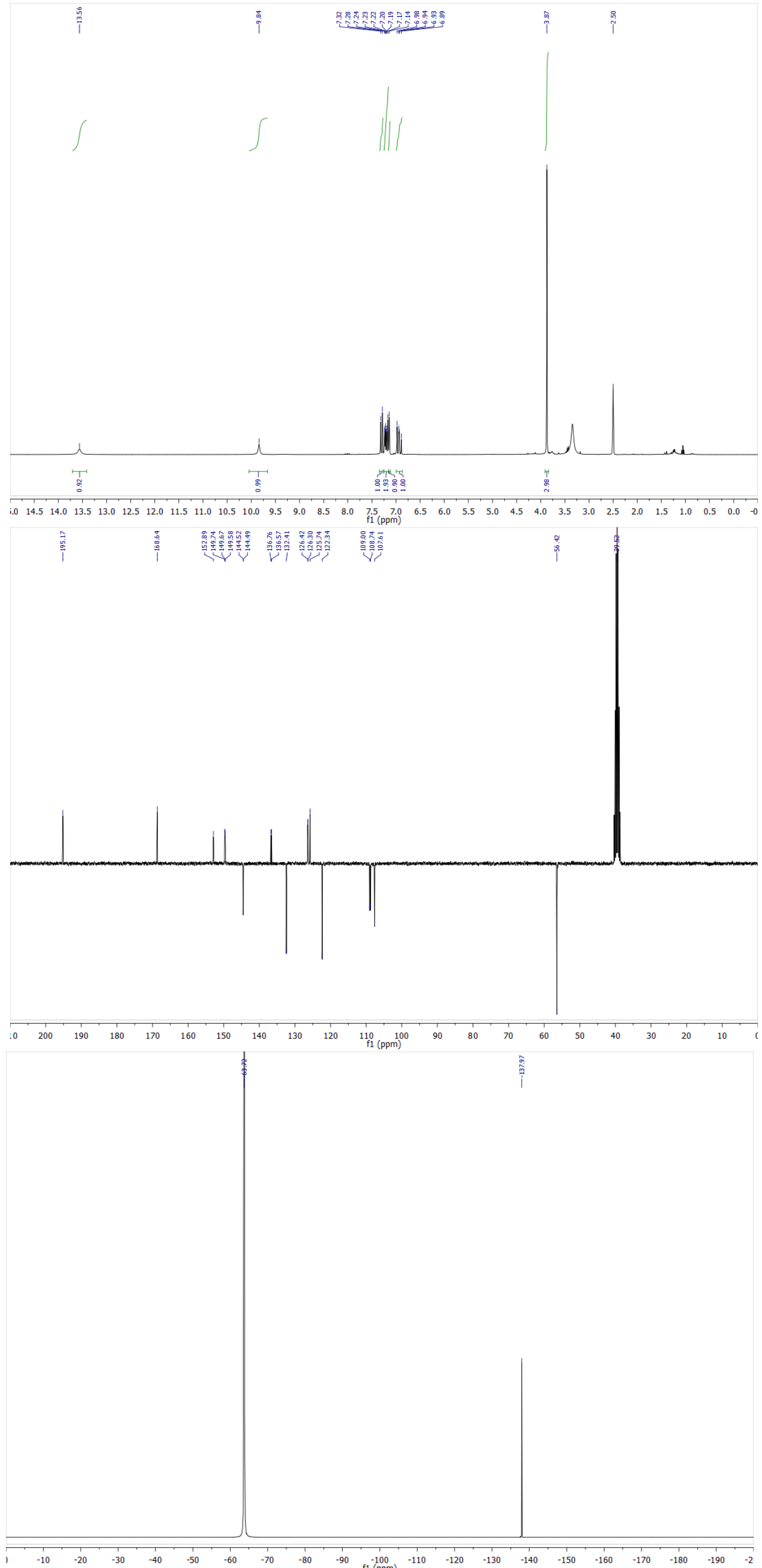

Figure S11: ^{1}H-NMR, ^{13}C-NMR, and ^{19}F-NMR spectra of **HPAR3F5OM**.

10 volumes of PBS complemented with 20mM of imidazole and with 5 volumes of PBS complemented with 40 mM of imidazole. His-tagged proteins were eluted with 5 volumes of PBS complemented with 0.5M of imidazole. The buffer was exchanged to PBS (0.05 M phosphate buffer and 0.150 M NaCl) using PD-10 desalting columns or Midi-Trap G-25 (GE Healthcare). The purity of the proteins was evaluated using SDS–PAGE electrophoresis stained with Coomassie blue.

2.2.4 nirFAST expression in bacteria and purification

Plasmid encoding nirFAST fused to a His-tag was transformed in competent *Escherichia coli* expression strain (BL21(DE3) Competent E. coli / ref C2527H New England Biolabs). Cells were grown at 37 °C in lysogen broth medium supplemented with 50 μg.mL^{-1} kanamycin (and 34 μg.mL^{-1} of chloramphenicol for Rosetta) to OD(600 nm) 0.6. Expression was induced overnight at 16 °C by adding isopropyl β-D-1-thiogalactopyranoside (IPTG) to a final concentration of 1 mM. Cells were collected by centrifugation (4,300 × g for 20 min at 4 °C) and frozen. For purification, the cell pellet was resuspended in lysis buffer (PBS supplemented with 2.5 mM $MgCl_2$, 1 mM of protease inhibitor phenylmethanesulfonyl fluoride and 0.025 mg.mL^{-1} DNase, pH 7.4) and sonicated (5 min, 20 % of amplitude) on ice. The lysate was incubated for 2 h on ice to allow DNA digestion by DNase. Cellular fragments were removed by centrifugation (9000×g for 1 h at 4 °C). The supernatant was incubated overnight at 4 °C by gentle agitation with pre-washed Ni-NTA agarose beads in PBS buffer complemented with 20 mM of imidazole. Beads were washed with ten volumes of PBS complemented with 20 mM of imidazole and with five volumes of PBS complemented with 40 mM of imidazole. His-tagged proteins were eluted with five volumes of PBS complemented with 0.5 M of imidazole. The buffer was exchanged to PBS (0.05 M phosphate buffer and 0.15 M NaCl) using PD-10 desalting columns or Midi-Trap G-25 (GE Healthcare). The purity of the proteins was evaluated using SDS–PAGE electrophoresis stained with Coomassie blue.

2.2.5 Preparation and storage of the solutions

The stock solutions of the free fluorogens were first prepared at 10 mM concentration in spectroscopy grade dimethyl sulfoxide (DMSO) and stored in a glass vial covered with aluminum foil at – 20°C.
The **pFAST** and **nirFAST** stock solutions in 1x pH = 7.4 PBS buffer (140 mM NaCl, 10 mM Na_2HPO_4) were also stored at – 20°C. They were subsequently diluted to the final concentrations in 1x pH = 7.4 PBS buffer just prior to use. The resulting solutions have been covered with aluminum foil and preserved in the dark for the duration of experiment.
All fluorogens and **pFAST** solutions were prepared always in a glass vial to avoid non-specific adsorption.

2.3 Production of labeled mammalian cells

HeLa cells (ATCC CCL2) were cultured in Dulbecco's Modified Eagle Medium (DMEM High glucose, Hyclone, Cytiva) supplemented with phenol red and 10% (vol/vol) fetal calf serum at 37°C in a 5% CO_2 atmosphere. The cells were then seeded (1.4×10^5 cells/mL) in μ-Dish 35 mm Imaging Chamber IBIDI (Biovalley, Clinisciences) coated with poly-L-lysine (ref P4832-Sigma-Merck). Cells were transiently transfected using Genejuice (Merck) according to the manufacturer's protocols for 24–48 h prior to imaging. For imaging, live cells were washed with DPBS (Dulbecco's Phosphate-Buffered Saline), and treated with DMEM media (without serum and phenol red) containing the fluorogens at the indicated concentration. The cells were imaged directly without washing. Fixation of cells was performed using formaldehyde solution at 3.7% for 25 min. Fixed cells were then washed three times with DPBS and treated with DPBS containing the fluorogens at the indicated concentration prior to imaging.

2.4 Instruments

2.4.1 UV/Vis absorption and fluorescence spectrometers

UV/Vis absorption spectra were recorded on a UV/Vis spectrophotometer (Cary 300 UV-Vis, Agilent Technologies, Santa Clara, CA) at 293 K equipped with a Peltier 1×1 thermostatic cell holder (Agilent Technologies). Samples were contained either in 1 cm × 1 cm (3 mL; the cuvette content was stirred) or in 0.3 cm × 0.3 cm (54 µL; the cuvette content was not stirred) quartz cuvettes (Hellma Optics, Jena, Germany). Fluorescence measurements were acquired on a LPS 220 spectrofluorometer (PTI, Monmouth Junction, NJ), equipped with a TLC50 cuvette holder (Quantum Northwest, Liberty Lake, WA) thermoregulated at 293 K.

2.4.2 Light sources

In order to photoconvert the reactants and track the evolution of fluorescent products via absorbance and fluorescence under time constant illumination, we used: a (i) 365, (ii) 405 or (iii) 480 nm Light Emitting Diode (NCSU033B, Nichia Corp., Anan, Japan; LHUV-405, LXZ1-PB01, Lumileds, NL) monochromatically filtered (BP ZET 365-20, Chroma technology, Bellows Falls, VT, BP ET405/20x; Chroma Technology, VT, BP FF01-479/40-25, Semrock, NY) to generate a spatially homogeneous source of illumination.

2.4.3 pH measurements

By assimilating activity and concentration, the proton concentration was directly measured after calibration of the pH meter (PHM210 standard pH meter from MeterLab, Radiometer Analytical) equipped with a combined pH electrode (BNC plug).

2.4.4 Confocal microscopes

The confocal micrographs of the cells were acquired either on a Zeiss LSM 710 Laser Scanning Microscope equipped with continuous laser lines at 405 and 488 nm and a Plan NeoFluar 20×/0.5 objective, or on a Zeiss LSM 980 confocal Laser Scanning Microscope equipped with a 63 ×/1.4 NA oil immersion objective, continuous laser lines delivering light at 405, 488, 561 and 639 nm and equipped with photomultiplier modules. ZEN black software was used to collect the data. The images were analyzed with Fiji (Image J) or ZEN blue.

2.4.5 Home-built microscope for patterned illumination

The images of Figures 4c,d of the Main Text have been recorded with a highly versatile dual–digital micromirror device (DMD) microscope that enables independent and simultaneous spatial and temporal modulation of illumination at two wavelengths, accommodates a wide range of spatial scales through objective interchangeability, and supports diverse excitation and emission wavelength combinations.[10]

2.5 Methods

2.5.1 Measurement of light intensity

The light intensities were measured either through exploiting fluorescent actinometers[11] or by using a power meter.
In this manuscript, we provide the values of the light intensities in $\mathrm{E.m^{-2}.s^{-1}}$ (or mol. of photons.$\mathrm{m^{-2}.s^{-1}}$). This unit is currently used in actinometry. However, it is not often used in other fields such as optical microscopy, in which the researchers prefer to adopt $\mathrm{W.m^{-2}}$. We provide below the conversion between both units.
We consider a monochromatic light of wavelength λ_{exc}. Its values in $\mathrm{E.m^{-2}.s^{-1}}$ and $\mathrm{W.m^{-2}}$ are respectively denoted as $I(\lambda_{\text{exc}}, \mathrm{E.m^{-2}.s^{-1}})$ and $I(\lambda_{\text{exc}}, \mathrm{W.m^{-2}})$. The relation between $I(\lambda_{\text{exc}}, \mathrm{E.m^{-2}.s^{-1}})$ and $I(\lambda_{\text{exc}}, \mathrm{W.m^{-2}})$ is given in Eq.(S43)

$$I(\lambda_{\text{exc}}, \mathrm{W.m^{-2}}) = \frac{hcN_A}{\lambda_{\text{exc}}} \times I(\lambda_{\text{exc}}, \mathrm{E.m^{-2}.s^{-1}}) \approx 0.12 \times \frac{\mathrm{I}(\lambda_{\text{exc}}, \mathrm{E.m^{-2}.s^{-1}})}{\lambda_{\text{exc}}\,(\mathrm{m})} \tag{S43}$$

with the Planck constant $h = 6.63\ 10^{-34}\ \mathrm{m^2.kg.s^{-1}}$, speed of light in a vacuum $c = 3.00\ 10^8\ \mathrm{m.s^{-1}}$, the Avogadro number $N_A = 6.02\ 10^{23}\ \mathrm{mol^{-1}}$, and where λ_{exc} is in m.

2.5.1.1 Cuvette experiments The light intensity delivered from the light sources (i) reported in subsection 2.4.2 has been calibrated by using α-(4-diethylamino)phenyl)-N-phenylnitrone as an actinometer and rhodamine B as a fluorescent reporter. The light intensity delivered from light sources (ii) and (iii) reported in subsection 2.4.2 has been calibrated by using 1 μM Dronpa-2 solution in 1x pH = 7.4 PBS buffer.[11]

2.5.1.2 Confocal microscopes

The light intensity applied at 405 and 488 nm laser in the cell experiments was measured from analyzing the time evolution of the Dronpa-2 fluorescence at the nucleus of Dronpa-2-labeled fixed Hela cells recorded at different light powers over area of 40×40 pixels^2 (frame: 512×512 pixel^2; zoom factor: 1; dwell time: 1.02 μs).[11]

The light intensity applied at 445, 514, 543, 561, and 639 nm with the lasers during the cell experiments in confocal microscopy was measured by analyzing the fluorescence intensity of a 10 μM DDAO solution in aqueous HEPES buffer (pH 7.9; 100 mM NaCl, 5 mM NaOH, 10 mM HEPES), recorded at different light powers over an area of 40×40 pixels^2 (frame: 512 × 512 pixels^2; zoom factor: 4; dwell time: 0.49 μs), following the protocols described in a previous report.[11]

2.5.2 Calibration of the detector sensitivity in confocal microscopy

In order to retrieve the hue of the fluorescence emission, it is first necessary to measure the relative sensitivities of the detection channels, which depend on the detector type, the spectral range and the voltage gain. In this work, we used the GaAsP (for the visible range) and multialkali (for NIR) photomultipliers available on the LSM 980 microscope. For calibration, we imaged live **H2B-pFAST** HeLa cells in DMEM buffer in the presence of 1 μM **HBR3F5OM** under 488 nm illumination. This fluorogen was chosen as a convenient reference since its emission covers the 500-750 nm spectral region of interest.

First, the dependence of sensitivity on wavelength was investigated by imaging the same cell with both detector types and varying the channel spectral range, the width of which was kept at 50 nm, from 500-550 to 700-750 nm. We verified that under these conditions, photobleaching was negligible at the end of the sequence. An ROI in the labeled zone was chosen and its average signal intensity $S(\lambda)$ measured, where λ is the center of the detection spectral range. For each detector type, we define an approximate relative wavelength sensitivity $s_\lambda(\lambda)$:

$$\begin{aligned} s_\lambda(\lambda) &\approx \frac{S(\lambda)}{S(\lambda_{ref})}\frac{b(\lambda_{ref})}{b(\lambda)} \\ \lambda_{ref} &= 500 \text{ nm} \end{aligned} \tag{S44}$$

where $b(\lambda)$ is the brightness of the fluorescent complex. Since $S(\lambda)$ is not continuous, linear interpolation and extrapolation was needed to extend its domain to the entire 500-750 nm region. The relative sensitivities of the multialkali (MA) and GaAsP detectors were defined at the same reference wavelength:

$$\begin{aligned} s_{GaAsP} &= 1 \\ s_{MA} &= \frac{S_{MA}(\lambda)}{S_{GaAsP}(\lambda)} \\ \lambda_{ref} &= 500 \text{ nm} \end{aligned} \tag{S45}$$

Second, the dependence of sensitivity on gain voltage was investigated by imaging the same cell with both detector types and varying the voltage U_g from 650 to 800 V in 50 V increments. The detection range was set to 500-700 nm for both detector types. As before, the ROI average signal $S(U_g)$ was measured. For each detector type, we define a gain sensitivity $s_g(U_g)$:

$$\begin{aligned} s_g(U_g) &= \frac{S(U_g)}{S(U_{g,ref})} \\ U_{g,ref} &= 650\ V \end{aligned} \tag{S46}$$

Finally, we can define the sensitivity $s(\lambda)$ for an arbitrary detection channel with detection range $\lambda_1 - \lambda_2$, gain voltage U_g and detector type det:

$$s(\lambda) = \begin{cases} s_{g,det}(U_g)s_{det}s_{\lambda,det}(\lambda) & \text{if } \lambda \in [\lambda_1, \lambda_2] \\ 0 & \text{otherwise} \end{cases} \tag{S47}$$

2.5.3 Methods for acquisition of the thermokinetic and photophysical parameters of the free fluorogens

2.5.3.1 Measurement of $K_{\mathbf{FH}}$ The present fluorogens contain an ionizable phenol group yielding an acid and a basic state denoted **FH** and **F**$^-$. At chemical equilibrium, one can write Eqs.(S48–S49)

$$FH = \frac{H}{H + K_{\mathbf{FH}}} F_{tot} \tag{S48}$$

$$F^- = \frac{K_{\mathbf{FH}}}{H + K_{\mathbf{FH}}} F_{tot} \tag{S49}$$

where H designates the proton concentration and $K_{\mathbf{FH}}$ is the proton exchange thermodynamic constant associated to **FH**. In the present study, we retrieved $K_{\mathbf{FH}}$ by analyzing the evolutions of the absorbance $A(\lambda)$ of the free fluorogens as a function of pH were analyzed with Eq.(S50)

$$A(\lambda) = (\varepsilon_{F^-} F^- + \varepsilon_{FH} FH)\ell \tag{S50}$$

where ε_{F-}, ε_{FH}, and ℓ respectively designate the molar absorption coefficients of **F**$^-$ and **FH**, and the optical path length, and where F^- and FH are given in Eqs.(S48,S49).

2.5.3.2 Measurement of $k^{\Delta}_{FF'} + k^{\Delta}_{F'F}$ The sum of the thermal rate constants $k^{\Delta}_{FF'} + k^{\Delta}_{F'F}$ associated with thermal relaxation of the fluorogen after photoisomerization was determined by performing a thermal return experiment. A solution of free fluorogen was illuminated with light intensity I until it reached the steady state. Then the illumination was turned off for a period of time Δt, before illuminating the sample again up to its steady state. This sequence was repeated for a range of Δt values. The recovery of fluorescence ΔI^{Δ}_{F} during the dark period as a function of Δt is given in Eq.(S51).

$$\Delta I^{\Delta}_{F}(\Delta t) = I_{F,\infty} + \Delta I_F e^{-(k^{\Delta}_{FF'} + k^{\Delta}_{F'F})\Delta t} \tag{S51}$$

Fitting the experimental data with Eq.(S51) yielded $k^{\Delta}_{FF'} + k^{\Delta}_{F'F}$.

2.5.3.3 Measurement of $\sigma_{FF'} + \sigma_{F'F}$ The effective cross section associated with photoisomerization of the free fluorogen, $\sigma_{FF'} + \sigma_{F'F}$, was determined by fitting the time evolution of fluorescence intensity of a solution of free fluorogen under illumination at constant light intensity I as reported in reference.[12] The dependence of the inverse of the characteristic time τ retrieved at different I values was then linearly fitted with Eq.(S52).

$$\frac{1}{\tau(I)} = k^{\Delta}_{F'F} + k^{\Delta}_{FF'} + I(\sigma_{FF'} + \sigma_{F'F}) \tag{S52}$$

$\sigma_{FF'} + \sigma_{F'F}$ was eventually determined from the slope of the fit.

2.5.4 Methods for acquisition of the thermokinetic and photophysical parameters of the complexes between pFAST or nirFAST and the fluorogens

2.5.4.1 Measurement of the quantum yield of fluorescence emission The overall emission quantum yield after one-photon excitation, Φ, was calculated from Eq. (S5)

$$\Phi_F(\lambda_{exc}) = \Phi_R \times \frac{I_S}{A_S} \times \frac{A_R}{I_R} \times \frac{n_S^2}{n_R^2} \tag{S5}$$

where the subscripts S and R stand for samples and standard reference respectively, A is the absorbance at the excitation wavelength λ_{exc}, I is the integrated area of the emission spectrum, and n is the refractive index of the solvent. The standard reference for the quantum yield measurements was either rhodamine 6G in ethanol or fluorescein-sodium salt in 0.1 M NaOH with Φ_{ref} = 0.95 (rhodamine 6G) and 0.98 (fluorescein-sodium salt). The absorption and emission spectra were recorded at five different concentrations of fluorogen in presence of excess **pFAST** or **nirFAST** and 3 different concentrations of standard fluorophore. To record emission spectra, λ_{exc} was kept fixed for both the fluorogen and standard reference. The slopes were calculated from integrated area vs absorbance plot for the fluorogen and standard reference and used to calculate the fluorescence quantum yield, Φ (λ_{exc}).

2.5.4.2 Measurement of the thermodynamic dissociation constant of the complex between pFAST or nirFAST and the thermodynamically stable state of the fluorogen (K_d) The thermodynamic dissociation constant of the complex between **pFAST** and the thermodynamically stable state of the fluorogen K_d was determined by fitting the dependence of the fluorescence intensity I_F on the total concentration of the fluorogen F_{tot} at constant concentration of the protein P_{tot} by using Eq.(S53), which was derived from Eq.(S1,S10). Since the complex between **pFAST** and the thermodynamically stable state of the fluorogen is photoactive, this dependence has been retrieved from extrapolating to time zero the decay of the fluorescence signal upon constant illumination.

$$I_F = IQ_B \frac{(F_{tot} + P_{tot} + K_d) - \sqrt{(F_{tot} + P_{tot} + K_d)^2 - 4F_{tot}P_{tot}}}{2} \tag{S53}$$

where IQ_B is a constant scaling factor.

2.5.4.3 Measurement of k_{on}, k_{off} The rate constants associated with the formation of the complex between **pFAST** and the thermodynamically stable (Z) stereoisomer of the fluorogen can be measured using a stop-flow experiment by rapidly mixing the fluorogen and protein solutions and following the fluorescence signal over time.
In the investigated series of fluorogens, we only observed the (Z) stereoisomer at chemical equilibrium. Hence, $K_F \approx 0$. In order to eliminate the impact of photoisomerization, this experiment has further been performed at low light intensity and high enough concentrations so that the time evolution of the fluorescence signal at short time is governed by the binding step involving the , which is much faster than the photoisomerization step ($k_{on}F_{tot} + k_{off} \gg k^{\Delta}_{FF'} + k^{\Delta}_{F'F} + I(\sigma_{FF'} + \sigma_{F'F}), k^{\Delta}_{BB'} + k^{\Delta}_{B'B} + I(\sigma_{BB'} + \sigma_{B'B})$). Then, the system obeys the two-state model shown in Figure (S12).

$$\mathbf{F+P} \underset{k_{off}}{\overset{k_{on}}{\rightleftharpoons}} \mathbf{B}$$

Figure S12: Kinetic model relevant under the experimental conditions of our stop-flow binding experiment.

We consider that the two feeding syringes respectively contain **F** and **P** such that their respective initial concentrations in the cuvette after fast mixing are F_{tot} and P_{tot}. The instantaneous concentrations of **F**, **P**, and **B** in the cuvette, F, P, and B, monotonously evolve towards the equilibrium concentrations $F(\infty)$, $P(\infty)$, and $B(\infty)$ which are given by the expressions (S1–S3). The time evolution of the concentrations is driven by the system of differential equations (S54,S55),

$$\dot{P} = -k_{on}FP + k_{off}B \tag{S54}$$
$$\dot{B} = k_{on}FP - k_{off}B \tag{S55}$$

with solution (S56–S59).[13]

$$B(t) = B(\infty)\left\{1 - \frac{e^{-\frac{t}{\tau}}}{1 + k_{on}\tau B(\infty)\left[1 - e^{-\frac{t}{\tau}}\right]}\right\} \tag{S56}$$
$$F(t) = F_{tot} - B(t) \tag{S57}$$
$$P(t) = P_{tot} - B(t) \tag{S58}$$
$$\tau = \frac{1}{k_{on}(F_{tot} + P_{tot}) + k_{off}} \tag{S59}$$

and time evolution of the fluorescence intensity of this system governed by Eq.(S60)

$$I_F(t) = Q_B I B(\infty)\left\{1 - \frac{e^{-\frac{t}{\tau}}}{1 + k_{on}\tau B(\infty)\left[1 - e^{-\frac{t}{\tau}}\right]}\right\} \tag{S60}$$

by neglecting the brightness of the free fluorogen.

with

$$\frac{1}{\tau(F_{tot})} = k_{on}F_{tot} + k_{off} \tag{S61}$$

$$I_{F,\infty} = F_{tot}I(Q_F + \frac{Q_B k_{on} P_{tot}}{k_{on}F_{tot} + k_{off}}) \tag{S62}$$

$$\Delta I_F = IF_{tot}Q_F - I_{F,\infty} \tag{S63}$$

The time evolution of the fluorescence signal at different F_{tot} values is processed to retrieve the characteristic time $\tau(F_{tot})$ with Eq.(S60). k_{on} is then retrieved from the slope of the dependence of $1/\tau$ on F_{tot} with Eq.(S59). Once k_{on} is extracted, k_{off} is computed by using Eq.(S64)

$$k_{off} = K_d k_{on} \tag{S64}$$

2.5.4.4 Measurement of $k^{\Delta}_{BB'} + k^{\Delta}_{B'B}$ The sum of the thermal rate constants $k^{\Delta}_{BB'} + k^{\Delta}_{B'B}$ was determined by performing a thermal return experiment in the presence of protein in excess such that $P_{tot} \gg K'_d \gg K_d$. The solution was illuminated with light intensity I until it reached the steady state. Then the illumination was turned off for a period of time Δt, before illuminating the sample again up to its steady state. This sequence was repeated for a range of Δt values. The fluorescence recovery associated with the thermal return follows a mono-exponential decay given in Eq.(S65).

$$\Delta I^{\Delta}_F(\Delta t) = I_{F,\infty} + \Delta I_F e^{-\Delta t/\tau} \tag{S65}$$

with

$$\frac{1}{\tau} = \langle k_+ \rangle + \langle k_- \rangle \tag{S66}$$

$$\langle k_+ \rangle = \frac{K_d k^{\Delta}_{FF'} + P_{tot} k^{\Delta}_{BB'}}{K_d + P_{tot}} \approx k^{\Delta}_{BB'} \tag{S67}$$

$$\langle k_- \rangle = \frac{K'_d k^{\Delta}_{F'F} + P_{tot} k^{\Delta}_{B'B}}{K'_d + P_{tot}} \approx k^{\Delta}_{B'B} \tag{S68}$$

Fitting the data with Eq.(S65) yields τ and from it, $k^{\Delta}_{BB'} + k^{\Delta}_{B'B}$.
Note that in practice $k^{\Delta}_{BB'} + k^{\Delta}_{B'B}$ was found to be extremely slow ($< 0.0001 s^{-1}$) for all the studied fluorogens.

2.5.4.5 Measurement of $k^{\Delta}_{BB'}$ and $k^{\Delta}_{B'B}$ At large total concentration in protein scaffold such that $P_{tot} \gg K'_d \gg K_d$, a measurement of the B'/B ratio can be achieved by NMR spectroscopy in order to determine $k^{\Delta}_{BB'}$ and $k^{\Delta}_{B'B}$. Indeed

$$F(\infty) \approx 0 \tag{S69}$$

$$F'(\infty) \approx 0 \tag{S70}$$

$$B(\infty) = \frac{F_{tot}}{K^{\Delta}_B + 1} \tag{S71}$$

$$B'(\infty) = \frac{F_{tot}K^{\Delta}_B}{K^{\Delta}_B + 1} \tag{S72}$$

which yields

$$\frac{B'(\infty)}{B(\infty)} = K^{\Delta}_B = \frac{k^{\Delta}_{BB'}}{k^{\Delta}_{B'B}} \tag{S73}$$

Since K^{Δ}_B and $k^{\Delta}_{BB'} + k^{\Delta}_{B'B}$ are known, $k^{\Delta}_{BB'}$ and $k^{\Delta}_{B'B}$ can be calculated as

$$k^{\Delta}_{BB'} = \frac{(k^{\Delta}_{BB'} + k^{\Delta}_{B'B})K^{\Delta}_B}{1 + K^{\Delta}_B} \tag{S74}$$

$$k^{\Delta}_{B'B} = \frac{k^{\Delta}_{BB'} + k^{\Delta}_{B'B}}{1 + K^{\Delta}_B} \tag{S75}$$

Note that $k^{\Delta}_{BB'}$ was found to be negligible for all studied fluorogens.

2.5.4.6 Measurement of $\sigma_{BB'}+\sigma_{B'B}$ $\sigma_{BB'}+\sigma_{B'B}$ was measured by analyzing the time evolution of the fluorescence signal from a solution containing both the protein scaffold and the fluorogen in the presence of an excess of protein so that $P_{tot} \gg K'_d \gg K_d$ and under experimental conditions when fluorogen photoisomerization is rate limiting. Under such conditions,

$$\langle k_+ \rangle = \frac{K_d(k^{\Delta}_{FF'} + I\sigma_{FF'}) + P_{tot}(k^{\Delta}_{BB'} + I\sigma_{BB'})}{K_d + P_{tot}} \approx k^{\Delta}_{BB'} + I\sigma_{BB'} \tag{S76}$$

$$\langle k_- \rangle = \frac{K'_d(k^{\Delta}_{F'F} + I\sigma_{F'F}) + P_{tot}(k^{\Delta}_{B'B} + I\sigma_{B'B})}{K'_d + P_{tot}} \approx k^{\Delta}_{B'B} + I\sigma_{B'B} \tag{S77}$$

the time evolution of the fluorescence intensity follows a mono-exponential decay given in Eq.(S78)

$$I_F(t) = I_{F,\infty} + \Delta I_F e^{-t/\tau} \tag{S78}$$

with

$$\frac{1}{\tau(I)} = k^{\Delta}_{BB'} + k^{\Delta}_{B'B} + I(\sigma_{BB'} + \sigma_{B'B}). \tag{S79}$$

The characteristic time τ was retrieved from fitting the time evolution of the fluorescence intensity with Eq.(S78) for a range of light intensities I. $\sigma_{BB'} + \sigma_{B'B}$ was then extracted from the slope of a linear fit of $1/\tau$ against I.

2.5.4.7 Measurement of $Q'_B/Q_{B'}$, $\sigma_{BB'}$, and $\sigma_{B'B}$ Direct measurement of the concentrations of the proportions of the states **B** and **B'** by NMR spectroscopy under illumination to retrieve $\sigma_{BB'}/\sigma_{B'B}$ as reported above proved to be difficult. Therefore, an indirect approach leveraging knowledge on the photochemical properties of the free fluorogen was used.

This approach involves starting with a mixture of the fluorogen states **F** and **F'** at total concentration F_{tot} with a known F'/F ratio. This ratio can be controlled by applying a calibrated jump of light intensity right before the next step. Typically either no (**a**) or high (**b**) light intensity is used, fixing the F'/F ratio to K^{Δ}_F or Σ_F respectively. Then an excess of protein such that $P_{tot} > F_{tot}$ and $P_{tot} \gg K'_d > K_d$ is rapidly added and mixed. It results in an initial state with the concentrations of **B** and **B'** in the same ratio as the ones of **F** and **F'**.[b]

Upon illumination at light intensity I, the fluorescence signal evolves as per the model in Eq.(S78). From the fit, we extracted the initial fluorescence intensities with expressions given in Eqs. (S80,S81)

$$I_{F,a}(0) = \frac{F_{tot}I(Q_B + Q_{B'}K_F)}{1 + K_F} \tag{S80}$$

$$I_{F,b}(0) = \frac{F_{tot}I(Q_B + Q_{B'}\Sigma_F)}{1 + \Sigma_F} \tag{S81}$$

Then, we could compute the ratio of brightnesses of the complexes $y = Q'_B/Q_{B'}$ by using Eq.(S82)

$$\frac{Q_{B'}}{Q_B} = y = \frac{1 + K_F - x(1 + \Sigma_F)}{xK_F(1 + \Sigma_F) - \Sigma_F(1 + K_F)} \tag{S82}$$

with

$$x = \frac{I_{F,b}(0)}{I_{F,a}(0)} \tag{S83}$$

In both cases (**a**) and (**b**), the fluorescence intensity tends towards $I_F(\infty)$ at steady state given in Eq.(S84), which is governed by the light-driven kinetics.

$$I_F(\infty) = \frac{F_{tot}I(1 + y\Sigma_B)}{1 + \Sigma_B} \tag{S84}$$

[b] The addition must be much faster than the thermal kinetics $k^{\Delta}_{FF'} + k^{\Delta}_{F'F}$ so that the ratio remains unchanged.

Then, Σ_B can be calculated using Eq.(S85).

$$\Sigma_B = \frac{1 - z + K_F(1 - yz)}{z(1 + yK_F) - y(1 + K_F)} \tag{S85}$$

with

$$z = \frac{I_F(\infty)}{I_{F,a}(0)} \tag{S86}$$

Finally, $\sigma_{BB'}$ and $\sigma_{B'B}$ can be determined from $\sigma_{BB'} + \sigma_{B'B}$ and Σ_B using Eqs. (S87,S88).

$$\sigma_{BB'} = \frac{(\sigma_{BB'} + \sigma_{B'B})\Sigma_B}{1 + \Sigma_B} \tag{S87}$$

$$\sigma_{B'B} = \frac{\sigma_{BB'} + \sigma_{B'B}}{1 + \Sigma_B} \tag{S88}$$

2.5.4.8 Measurement of K'_d **F'** is kinetically unstable. Hence, a direct measurement of K'_d by titration such as reported above is impossible. To overcome this limitation, we used an indirect method exploiting the difference of the rate constants associated with the thermal recovery after photoisomerization for the free and bound fluorogen. The kinetics of thermal return of a solution of photoisomerized fluorogen with protein concentrations such as $P_{tot} \gg F_{tot}$ and $P_{tot} \gg K_d$ was measured as in subsection 2.5.3.2 at different total protein concentrations P_{tot}. It was fitted with Eq.(S51) to retrieve τ. K'_d was eventually extracted from fitting the dependence of $1/\tau$ on P_{tot} with Eq.(S89)

$$\frac{1}{\tau(P_{tot})} = k^{\Delta}_{BB'} + \frac{K'_d k^{\Delta}_{F'F} + P_{tot} k^{\Delta}_{B'B}}{K'_d + P_{tot}} \tag{S89}$$

2.5.5 Image segmentation and extraction of single-cell kinetic profiles

2.5.5.1 Primary cell segmentation Each video contained between one and two cells with labelled nuclei. To segment the images, we selected a reference frame consiting in the absolute difference between the fifth and the first frame of the green channel to capture the pixels showing significant dynamics. We retrieved the combined nucleus probability flow map computed with Cellpose (CellposeModel default settings, version 4.0.7). We applied a threshold (30) on that map and selected the largest object in the binary mask as our labeled nucleus, with an additional safeguard for cases where nuclei occupies a larger area than the surrounding image content. For a few cells with high expression level, this threshold included the cytoplasm in the segmentation. Those cells were adressed manually by changing the threshold to 100. A few remaining cell errors were corrected with a more robust segmentation: fluorescence overintensity spots were suppressed using percentile-based masking, and nuclei with low contrast relative to cytoplasmic autofluorescence were enhanced on the average or maximum intensity projections. Cellpose standard model is trained on cells with a mean diameter of 30 pixels, to account for large nucleus size and shape variability, increased downscaling and relaxed flow thresholds were applied.

2.5.5.2 Sub-segmentation of cells into spatial micro-regions We collected between 10 and 15 video for each of the FAST variants. To artificially augment our dataset, we subdivided each individual mask into multiple micro-regions. A regular grid of seed points was placed within the cell boundary (mesh spacing: 2.6 μm - 20/256 pixels), and a watershed transform was applied under the constraint of the cell mask. We removed from the dataset samples with an area below 10 pixels to limit noise-dominated measurements. We obtained a total dataset of 2335 micro-regions for the labeling proteins.

2.5.5.3 Extraction and normalization of fluorescence kinetics For every micro-region, the mean fluorescence intensity was extracted as a function of time for the red and green channels. Each time-series was normalized to its first time point. Normalized traces from the two channels were then concatenated to form a single kinetic trace for each micro-region as displayed in Figure 7 of the Main Text. Each time trace contains 150 datapoints.

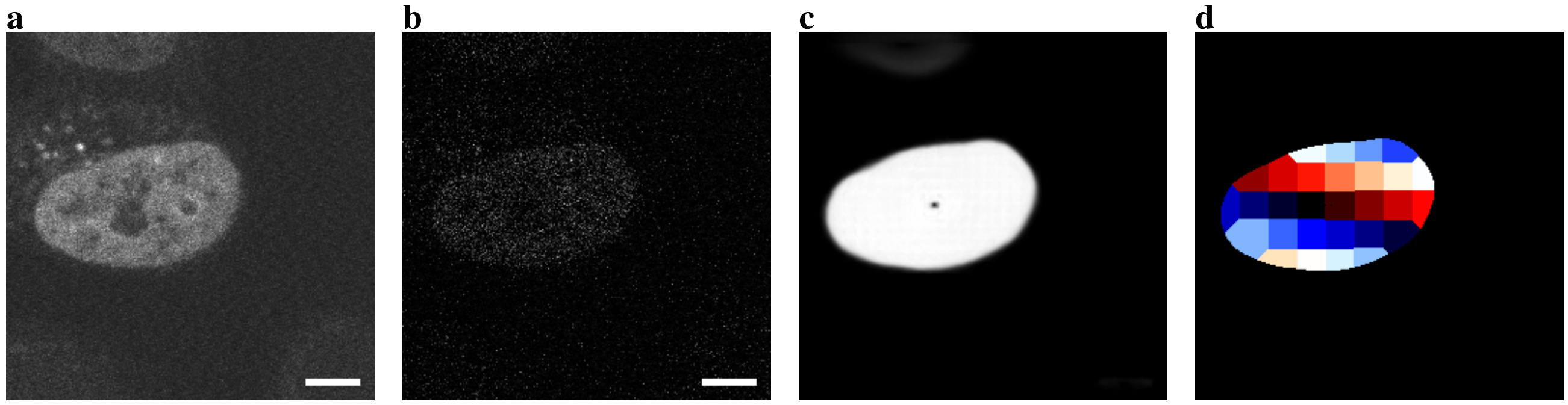

Figure S13: **Segmentation and sub-segmentation workflow.** **a**: Mean of fluorescence frames over the movie; **b**: Reference image (absolute difference between frame 5 and frame 1) used for segmentation; **c**: Cellpose probability map; **d**: Final micro-region partition used for data extraction. Scale bar: 5 μm.

2.5.5.4 Train–test partitioning based on whole cells To prevent information leakage, the train-test split was performed at the level of whole cells and not individual micro-regions. This ensures that the classifier is evaluated on fluorescence trajectories from entirely unseen cells rather than on neighbouring regions of the same object. We selected 3 cells per protein for the test set and the remaining cells were used for the training. All training procedures were performed with fixed random seeds to enforce reproducibility.

2.5.5.5 Cross-validation and model selection We tested several classification models (Linear Discriminant Analysis, Gaussian Naive Bayes and Random Forest) with cross-validation (15-folds). The training set was split into training and validation subsets at the cell level, with 3 cells kept for validation. Since the time traces are relatively long (150 points) compared to the dataset size (1850 samples), we applied a dimensionality reduction to the sub-training set using Principal Component Analysis (PCA). The number of components was treated as a hyperparameter and optimized during cross-validation. The best performing model (Linear Discriminant Analysis) and number of PCA components (8) were selected based on average and standard deviation of cross-validated balanced accuracy.

2.5.5.6 Final model training and evaluation PCA (8 components) was fitted on the training set (all training cells and their micro-regions) and the transform was applied to the test set (3 cells per protein). The LDA classifier was trained on the full training dataset. Model performance was assessed on the unseen test set using balanced accuracy and confusion matrices. Micro-region wise, we obtain an accuracy score of 98% with 12 missclassifications out of the 485 micro-region samples. Further, with a voting strategy per cell, we can reach 100% accuracy to correctly classify the 18 test cells.

3 Investigation of the free fluorogens

3.1 Absorption and emission spectra of the free fluorogens

Figure S14a–f displays the absorption and emission spectra of the free fluorogens in their thermodynamically stable (*Z*) configuration. Table S1 sums up the results.

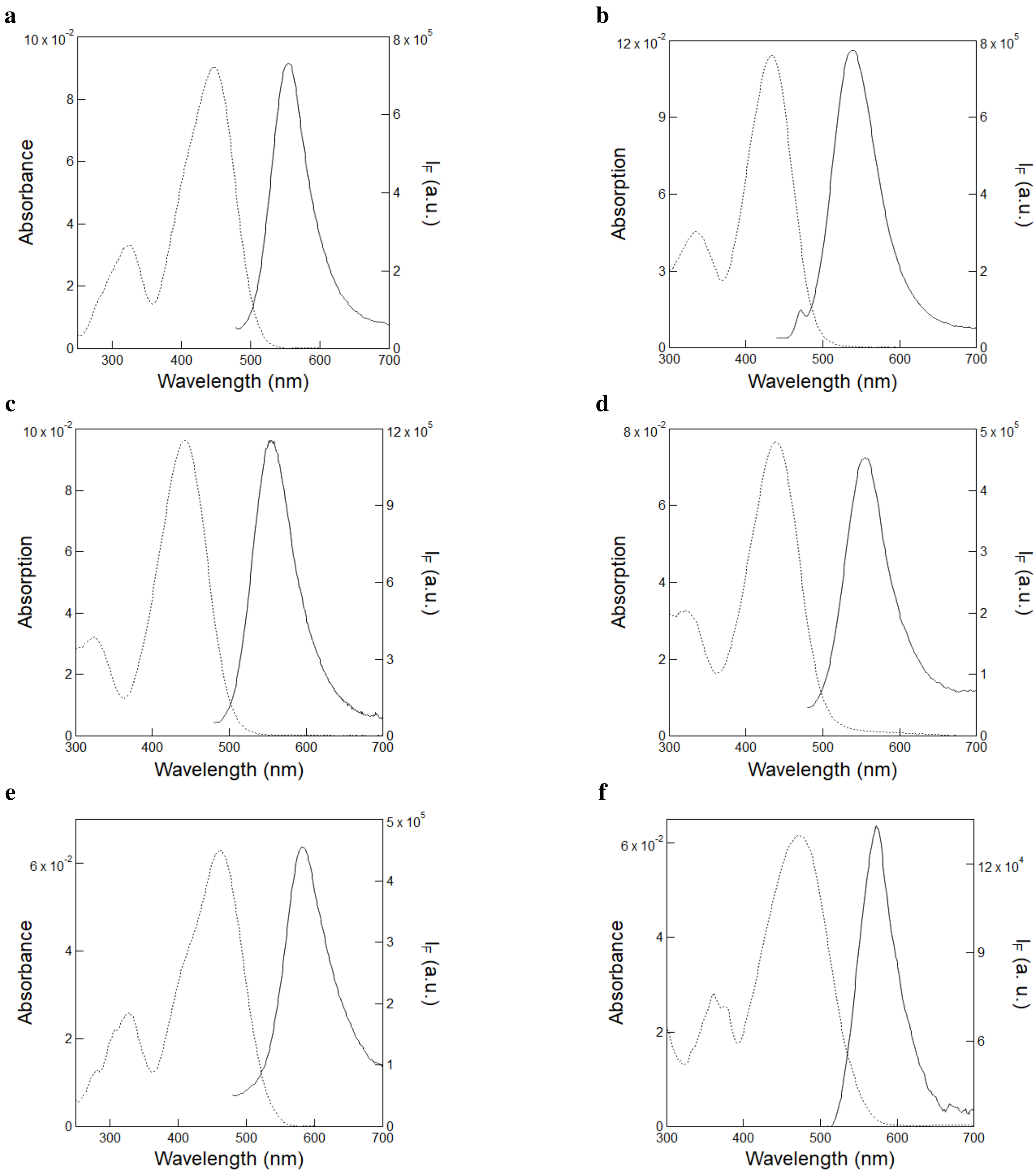

Figure S14: *UV-Vis absorption and emission spectra of the free fluorogens*. The absorption (dotted line) and emission (solid line; λ_{exc} = 405 nm) spectra of free fluorogens **HBR3Cl (a)**, **HBR3CN (b)**, **HBR3Cl5F (c)**, **HBR35DF5 (d)**, **HBR3F5OM (e)** and **HBIR3Cl (f)**. The spectra were recorded using 10 μM fluorogen solution in 1$\times$ pH = 7.4 PBS buffer contained in 54 μL quartz cuvette (3 mm optical pathlength). $T = 293$ K.

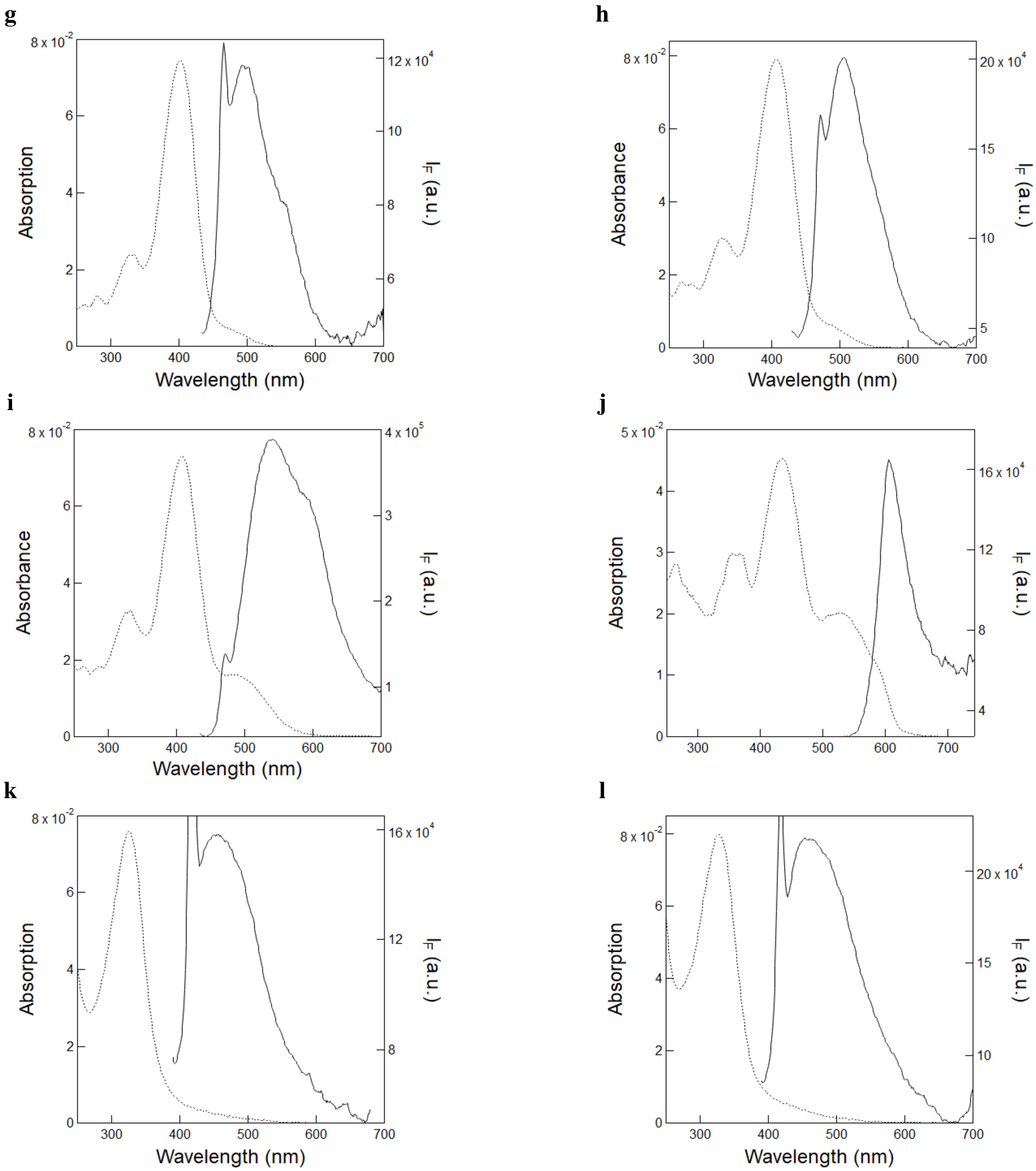

Figure S14: *UV-Vis absorption and emission spectra of the free fluorogens*. The absorption (dotted line) and emission (solid line; λ_{exc} = 365 nm (**HBO3M** and **HBO35DM**); λ_{exc} = 405 nm (**HBR3M**, **HBR25DM** and **HBR35DOM**); λ_{exc} = 480 nm (**HBIR 35DOM**)) spectra of free fluorogens **HBR3M (g)**, **HBR25DM (h)**, **HBR35DOM (i)**, **HBIR35DOM (j)**, **HBO3M (k)** and **HBO35DM (l)**. The spectra were recorded using 10 μM **HBR3M**, **HBR25DM**, **HBR35DOM**, **HBIR35DOM**, 20 μM **HBO3M** and 50 μM **HBO35DM** solution in 1$\times$ pH = 7.4 PBS buffer contained in 54 μL quartz cuvette (3 mm optical pathlength). $T = 293$ K.

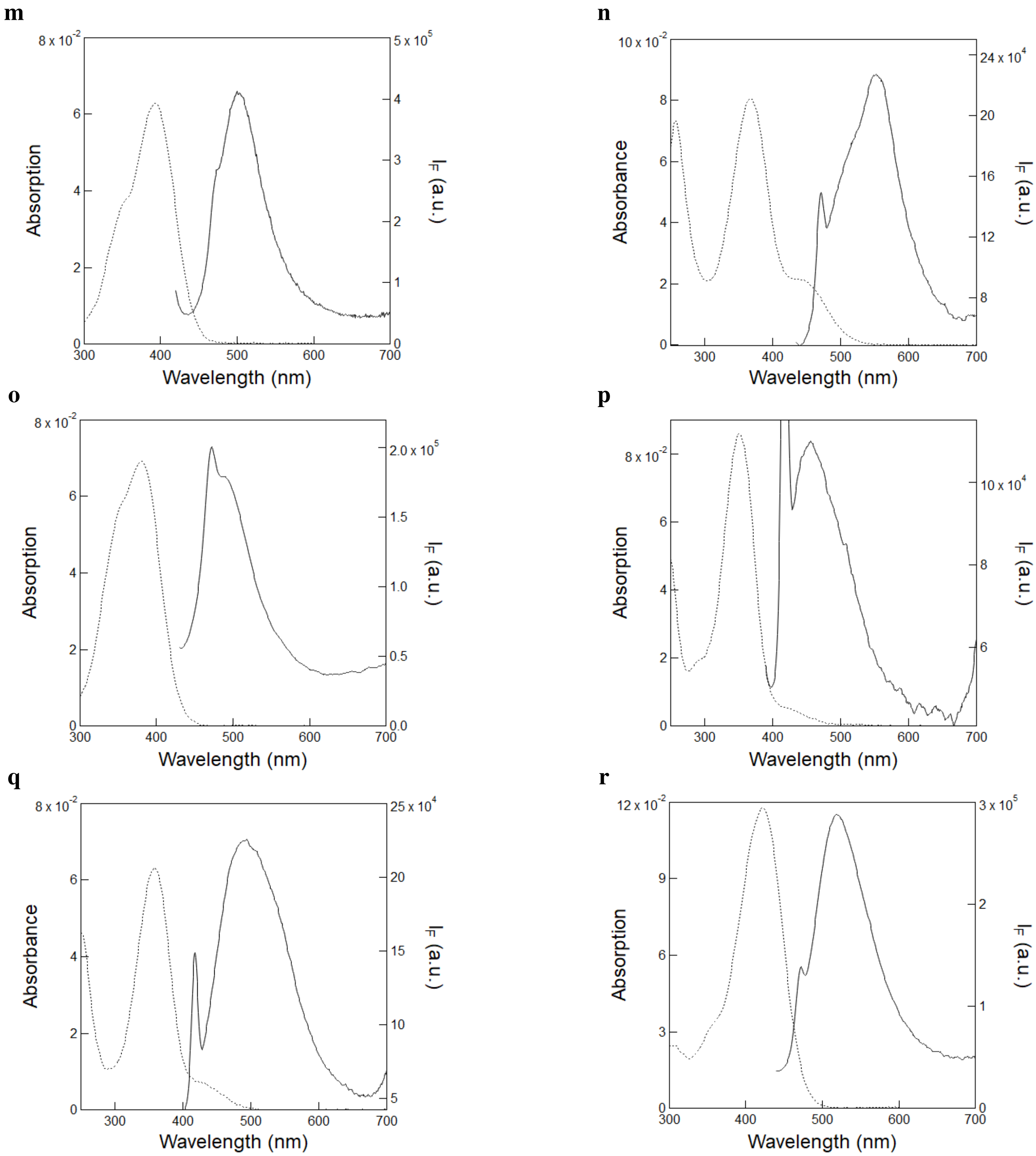

Figure S14: *UV-Vis absorption and emission spectra of the free fluorogens*. The absorption (dotted line) and emission (solid line; λ_{exc} = 365 nm (**HBP35DOM**, **HBT35DM** and **HBT35DOM**); λ_{exc} = 405 nm (**HBP3CN**, **HBT3CN** and **HBTH3CN**)) spectra of free fluorogens **HBP3CN (m)**, **HBP35DOM (n)**, **HBT3CN (o)**, **HBT35DM (p)**, **HBT35DOM (q)** and **HBTH3CN (r)**. The spectra were recorded using 10 μM fluorogen solution in $1\times$ pH = 7.4 PBS buffer contained in 54 μL quartz cuvette (3 mm optical pathlength). $T = 293$ K.

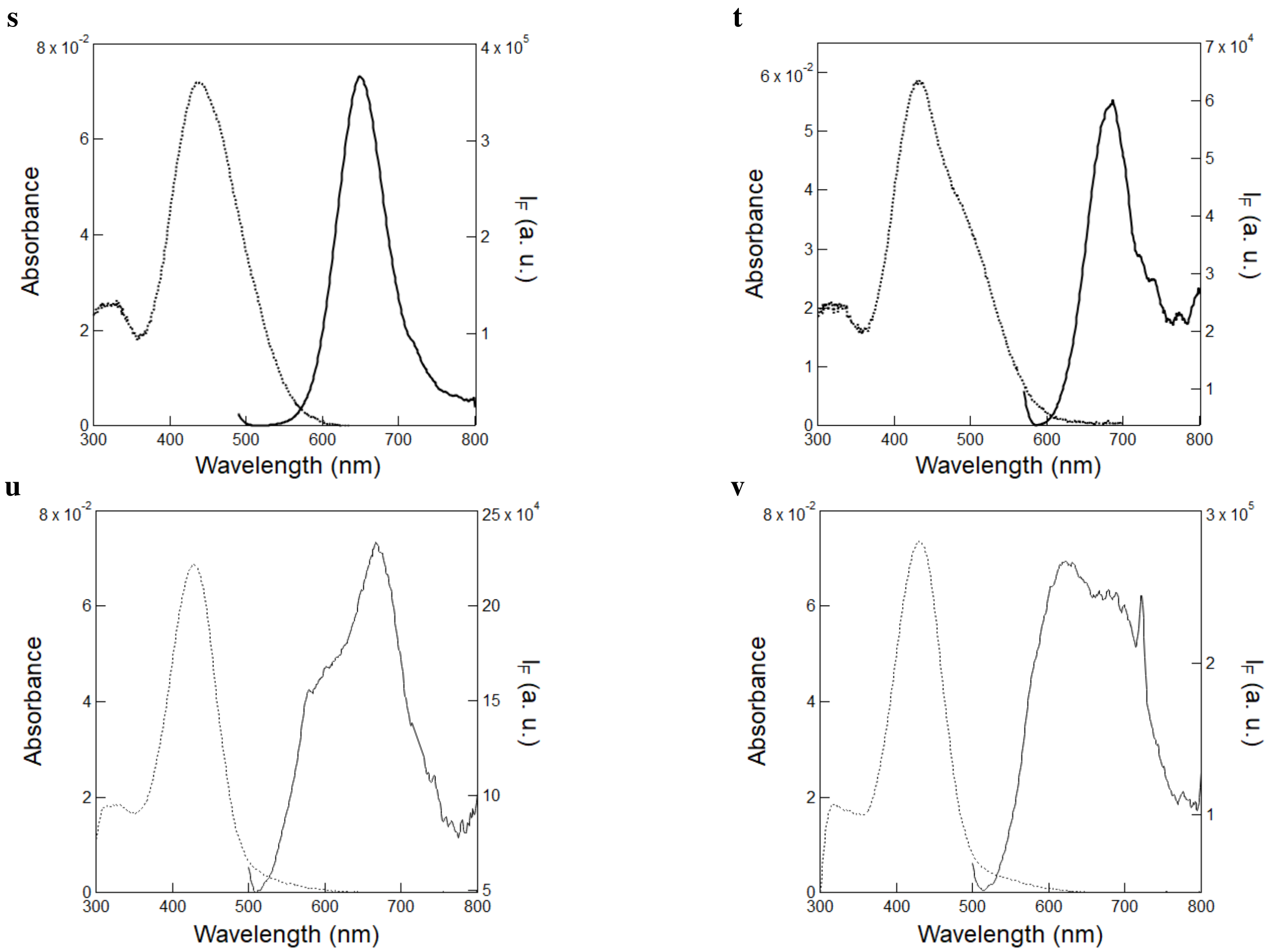

Figure S14: *UV-Vis absorption and emission spectra of the free fluorogens*. The absorption (dotted line) and emission (solid line; λ_{exc} = 480 nm) spectra of free fluorogens **HPAR3Cl (s)**, **HPAR3F5OM (t)**, **HPAR3OM (u)** and **HPAR35DOM (v)**. The spectra were recorded using 10 μM fluorogen solution in 1$\times$ pH = 7.4 PBS buffer contained in 54 μL quartz cuvette (3 mm optical pathlength). $T = 293$ K.

3.2 Measurement of the effective photoisomerization cross section of the free fluorogens

To measure the effective cross section of photoisomerization of the free fluorogens, 54 μL of 10 or 15 μM fluorogen solution in 1$\times$ pH = 7.4 PBS buffer were subjected to a sequence of light pulses of increasing constant intensity I from 365, 405, or 480 nm LEDs separated by long enough periods of darkness to allow for thermal return after photoisomerization to occur. Each time evolution of the fluorescence signal recorded at its maximal emission wavelength was then satisfactorily fitted with a mono-exponential fitting function to retrieve the characteristic time of photoisomerization τ.[12] The effective photoisomerization cross section of the free fluorogen was eventually extracted from the satisfactory linear fit of the inverse of τ vs light intensity I using Eq. (S52) at 365, 405, or 480 nm.

3.2.1 Measurements at 365 nm

Figure S15a–h displays the measurement of the effective photoisomerization cross section for **HBO3M** (Figure S15a,b), **HBO35DM** (Figure S15c,d), **HBT35DM** (Figure S15e,f), and **HBT35DOM** (Figure S15g,h) at 365 nm. From the linear fit of the inverse of τ vs light intensity I, we retrieved 172 $\pm$ 6, 287 $\pm$ 17, 675 $\pm$ 18, and 605 $\pm$ 34 m^2.mol^{-1} for the effective cross section of photoisomerization of **HBO3M**, **HBO35DM**, **HBT35DM**, and **HBT35DOM** at 365 nm.

Table S1: *Photophysical properties of the free fluorogens in their thermodynamically stable (Z) configuration.* Solvent: 1× pH = 7.4 PBS buffer. $T = 293$ K.

Fluorogens	$\lambda_{abs}^{max\dagger}$ (nm)	$\lambda_{em}^{max\ddagger}$ (nm)	ϵ^* ($mM^{-1}cm^{-1}$)
**HBO3M**	325	452	11
**HBT35DM**	350	454	29
**HBO35DM**	327	455	5
**HBT3CN**	380	483	23
**HBT35DOM**	358	491	21
**HBR3M**	402	500	25
**HBP3CN**	393	502	21
**HBR25DM**	407	505	26
**HBTH3CN**	422	519	39
**HBR3CN**	433	538	38
**HBR35DOM**	408	540	24
**HBP35DOM**	367	552	13
**HBR3Cl5F**	442	554	32
**HBR35DF**	439	555	34
**HBR3Cl**	446	555	30
**HBIR3Cl**	472	573	20
**HBR3F5OM**	462	582	21
**HBIR35DOM**	435	605	15
**HPAR35DOM**	430	620	25
**HPAR3Cl**	437	650	24
**HPAR3OM**	428	666	23
**HPAR3F5OM**	434	685	20

† λ_{abs}^{max} wavelength of maximum absorption.
‡ λ_{em}^{max} wavelength of maximum emission.
* ϵ molar absorption coefficient at λ_{abs}^{max}.

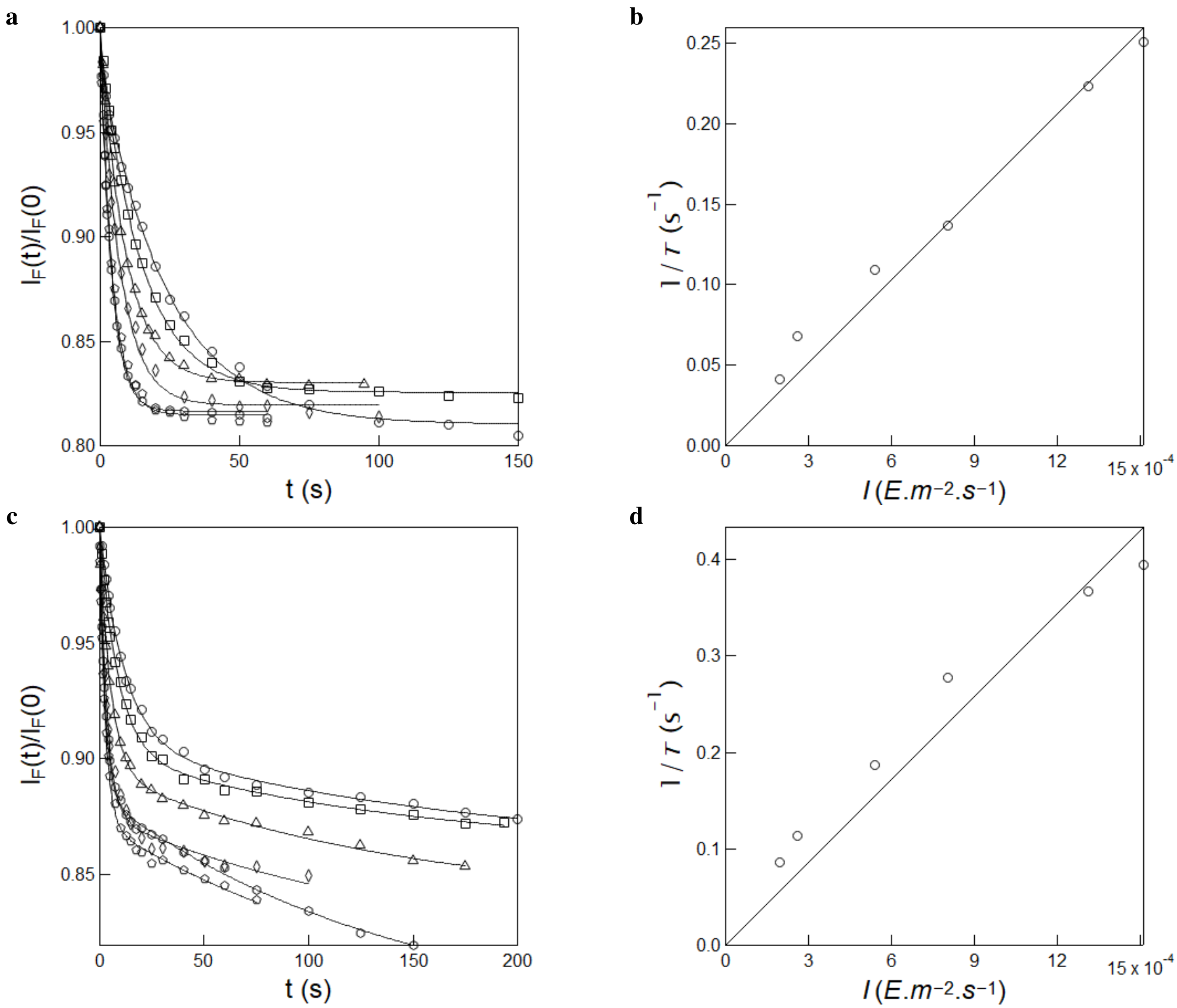

Figure S15: *Measurement of the effective cross section of photoisomerization of* ***HBO3M*** *and* ***HBO35DM*** *under 365 nm illumination.* **a,c**: Time evolution of the normalized fluorescence emission at 500 nm from 20 μM **HBO3M (a)** and 50 μM **HBO35DM (c)** in 1$\times$ pH = 7.4 PBS buffer in a 54 μL cuvette (3 mm optical pathlength) upon irradiation at 365 nm at various constant light intensities (in 10^{-4} E.m^{-2}.s^{-1}): 2.0 (circles), 2.6 (squares), 5.4 (triangles), 8.0 (diamonds), 13.1 (pentagons) and 15.1 (hexagons). Markers: experimental data; solid lines: Monoexponential fit with linear drift delivering τ (s): 24.1, 14.7, 9.1, 7.3, 4.5, and 3.9 (for **HBO3M**) and 11.6, 8.8, 5.3, 3.6, 2.7, and 2.5 (for **HBO35DM**); **b,d**: Extraction of the effective cross section of photoisomerization for **HBO3M (b)** and **HBO35DM (d)** from the τ values retrieved in **a,c**. Markers: experimental data; solid line: linear fit with Eq.(S52). $T = 293$ K.

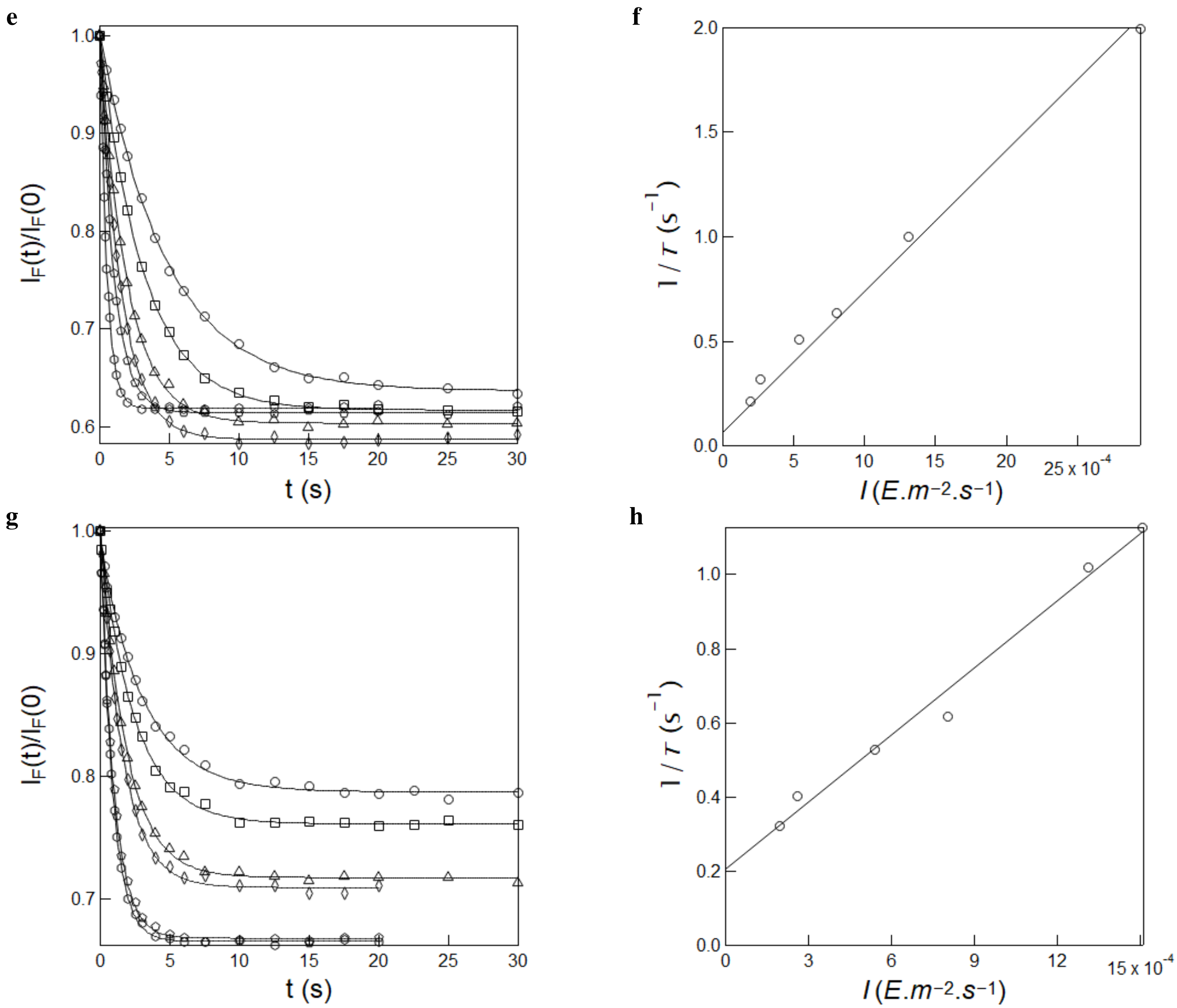

Figure S15: *Measurement of the effective cross section of photoisomerization of* ***HBT35DM*** *and* ***HBT35DOM*** *under 365 nm illumination.* **e,g**: Time evolution of the normalized fluorescence emission at 500 nm from 10 μM **HBT35DM (e)** and **HBT35DOM (g)** in 1× pH = 7.4 PBS buffer in a 54 μL cuvette (3 mm optical pathlength) upon irradiation at 365 nm at various constant light intensities (in 10^{-4} $E.m^{-2}.s^{-1}$): 2.0 (circles), 2.6 (squares), 5.4 (triangles), 8.0 (diamonds), 13.1 (pentagons) and 29.5 (hexagons) with **HBT35DM** and 2.0 (circles), 2.6 (squares), 5.4 (triangles), 8.0 (diamonds), 13.1 (pentagons) and 15.1 (hexagons) with **HBT35DOM**. Markers: experimental data; solid lines: Monoexponential fit delivering τ (s): 4.8, 3.2, 2.0, 1.6, 1.0, and 0.5 (for **HBT35DM**) and 3.1, 2.5, 1.9, 1.6, 1.0, and 0.9 (for **HBT35DOM**); **f,h**: Extraction of the effective cross section of photoisomerization for **HBT35DM (f)** and **HBT35DOM (h)** from the τ values retrieved in **e,g**. Markers: experimental data; solid line: linear fit with Eq.(S52). $T = 293$ K.

3.2.2 Measurements at 405 nm

Figure S16a–n displays the measurement of the effective photoisomerization cross section for **HBR3Cl** (Figure S16a,b), **HBR3CN** (Figure S16c,d), **HBR3Cl5F** (Figure S16e,f), **HBR35DF** (Figure S16g,h), **HBT3CN** (Figure S16i,j), **HBP3CN** (Figure S16k,l) and **HBTH3CN** (Figure S16m,n) at 405 nm. From the linear fit of the inverse of τ vs light intensity I, we retrieved 1472 ± 18, 1691 ± 11, 728 ± 11, 962 ± 13. 1090 ± 40, 1502 ± 57 and 1435 ± 35 $m^2.mol^{-1}$ for the effective cross section of photoisomerization of **HBR3Cl**, **HBR3CN**, **HBR3Cl5F**, **HBR35DF**, **HBT3CN**, **HBP3CN** and **HBTH3CN** at 405 nm.

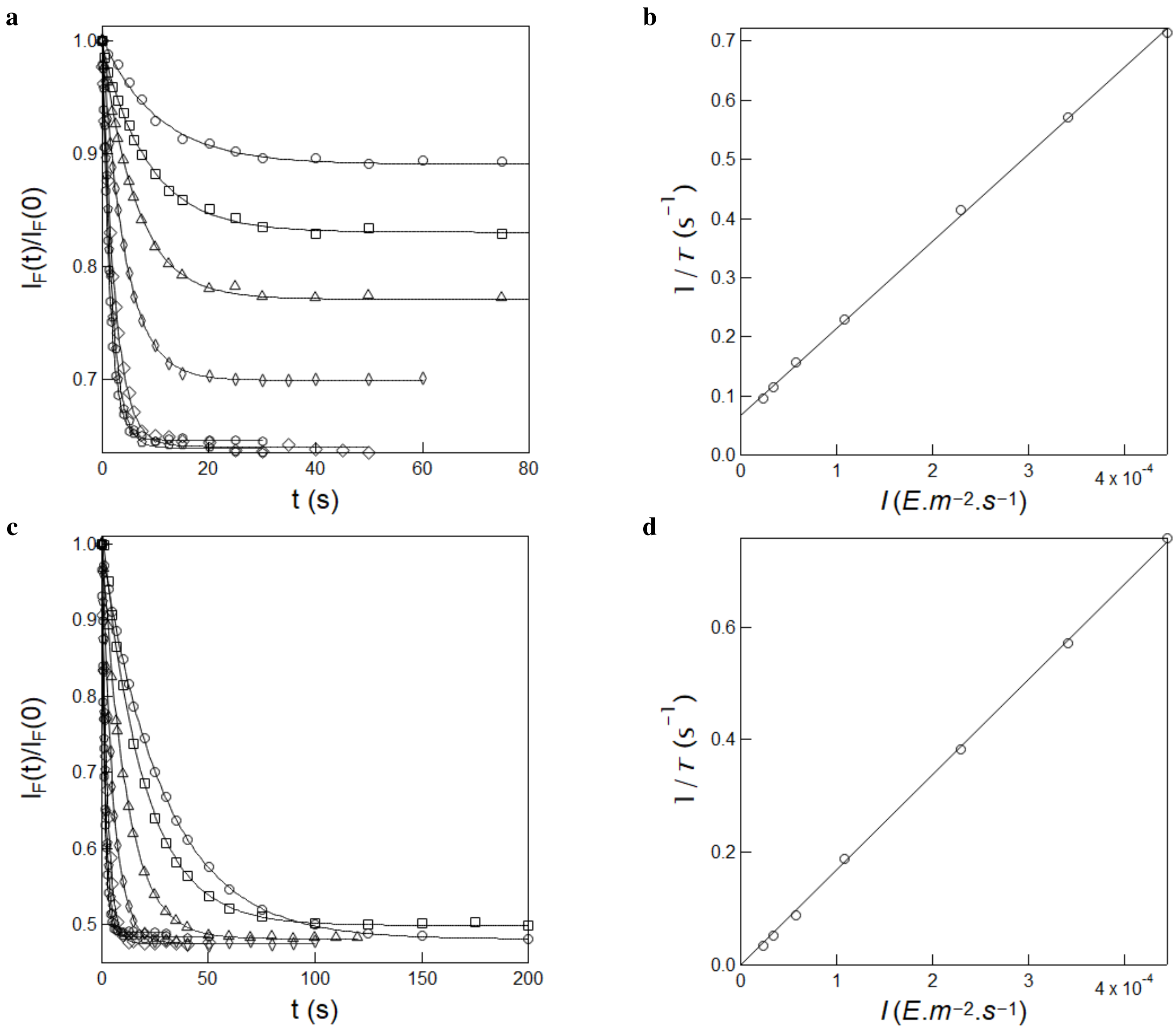

Figure S16: *Measurement of the effective cross section of photoisomerization of **HBR3Cl** and **HBR3CN*** under 405 nm illumination. **a,c**: Time evolution of the normalized fluorescence emission at 550 nm from 10 μM **HBR3Cl (a)** and **HBR3CN (c)** in 1$\times$ pH = 7.4 PBS buffer in a 54 μL cuvette (3 mm optical pathlength) upon irradiation at 405 nm at various constant light intensities (in 10^{-5} E.m^{-2}.s^{-1}): 2.4 (circles), 3.5 (squares), 5.7 (triangles), 10.7 (diamonds), 22.9 (discs), 34.1 (pentagons) and 44.4 (hexagons). Markers: experimental data; solid lines: Monoexponential fit delivering τ (s): 10.5, 8.7, 6.4, 4.3, 2.4, 1.7, and 1.4 (for **HBR3Cl**) and 29.5, 19.5, 11.3, 5.3, 2.6, 1.7, and 1.3 (for **HBR3CN**); **b,d**: Extraction of the effective cross section of photoisomerization for **HBR3Cl (b)** and **HBR3CN (d)** from the τ values retrieved in **a,c**. Markers: experimental data; solid line: linear fit with Eq.(S52). $T = 293$ K.

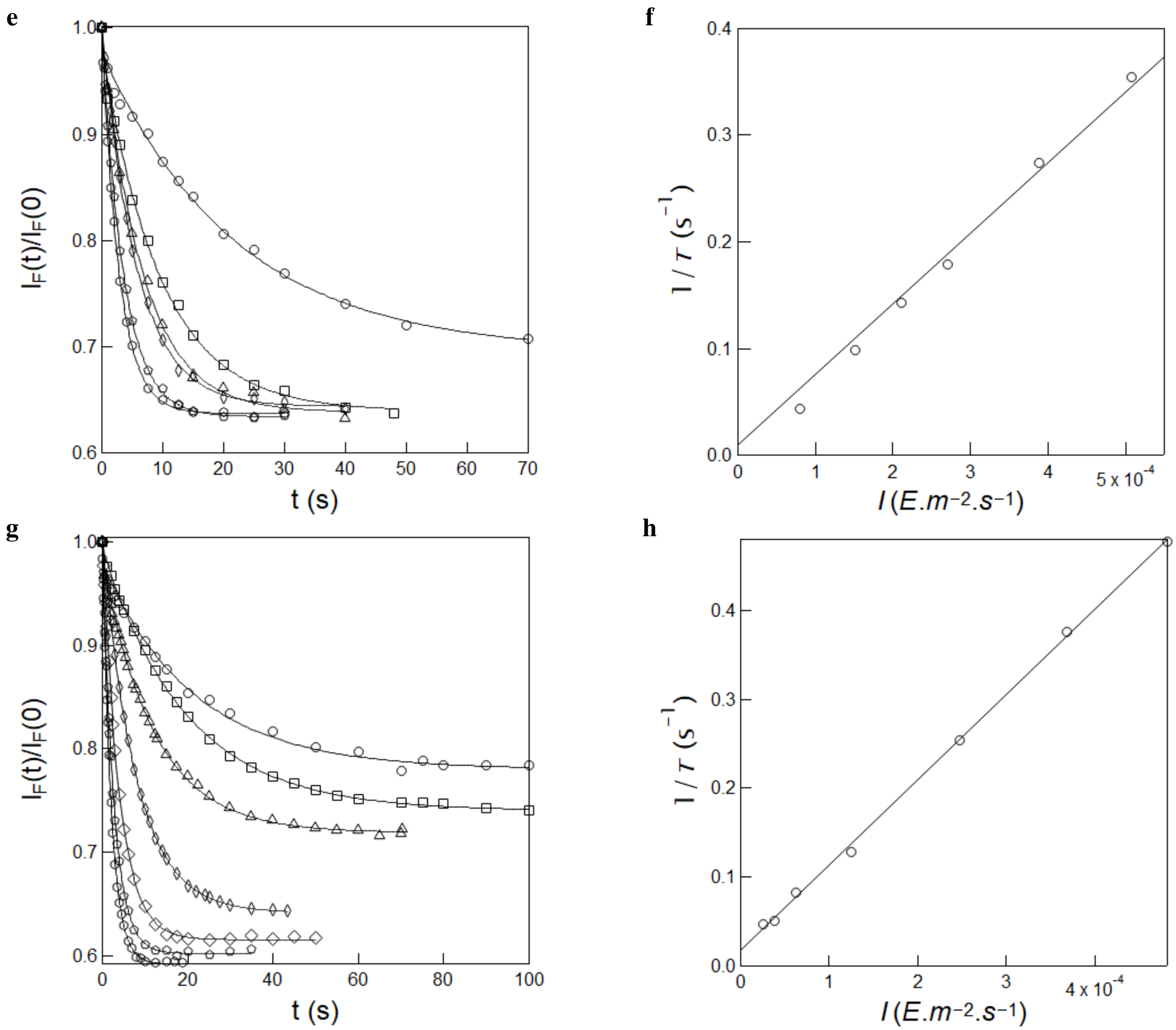

Figure S16: *Measurement of the effective cross section of photoisomerization of **HBR3Cl5F** and **HBR35DF** under 405 nm illumination.* **e,g**: Time evolution of the normalized fluorescence emission at 550 nm from 10 μM **HBR3Cl5F (e)** and **HBR35DF (g)** in 1× pH = 7.4 PBS buffer in a 54 μL cuvette (3 mm optical pathlength) upon irradiation at 405 nm at various constant light intensities (in 10^{-5} E.m^{-2}.s^{-1}): 7.9 (circles), 15.1 (squares), 21.0 (triangles), 27.0 (diamonds), 38.9 (pentagons) and 50.8 (hexagons) with **HBR3Cl5F**, and 2.6 (circles), 3.8 (squares), 6.2 (triangles), 12.5 (diamonds), 24.8 (disks), 36.8 (pentagons) and 48.2 (hexagons) with **HBR35DF**. Markers: experimental data; solid lines: Monoexponential fit delivering τ (s): 22.0, 9.6, 6.7, 5.7, 3.6, and 2.8 (for **HBR3Cl5F**) and 21.5, 19.7, 12.2, 7.8, 3.9, 2.6 and 2.1 (for **HBR35DF**); **f,h**: Extraction of the effective cross section of photoisomerization for **HBR3Cl5F (f)** and **HBR35DF (h)** from the τ values retrieved in **e,g**. Markers: experimental data; solid line: linear fit with Eq.(S52). $T = 293$ K.

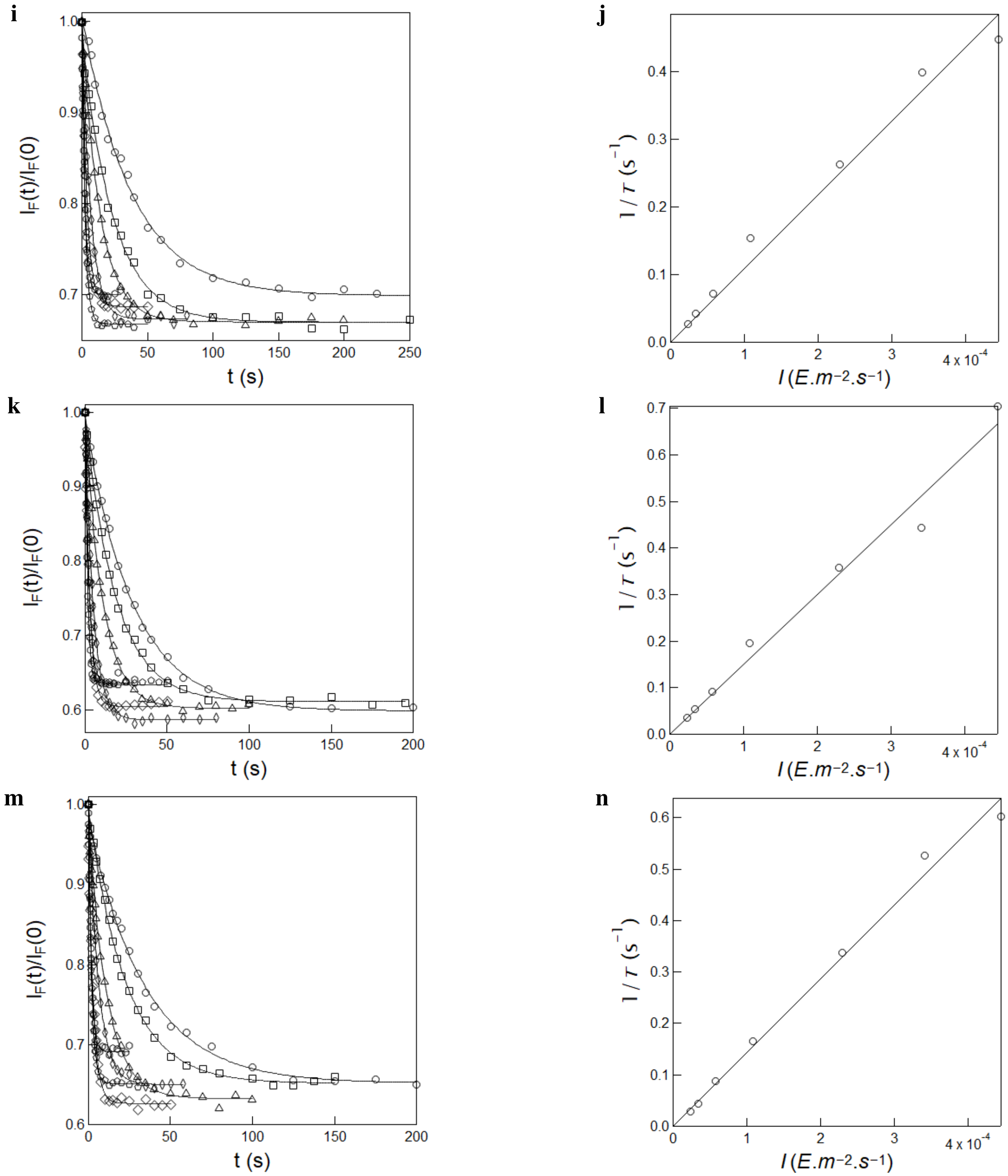

Figure S16: *Measurement of the effective cross section of photoisomerization of* ***HBT3CN****,* ***HBP3CN*** *and* ***HBTH3CN*** *under 405 nm illumination.* **i,k,m**: Time evolution of the normalized fluorescence emission at 500 nm from 10 μM **HBT3CN (i)**, **HBP3CN (k)** and **HBTH3CN (m)** in 1× pH = 7.4 PBS buffer in a 54 μL cuvette (3 mm optical pathlength) upon irradiation at 405 nm at various constant light intensities (in 10^{-5} E.m^{-2}.s^{-1}): 2.4 (circles), 3.5 (squares), 5.7 (triangles), 10.7 (diamonds), 22.9 (discs), 34.1 (pentagons) and 44.4 (hexagons). Markers: experimental data; solid lines: Monoexponential fit delivering τ (s): 37.7, 23.8, 13.9, 6.5, 3.8, 2.5 and 2.2 (for **HBT3CN**); 28.9, 18.7, 10.9, 5.1, 2.8, 2.2 and 1.4 (for **HBP3CN**) and 34.5, 22.7, 11.4, 6.1, 2.9, 1.9 and 1.7 (for **HBTH3CN**); **j,l,n**: Extraction of the effective cross section of photoisomerization for **HBT3CN (j)**, **HBP3CN (l)** and **HBTH3CN (n)** from the τ values retrieved in **i,k,m**. Markers: experimental data; solid line: linear fit with Eq.(S52). $T = 293$ K.

3.2.3 Measurements at 480 nm

The effective cross section of photoisomerization has been similarly measured at λ_{exc} = 480 nm. Figure S17a–l displays the results for **HBR3Cl** (Figure S17a,b), **HBR3CN** (Figure S17c,d), **HBR3Cl5F** (Figure S17e,f), **HBR35DF** (Figure S17g,h), **HPAR3Cl** (Figure S17i,j) and **HPAR3F5OM** (Figure S17k,l). Hence, we extracted 1040 ± 47, 1561 ± 31, 1367 ± 43, 1155 ± 62, 1296 ± 34 and 928 ± 29 $m^2.mol^{-1}$ at λ_{exc} = 480 nm for **HBR3Cl**, **HBR3CN**, **HBR3Cl5F**, **HBR35DF**, **HPAR3Cl** and **HPAR3F5OM** respectively.

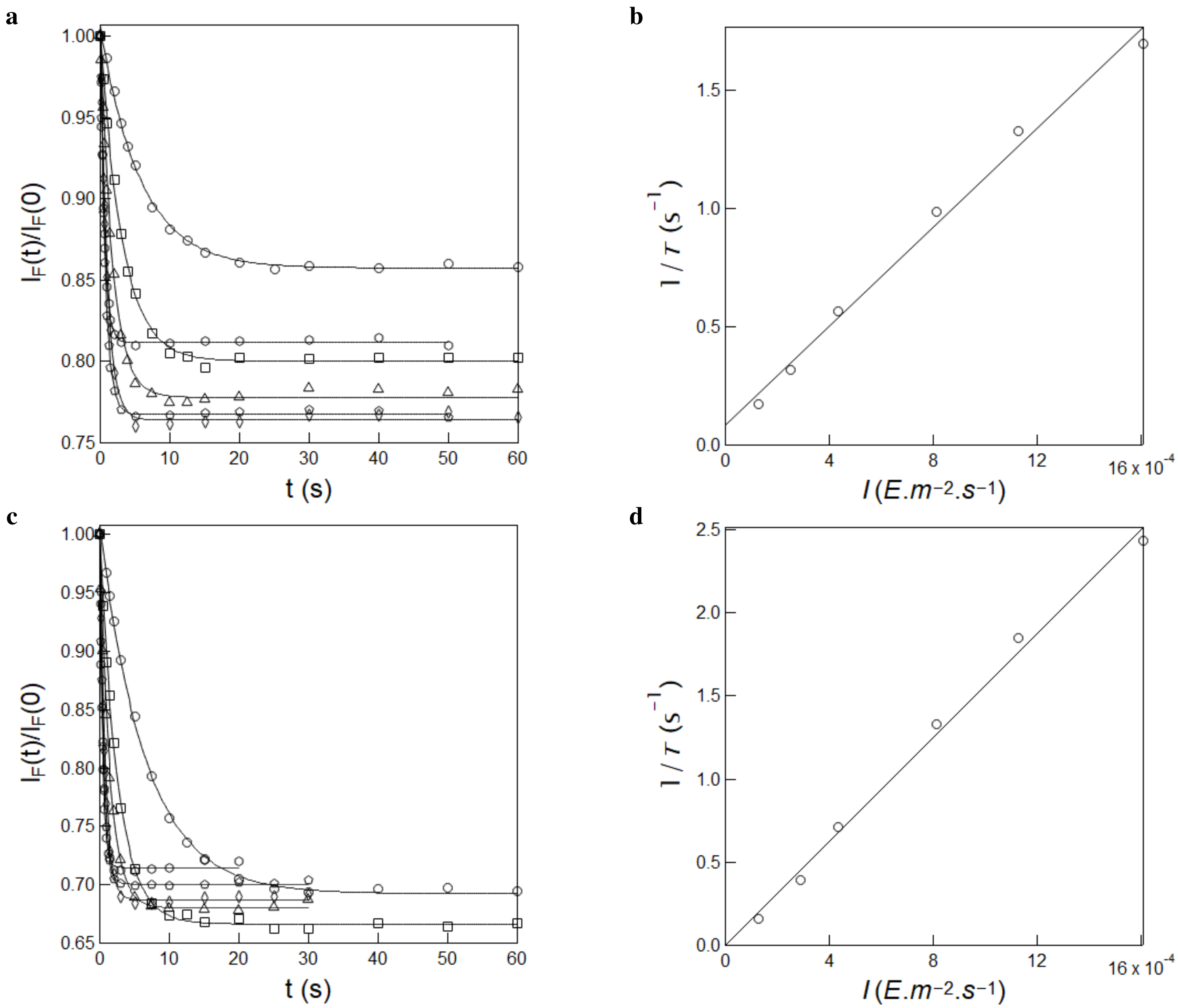

Figure S17: *Measurement of the effective cross section of photoisomerization of* ***HBR3Cl*** *and* ***HBR3CN*** under 480 nm illumination. **a,c**: Time evolution of the normalized fluorescence emission at 550 nm from 15 μM **HBR3Cl (a)** and **HBR3CN (c)** in 1× pH = 7.4 PBS buffer in a 54 μL cuvette (3 mm optical pathlength) upon irradiation at 480 nm at constant various light intensities (in 10^{-4} $E.m^{-2}.s^{-1}$): 1.3 (circles), 2.5 (squares), 4.4 (triangles), 8.2 (diamonds), 11.3 (pentagons) and 16.1 (hexagons). Markers: experimental data; solid lines: Monoexponential fit delivering τ (s): 5.8, 3.1, 1.8, 1.0, 0.7, and 0.6 (for **HBR3Cl**) and 6.5, 2.6, 1.5, 0.8, 0.5, and 0.4 (for **HBR3CN**); **b,d**: Extraction of the effective cross section of photoisomerization for **HBR3Cl (b)** and **HBR3CN (d)** from the τ values retrieved in **a,c**. Markers: experimental data; solid line: linear fit with Eq.(S52). $T = 293$ K.

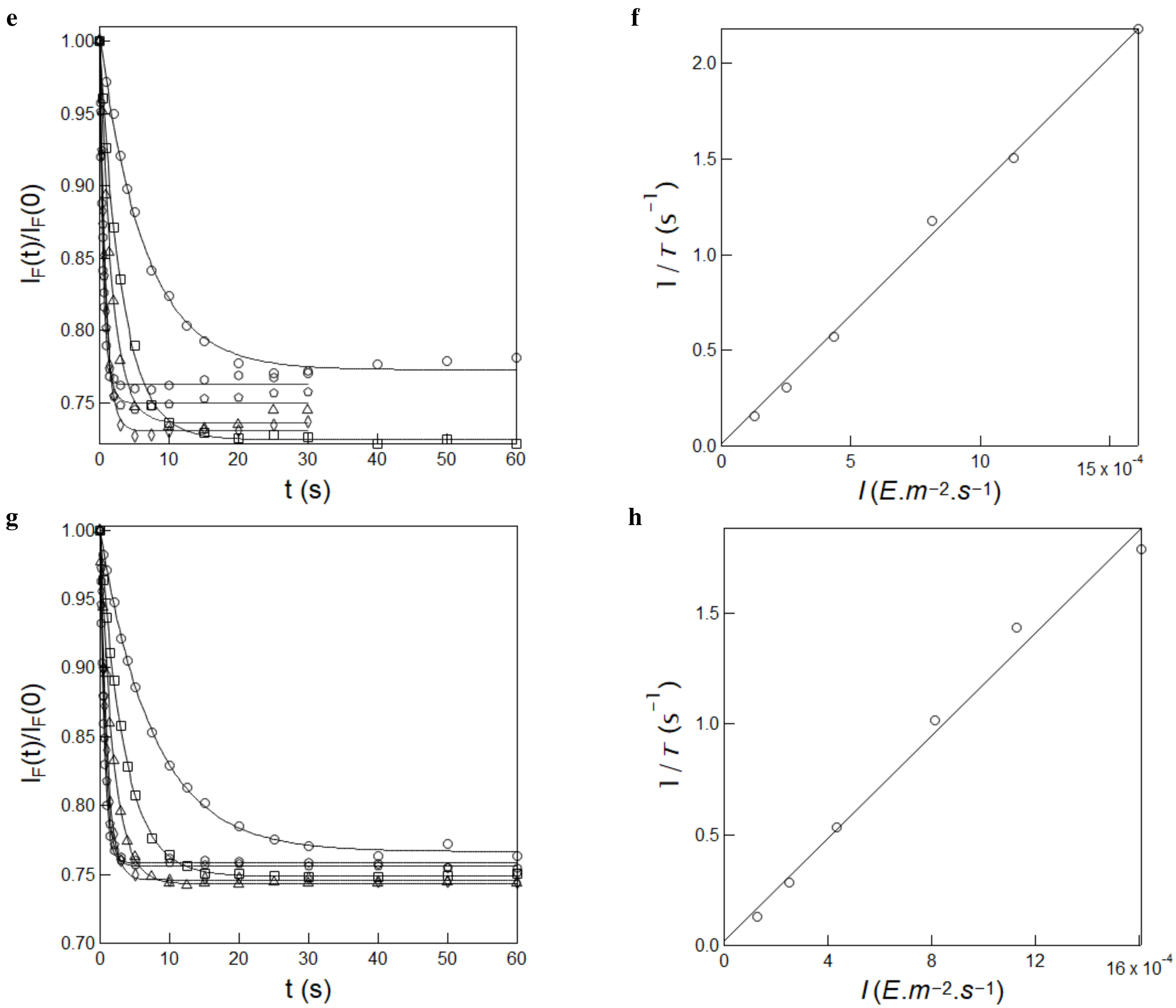

Figure S17: *Measurement of the effective cross section of photoisomerization of* ***HBR3Cl5F*** *and* ***HBR35DF*** under 480 nm illumination. **a,c**: Time evolution of the normalized fluorescence emission at 550 nm from 15 μM **HBR3Cl5F (e)** and **HBR35DF (g)** in 1× pH = 7.4 PBS buffer in a 54 μL cuvette (3 mm optical pathlength) upon irradiation at 480 nm at constant various light intensities (in 10^{-4} E.m^{-2}.s^{-1}): 1.3 (circles), 2.5 (squares), 4.4 (triangles), 8.2 (diamonds), 11.3 (pentagons) and 16.1 (hexagons) Markers: experimental data; solid lines: Monoexponential fit delivering τ (s): 6.4, 3.3, 1.7, 0.9, 0.7, and 0.4 (for **HBR3Cl5F**) and 7.7, 3.5, 1.9, 1.0, 0.7, and 0.6 (for **HBR35DF**); **f,h**: Extraction of the effective cross section of photoisomerization for **HBR3Cl5F (f)** and **HBR35DF (h)** from the τ values retrieved in **e,g**. Markers: experimental data; solid line: linear fit with Eq.(S52). $T = 293$ K.

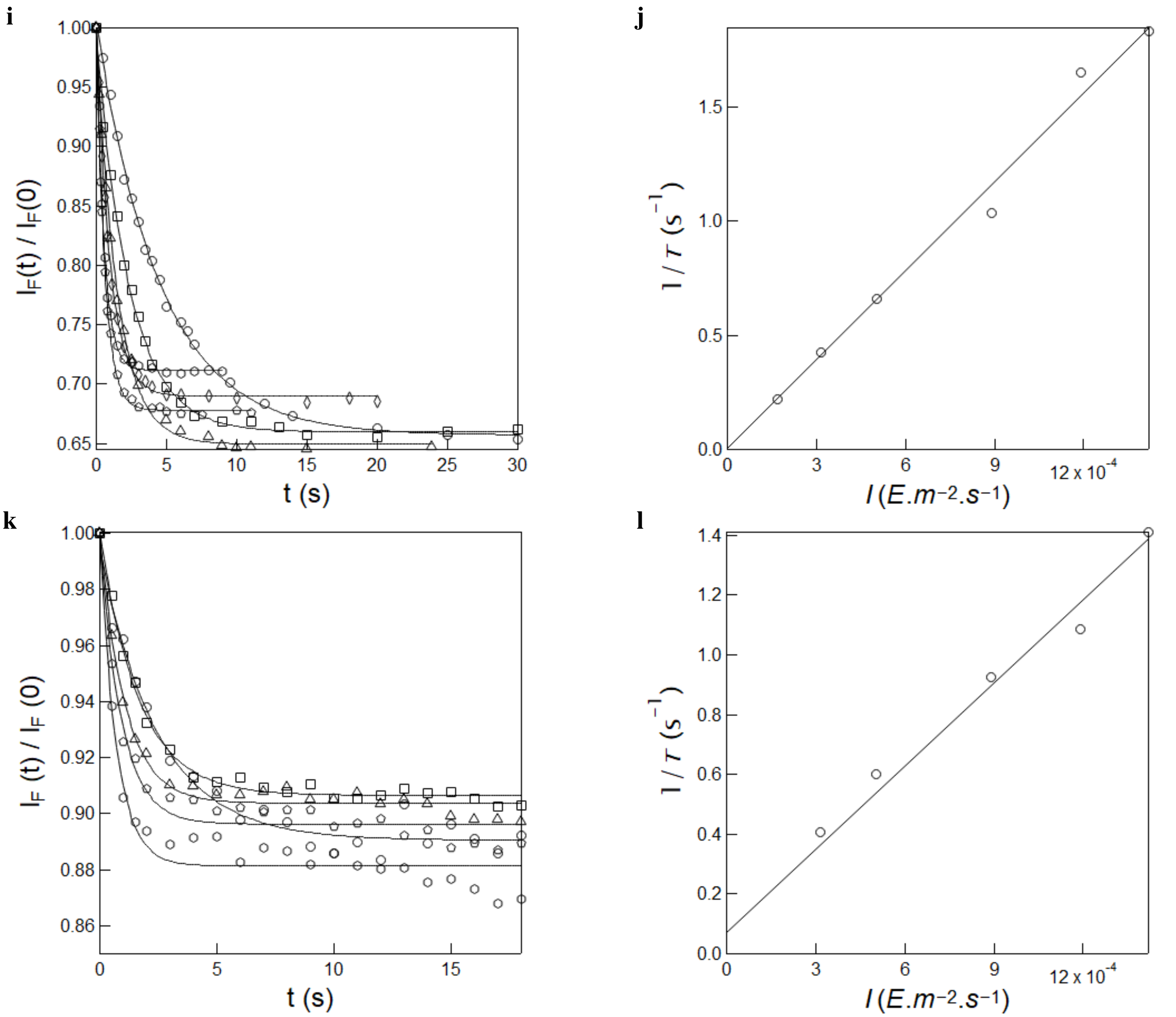

Figure S17: *Measurement of the effective cross section of photoisomerization of **HPAR3Cl** and **HPAR3F5OM** under 480 nm illumination.* **i,k**: Time evolution of the normalized fluorescence emission at 500 nm from 10 μM **HPAR3Cl (i)** and **HPAR3F5OM (k)** in 1× pH = 7.4 PBS buffer in a 54 μL cuvette (3 mm optical pathlength) upon irradiation at 480 nm at various constant light intensities (in 10^{-4} E.m^{-2}.s^{-1}): 1.7 (circles), 3.2 (squares), 5.0 (triangles), 8.9 (diamonds), 11.9 (pentagons) and 14.2 (hexagons) with **HPAR3Cl** and 3.2 (circles), 5.0 (squares), 8.9 (triangles), 11.9 (pentagons) and 14.2 (hexagons) with **HPAR3F5OM**. Markers: experimental data; solid lines: Monoexponential fit delivering τ (s): 4.5, 2.3, 1.5, 1.0, 0.6 and 0.5 (for **HPAR3Cl**) and 2.5, 1.7, 1.1, 0.9, and 0.7 (for **HPAR3F5OM**); **j,l**: Extraction of the effective cross section of photoisomerization for **HPAR3Cl (j)** and **HPAR3F5OM (l)** from the τ values retrieved in **i,k**. Markers: experimental data; solid line: linear fit with Eq.(S52). $T = 293$ K.

3.3 Kinetics of thermal recovery from the photoisomerized state of the free fluorogen

To measure the effective rate constant associated with thermal recovery of the free fluorogens, 54 μL of 10 μM fluorogen solution in 1$\times$ pH = 7.4 PBS buffer was repeatedly illuminated with 365 (I=13.1×10^{-4} E.m^{-2}.s^{-1}), 405 (I = 3.7×10^{-4} E.s^{-1}.m^{-2}) or 480 (I=14.2×10^{-4} E.m^{-2}.s^{-1}) nm LED, and the fluorescence signal was monitored at the respective emission maximum until the photostationary state was reached. These illumination steps were separated by increasing delay times (Δt), where solutions were kept at complete darkness. Recovery of the fluorescence signal was measured and plotted against the corresponding delay time Δt. The relaxation time associated with thermal recovery of the photoisomerized state was then extracted from the monoexponentially fitting of the dependence of the fluorescence recovery on the delay time Δt by using Eq.(S51). The effective rate constant associated with thermal recovery was eventually retrieved as the inverse of the extracted characteristic time.

The fluorescence level recovered after each relaxation in the darkness was satisfactorily fitted monoexponentially with Eq.(S51) for most of the investigated fluorogens. Hence, we extracted 13.0 ± 0.7, 3359 ± 336, 96 ± 5, 54 ± 5, 16 ± 1, 7 ± 0.4, 238 ± 15, and 15 ± 3 s for the characteristic time of thermal return for **HBR3Cl** (Figure S18b), **HBR3CN** (Figure S18d), **HBR3Cl5F** (Figure S18f), and **HBR35DF** (Figure S18h), **HBT35DM** (Figure S18j) and **HBT35DOM** (Figure S18l),**HPAR3Cl** (Figure S18n) and **HPAR3F5OM** (Figure S18p) respectively, which finally yielded $k^{\Delta}_{F'F} = 0.08$, 3.0×10^{-4}, 0.01, 0.02, 0.06, 0.14, 4.2×10^{-3}, and 0.07 s^{-1} at 293 K for **HBR3Cl**, **HBR3CN**, **HBR3Cl5F**, **HBR35DF**, **HBT35DM**, **HBT35DOM**, **HPAR3Cl** and **HPAR3F5OM** respectively.

In contrast, although exhibit photoisomerization under illumination at 365 nm, we noticed that the photoisomerized state of **HBO3M**, **HBO35DM**, **HBT3CN**, **HBP3CN** and **HBTH3CN** essentially did not recover over a time scale of an hour (Figure S19a–e).

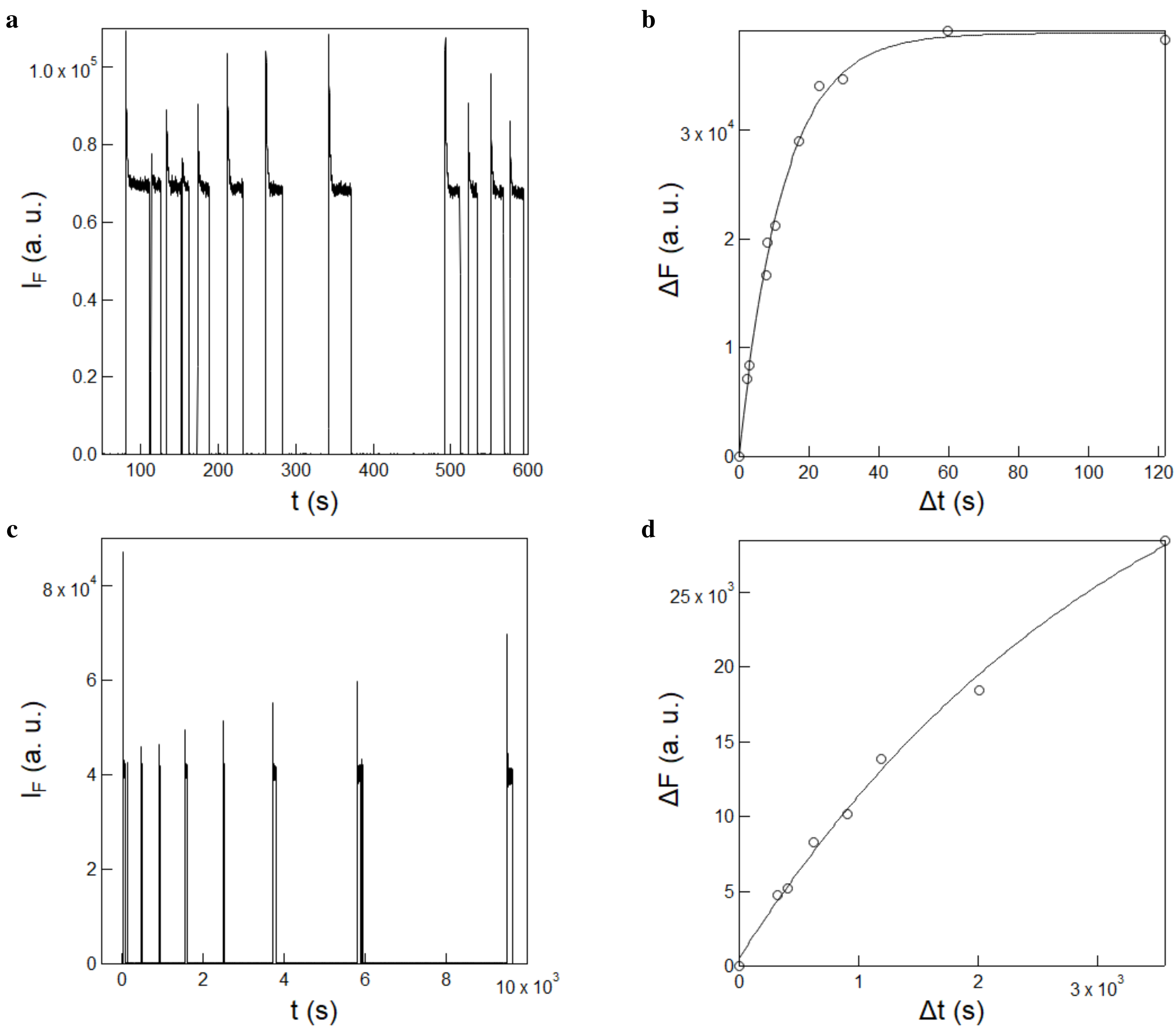

Figure S18: *Measurement of the rate constant of thermal recovery from the photoisomerized state of **HBR3Cl** and **HBR3CN***. **a,c**: Time evolution of the fluorescence emission at 550 nm from 10 μM **HBR3Cl** (**a**) and **HBR3CN** (**c**) in 1× pH = 7.4 PBS buffer exposed to a sequence of long enough 405 nm light pulses at 2.3×10^{-4} E.m^{-2}.s^{-1} to reach the photostationary state while varying the time of recovery in the dark in between the pulses; **b,d**: Extraction of the rate constant associated with the thermal return of the photoisomerized **HBR3Cl** (**b**) and **HBR3CN** (**d**) stereoisomer from the recovery of fluorescence signal against the delay time (Δt) retrieved in **a** and **c**. Markers: experimental data; solid line: monoexponential fit using Eq.(S51). $T = 293$ K.

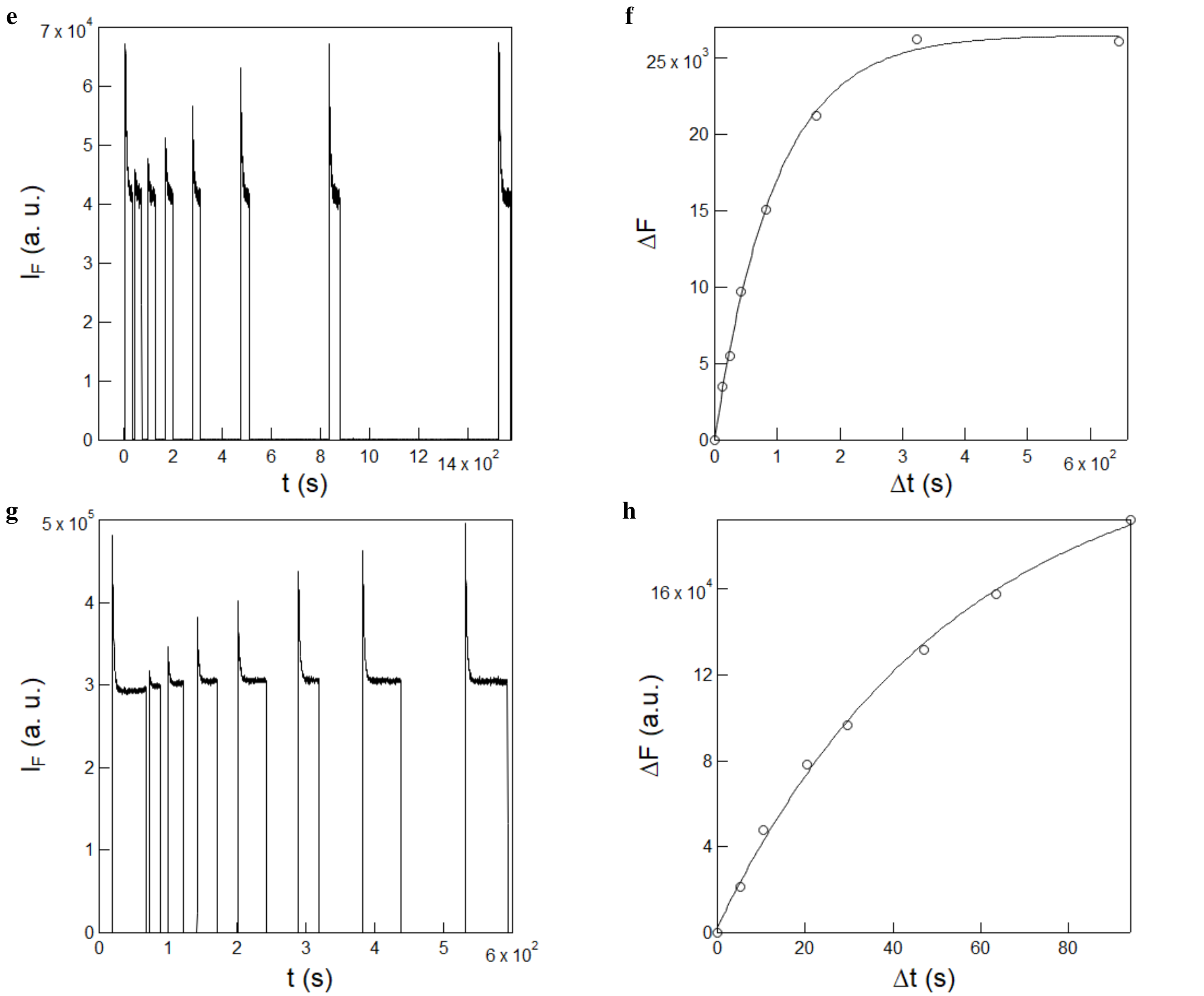

Figure S18: *Measurement of the rate constant of thermal recovery from the photoisomerized state of* ***HBR3Cl5F*** *and* ***HBR35DF***. **e,g**: Time evolution of the fluorescence emission at 550 nm from 10 μM **HBR3Cl5F** (**e**) and **HBR35DF** (**g**) in 1$\times$ pH = 7.4 PBS buffer exposed to a sequence of long enough 405 nm light pulses at 2.3$\times 10^{-4}$ E.m^{-2}.s^{-1} to reach the photostationary state while varying the time of recovery in the dark in between the pulses; **f,h**: Extraction of the rate constant associated with the thermal return of the photoisomerized **HBR3Cl5F** (**f**) and **HBR35DF** (**h**) stereoisomer from the recovery of fluorescence signal against the delay time (Δt) retrieved in **e** and **g**. Markers: experimental data; solid line: monoexponential fit using Eq.(S51). $T = 293$ K.

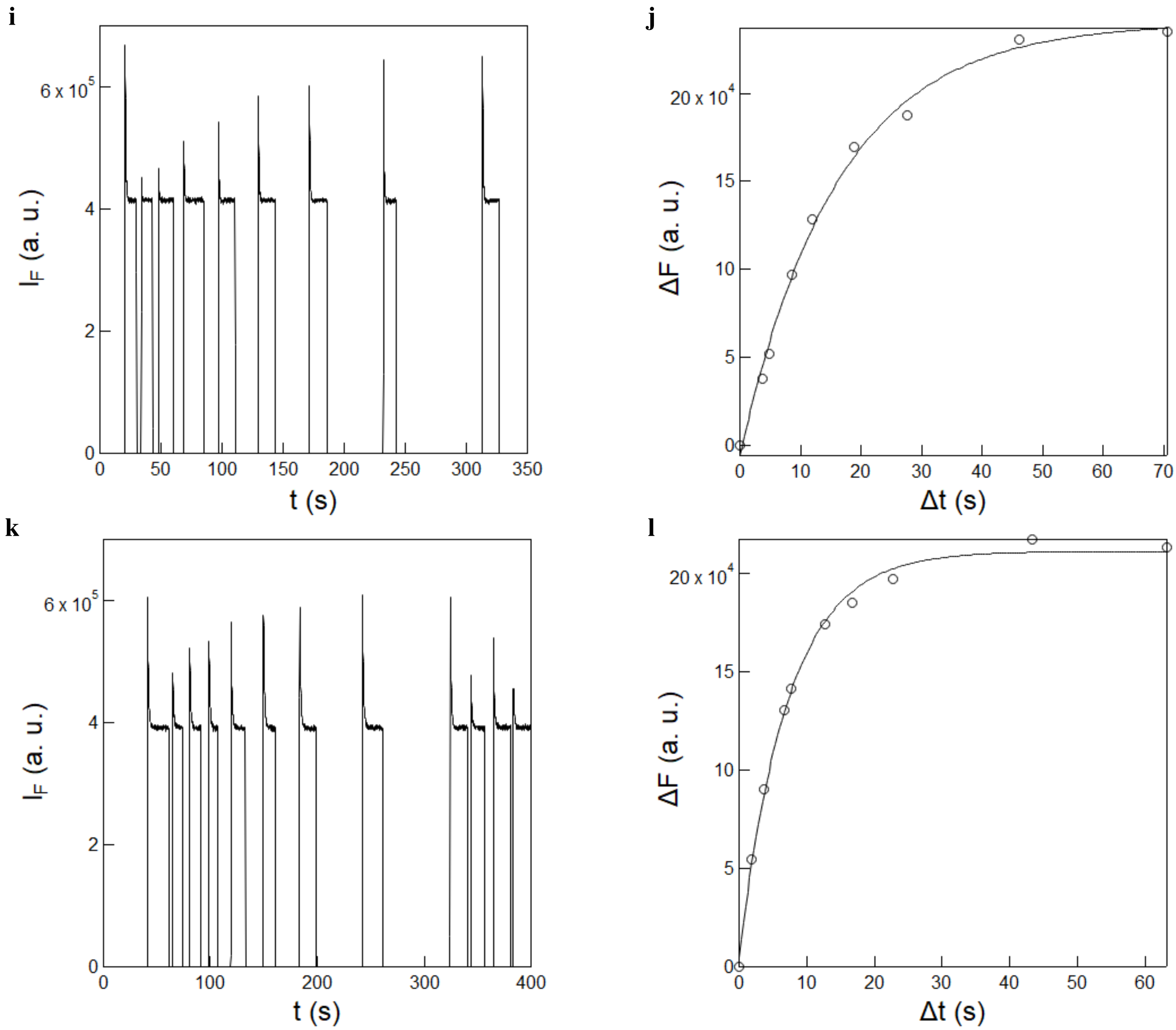

Figure S18: *Measurement of the rate constant of thermal recovery from the photoisomerized state of* ***HBT35DM*** *and* ***HBT35DOM***. **i,k**: Time evolution of the fluorescence emission at 500 nm from 10 μM **HBT35DM** (**i**) and **HBT35DOM** (**k**) in 1$\times$ pH = 7.4 PBS buffer exposed to a sequence of long enough 365 nm light pulses at 13.1$\times10^{-4}$ E.m^{-2}.s^{-1} to reach the photostationary state while varying the time of recovery in the dark in between the pulses; **j,l**: Extraction of the rate constant associated with the thermal return of the photoisomerized **HBT35DM** (**j**) and **HBT35DOM** (**l**) stereoisomer from the recovery of fluorescence signal against the delay time (Δt) retrieved in **i** and **k**. Markers: experimental data; solid line: monoexponential fit using Eq.(S51). T = 293 K.

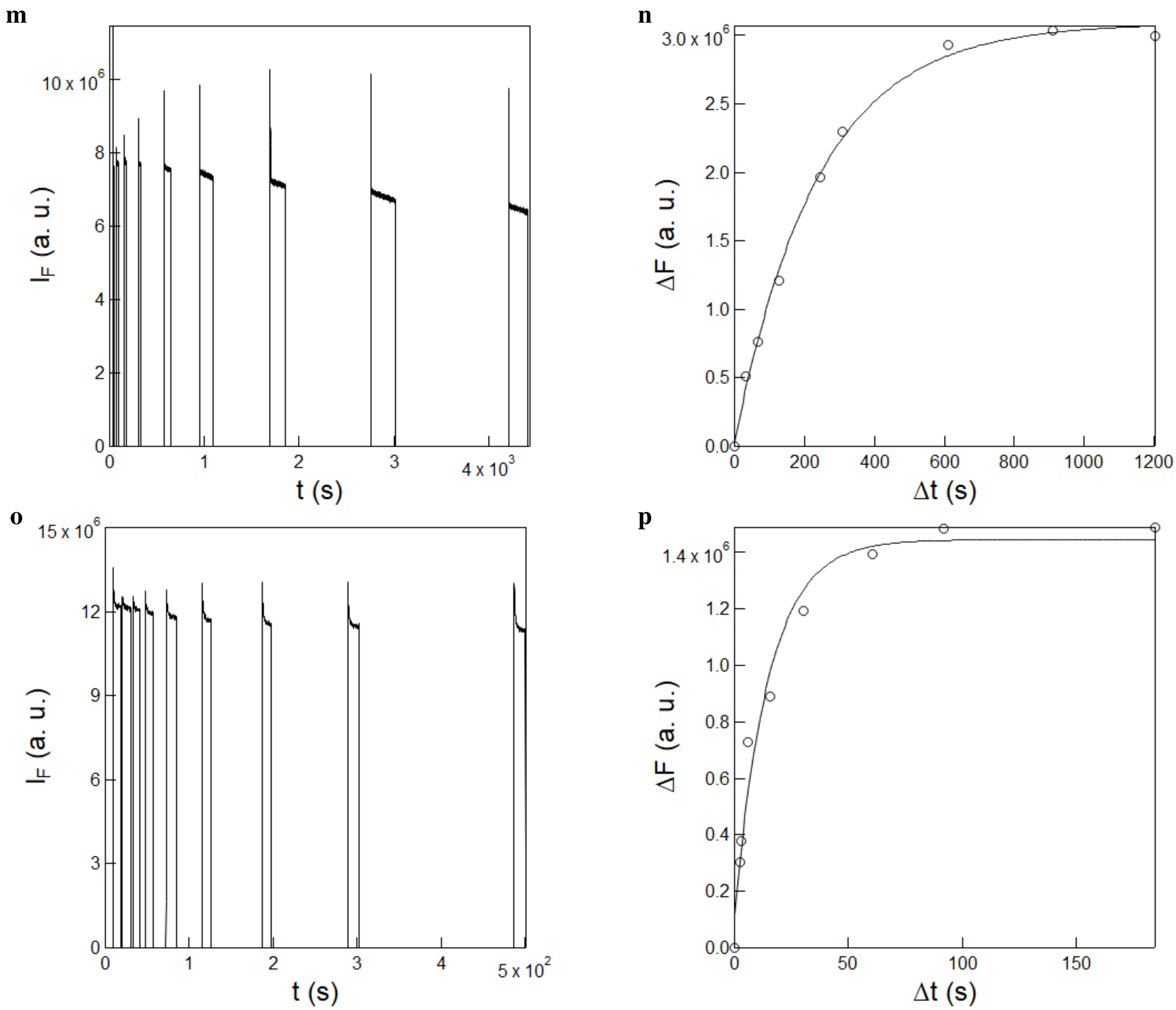

Figure S18: *Measurement of the rate constant of thermal recovery from the photoisomerized state of* ***HPAR3Cl*** *and* ***HPAR3F5OM***. **m,o**: Time evolution of the fluorescence emission at 660 nm from 10 μM **HPAR3Cl** (**m**) and **HPAR3F5OM** (**o**) in 1$\times$ pH = 7.4 PBS buffer exposed to a sequence of long enough 480 nm light pulses at 14.2×10^{-4} E.m^{-2}.s^{-1} to reach the photostationary state while varying the time of recovery in the dark in between the pulses; **n,p**: Extraction of the rate constant associated with the thermal return of the photoisomerized **HPAR3Cl** (**n**) and **HPAR3F5OM** (**p**) stereoisomer from the recovery of fluorescence signal against the delay time (Δt) retrieved in **m** and **o**. Markers: experimental data; solid line: monoexponential fit using Eq.(S51). $T = 293$ K.

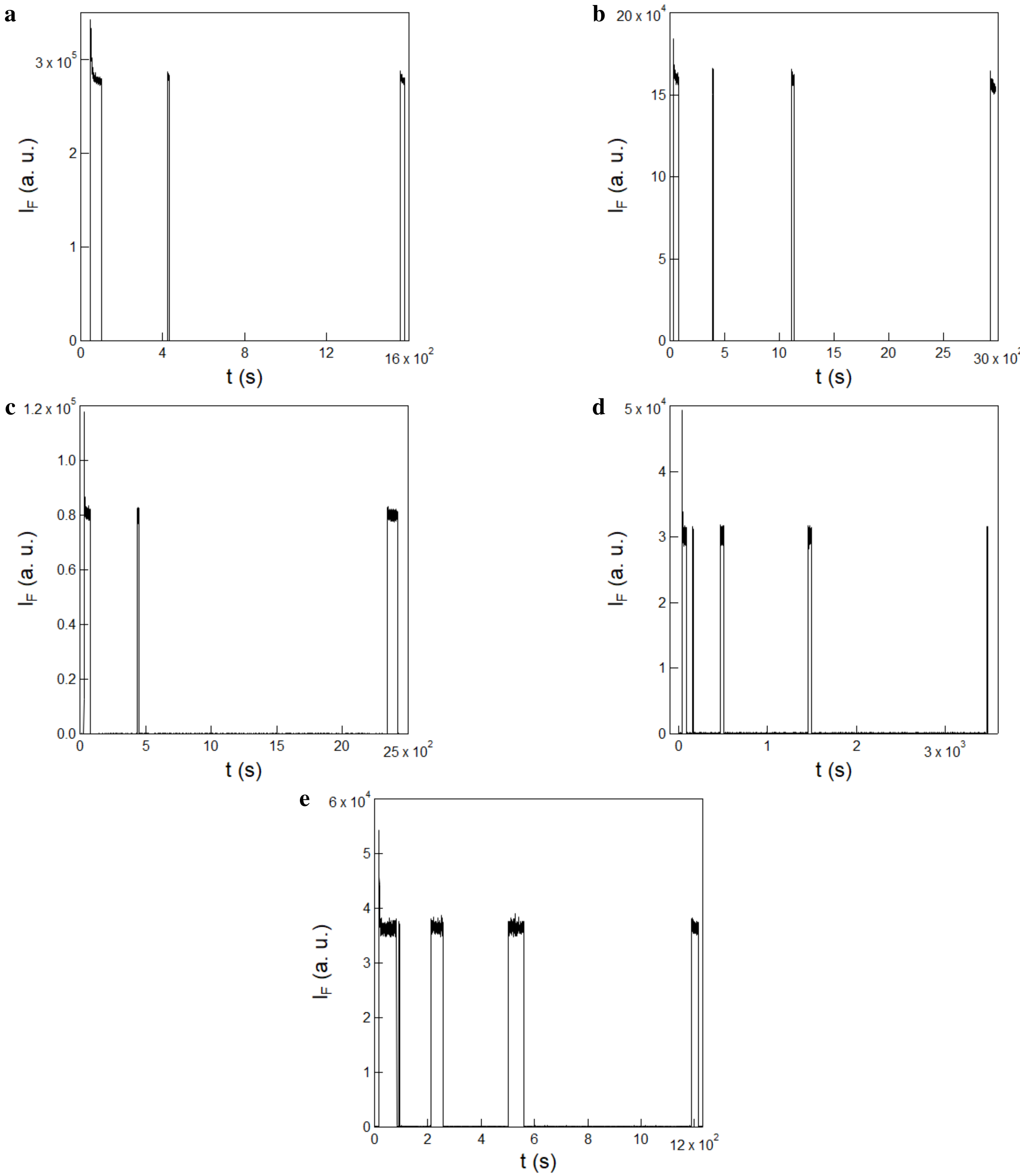

Figure S19: *Attempt to measure the rate constant of thermal recovery from the photoisomerized state of* ***HBO3M***, ***HBO35DM***, ***HBT3CN***, ***HBP3CN*** *and* ***HBTH3CN***. Time evolution of the fluorescence emission at 500 nm from 20 μM **HBO3M, HBO35DM** and 10 μM **HBT3CN**, **HBP3CN** and **HBTH3CN** in 1$\times$ pH = 7.4 PBS buffer exposed to a sequence of long enough 365 nm (15.1×10^{-4} E.m^{-2}.s^{-1}) for **HBO3M**, **HBO35DM** or 405 nm (1.1×10^{-4} E.m^{-2}.s^{-1}) for **HBT3CN**, **HBP3CN** and **HBTH3CN** light pulses to reach the photostationary state while varying the time of recovery in the dark in between the pulses. $T = 293$ K.

We conducted analogous photochemical experiments on **HBR3F5OM**, **HBIR3Cl**, **HBR25DM** and **HBR35DOM** using either 405 or 480 nm LED as the light source. Under identical conditions, we observed a constant fluorescence intensity over time for these fluorogens (Figure S20a,b). This observation may have led us to deduce that these fluorogens do not undergo photoisomerization. However, except for **HBR35DOM**, their photoisomerization was observed in imaging experiments performed with confocal microscopy. Hence, we rather concluded that these fluorogens possess extremely rapid thermal recovery (<1 s) so as to forbid to observe any fluorescence change upon applying a light jump at the light intensity (10^{-3} E.m^{-2}.s^{-1} range) employed.

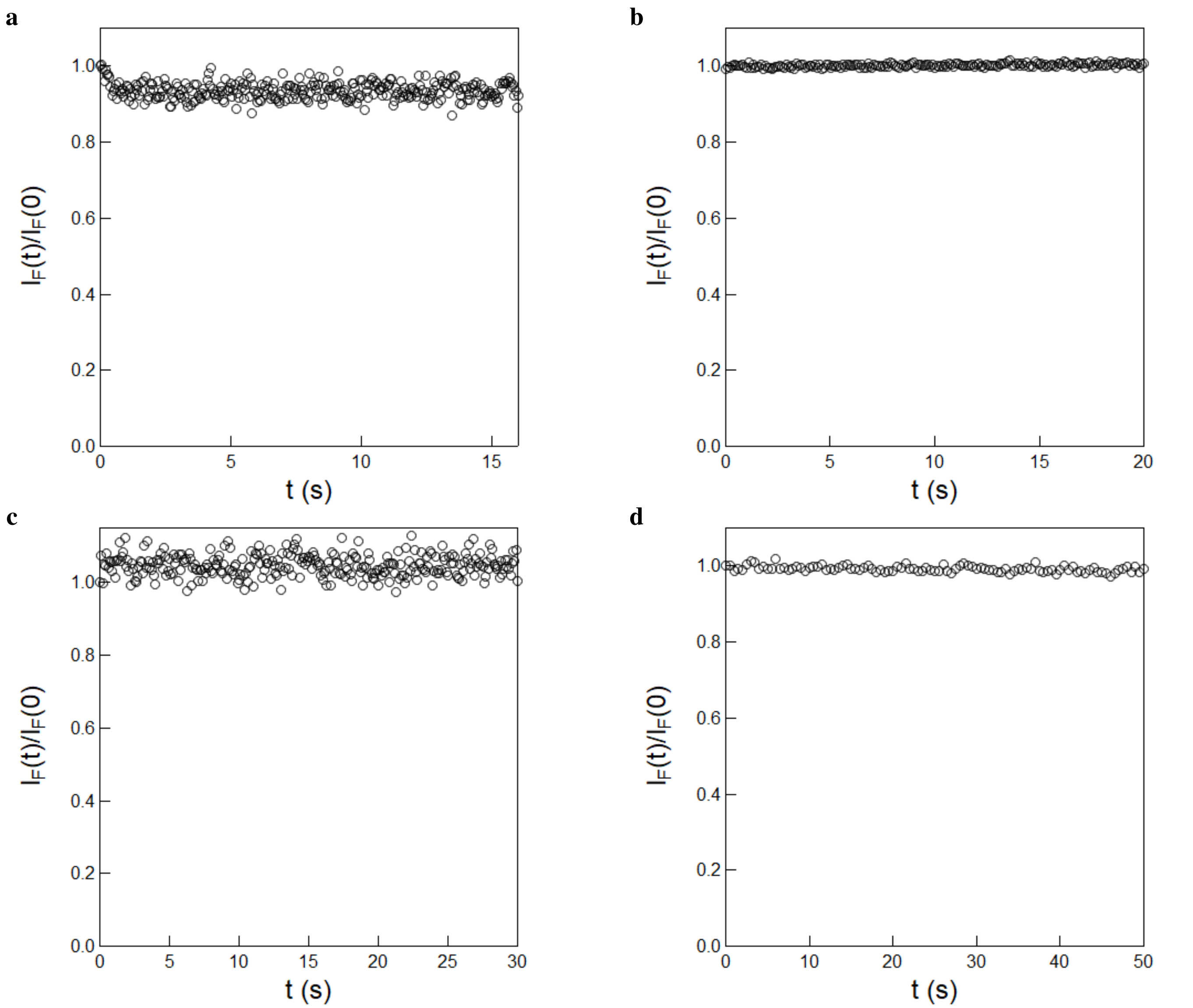

Figure S20: *An attempt to measure the rate constant of thermal recovery from the photoisomerized state of* ***HBR3F5OM****,* ***HBIR3Cl****,* ***HBR25DM*** *and* ***HBR35DOM***. Time evolution of the fluorescence emission at 550 nm from 10 μM **HBR3F5OM (a)**, **HBIR3Cl (b)**, **HBR25DM (c)** and **HBR35DOM (d)** in 1× pH = 7.4 PBS buffer in a 54 μL cuvette (3 mm optical pathlength) upon irradiation at 405 nm (for **HBR3F5OM**, **HBR25DM** and **HBR35DOM**) and 480 nm (for **HBIR3Cl**) at constant light intensity. $T = 293$ K.

3.4 Measurement of the proton exchange constants of the free fluorogens

To determine the proton exchange constant of the free fluorogens in their (*Z*) thermodynamically stable state, we used 2–4 μM fluorogen solutions in 10 mM universal buffer prepared as follows: To 100 mL of Milli-Q water were dissolved 82 mg of sodium acetate (CH_3COONa), 120 mg of sodium dihydrogenophosphate (NaH_2PO_4), and 518.2 mg of 2-(cyclohexylamino)ethane-1-sulfonic acid (CHES). The pH of the initial solution (pH = 5.90) was then adjusted to pH = 9 with 1 M NaOH. The dependence of the absorption spectrum obeying Eq.(S50) was globally analyzed by exploiting the SPECFIT/32TM Global Analysis System (Version 3.0 for 32-bit Windows systems)[14–17] to extract $K_{\mathbf{FH}}$.
Figures S21a–m display the pH-dependence of the absorption spectrum of **HBR3Cl**, **HBR3CN**, **HBR3Cl5F**, **HBR35DF**, **HBR3F5OM**, **HBIR3Cl**, **HBT35DOM**, **HBIR35DOM**, **HBT3CN**, **HBP3CN**, **HBTH3CN**, **HPAR3Cl**, and **HPAR3F5OM**. The extracted values of p$K_{\mathbf{FH}}$ are given in Table S2.

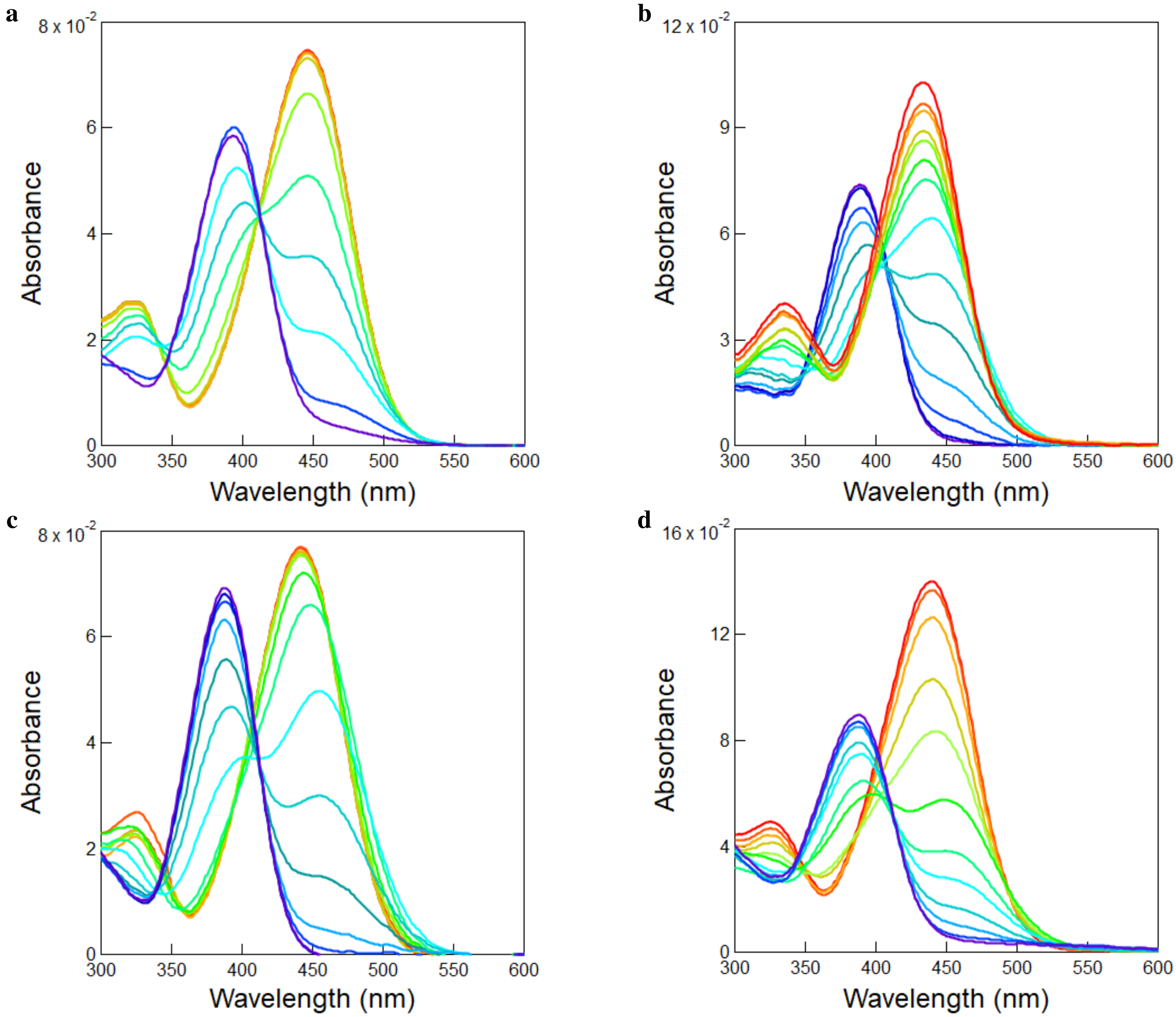

Figure S21: *Determination of the proton exchange constant K_{FH} of the free fluorogens*. pH-dependence of the absorption spectrum of 2.4 μM **HBR3Cl (a)**, 3 μM **HBR3CN (b)**, 2.4 μM **HBR3Cl5F (c)** and 4 μM **HBR35DF (d)** in 10 mM universal buffer. The absorption spectra are displayed with rainbow colors upon evolving from red (at the highest investigated pH) to violet (at the lowest investigated pH): pH = 9.9, 9.4, 9.0, 8.5, 7.7, 7.1, 6.7, 6.2, 5.4, 4.8 for **HBR3Cl**; pH = 9.0, 8.8, 8.4, 7.8, 7.5, 7.1, 6.7, 6.1, 5.6, 5.0, 4.6, 4.2, 3.1, 2.6 for **HBR3CN**; pH = 9.7, 8.8, 8.3, 7.5, 7.0, 6.6, 6.0, 5.2, 4.7, 4.3, 3.7, 3.3, 2.9, 2.7 for **HBR3Cl5F**; pH = 9.0, 8.5, 7.8, 7.1, 6.6, 5.7, 5.3, 4.9, 4.6, 4.2, 3.6, 3.0 for **HBR35DF**. The extracted values of p$K_{\mathbf{FH}}$ are 6.8, 5.1, 4.8 and 5.3 for **HBR3Cl**, **HBR3CN**, **HBR3Cl5F** and **HBR35DF** respectively. $T = 293$ K.

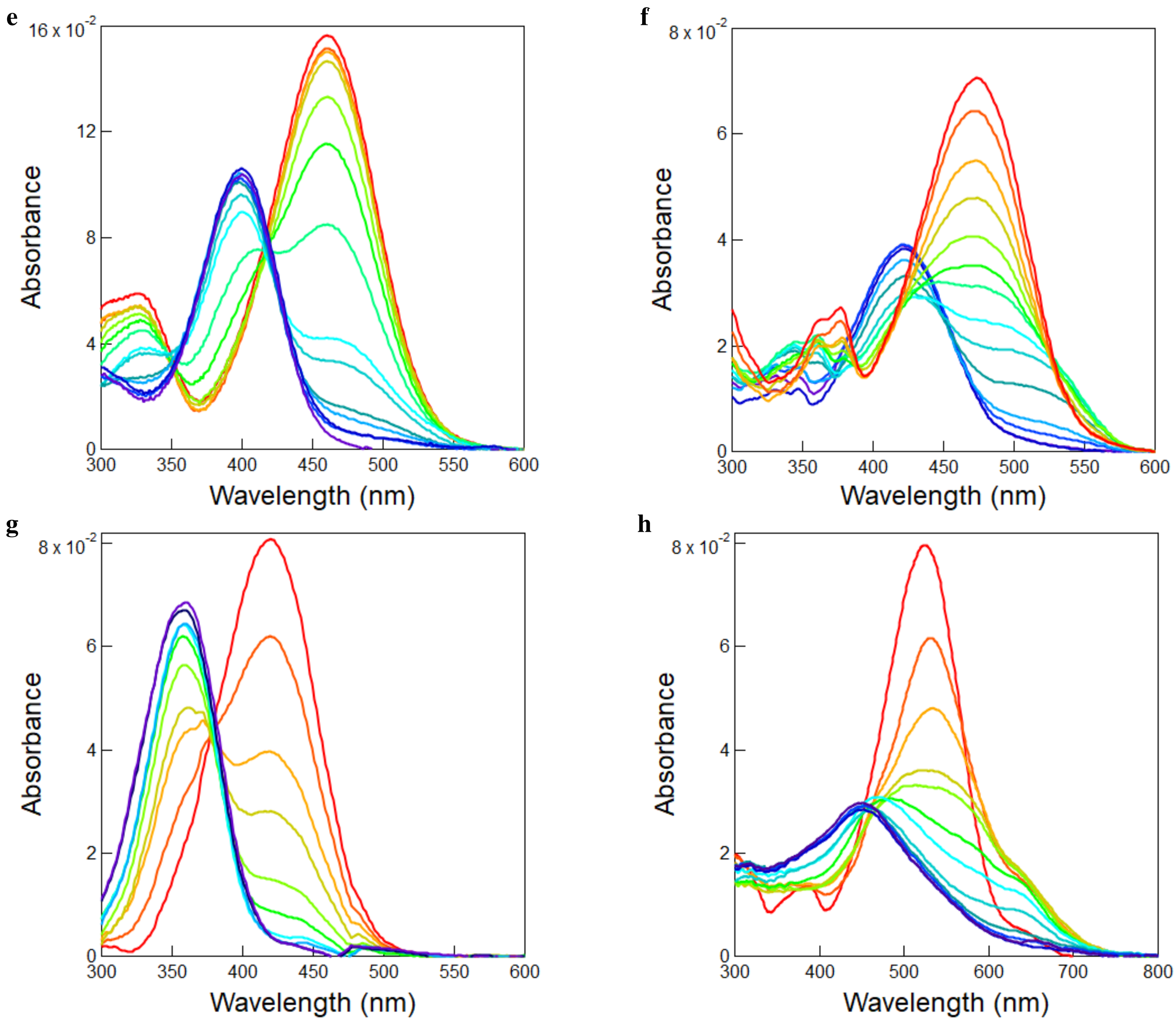

Figure S21: *Determination of the proton exchange constant K_{FH} of the free fluorogens*. pH-dependence of the absorption spectrum of **HBR3F5OM (e)**, **HBIR3Cl (f)**, **HBT35DOM (g)** and **HBIR35DOM (h)** recorded at 3 μM in 10 mM universal buffer. The absorption spectra are displayed with rainbow colors upon evolving from red (at the highest investigated pH) to violet (at the lowest investigated pH): pH = 9.9, 9.5, 9.0, 8.6, 8.0, 7.5, 7.0, 6.5, 6.1, 5.5, 5.1, 4.7, 3.9, 3.3 for **HBR3F5OM**, pH = 3.0, 3.8, 4.8, 5.4, 6.0, 6.3, 6.7, 6.9, 7.1, 7.3, 7.7, 8.1, 8.8, 9.1 for **HBIR3Cl**, pH = 9.6, 9.0, 8.6, 8.0, 7.7, 7.3, 6.9, 6.5, 5.5, 4.5 for **HBT35DOM** and pH = 9.8, 9.3, 8.9, 8.6, 7.9, 7.7, 7.1, 6.8, 6.3, 5.2, 4.6, 3.9 for **HBIR35DOM**. The extracted values of p$K_{\mathbf{FH}}$ are 6.9, 7.1, 8.6 and 8.4 for **HBR3F5OM**, **HBIR3Cl**, **HBT35DOM** and **HBIR35DOM** respectively. $T = 293$ K.

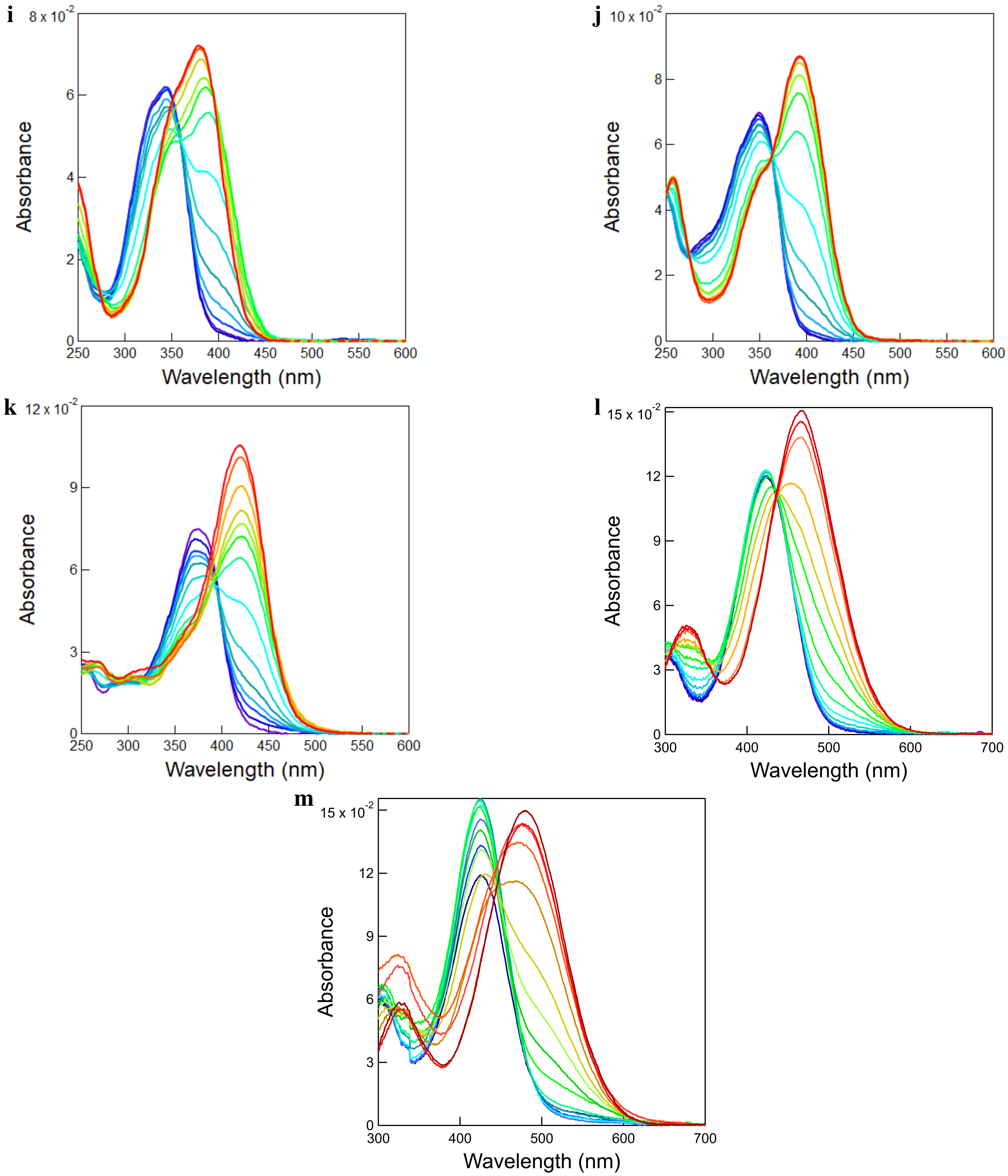

Figure S21: *Determination of the proton exchange constant* K_{FH} *of the free fluorogens*. pH-dependence of the absorption of **HBT3CN (i)**, **HBP3CN (j)**, **HBTH3CN (k)** recorded at 3 μM and **HPAR3Cl (l)**, **HPAR3F5OM (m)** recorded at 4 μM in 10 mM universal buffer. The absorption spectra are displayed with rainbow colors upon evolving from red (at the highest investigated pH) to violet (at the lowest investigated pH): pH = 9.0, 8.8, 8.4, 7.8, 7.5, 7.1, 6.7, 6.1, 5.6, 5.0, 4.6, 4.2, 3.1, 2.6 for **HBR3CN**; pH = 9.0, 8.6, 8.1, 7.4, 6.8, 6.5, 6.1, 5.5, 5.1, 4.8, 4.4, 3.9, 3.1, 2.6 for **HBT3CN**; pH = 9.0, 8.6, 8.1, 7.4, 6.9, 6.6, 6.2, 5.6, 5.1, 4.8, 4.5, 4.0, 3.2, 2.6 for **HBP3CN**, pH = 9.0, 8.7, 8.1, 7.3, 7.0, 6.6, 6.3, 5.8, 5.3, 4.9, 4.6, 4.3, 3.4, 2.7 for **HBTH3CN**, pH = 2.9, 3.1, 3.8, 4.2, 4.6, 4.6, 4.9, 5.2, 5.6, 5.9, 6.5, 6.8, 7.1, 7.2, 7.5, 8.5, 8.8, 9.1 for **HPAR3Cl** and pH = 3.8, 4.1, 4.7, 4.9, 5.4, 5.9, 6.5, 7.0, 7.3, 7.6, 8.1, 9.0 for **HPAR3F5OM**. The extracted pK_a values are 5.3, 5.2, 5.6, 7.7 and 7.7 for **HBT3CN**, **HBP3CN**, **HBTH3CN**, **HPAR3Cl** and **HPAR3F5OM** respectively. T = *293 K*.

Table S2: *Proton exchange properties of the (Z) stereoisomer of the free fluorogens.* Solvent: 1× pH = 7.4 PBS buffer. $T = 293$ K.

Fluorogens	pK_a
**HBR3Cl5F**	4.8
**HBR3CN**	5.1
**HBP3CN**	5.2
**HBT3CN**	5.3
**HBR35DF**	5.3
**HBTH3CN**	5.6
**HBR3Cl**	6.8
**HBR3F5OM**	6.9
**HBIR3Cl**	7.1
**HPAR3Cl**	7.7
**HPAR3F5OM**	7.7
**HBIR35DOM**	8.4
**HBT35DOM**	8.6

4 Investigation of the pFAST-fluorogen and nirFAST-fluorogen complexes

4.1 Absorption and emission spectra

The absorption and emission spectra of the **pFAST**-**fluorogen** and **nirFAST**-**fluorogen** complexes in 1× pH = 7.4 PBS buffer have been recorded in 54 μL quartz cuvette (3 mm optical pathlength) at protein concentration much higher than the nanomolar range of the dissociation constant and with an excess of protein over the fluorogen.

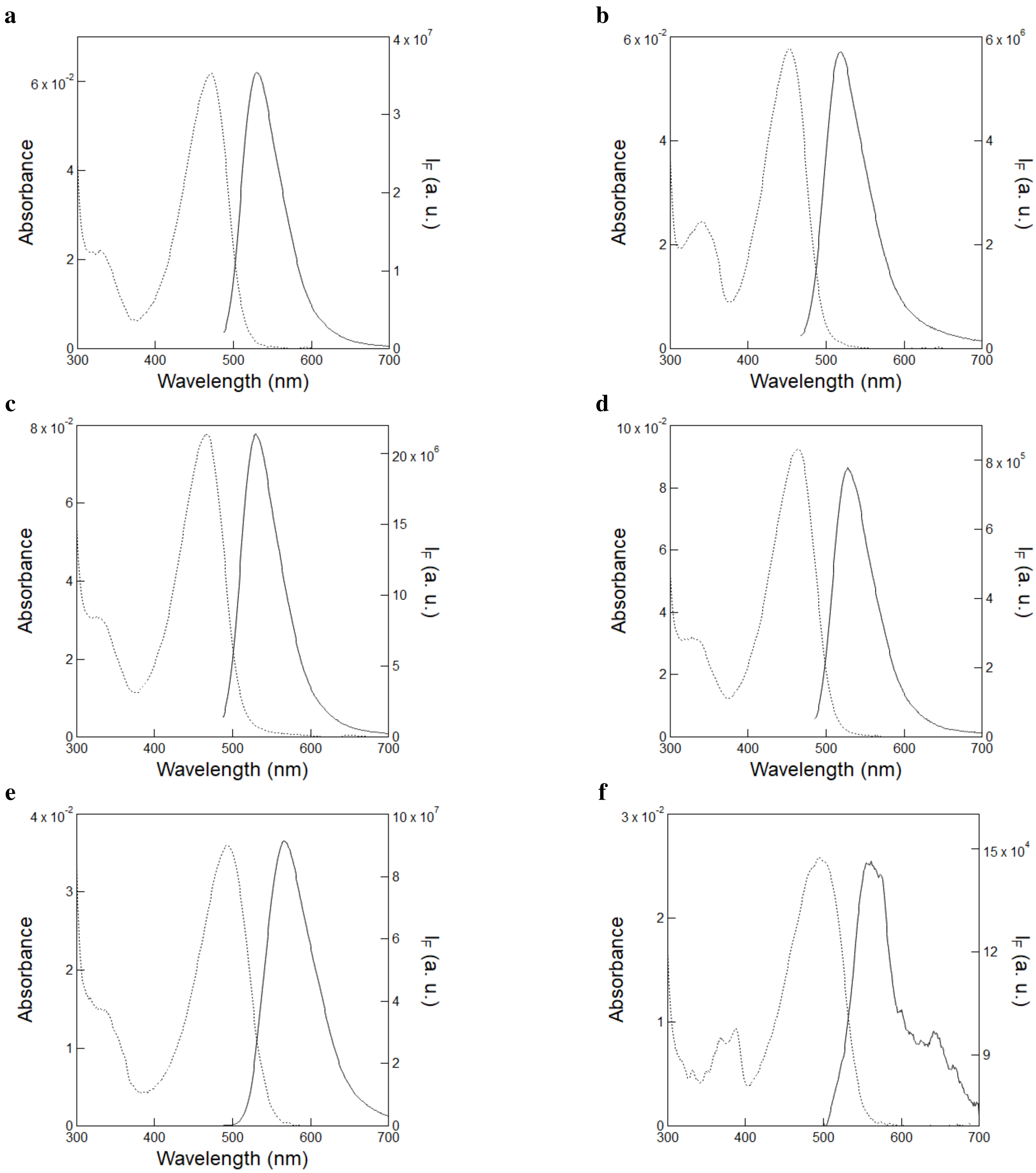

Figure S22: *UV-Vis absorption and emission spectra of the complexes between* ***pFAST*** *and the fluorogens.* The absorption (dotted line) and emission (solid line; λ_{exc} = 480 nm) spectra with **HBR3Cl (a)**, **HBR3CN (b)**, **HBR3Cl5F (c)**, **HBR35DF (d)**, **HBR3F5OM (e)** and **HBIR3Cl (f)** were recorded at 5 μM (for **HBR3Cl**, **HBR3CN**, **HBR3Cl5F**, **HBR35DF**), 4 μM (**HBR3F5OM**), and 3 μM (**HBIR3Cl**) in the presence of 32 μM **pFAST** in 1$\times$ pH = 7.4 PBS buffer. T = 293 K.

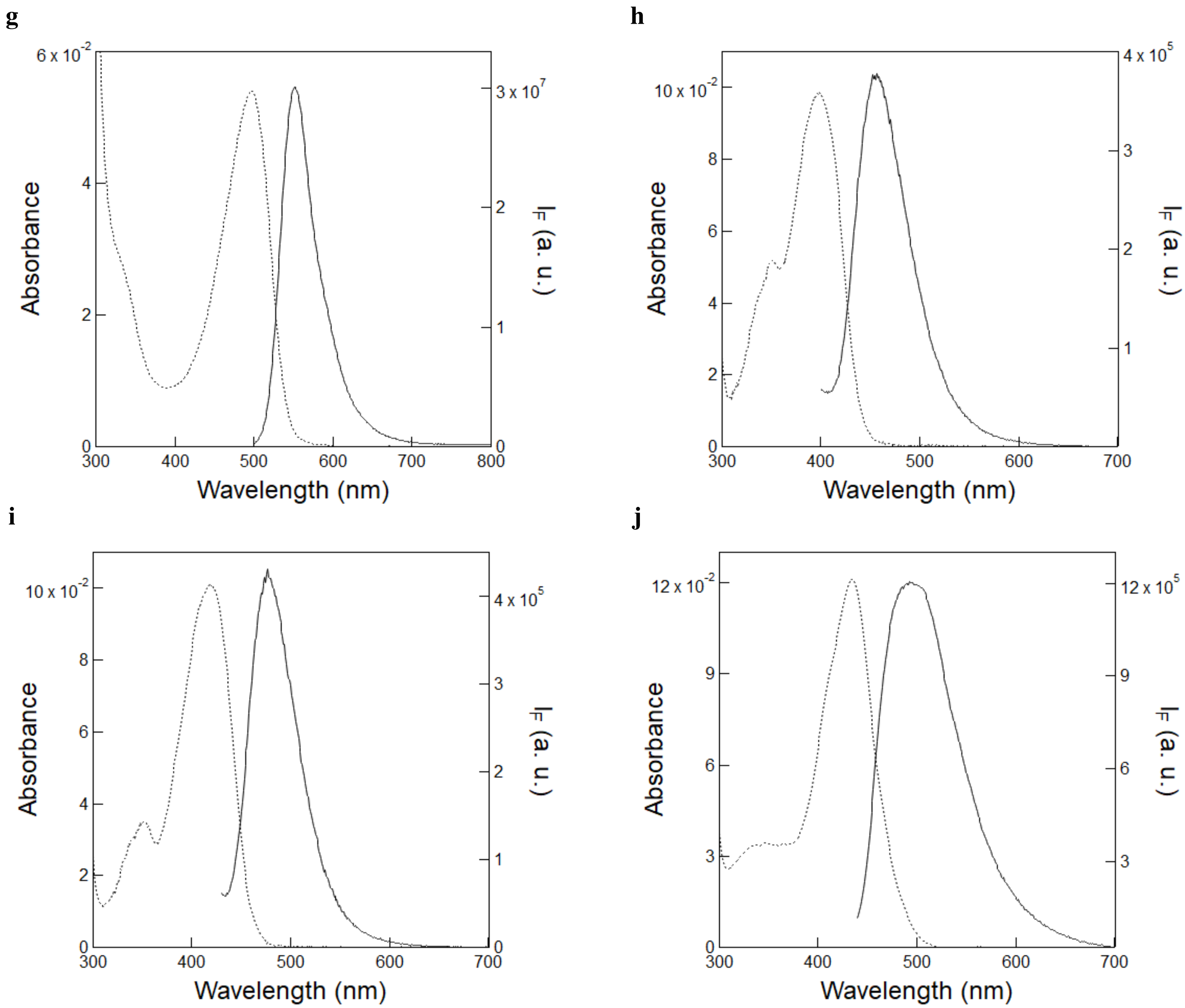

Figure S22: *UV-Vis absorption and emission spectra of the complexes between **pFAST** and the fluorogens.* The absorption (dotted line) and emission (solid line; λ_{exc} = 480 nm (**HBR25DM**), λ_{exc} = 380 nm (**HBT3CN**) and λ_{exc} = 420 nm (**HBP3CN**, **HBT3CN**)) spectra with **HBR25DM (g)**, **HBT3CN (h)**, **HBP3CN (i)**, and **HBTH3CN (j)** were recorded at 4 μM **HBR25DM**, 10 μM **HBT3CN**, **HBP3CN**, and **HBTH3CN**, in the presence of 32 μM **pFAST** in 1$\times$ pH = 7.4 PBS buffer. T = 293 K.

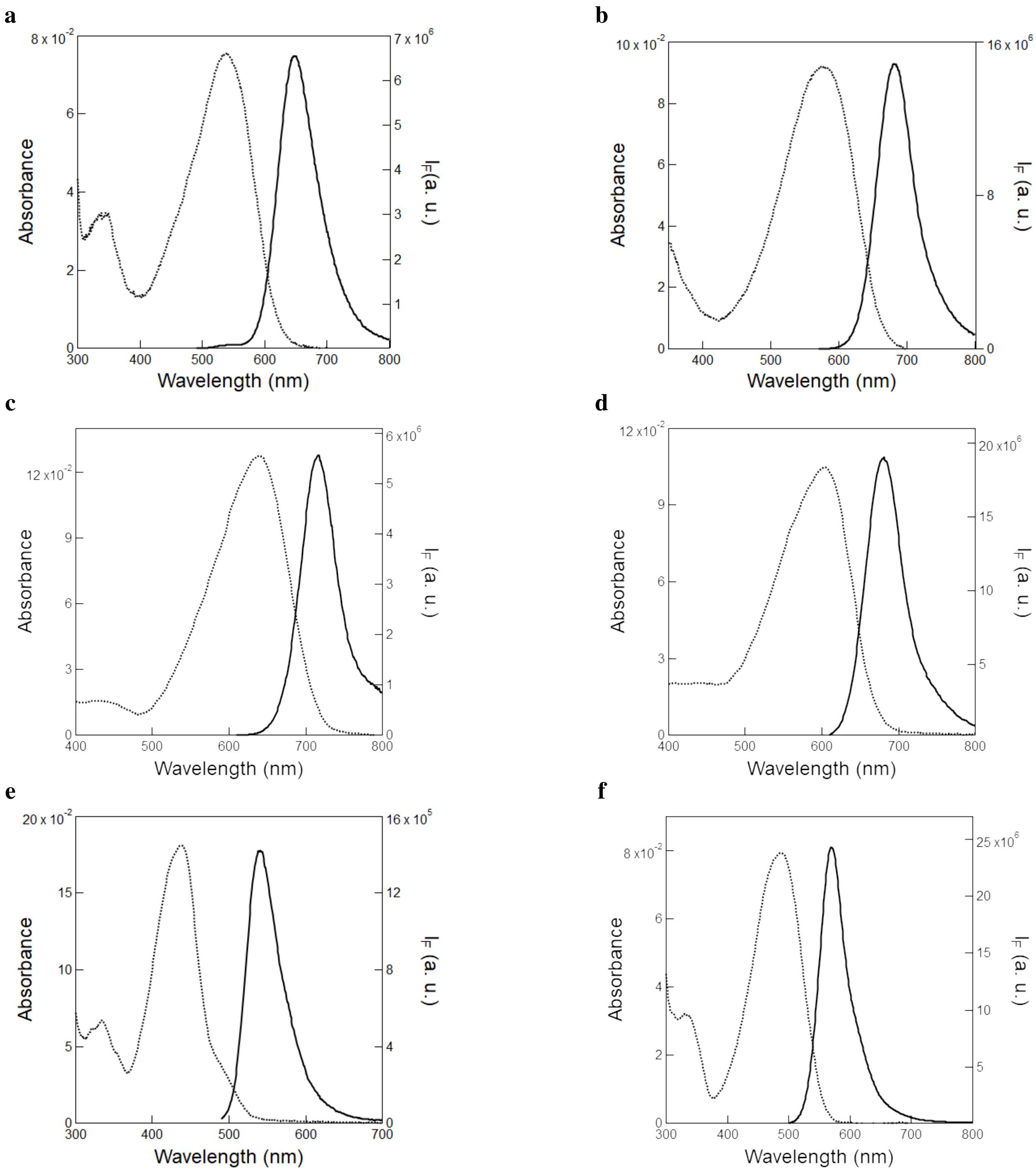

Figure S23: *UV-Vis absorption and emission spectra of the complexes between **nirFAST** and the fluorogens.* The absorption (dotted line) and emission (solid line) spectra with **HPAR3Cl (a)**, **HPAR3F5OM (b)**, **HPAR3OM (c)**, **HPAR35DOM (d)**, **HBR3Cl (e)** and **HBR3F5OM (f)** (λ_{exc} = 480 nm for **(a)**, **(e)** and **(f)**, λ_{exc} = 560 nm for **(b)** and λ_{exc} = 600 nm for **(c)** and **(d)**) were recorded at 10 μM of fluorogens in the presence of 30 μM **nirFAST** in 1$\times$ pH = 7.4 PBS buffer. T = 293 K.

g

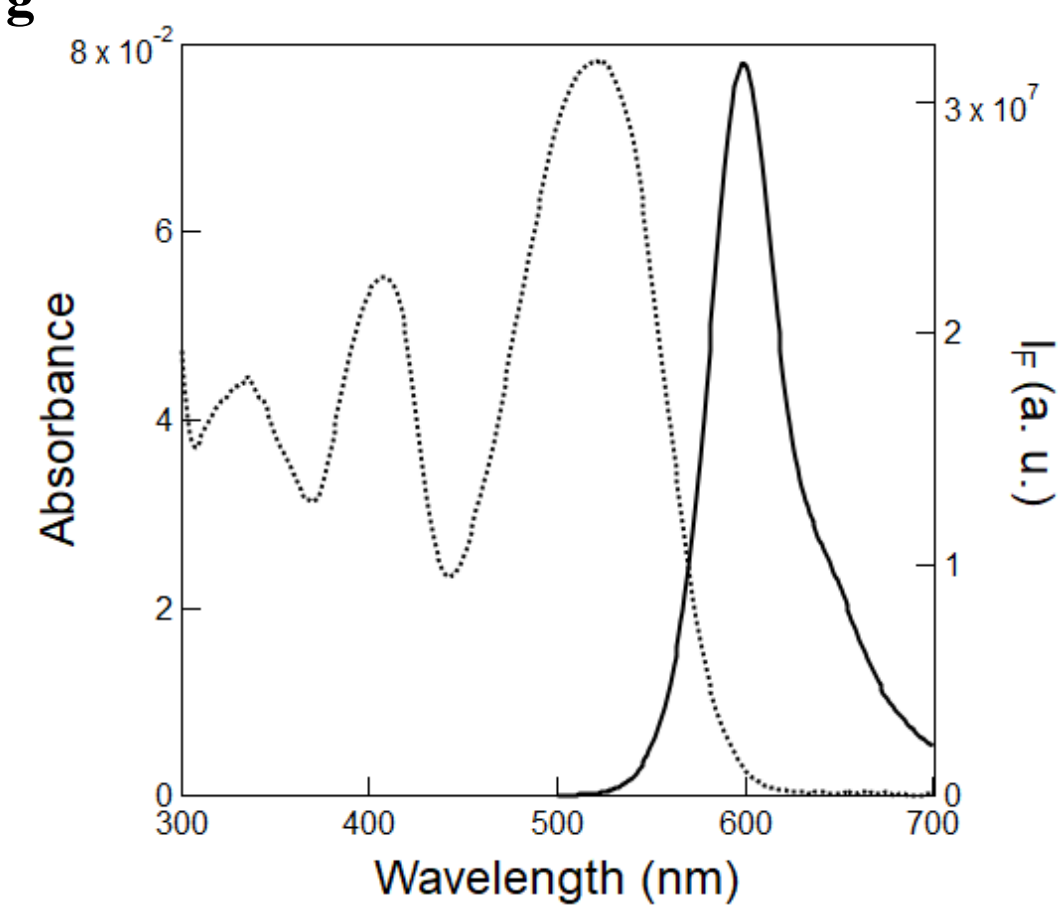

Figure S23: *UV-Vis absorption and emission spectra of the complexes between* ***nirFAST*** *and the fluorogens.* The absorption (dotted line) and emission (solid line) spectra with **HBR35DOM (g)** (λ_{exc} = 480 nm) were recorded at 10 μM of fluorogen in the presence of 30 μM **nirFAST** in 1$\times$ pH = 7.4 PBS buffer. T = 293 K.

Table S3: *Photophysical properties of the complex between **pFAST** and the (Z)-stereoisomer of the fluorogens.* Solvent: 1× pH = 7.4 PBS buffer. T = 293 K.

Fluorogens	λ_{abs}^{max} (nm)	λ_{em}^{max} (nm)	ε ($mM^{-1}cm^{-1}$)	Φ	Brightness ($M^{-1}cm^{-1}$)
**HBT3CN**	398	455	33	–	–
**HBO3M**	392	473	14	0.076	1000
**HBP3CN**	420	478	34	–	–
**HBO35DM**	411	483	6	0.23	1300
**HBT35DM**	433	499	33	0.10	3300
**HBTH3CN**	432	500	39	–	–
**HBR3CN**	454	516	44	0.02	880
**HBR35DF**	464	528	56	0.06	3360
**HBR3Cl**	471	530	41	0.08	3280
**HBR3Cl5F**	467	530	51	0.05	2550
**HBT35DOM**	449	532	60	0.27	16200
**HBR3M**	481	542	54	0.23	12400
**HBR25DM**	498	552	45	0.29	13000
**HBP35DOM**	487	554	37	0.33	12200
**HBIR3Cl**	497	557	29	0.002	50
**HBR3F5OM**	494	567	30	0.42	12600
**HBR35DOM**	520	600	44	0.35	15000
**HBIR35DOM**	562	616	40	0.10	4000

Abbreviations are as follows: λ_{abs}^{max} and λ_{em}^{max}: wavelengths of maximum absorption and emission respectively; ε: molar absorption coefficient at λ_{abs}^{max}; Φ: fluorescence quantum yield (see subsection 2.5.4 for details. Standard error: 10 %); Molecular brightness = $\Phi \times \varepsilon$. The photophysical data for **HBR3M**, **HBR25DM**, **HBR35DOM**, **HBIR35DOM**, **HBT35DM**, **HBT35DOM**, **HBO3M**, **HBO35DM** and **HBP35DOM** with **pFAST** were taken from.[4]

Table S4: *Photophysical properties of the complex between **nirFAST** and the (Z)-stereoisomer of the fluorogens.* Solvent: 1× pH = 7.4 PBS buffer. T = 293 K.

Fluorogens	λ_{abs}^{max} (nm)	λ_{em}^{max} (nm)	ϵ ($mM^{-1}cm^{-1}$)	Φ	Brightness ($M^{-1}cm^{-1}$)
**HBR3Cl**	439	540	60	0.08	4800
**HBR3F5OM**	487	570	26	0.15	4000
**HBR35DOM**	521	600	26	0.24*	6240
**HPAR3Cl**	540	650	24	0.08	1920
**HPAR3F5OM**	580	680	30	0.14	4200
**HPAR3OM**	603	681	35	0.20*	7000
**HPAR35DOM**	639	716	43	0.069*	2970

Abbreviations are as follows: λ_{abs}^{max} and λ_{em}^{max}: wavelengths of maximum absorption and emission respectively; ε: molar absorption coefficient at λ_{abs}^{max}; Φ: fluorescence quantum yield (see subsection 2.5.4 for details. Standard error: 10 %); Molecular brightness = $\Phi \times \varepsilon$. *The data was taken from.[5]

4.2 Measurement of the thermodynamic dissociation constant of the complex between pFAST or nirFAST and the thermodynamically stable state of the fluorogen (K_d)

4.2.1 Complexes with pFAST

4.2.1.1 HBR3Cl, HBR3Cl5F, HBR35DF, HBR3M, HBR25DM 2.5 mL of 10 nM pFAST solution in 1× pH = 7.4 PBS buffer in a quartz cuvette (1×1 cm^2) were subjected to irradiation at 480 nm ($I = 1.1 \times 10^{-3}$ E.m^{-2}.s^{-1}) up to the photostationary state. The cuvette content was then kept in the dark for 5–10 min to get thermal recovery of the photoisomerized fluorogen before sequentially adding aliquots of 5 μL of 100 nM fluorogen and 10 nM pFAST in 1× pH = 7.4 PBS buffer and irradiating again the resulting solution with 480 nm light to reach the photostationary state.
We then subsequently fitted the F_{tot}-dependence of the fluorescence intensity I_F at initial and final times of the investigated time window by using Eq. (S10).
Figure S24a–j displays the results for **HBR3Cl**, **HBR3Cl5F**, **HBR35DF**, **HBR3M** and **HBR25DM**. We retrieved for K_d 0.09 ± 0.01 and 0.10 ± 0.01 nM (for **HBR3Cl**), 0.12 ± 0.01 and 0.16 ± 0.01 nM(for **HBR3Cl5F**), 0.14 ± 0.01 and 0.13 ± 0.01 nM (for **HBR35DF**), 3.00 ± 0.20 and 3.30 ± 0.30 (for **HBR3M**) and 0.80 ± 0.05 and 0.90 ± 0.05 nM (for **HBR25DM**) from processing the fluorogen concentration dependence of the fluorescence signal at initial and photostationary state.[c]

4.2.1.2 HBR3F5OM, HBR35DOM, HBT35DM Upon applying the same protocol on **HBR3F5OM**, **HBR35DOM** and **HBT35DM** (Figure S25a,c,e), we did not observe any time evolution of the fluorescence signal. Hence, we reduced analysis to processing the fluorogen concentration dependence of the observed fluorescence signal and retrieved 0.70 ± 0.04 (**HBR3F5OM**), 7.70 ± 0.50 (**HBR35DOM**) and 14.00 ± 1.50 (**HBT35DM**) nM for K_d.[d]

[c] We additionally extracted the total protein concentration from data fitting: 0.7, 0.9, 1.6, 4.6 and 5.0 nM were found for **HBR3Cl**, **HBR3Cl5F**, **HBR35DF**, **HBR3M** and **HBR25DM** respectively. Such values are lower than the nominal 10 nM concentration of protein, which could be attributed to nonspecific adsorption of the protein onto the glass surface.

[d] We additionally extracted the total protein concentration from data fitting: 7.8 (**HBR3F5OM**), 1.3 (**HBR35DOM**) and 5.7 nM (**HBR3F5OM**) was found, which again suggests nonspecific adsorption of the protein to occur onto the glass surface.

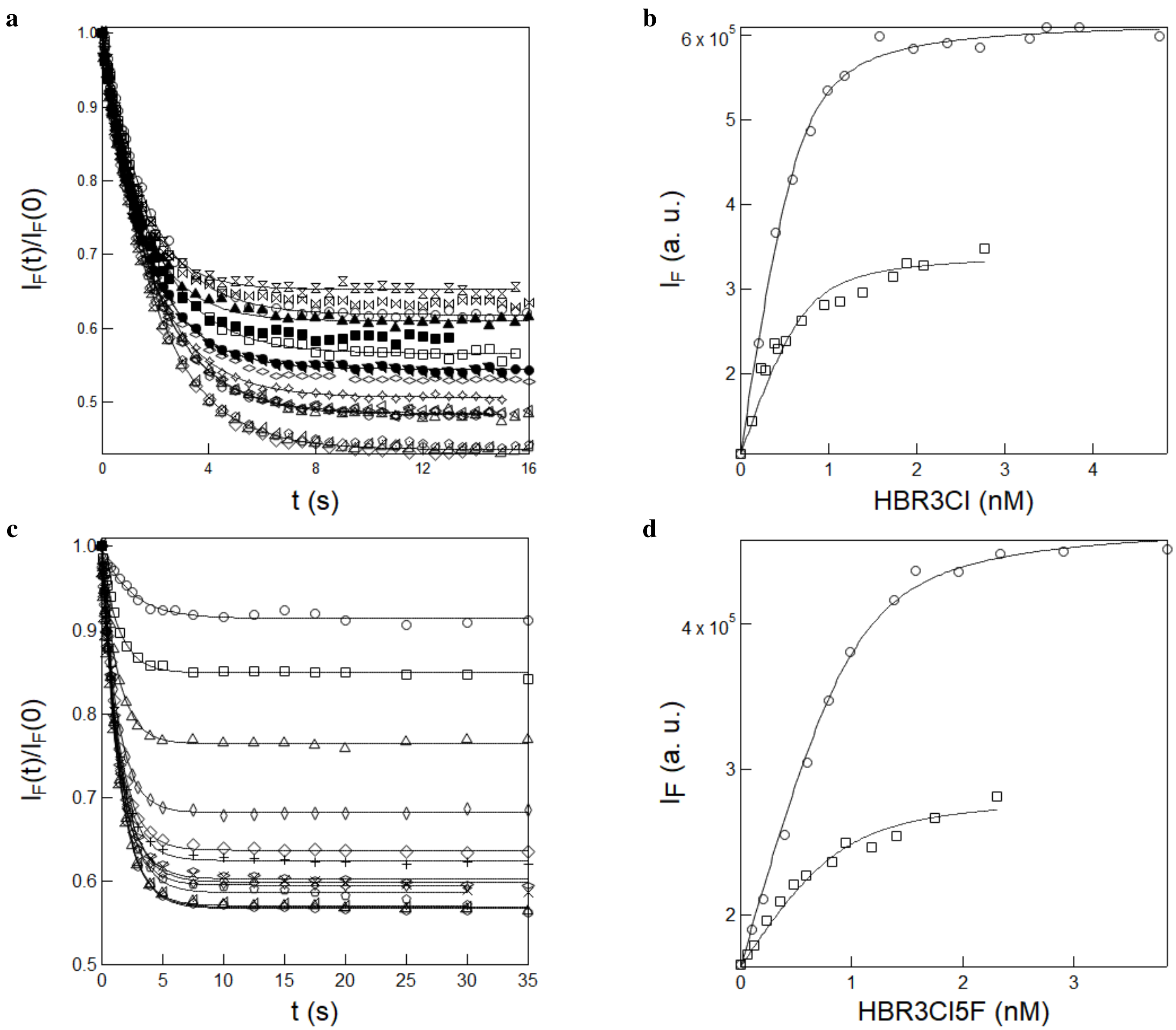

Figure S24: *Measurement of the thermodynamic dissociation constant of the complex between* ***pFAST*** *and the (Z)-stereoisomer of* ***HBR3Cl*** *and* ***HBR3Cl5F***. **a, c**: Time evolution of fluorescence emission at 530 nm from 10 nM **pFAST** solution in $1\times$ pH = 7.4 PBS buffer after successive addition of aliquots of 5 μL of 100 nM **HBR3Cl (a)** or 100 nM **HBR3Cl5F (c)** and 10 nM **pFAST** in $1\times$ pH = 7.4 PBS buffer upon constant illumination at 480 nm ($I = 1.1 \times 10^{-3}$ E.m^{-2}.s^{-1}) up to the photostationary state. Markers: experimental data; solid line: Monoexponential fit; **b, d**: Dependence of fluorescence intensity at initial (circles) and photostationary state (squares) on the concentration of **HBR3Cl (b)** and **HBR3Cl5F (d)** retrieved from **a** and **c** respectively. Markers: experimental data; solid line: Fit with Eq. (S10). $T = 293$ K.

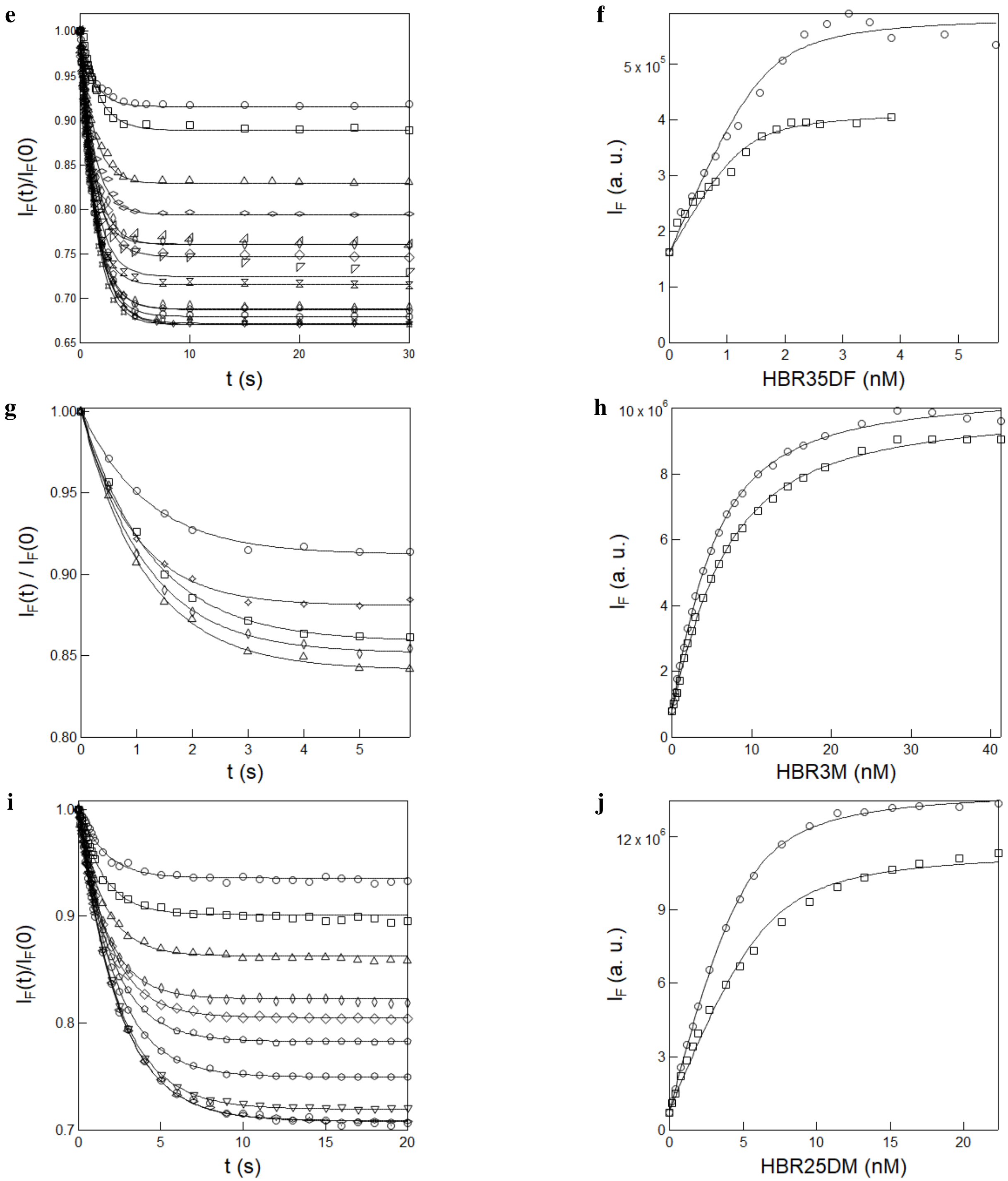

Figure S24: *Measurement of the thermodynamic dissociation constant of the complex between* ***pFAST*** *and the (Z)-stereoisomer of* ***HBR35DF****,* ***HBR3M*** *and* ***HBR25DM***. **e,g,i**: Time evolution of fluorescence emission at 530 nm from 10 nM **pFAST** solution in $1\times$ pH = 7.4 PBS buffer after successive addition of aliquots of 5 μL of 100 nM **HBR35DF**, 500 nM **HBR3M** and **HBR25DM** containing 10 nM **pFAST** in $1\times$ pH = 7.4 PBS buffer upon constant illumination at 480 nm ($I = 1.1 \times 10^{-3}$ E.m^{-2}.s^{-1}) up to the photostationary state. Markers: experimental data; solid line: Monoexponential fit; **f**: Dependence of fluorescence intensity at initial (circles) and photostationary state (squares) on the concentration of **HBR35DF (f)**, **HBR3M (h)** and **HBR25DM (j)** retrieved from **e,g,i**. Markers: experimental data; solid line: Fit with Eq. (S10). $T = 293$ K.

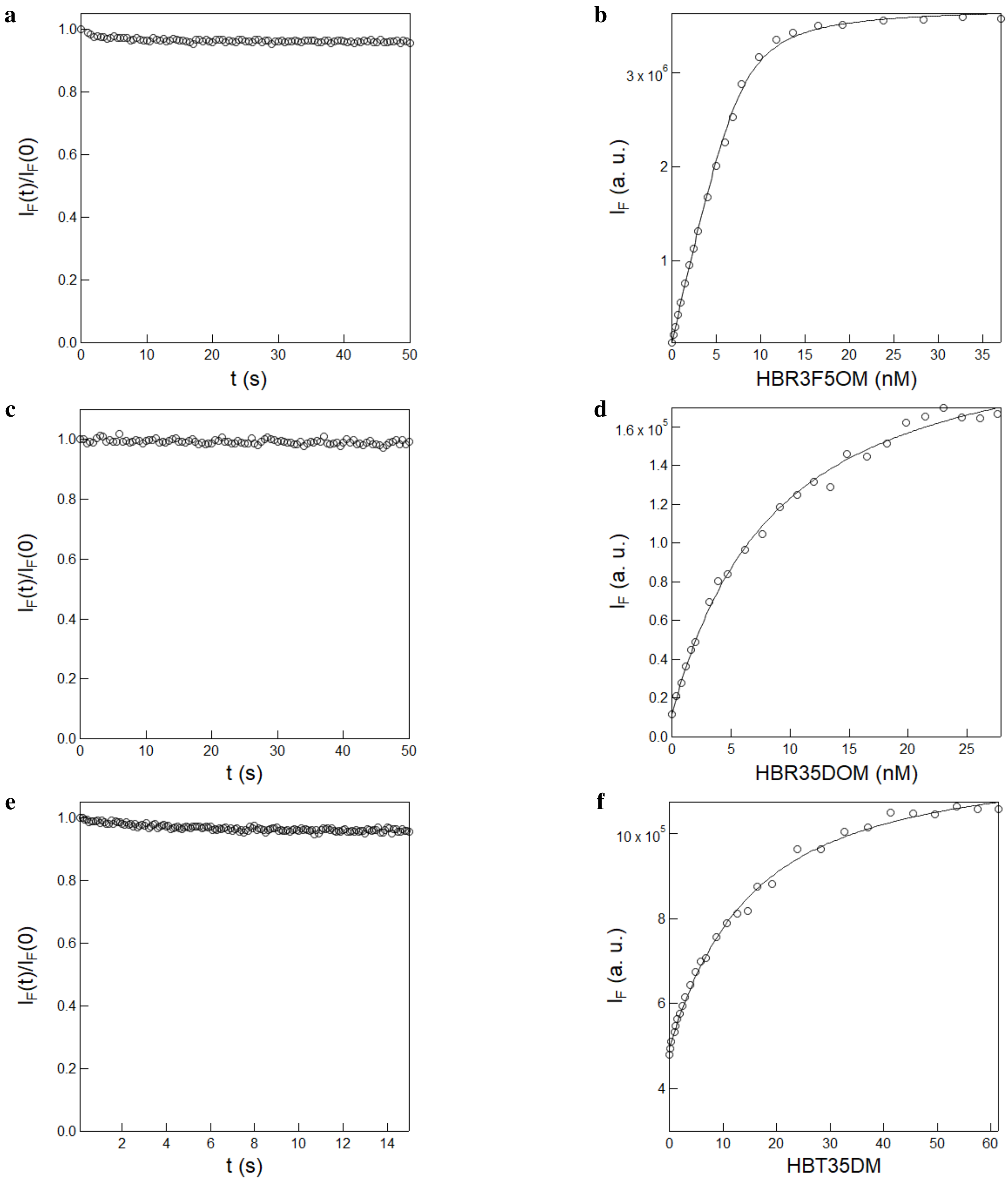

Figure S25: *Measurement of the thermodynamic dissociation constant of the complex between* ***pFAST*** *and the (Z)-stereoisomer of* ***HBR3F5OM****,* ***HBR35DOM*** *and* ***HBT35DM***. **a,c,e**: Time evolution of fluorescence emission at 570 nm (for **HBR3F5OM**), 610 nm (**HBR35DOM**) and 500 nm (for **HBT35DM**) from 10 nM **pFAST** solution in 1× pH = 7.4 PBS buffer after addition of 5 μL of 500 nM **HBR3F5OM (a)**, **HBT35DM (e)** or 200 nM **HBR35DOM (c)** and 10 nM **pFAST** in 1× pH = 7.4 PBS buffer upon constant illumination at 480 nm ($I = 1.1 \times 10^{-3}$ E.m^{-2}.s^{-1}). Markers: experimental data; **b,d,f**: Dependence of the fluorescence intensity at initial state (circles) on the concentration of **HBR3F5OM (b)**, **HBR35DOM (d)** and **HBT35DM (f)**. Markers: experimental data; solid line: Fit with Eq. (S10). $T = 293$ K.

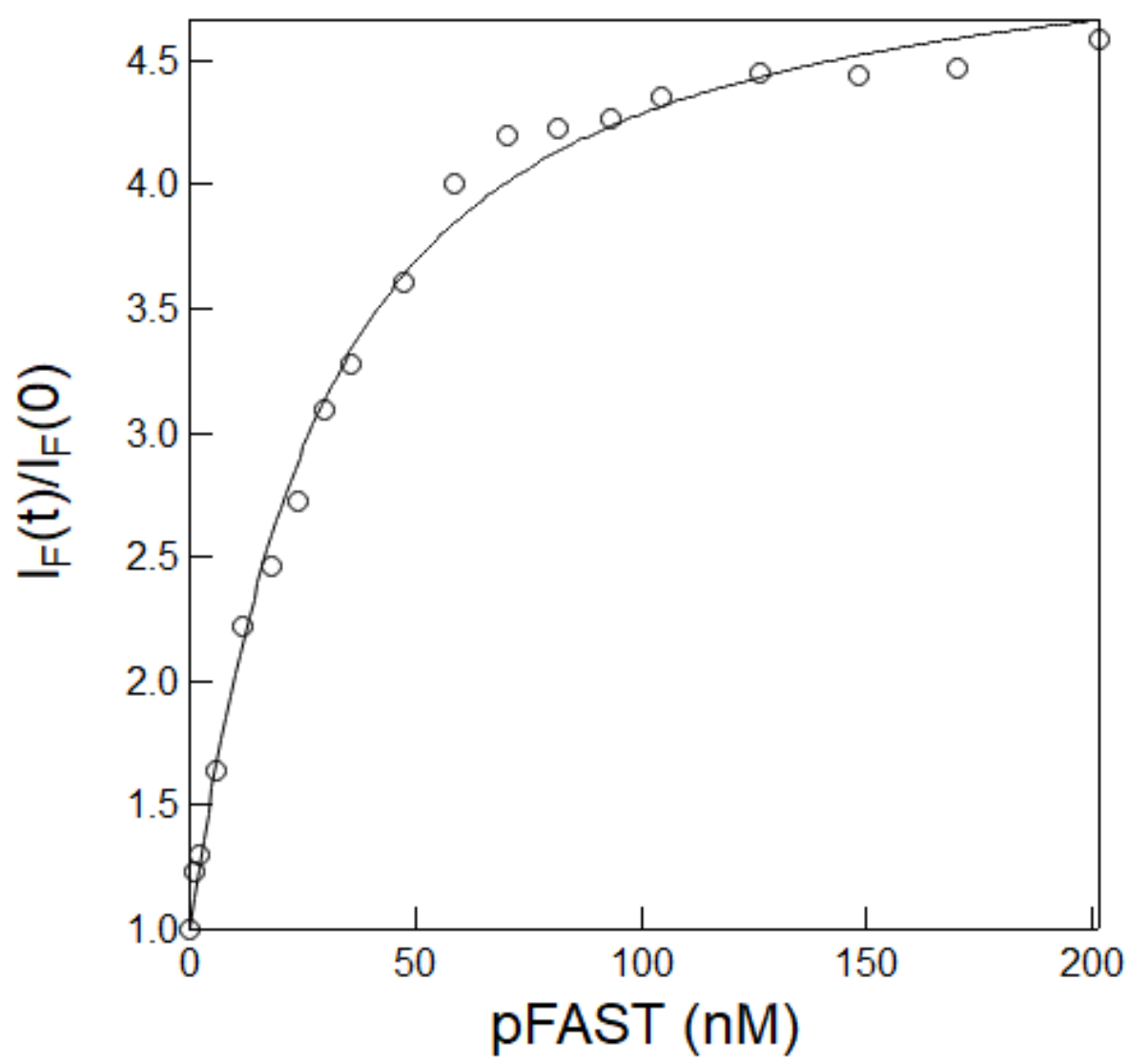

Figure S26: *Measurement of the thermodynamic dissociation constant of the complex between **pFAST** and the (Z)-stereoisomer of **HBR3CN***. Fluorogen concentration dependence of the initial fluorescence signal at 530 nm from titrating 10 nM **HBR3CN** solution in 1× pH = 7.4 PBS buffer after successive addition of aliquots of 3000 nM **pFAST** and 10 nM **HBR3CN** solution in 1× pH = 7.4 PBS buffer upon excitation using 480 nm Xe lamp. Markers: experimental data; solid line: Fit with Eq. (S10). $T = 293$ K.

4.2.1.3 HBR3CN The protocol described above has been modified for extracting K_d with **HBR3CN**. Indeed, thermal recovery was too slow for **HBR3CN**. Hence, we performed a regular titration by using the Xenon lamp of our fluorimeter for reading out fluorescence without generating any significant photoisomerization due to its low intensity (Figure S26). Hence, we processed the protein concentration-dependence of the fluorescence signal and retrieved 22.7 ± 1.50 (for **HBR3CN**) for K_d.[e]

[e]We additionally retrieved from fitting 10 nM for the total fluorogen concentration with **HBR3CN**.

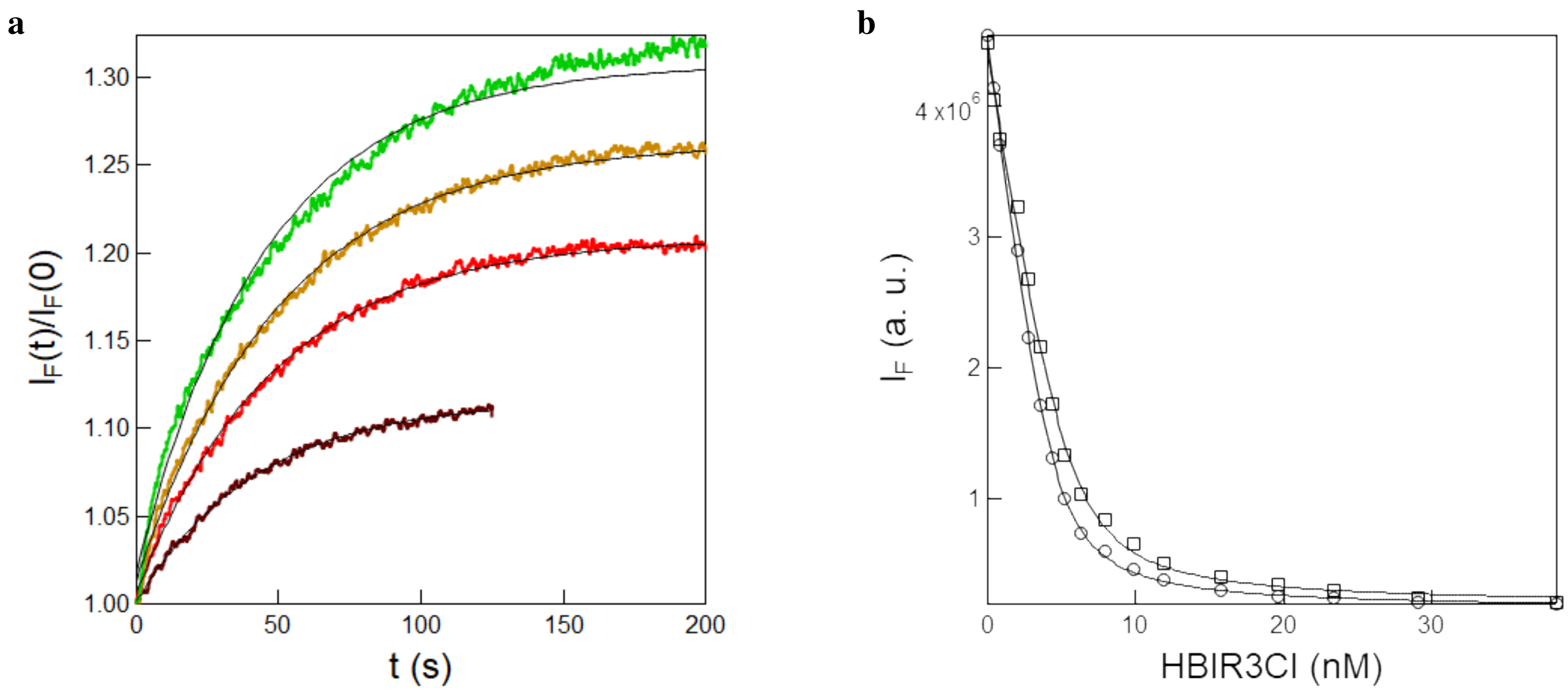

Figure S27: *Measurement of the thermodynamic dissociation constant of the complex between* ***pFAST*** *and the (Z)-stereoisomer of* ***HBIR3Cl****.* **a**: Time evolution of fluorescence emission at 610 nm from 10 nM **HBR35DOM** and **pFAST** solution in 1× pH = 7.4 PBS buffer after successive addition of aliquots of 5 μL of 1000 nM **HBIR3Cl** and 10 nM **pFAST** in 1× pH = 7.4 PBS buffer upon constant illumination at 480 nm ($I = 1.1 \times 10^{-3}$ E.m^{-2}.s^{-1}) up to the photostationary state. Markers: experimental data; solid line: Monoexponential fit; **b**: Dependence of fluorescence intensity at initial (circles) and photostationary state (squares) on the concentration of **HBIR3Cl** retrieved from **a**. Markers: experimental data; solid line: Fit with Eq.(S15). $T = 293$ K.

4.2.1.4 HBIR3Cl **HBIR3Cl** exhibits negligible fluorescence upon binding with **pFAST**. Therefore, in order to measure its affinity for **pFAST**, we designed a competition binding experiment by using the **HBR35DOM** nonphotoisomerizing fluorogen as a competitor.

Here, the titration experiments were performed by titrating 2.5 mL of 10 nM **HBR35DOM** and **pFAST** solution in 1× pH = 7.4 PBS buffer contained in a 3 mL quartz cuvette (1 cm optical path length) with 1000 nM **HBIR3Cl** and 10 nM **pFAST** solution in 1× pH = 7.4 PBS buffer.

After addition of each aliquot, the solution was subjected to irradiation at 480 nm ($I = 1.1 \times 10^{-3}$ E.m^{-2}.s^{-1}) up to the photostationary state (Figure S27a). The cuvette content was then kept in the dark for 5–10 min to get thermal recovery of the photoisomerized **HBIR3Cl** before proceeding to the next aliquot addition of **HBIR3Cl** and **pFAST** solution.

We retrieved $K_d = 0.40 \pm 0.04$ nM from processing the fluorogen concentration dependence of the fluorescence signal at both initial and photostationary state with Eq.(S15) (Figure S27b).[f]

[f]We additionally extracted the total **pFAST-HBR35DOM** concentration from data fitting: 4.5 nM was found. Such value is lower than the nominal 10 nM concentration of initial complex, which could be attributed to its nonspecific adsorption onto the glass surface.

4.2.2 Complexes with nirFAST

4.2.2.1 HPAR3Cl, HBR3F5OM 2.5 mL of 20 nM **nirFAST** solution in 1× pH = 7.4 PBS buffer in a quartz cuvette (1×1 cm^2) were subjected to irradiation at 480 nm or 640 nm ($I = 1.1 \times 10^{-3}$ $E.m^{-2}.s^{-1}$) up to the photostationary state. The cuvette content was then kept in the dark for 5–10 min to get thermal recovery of the photoisomerized fluorogen before sequentially adding aliquots of 5 μL of 500 nM fluorogen and 20 nM **nirFAST** in 1× pH = 7.4 PBS buffer and irradiating again the resulting solution with 480 nm or 640 nm light to reach the photostationary state.

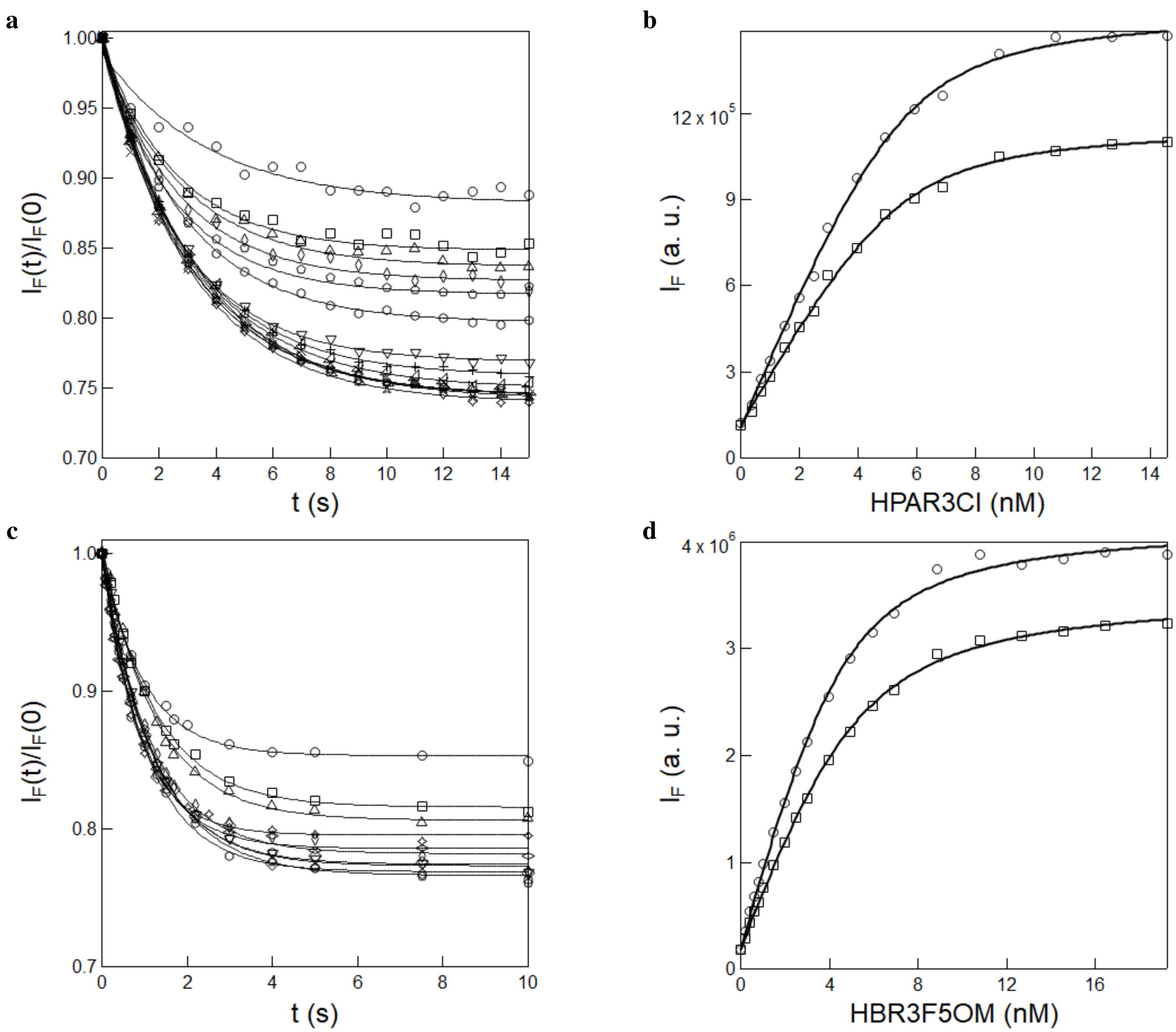

Figure S28: *Measurement of the thermodynamic dissociation constant of the complex between **nirFAST** and the (Z)-stereoisomer of **HPAR3Cl** and **HBR3F5OM**.* **a,c**: Time evolution of fluorescence emission at 650 nm (for **HPAR3Cl**) and 570 nm (for **HBR3F5OM**) from 20 nM **nirFAST** solution in 1× pH = 7.4 PBS buffer after successive addition of aliquots of 5 μL of 500 nM **HPAR3Cl (a)** and **HBR3F5OM (c)** and 20 nM **nirFAST** in 1× pH = 7.4 PBS buffer upon constant illumination at 480 nm ($I = 1.1 \times 10^{-3}$ $E.m^{-2}.s^{-1}$) up to the photostationary state. Markers: experimental data; solid line: Monoexponential fit; **b,d**: Dependence of fluorescence intensity at initial (circles) and photostationary state (squares) on the concentration of **HPAR3Cl (b)** and **HBR3F5OM (d)**. Markers: experimental data; solid line: Fit with Eq. (S10). $T = 293$ K.

Figure S28 display the results for **HPAR3Cl** and **HBR3F5OM**. We retrieved for K_d 0.52 ± 0.07 and 0.54 ± 0.09 nM for **HPAR3Cl** and 0.90 ± 0.07 and 1.10 ± 0.09 nM (for **HBR3F5OM**) from processing the fluorogen concentration dependence of the fluorescence signal at initial and photostationary state.[g]

4.2.2.2 HBR3Cl, HBR35DOM, HPAR3F5OM, HPAR3OM, HPAR35DOM Upon applying the same protocol on **HPAR3F5OM**, **HBR35DOM**, **HBR3Cl**, **HPAR3OM**, and **HPAR35DOM** (Figure S29a,c,e.g), we did not observe any time evolution of the fluorescence signal. Hence, we reduced analysis to processing the fluorogen concentration dependence of the observed fluorescence signal and retrieved 0.70 ± 0.03 (**HPAR3F5OM**), 6.70 ± 0.40 (**HBR35DOM**), 0.14 ± 0.03 (**HBR3Cl**), 0.70 ± 0.06 (**HPAR3OM**), and 1.6 ± 0.20 (**HPAR35DOM**) nM for K_d.[h]

4.2.2.3 HBIR3Cl **HBIR3Cl** exhibits negligible fluorescence upon binding with **nirFAST**. Therefore, we applied the protocol displayed in Figure S27 in order to measure its affinity for **nirFAST** by using the **HBR35DOM** nonphotoisomerizing fluorogen as a competitor.
We retrieved $K_d = 1.50 \pm 0.30$ nM from processing the fluorogen concentration dependence of the fluorescence signal at both initial and photostationary state with Eq.(S15) (Figure S30b).[i]

[g] We additionally extracted the total protein concentration from data fitting: 5.4 and 4.7 nM was found for **HPAR3Cl** and **HBR3F5OM** respectively. Such values are lower than the nominal 20 nM concentration of protein, which could be attributed to nonspecific adsorption of the protein onto the glass surface.

[h] We additionally extracted the total protein concentration from data fitting: 10, 3.5, 6, 1.8 and 2.1 nM were found for **HPAR3F5OM**, **HBR35DOM**, **HBR3Cl**, **HPAR3OM**, and **HPAR35DOM** respectively, which again suggests nonspecific adsorption of the protein to occur onto the glass surface.

[i] We additionally extracted the total **nirFAST-HBR35DOM** concentration from data fitting: 6.0 nM was found. Such value is lower than the nominal 10 nM concentration of initial complex, which could be attributed to its nonspecific adsorption onto the glass surface.

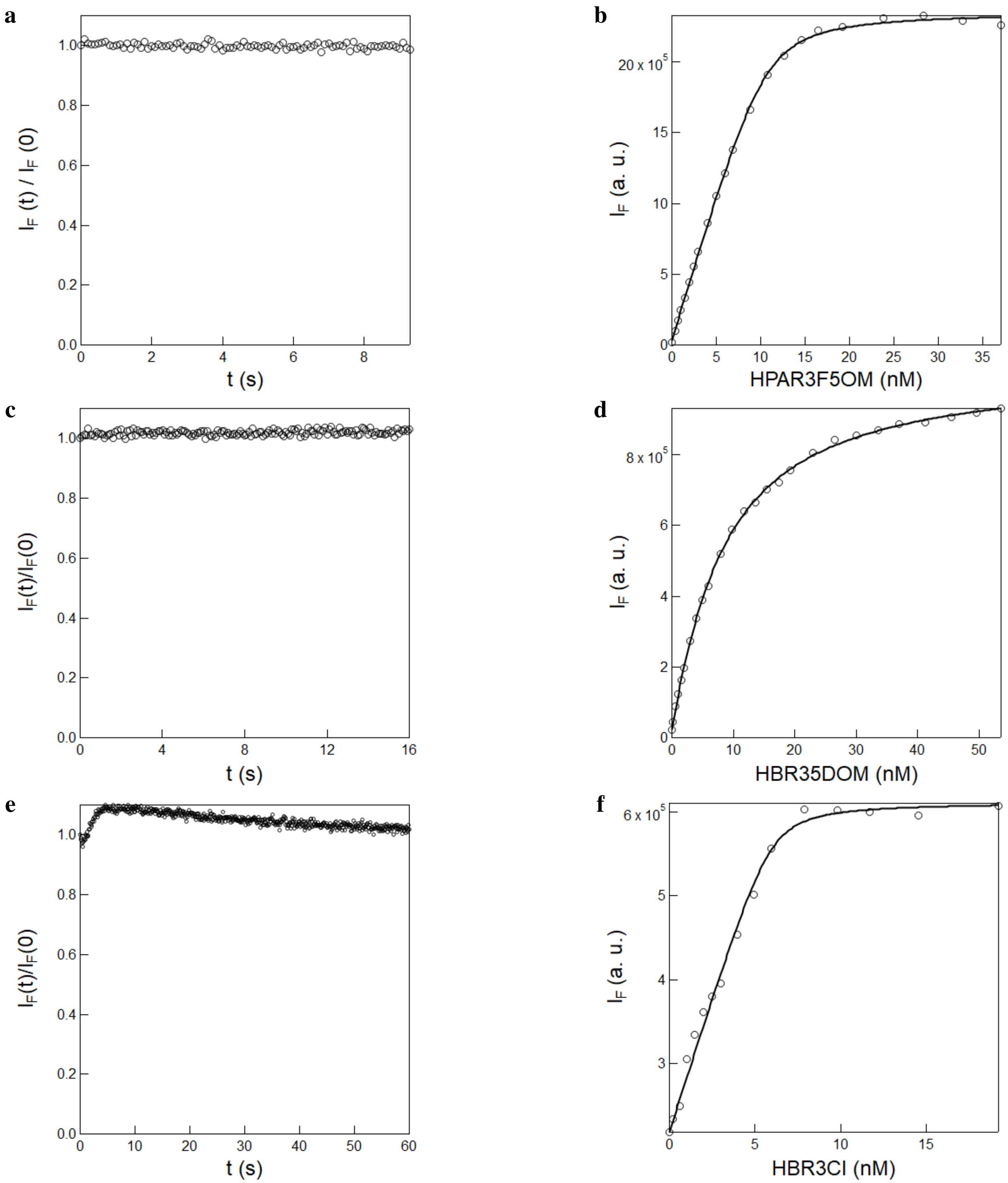

Figure S29: *Measurement of the thermodynamic dissociation constant of the complex between* ***nirFAST*** *and the (Z)-stereoisomer of* ***HPAR3F5OM****,* ***HBR35DOM****, and* ***HBR3Cl****.* **a, c, e**: Time evolution of fluorescence emission at 680 (**HPAR3F5OM**), 600 (**HBR35DOM**) and 540 (**HBR3Cl**) nm from 20 nM **nirFAST** solution in 1× pH = 7.4 PBS buffer after addition of aliquots of 100 μL of 500 nM **HPAR3F5OM (a)**, **HBR35DOM (c)**, or **HBR3Cl (e)** and 20 nM **nirFAST** in 1× pH = 7.4 PBS buffer upon constant illumination at 480 nm ($I = 1.1 \times 10^{-3}$ E.m^{-2}.s^{-1}). Markers: experimental data; **b, d**: Dependence of fluorescence intensity at initial state (circles) on the concentration of **HPAR3F5OM (b)**, **HBR35DOM (d)**, and **HBR3Cl (f)**. Markers: experimental data; solid line: Fit with Eq. (S10). $T = 293$ K.

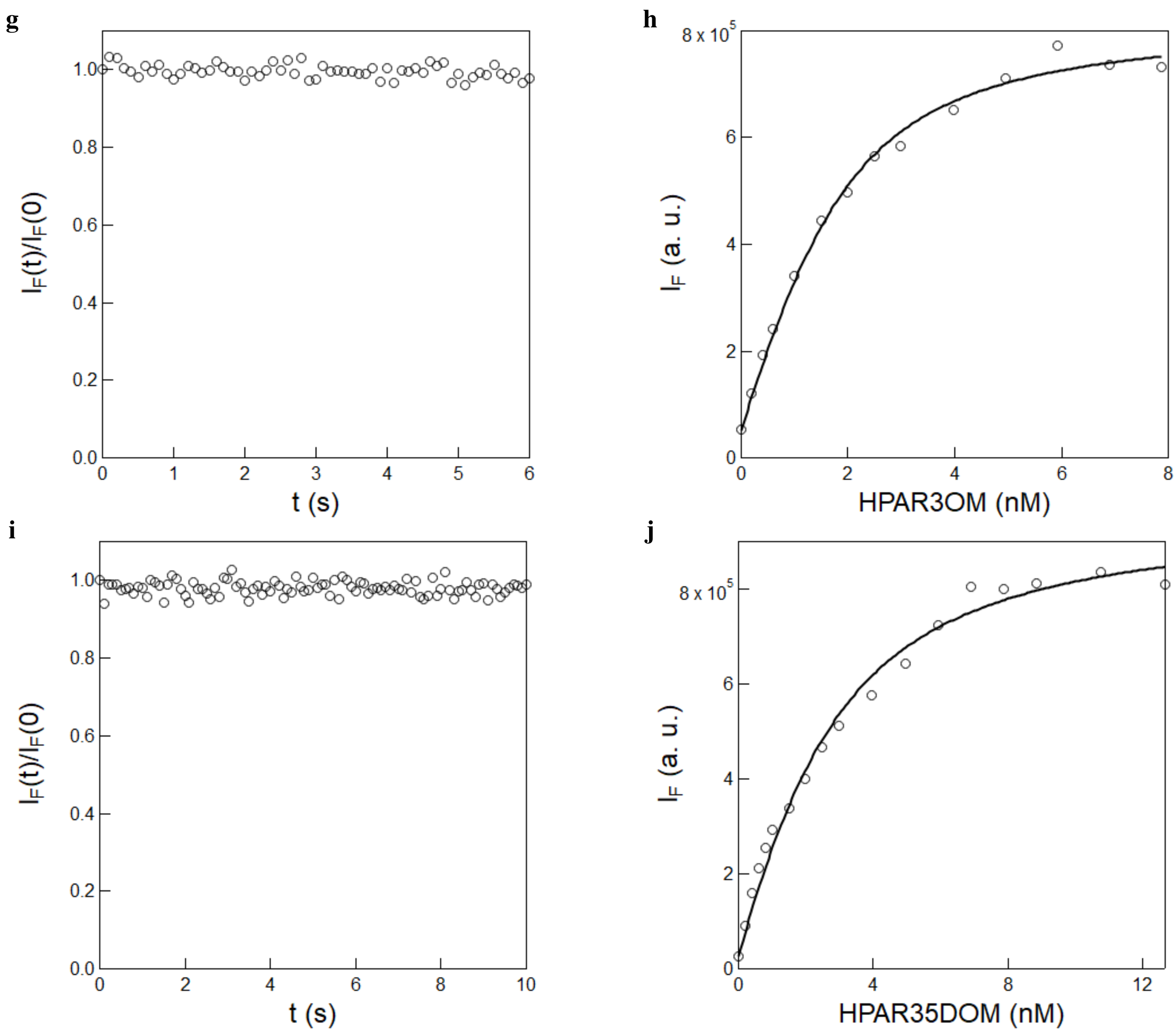

Figure S29: *Measurement of the thermodynamic dissociation constant of the complex between **nirFAST** and the (Z)-stereoisomer of **HPAR3OM** and **HPAR35DOM***. **g,i**: Time evolution of fluorescence emission at 680 nm (**HPAR3OM**) and 715 nm (**HPAR35DOM**) from 20 nM **nirFAST** solution in 1× pH = 7.4 PBS buffer after addition of aliquots of 100 μL of 500 nM **HPAR3OM (g)**, **HPAR35DOM (i)** and 20 nM **nirFAST** in 1× pH = 7.4 PBS buffer upon constant illumination at 640 nm ($I = 1.1 \times 10^{-4}$ E.m^{-2}.s^{-1}). Markers: experimental data; **h,j**: Dependence of fluorescence intensity at initial state (circles) on the concentration of **HPAR3OM (h)** and **HPAR35DOM (j)**. Markers: experimental data; solid line: Fit with Eq. (S10). $T = 293$ K.

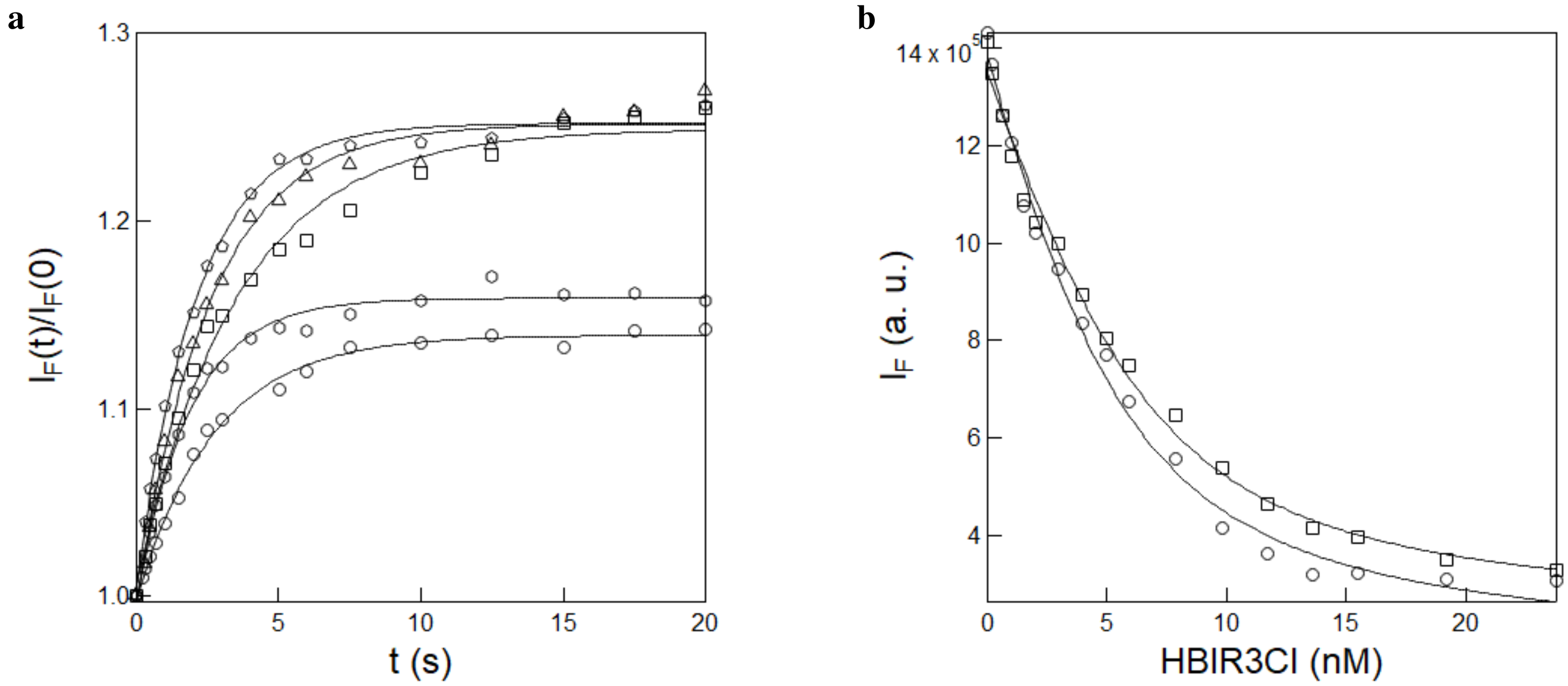

Figure S30: *Measurement of the thermodynamic dissociation constant of the complex between **nirFAST** and the (Z)-stereoisomer of **HBIR3Cl**.* **a**: Time evolution of fluorescence emission at 610 nm from 10 nM **HBR35DOM** and **nirFAST** solution in 1× pH = 7.4 PBS buffer after successive addition of aliquots of 5 μL of 1000 nM **HBIR3Cl** and 10 nM **nirFAST** in 1× pH = 7.4 PBS buffer upon constant illumination at 480 nm ($I = 1.1 \times 10^{-3}$ E.m^{-2}.s^{-1}) up to the photostationary state. Markers: experimental data; solid line: Monoexponential fit; **b**: Dependence of fluorescence intensity at initial (circles) and photostationary state (squares) on the concentration of **HBIR3Cl** retrieved from **a**. Markers: experimental data; solid line: Fit with Eq.(S15). $T = 293$ K.

Table S5: *Thermodynamic properties of the complex between* ***pFAST*** *and the (Z)-stereoisomer of the fluorogens.* Solvent: 1× pH = 7.4 PBS buffer. T = 293 K.

Fluorogens	K_d (nM)
**HBR3Cl**	0.10 ± 0.01
**HBR3Cl5F**	0.14 ± 0.01
**HBR35DF**	0.14 ± 0.01
**HBIR3Cl**	0.40 ± 0.04
**HBR3F5OM**	0.70 ± 0.04
**HBR25DM**	0.80 ± 0.05
**HBR3M**	3.0 ± 0.2
**HBR35DOM**	7.7 ± 0.5
**HBT35DM**	14.0 ± 1.5
**HBR3CN**	22.7 ± 1.5

Table S6: *Thermodynamic properties of the complex between* ***nirFAST*** *and the (Z)-stereoisomer of the fluorogens.* Solvent: 1× pH = 7.4 PBS buffer. T = 293 K.

Fluorogens	K_d (nM)
**HBR3Cl**	0.14 ± 0.03
**HPAR3Cl**	0.70 ± 0.04
**HPAR3F5OM**	0.70 ± 0.03
**HPAR3OM**	0.70 ± 0.06
**HBR3F5OM**	0.90 ± 0.07
**HBIR3Cl**	1.5 ± 0.3
**HPAR35DOM**	1.6 ± 0.2
**HBR35DOM**	6.7 ± 0.4

Abbreviation is as follows: K_d designates the thermodynamic dissociation constant of the complex between **FAST** and the (*Z*)-stereoisomer of the fluorogens.

4.3 Evaluation of the photoswitching robustness of pFAST-HBR3Cl

To address the photoswitching robustness of the **FAST**-Fluorogen systems, we investigated photoswitching of **pFAST-HBR3Cl** in two different buffers at the same pH = 7.4. In 10 mM phosphate + 140 mM NaCl buffer, the effective cross section of photoisomerization at 480 nm was 1670 ± 83 E·m^{-2}·s^{-1} and the characteristic time of thermal return was 4500 ± 441 s.[18] In 10 mM HEPES without NaCl, the effective cross section of photoisomerization was found to be 1630 ± 21 E·m^{-2}·s^{-1} and the characteristic time 5050 ± 457 s (Figure S31). The fair agreement between both the effective cross section of photoisomerization at 480 nm and characteristic time of thermal return at 293 K suggests that photoswitching associated to disruption of the **FAST-Fluorogen** complex is a robust phenomenon.

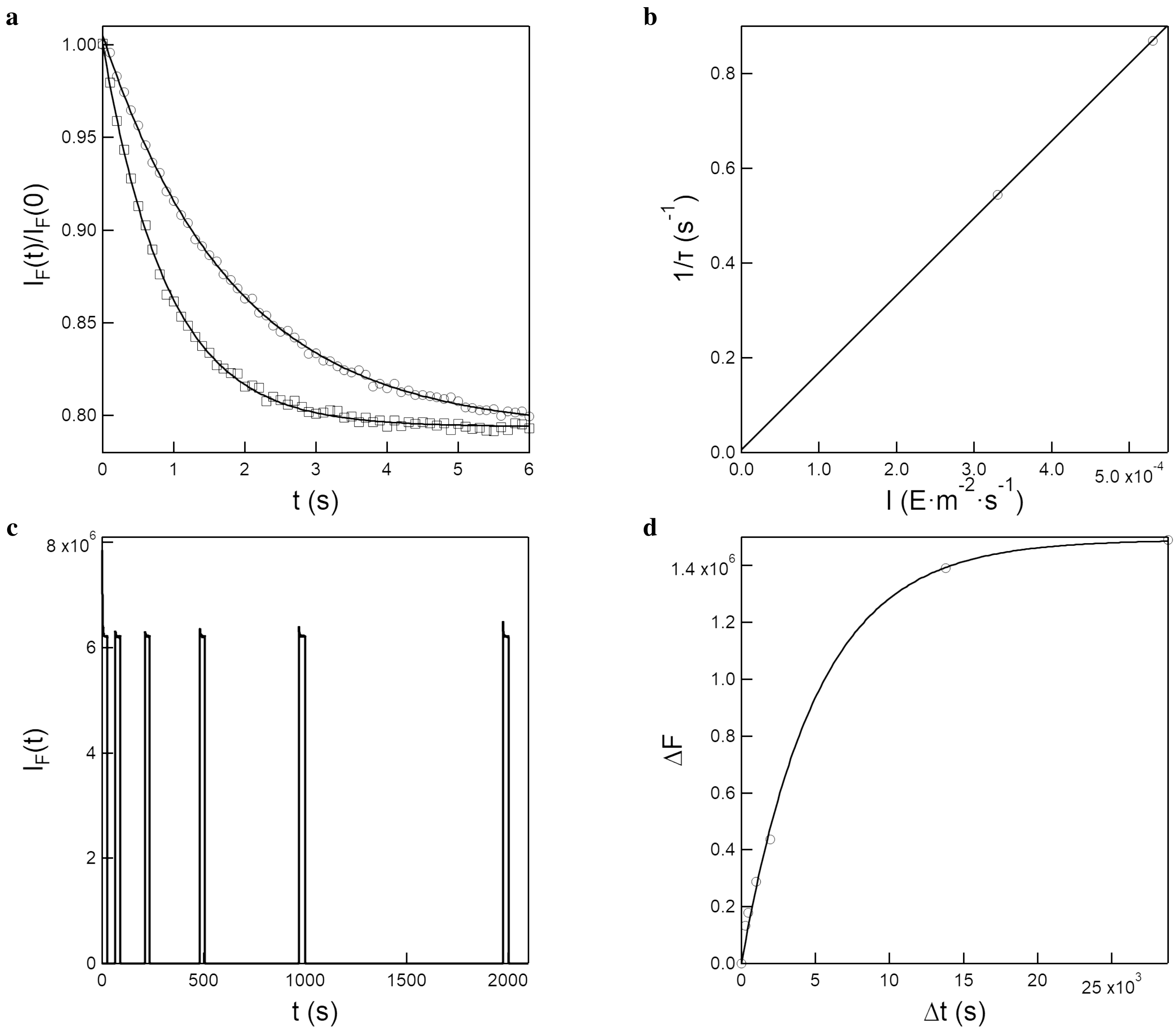

Figure S31: *Measurement of the effective cross section of photoisomerization and rate of thermal recovery of* ***pFAST-HBR3Cl in HEPES buffer***. **a**: Time evolution of the normalized fluorescence emission at 530 nm from 5 μM **HBR3Cl** and 100 μM **pFAST** in 10 mM HEPES pH = 7.4 buffer in a 54 μL cuvette (3 mm optical pathlength) upon irradiation at 480 nm at light intensities 3.3 (circles) and 5.3 (squares) $\times 10^{-4}$ E·m^{-2}·s^{-1}. Markers: experimental data; solid lines: Monoexponential fit delivering τ 1.84 and 1.15 (s). **b**: Extraction of the effective cross section of photoisomerization from the τ values retrieved in **a**. Markers: experimental data; solid line: linear fit with Eq.(S52). **c**: Time evolution of the fluorescence emission at 530 nm of the same solution exposed to a sequence of 480 nm light pulses at 3.3 $\times 10^{-4}$ E·m^{-2}·s^{-1} to reach the photostationary state while varying the delay Δt of recovery in the dark between the pulses (not shown: delays longer than 1 hour). **d**: Recovery of the fluorescence signal against the delay time retrieved in **c**. Markers: Experimental data; solid line: Monoexponential fit with Eq. (S51). $T = 293$ K.

4.4 Reference trajectory associated to replacing HBT35DM by HBR35DOM in pFAST in the CIE 1931 color space

To further appreciate the hue change generated from illuminating **pFAST** in the presence of a photoisomerizable fluorogen and a second fluorogen giving a **pFAST** complex of different color, we performed a titration experiment by recording the fluorescence emission spectrum from 700 nM **HBT35DM** and 100 nM **pFAST** in 1× pH = 7.4 PBS buffer upon adding aliquots of 10 μM **HBR35DOM** and 100 nM **pFAST** solution in the same solvent (Figure S32a). As anticipated, the fluorescence emission from the **pFAST:HBT35DM** complex is replaced by the one of the **pFAST:HBR35DOM** complex. As displayed in Figure S32b, the associated trajectory of the fluorescence emission hue in the CIE 1931 color space is linear and it essentially visits the whole distance between the two extrema associated with the **pFAST:HBT35DM** and **pFAST:HBR35DOM** complexes respectively.

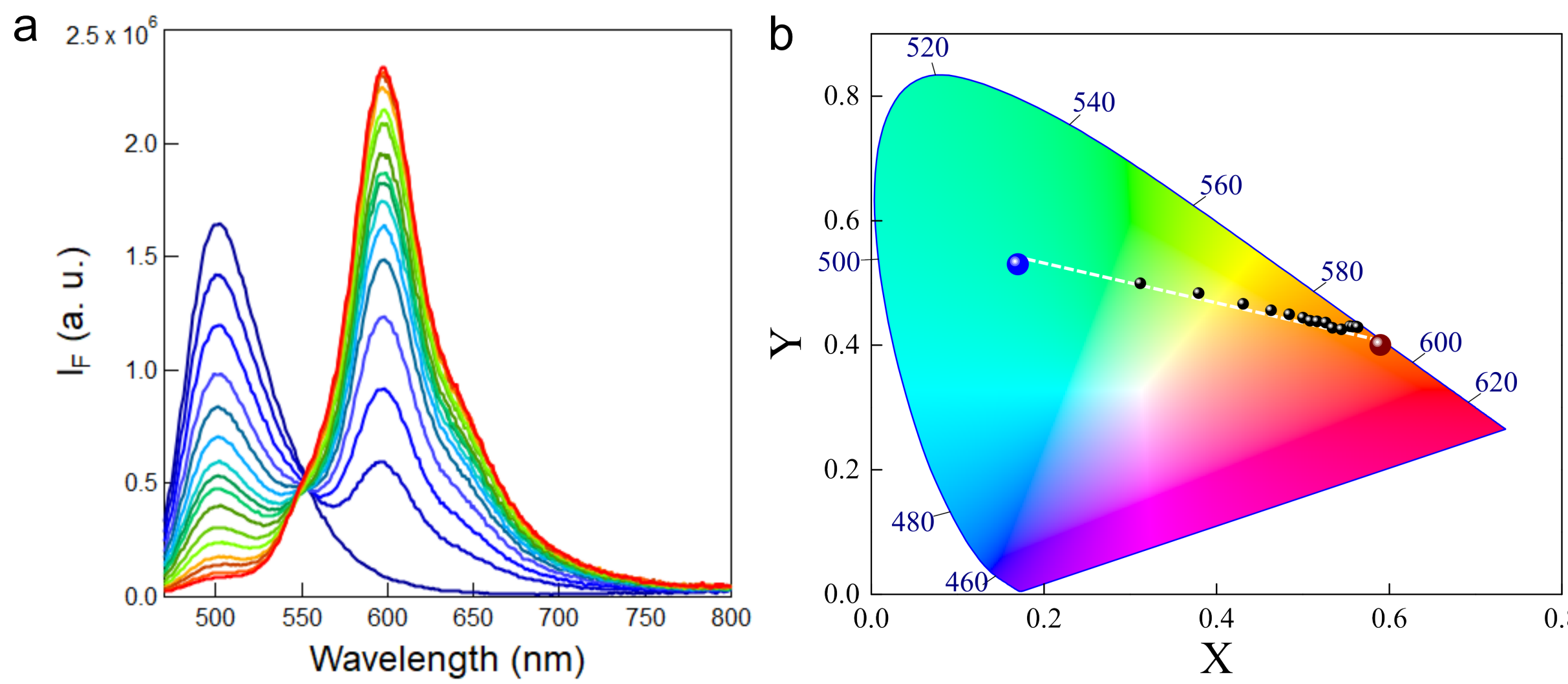

Figure S32: *Hue change originating from replacing a fluorogen by another one in a **pFAST** complex.* **a:** Evolution of the fluorescence emission spectra from 700 nM **HBT35DM** and 100 nM **pFAST** in 1× pH = 7.4 PBS buffer upon successive addition of aliquots of 10 μM **HBR35DOM** and 100 nM **pFAST** in 1× pH = 7.4 PBS buffer; **b:** Associated trajectory of the fluorescence emission hue in the CIE 1931 color space. The coordinates of the fluorescence emission from the pure complexes **pFAST:HBT35DM** and **pFAST:HBR35DOM** are displayed as blue and dark red circles respectively whereas the coordinates of the intermediate spectra obtained during the titration and aligned along a linear trajectory (dashed line) are displayed as black circles. $T = 298$ K.

5 Confocal microscopy experiments

5.1 Experiments involving one fluorogen

5.1.1 Labeling of live Hela cells

5.1.1.1 At the nucleus

Photoejection of HBR25DM As displayed in Figure S33, the fluorescence signal at the labeled nucleus monoexponentially decreased by up to 80 % upon repetitive scans in the presence of 0.1 μM of **HBR25DM** fluorogen with **pFAST**. Photoejection reversibility was evidenced by essentially recovering the initial fluorescence level upon introducing a sufficient delay (typically 120 s) before applying a new series of repetitive scans. Eventually, the fluorescence drop originating from photoejection could be abolished by increasing the fluorogen concentration above 1 μM, rejoining well established experimental conditions for **FAST** labeling.

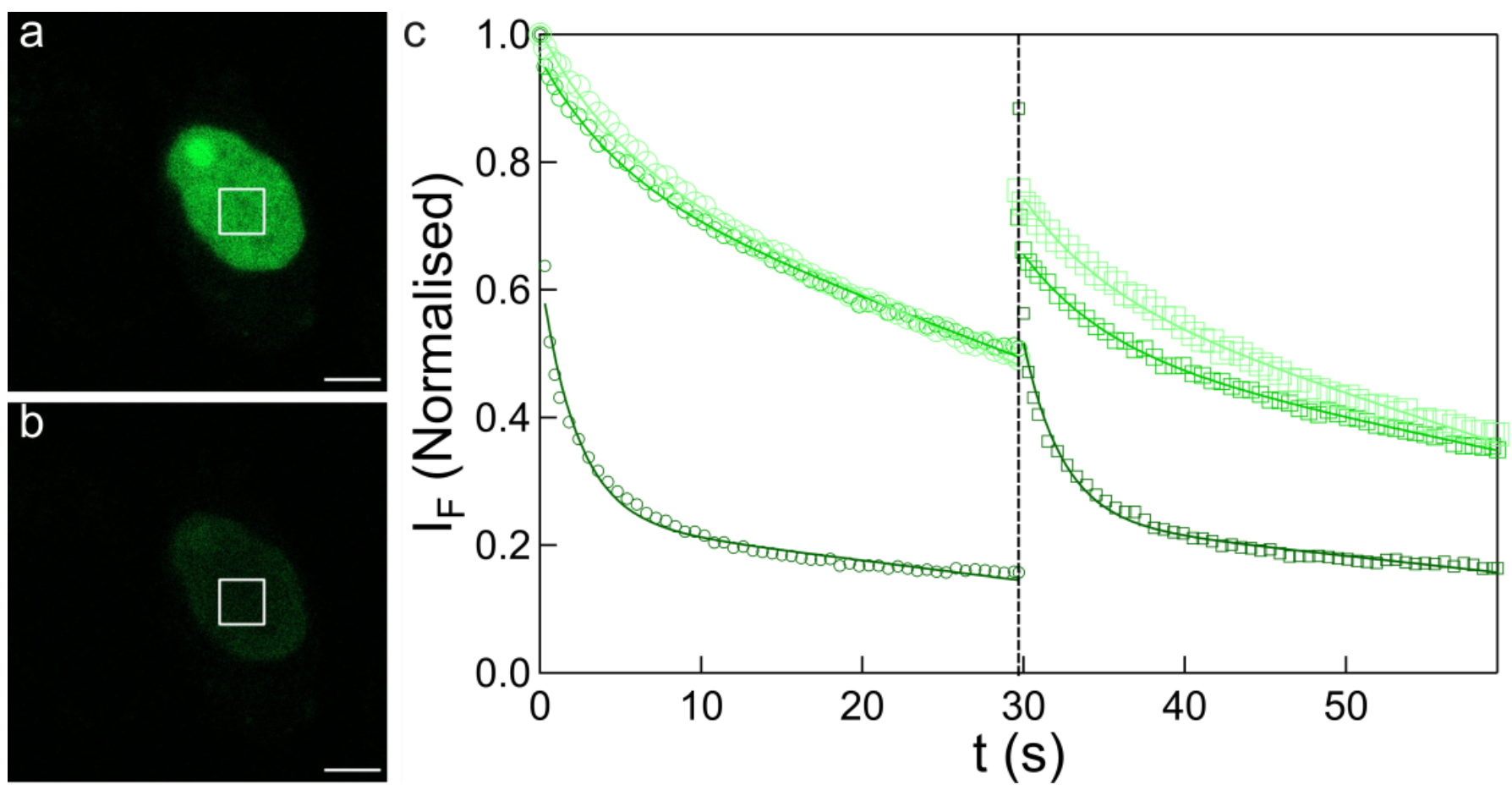

Figure S33: ***FAST** to generate a palette of negative ncRSFPs*. 488 nm light-induced fluorescence emission from **HBR25DM** (**a-c**) in **H2B-pFAST**-labeled living HeLa cells observed in confocal microscopy in the presence of the fluorogen in DMEM pH=7.4. Photoejection is evidenced at 0.1 μM concentration from the contrast at the nuclei between the initial (**a**) and photosteady state (**b**) images, its reversibility by the similar loss of fluorescence normalized by its initial value after two consecutive series of frame acquisition (circles and squares markers after the 1st and 2nd series respectively) in the region of interest at nuclei, and its concentration-dependence by the distinct decays observed at 0.1 (small green markers), 1 (medium green markers), or 10 (large green markers) μM fluorogen concentration (**c**). The vertical dashed line represents a 120 s thermal recovery period (not included in the time axis for compactness). Green lines: exponential fits of the observed fluorescence drops with Eq.(S33) by further adding a slow linear drift to account for photobleaching. Scale bar: 5 μm. $T = 298$ K. See Table S9 for the conditions of image acquisition.

Photoejection of HPAR3Cl As displayed in Figure S34, the fluorescence signal at the labeled nucleus monoexponentially decreased by up to 80 % upon repetitive scans in the presence of 0.1 μM of **HPAR3Cl** fluorogen with **nirFAST**. Photoejection reversibility was evidenced by essentially recovering the initial fluorescence level upon introducing a sufficient delay (typically 120 s) before applying a new series of repetitive scans.

5.1.1.2 At mitochondria

Photoejection in pFAST-HBR3F5OM To complement the experiments performed on live HeLa cells expressing **H2B-pFAST** in the nucleus, we examined whether photoejection could be observed in live HeLa cells expressing **Mito-pFAST** at mitochondria. Hence, such HeLa cells were conditioned with **HBR3F5OM** and imaged in confocal microscopy under 488 nm excitation. As displayed in Figure S35, those cells exhibit similar photoejection to the ones that express **H2B-pFAST**.

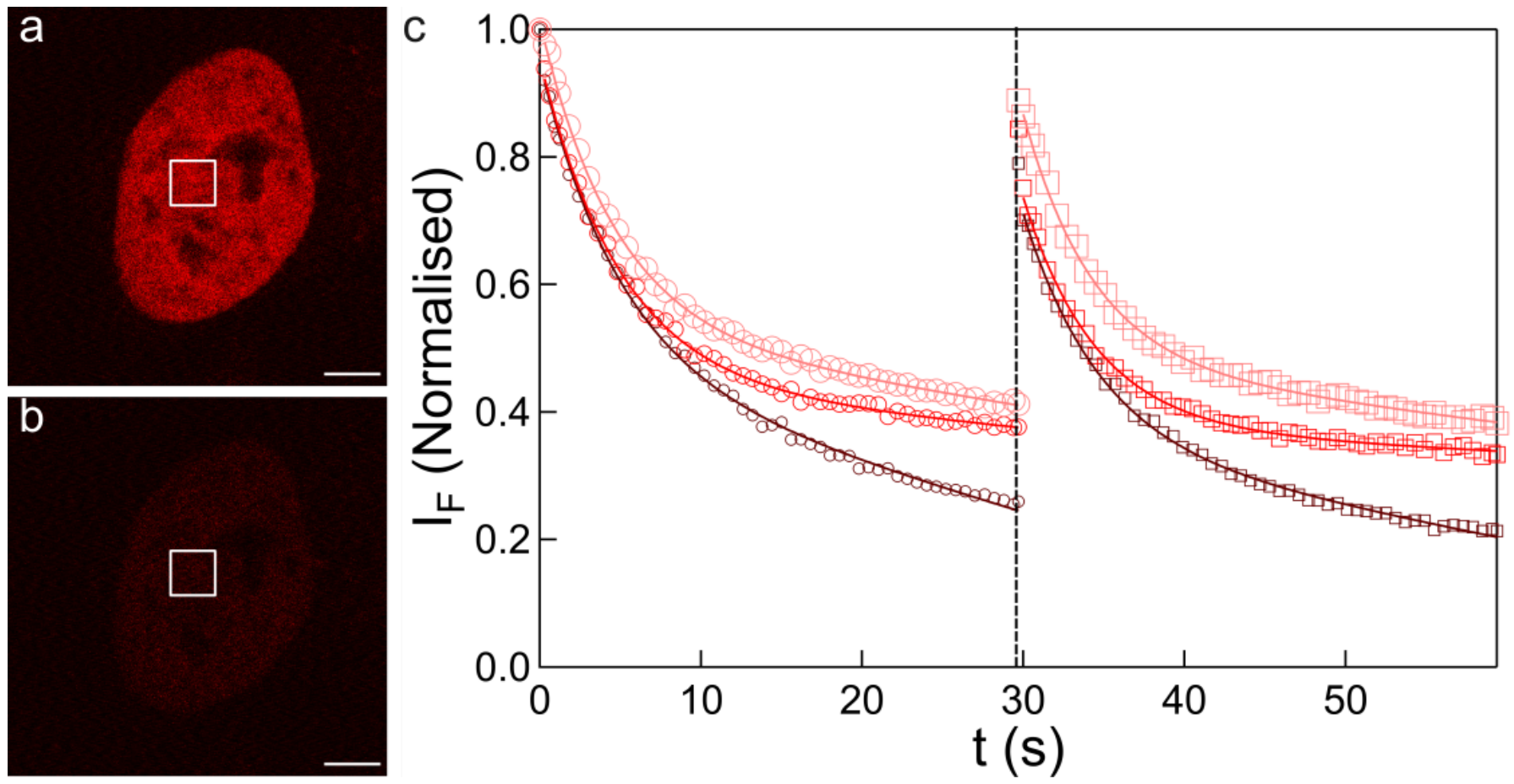

Figure S34: ***FAST** to generate a palette of negative ncRSFPs.* 488 nm light-induced fluorescence emission from **HPAR3Cl** (**a-c**) in **H2B-nirFAST**-labeled living HeLa cells observed in confocal microscopy in the presence of the fluorogen in DMEM pH=7.4. Photoejection is evidenced at 0.1 μM concentration from the contrast at the nuclei between the initial (**a**) and photosteady state (**b**) images, its reversibility by the similar loss of fluorescence normalized by its initial value after two consecutive series of frame acquisition (circles and squares markers after the 1st and 2nd series respectively) in the region of interest at nuclei, and its concentration-dependence by the distinct decays observed at 0.1 (small red markers), 1 (medium red markers), or 10 (large red markers) μM fluorogen concentration (**c**). The vertical dashed line represents a 120 s thermal recovery period (not included in the time axis for compactness). Red lines: exponential fits of the observed fluorescence drops with Eq.(S33) by further adding a slow linear drift to account for photobleaching. Scale bar: 5 μm. $T = 298$ K. See Table S9 for the conditions of image acquisition.

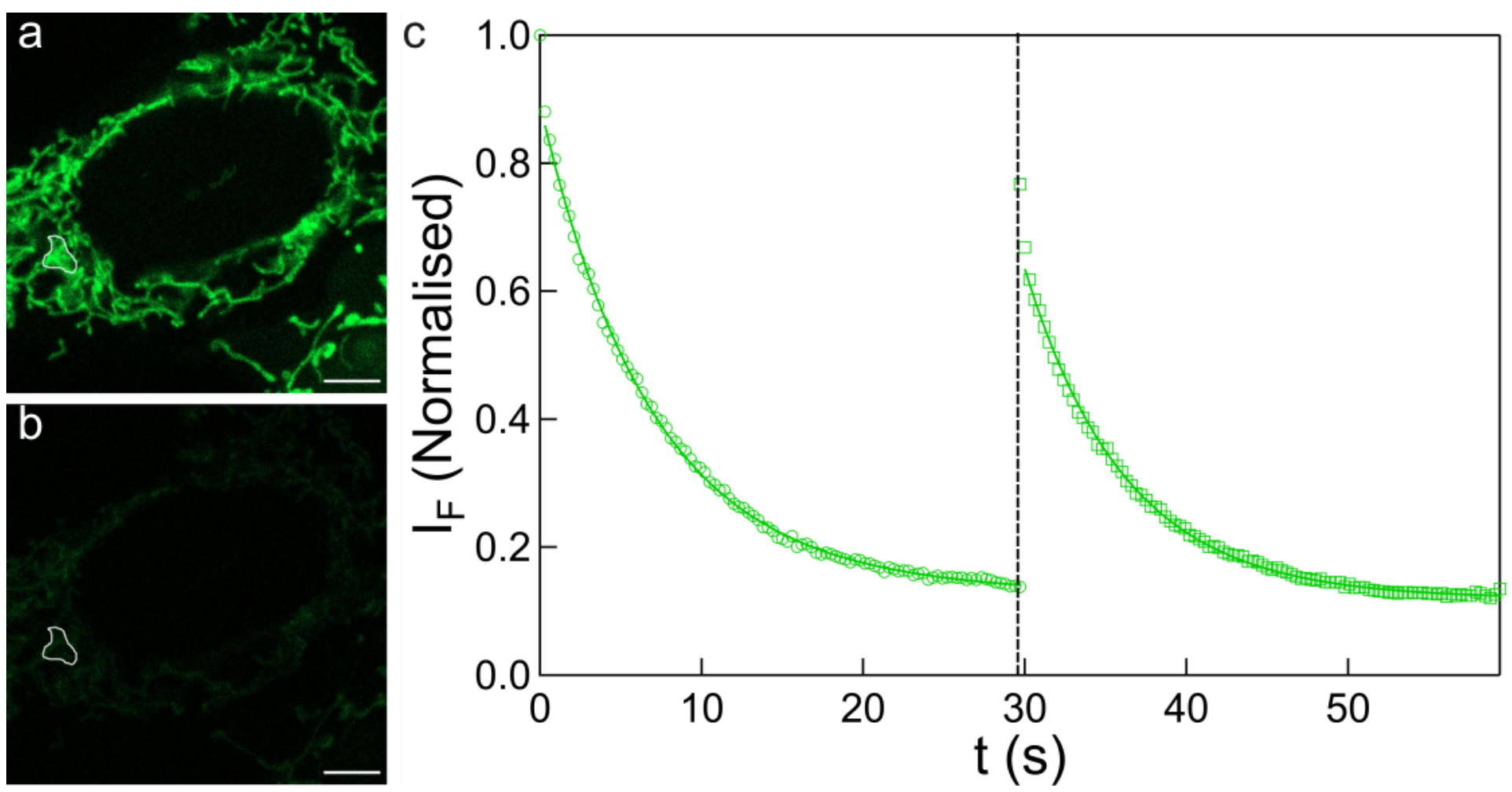

Figure S35: *FAST to generate negative ncRSFPs.* 488 nm light-induced photoejections of the **HBR3F5OM** fluorogen in live HeLa cells expressing **Mito-pFAST** (**a–c**; at mitochondria) observed in confocal microscopy in the presence of 0.1 μM **HBR3F5OM** in DMEM pH=7.4. Photoejection is evidenced from the contrast between the initial (**a**) and photosteady state (**b**) images, its reversibility by the similar loss of fluorescence normalized by its initial value after two consecutive series of frame acquisition (circles then, after 120 s, squares markers) in the region of interest (**c**). The vertical dashed line represents a 120 s thermal recovery period (not included in the time axis for compactness). Green lines: exponential fits of the observed fluorescence drops with Eq.(S33) by further adding a slow linear drift to account for photobleaching. Scale bar: 5 μm. $T = 310$ K. See Table S9 for the conditions of image acquisition.

Photoejection in nirFAST-HPAR3Cl We also evidenced photoejection in live HeLa cells expressing **Mito-nirFAST** at mitochondria. Hence, such HeLa cells were conditioned with **HPAR3Cl** and imaged in confocal microscopy under 488 nm excitation. As displayed in Figure S36, those cells exhibit similar photoejection to the ones that express **H2B-pFAST**.

5.1.1.3 At the membrane

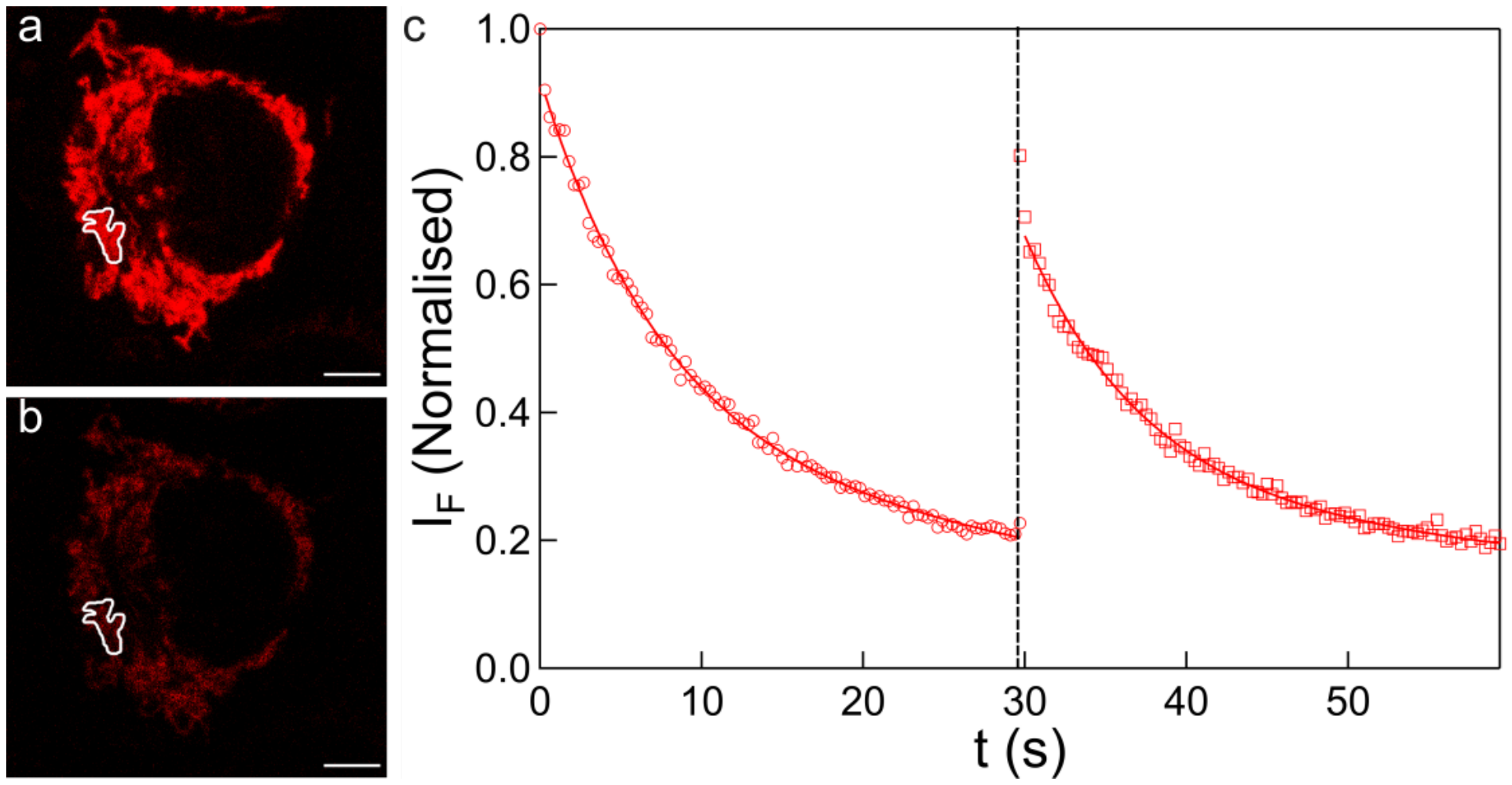

Figure S36: *FAST to generate negative ncRSFPs*. 488 nm light-induced photoejections of the **HPAR3Cl** fluorogen in live HeLa cells expressing **Mito-nirFAST** (**a**–**c**; at mitochondria) observed in confocal microscopy in the presence of 0.1 μM **HPAR3Cl** in DMEM pH=7.4. Photoejection is evidenced from the contrast between the initial (**a**) and photosteady state (**b**) images, its reversibility by the similar loss of fluorescence normalized by its initial value after two consecutive series of frame acquisition (circles then, after 120 s, squares markers) in the region of interest (**c**). The vertical dashed line represents a 120 s thermal recovery period (not included in the time axis for compactness). Red lines: exponential fits of the observed fluorescence drops with Eq.(S33) by further adding a slow linear drift to account for photobleaching. Scale bar: 5 μm. $T = 310$ K. See Table S9 for the conditions of image acquisition.

Photoejection in pFAST-HBR3F5OM To complement the experiments performed on live HeLa cells expressing **H2B-pFAST** in the nucleus, we examined whether photoejection could be observed in live HeLa cells expressing **Lyn11-pFAST** at the membrane. Hence, such HeLa cells were conditioned with **HBR3F5OM** and imaged in confocal microscopy under 488 nm excitation. As displayed in Figure S37, those cells exhibit similar photoejection to the ones that express **H2B-pFAST**.

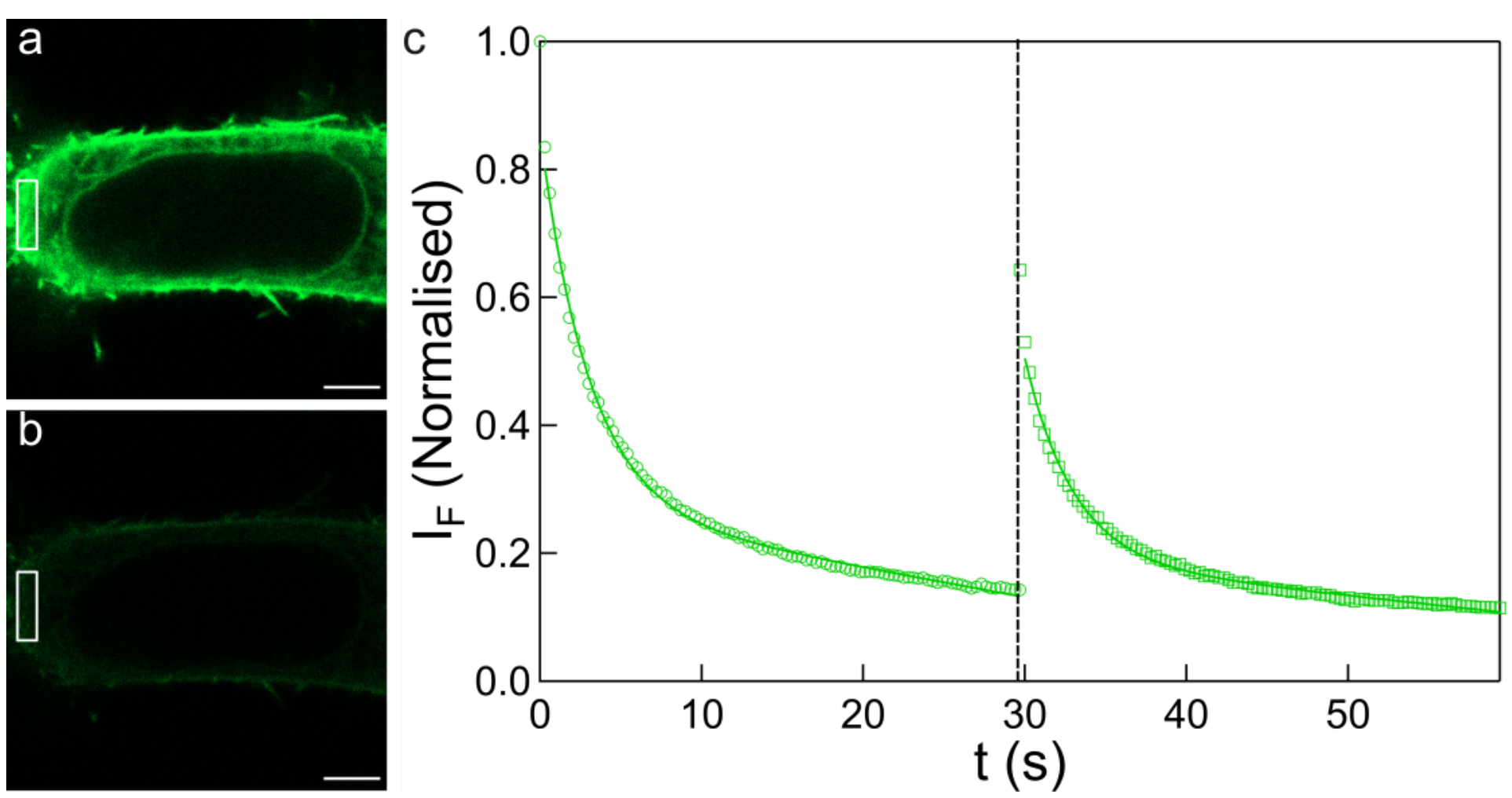

Figure S37: *FAST to generate negative ncRSFPs*. 488 nm light-induced photoejections of the **HBR3F5OM** fluorogen in live HeLa cells expressing **Lyn11-pFAST** (**a**–**c**; at the membrane) observed in confocal microscopy in the presence of 0.1 μM **HBR3F5OM** in DMEM pH=7.4. Photoejection is evidenced from the contrast between the initial (**a**) and photosteady state (**b**) images, its reversibility by the similar loss of fluorescence normalized by its initial value after two consecutive series of frame acquisition (circles then, after 120 s, squares markers) in the region of interest (**c**). The vertical dashed line represents a 120 s thermal recovery period (not included in the time axis for compactness). Green lines: exponential fits of the observed fluorescence drops with Eq.(S33) by further adding a slow linear drift to account for photobleaching. Scale bar: 5 μm. $T = 310$ K. See Table S9 for the conditions of image acquisition.

5.1.2 Labeling of live U2OS cells

In order to evaluate the robustness of the photoejection phenomenon, we turned to U2OS cells and evidenced the occurence of photoejection of **HBR3Cl** from **pFAST** in three different cellular compartments.

5.1.2.1 At the nucleus As displayed in Figure S38, the fluorescence signal at the labeled nucleus monoexponentially decreased by up to 74 % upon repetitive scans in the presence of 1 μM of **HBR3Cl** fluorogen with **H2B-pFAST**. Photoejection reversibility was evidenced by essentially recovering the initial fluorescence level upon introducing a sufficient delay (typically 180 s) before applying a new series of repetitive scans.

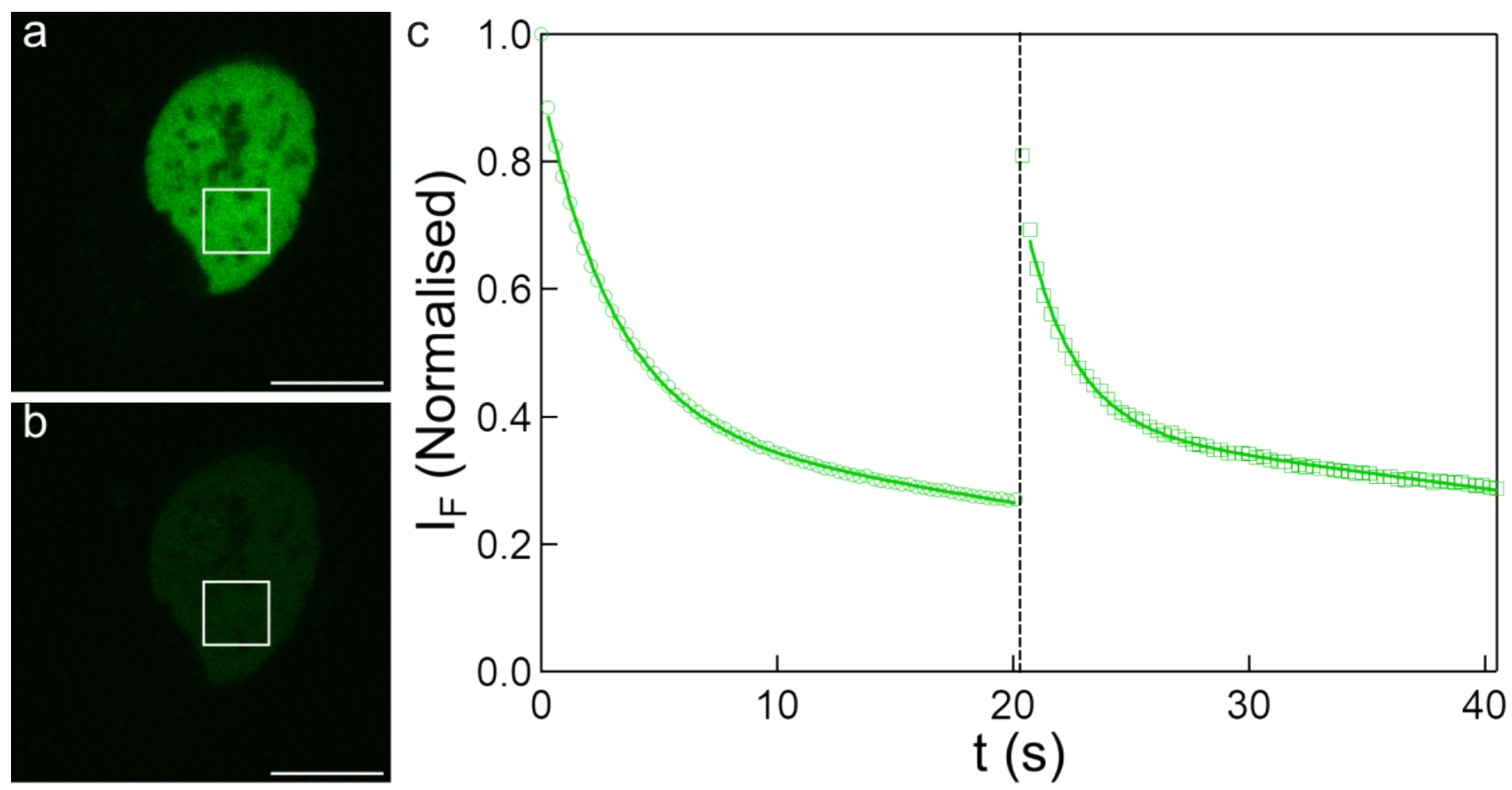

Figure S38: ***FAST** to generate a palette of negative ncRSFPs.* 488 nm light-induced fluorescence emission from **HBR3Cl** (**a-c**) in **H2B-pFAST**-labeled living U2OS cells observed in confocal microscopy in the presence of the fluorogen in DMEM pH=7.4. Photoejection is evidenced at 1 μM concentration from the contrast at the nuclei between the initial (**a**) and photosteady state (**b**) images, its reversibility by the similar loss of fluorescence normalized by its initial value after two consecutive series of frame acquisition (circles and squares markers after the 1st and 2nd series respectively) in the region of interest at nuclei (**c**). The vertical dashed line represents a 180 s thermal recovery period (not included in the time axis for compactness). Green lines: exponential fits of the observed fluorescence drops with Eq.(S33) by further adding a slow linear drift to account for photobleaching. Scale bar: 10 μm. $T = 310$ K. See Table S9 for the conditions of image acquisition.

5.1.2.2 At mitochondria As displayed in Figure S39, the fluorescence signal at the labeled mitochondria monoexponentially decreased by up to 78 % upon repetitive scans in the presence of 1 μM of **HBR3Cl** fluorogen with **Mito-pFAST**. Photoejection reversibility was evidenced by essentially recovering the initial fluorescence level upon introducing a sufficient delay (typically 180 s) before applying a new series of repetitive scans.

5.1.2.3 At the membrane As displayed in Figure S40, the fluorescence signal at the labeled membrane monoexponentially decreased by up to 34 % upon repetitive scans in the presence of 1 μM of **HBR3Cl** fluorogen with **pFAST**. Photoejection reversibility was evidenced by essentially recovering the initial fluorescence level upon introducing a sufficient delay (typically 180 s) before applying a new series of repetitive scans.

5.1.3 Benchmarking of RSpFAST for photoejection

We previously evaluated photoswitching fatigue and reversibility upon submitting the **pFAST**-**HBR3F5OM** system to a series of five photoejection-thermal recovery cycles in live HeLa cells expressing **H2B-pFAST** at the nucleus.[18] Photostability was found fair: after 4 cycles, the fluorescence signal decreased by 12 %.

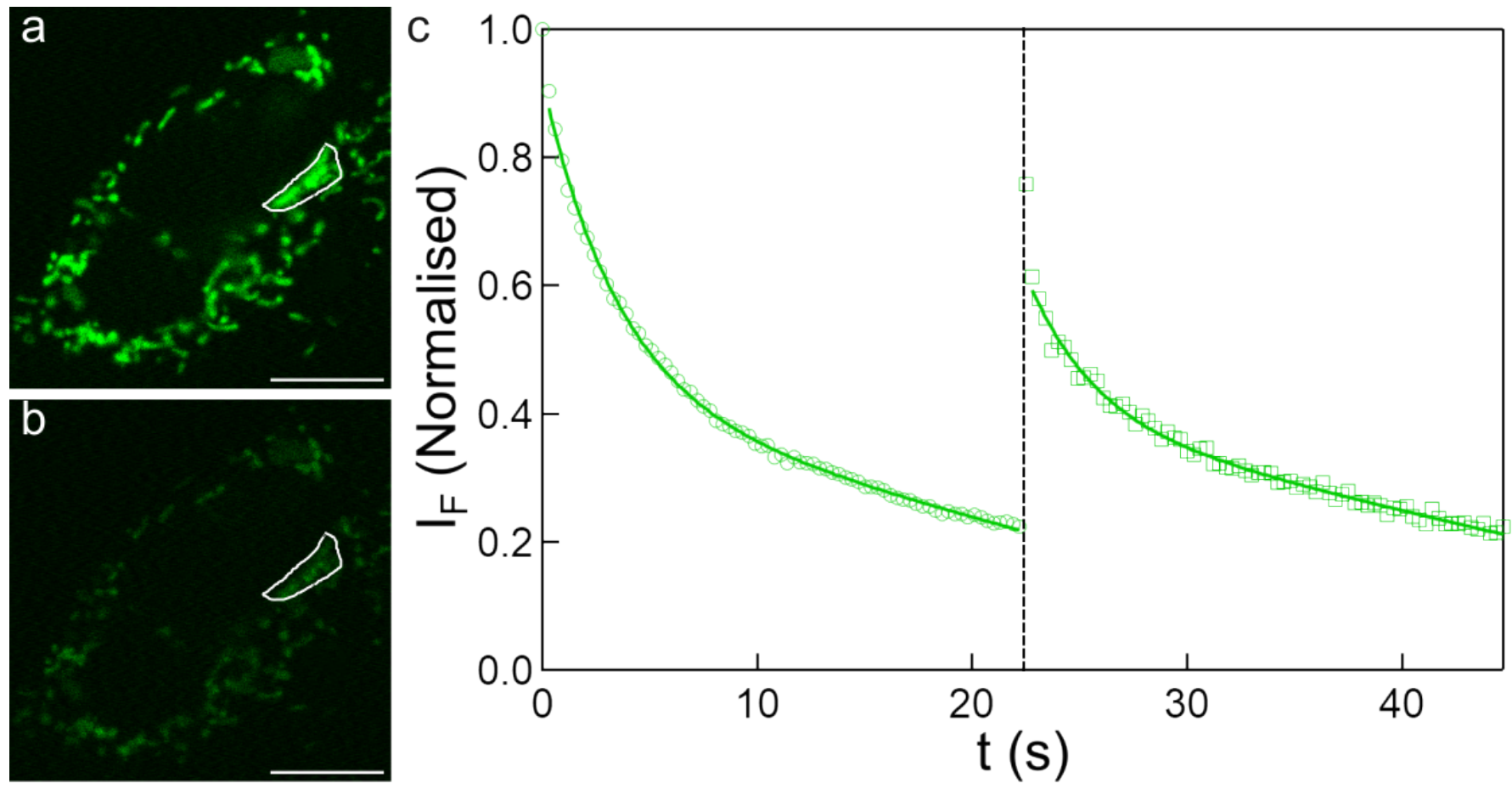

Figure S39: ***FAST** to generate a palette of negative ncRSFPs*. 488 nm light-induced fluorescence emission from **HBR3Cl** (**a-c**) in **Mito-pFAST**-labeled living U2OS cells observed in confocal microscopy in the presence of the fluorogen in DMEM pH=7.4. Photoejection is evidenced at 1 μM concentration from the contrast at mitochondria between the initial (**a**) and photosteady state (**b**) images, its reversibility by the similar loss of fluorescence normalized by its initial value after two consecutive series of frame acquisition (circles and squares markers after the 1st and 2nd series respectively) in the region of interest at mitochondria (**c**). The vertical dashed line represents a 180 s thermal recovery period (not included in the time axis for compactness). Green lines: exponential fits of the observed fluorescence drops with Eq.(S33) by further adding a slow linear drift to account for photobleaching. Scale bar: 5 μm. $T = 310$ K. See Table S9 for the conditions of image acquisition.

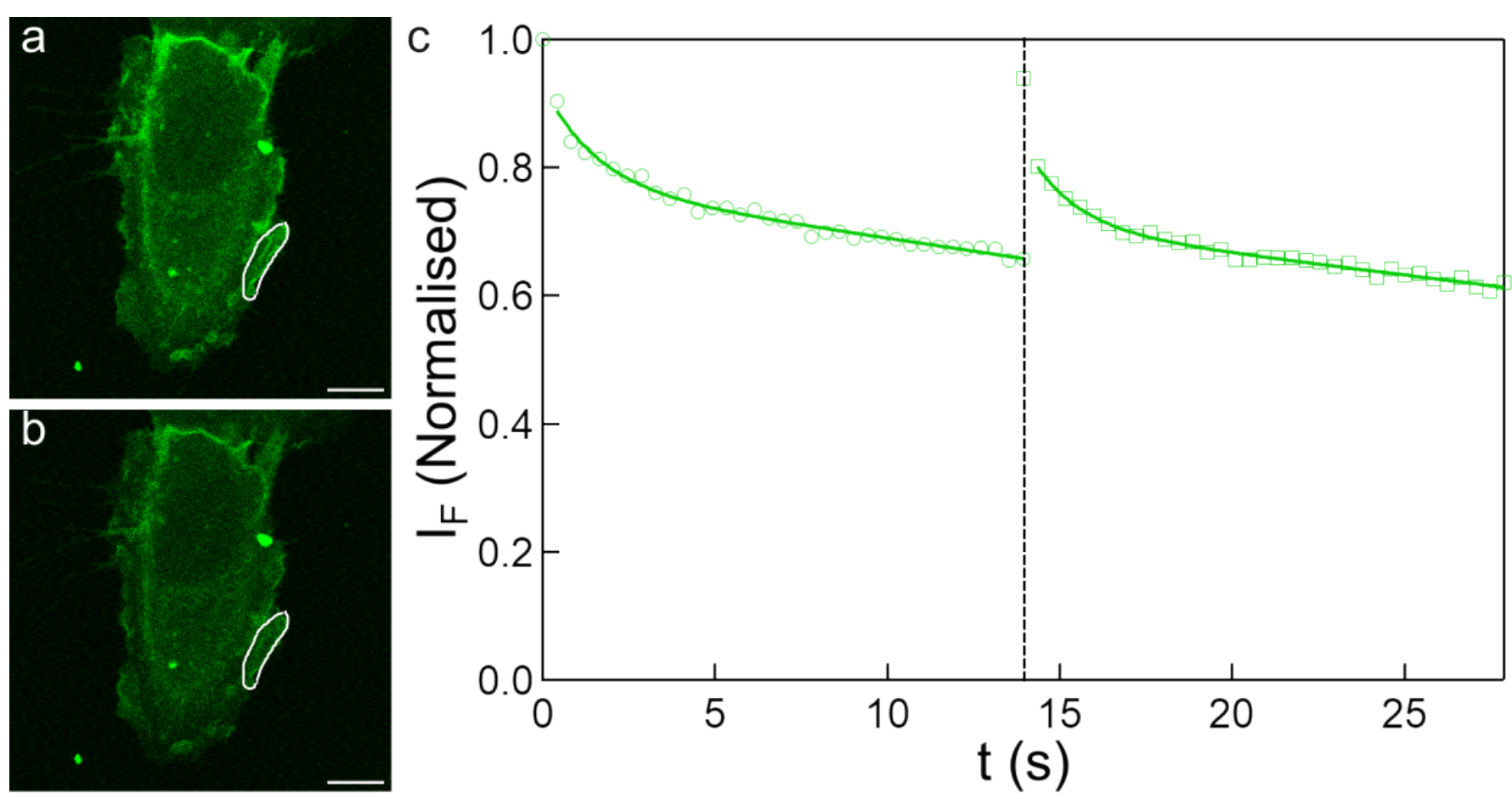

Figure S40: ***FAST** to generate a palette of negative ncRSFPs*. 488 nm light-induced fluorescence emission from **HBR3Cl** (**a-c**) in **Lyn11-pFAST**-labeled living U2OS cells observed in confocal microscopy in the presence of the fluorogen in DMEM pH=7.4. Photoejection is evidenced at 1 μM concentration from the contrast at the membrane between the initial (**a**) and photosteady state (**b**) images, its reversibility by the similar loss of fluorescence normalized by its initial value after two consecutive series of frame acquisition (circles and squares markers after the 1st and 2nd series respectively) in the region of interest at the 180 (**c**). The vertical dashed line represents a 180 s thermal recovery period (not included in the time axis for compactness). Green lines: exponential fits of the observed fluorescence drops with Eq.(S33) by further adding a slow linear drift to account for photobleaching. Scale bar: 5 μm. $T = 310$ K. See Table S9 for the conditions of image acquisition.

In a purpose of comparison of **RSFAST** with regular reversibly photoswitchable fluorescent proteins (green RSFPs), we adopted **Dronpa-2** that exhibits photophysical and photochemical features similar to the ones of our green **RSFASTs** (here exemplified by **pFAST-HBR3F5OM**) and relied on its photophysical/photochemical features as well as photoswitching fatigue and reversibility under the confocal microscopy conditions that were used to drive photoejection with **pFAST-HBR3F5OM**, which we previously reported.[18,19] Hence, we could establish a side-by-side comparison between **pFAST-HBR3F5OM** and **Dronpa-2** in confocal microscopy (see Table S7). It led us to conclude that **pFAST-**

HBR3F5OM and **Dronpa-2** exhibit essentially similar photophysical performances. In contrast, the cross section associated to the 488 nm-driven photoconversion of **pFAST-HBR3F5OM** from the bright to the dark state is much lower than the one of **Dronpa-2** (albeit in the same range of the one of popular RSFPs like **Dronpa** or representatives of the Skylan series; see reference[19]). Moreover, **pFAST-HBR3F5OM** does not exhibit any 405 nm light-driven fluorescence recovery from its dark state; fluorescence recovery is thermally-driven.

Table S7: *Side-by-side comparison between **pFAST-HBR3F5OM** and **Dronpa-2** in confocal microscopy.* T = 310 K.

Fluorescent protein	**pFAST-HBR3F5OM**	**Dronpa-2**
Molar absorption coefficient at 470 nm (M^{-1} cm^{-1})	30000	75000
Fluorescence quantum yield	0.4	0.2
Light intensity requirement at 470 nm (E m^{-2} s^{-1})	0.1	10^{-4} †
Effective switching off cross section at 470 nm (m^2 mol^{-1})	0.3–1.1*	198
Relaxation time for thermal recovery at 298 K (s)	1–10	50
Switching on cross section at 405 nm (m^2 mol^{-1})	–	415
Contrast ratio under 470 nm illumination	0.1	0.1

† Computed from the rate constant from thermal return given in.[19]

* Evaluated from monoexponentially fitting the fluorescence decay displayed in Figure 3c and e from the Main Text of the reference.[18]

5.2 Experiments involving two fluorogens

5.2.1 Labeling of live Hela cells

5.2.1.1 At the nucleus

HBR3Cl:HBR35DOM In order to further investigate the photostability during photoconversion, we subjected HeLa cells labeled with **pFAST** at the nucleus to repeated photoconversion cycles. As displayed in Figure S41, the fluorescence signals in the green and red wavelength channels at the **H2B-pFAST**-labeled nucleus respectively decreased by up to 41 % and increased by up to 483 % upon repetitive scans in the presence of 1 μM of **HBR3Cl** and 0.5 μM of **HBR35DOM**. Photocompetition reversibility was evidenced by essentially recovering the initial fluorescence levels upon introducing a sufficient delay (typically 180 s) before applying new series of repetitive scans. After repeating this illumination-recovery cycle 10 times, the levels of fluorescence of **HBR3Cl** and **HBR35DOM** decreased by only 26 % and 32 % respectively.

HPAR3OM:HBR35DOM Figure S42 shows that 543 nm illumination of live HeLa cells expressing H2B-**nirFAST** conditioned with 0.5:0.25 μM **HPAR3OM**:**HBR35DOM** in confocal microscopy reversibly shifts the initial color of the fluorescence emission at the cell nucleus towards the red one resulting from replacing the initial fluorogen by **HBR35DOM** in the **nirFAST** complex.

HBR3F5OM:HPAR3OM Figure S43 shows that 561 nm illumination of live HeLa cells expressing **H2B-nirFAST** conditioned with 1:1 μM **HBR3F5OM**:**HPAR3OM** in confocal microscopy reversibly shifts the initial hue of the fluorescence emission at the cell nucleus towards the greener one resulting from replacing the photoisomerized **HPAR3OM** fluorogen by **HBR3F5OM** in the **nirFAST** complex.

5.2.2 Labeling of live U2OS cells

In order to evaluate the robustness of the photocompetition phenomenon, we turned to U2OS cells and evidenced the occurence of photocompetition of **HBR3Cl:HBR35DOM** with **pFAST** in three different cellular compartments.

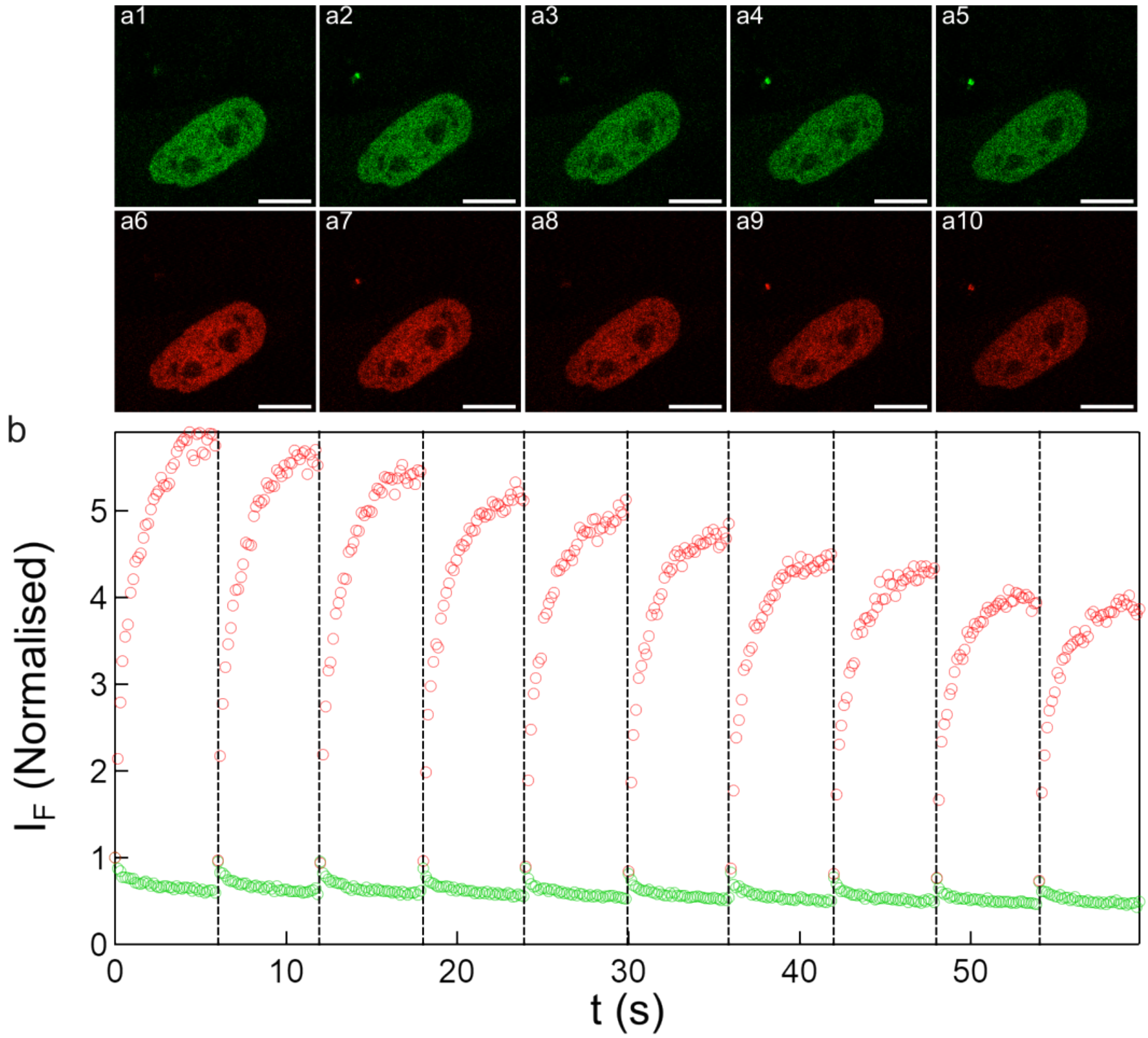

Figure S41: *Benchmarking of the photostability during photoconversion.* **a1-a10**: Fluorescence image of the first frame in the green (**a1-a5**) and last frame in red (**a6-a10**) channels of confocal microscopy from **H2B-pFAST**-labeled live HeLa cells conditioned with 1 μM **HBR3Cl** and 0.5 μM **HBR35DOM** in DMEM pH 7.4 over the course of 10 successive 488 nm light-induced photoejection – thermal recovery cycles (only every second cycle shown); **b**: Evolution of the fluorescence intensity normalized by its initial value in the labeled nucleus shown in **a** over the course of the 10 cycles. Each vertical dashed line represents a 180 s thermal recovery period in the dark (not included in the time axis for compactness). The duration of each illumination period is 5.9 s. See Table S9 for the conditions of image acquisition.

5.2.2.1 At the nucleus As displayed in Figure S44, the fluorescence signals in the green and red wavelength channels at the **H2B-pFAST**-labeled nucleus respectively decreased by up to 46 % and increased by up to 276 % upon repetitive scans in the presence of 1 μM of **HBR3Cl** and 0.5 μM of **HBR35DOM**. Photocompetition reversibility was evidenced by essentially recovering the initial fluorescence levels upon introducing a sufficient delay (typically 120 s) before applying a new series of repetitive scans.

5.2.2.2 At mitochondria As displayed in Figure S45, the fluorescence signals in the green and red wavelength channels at the **Mito-pFAST**-labeled nucleus respectively decreased by up to 39 % and increased by up to 552 % upon repetitive scans in the presence of 1 μM of **HBR3Cl** and 0.5 μM of **HBR35DOM**. Photocompetition reversibility was evidenced by essentially recovering the initial fluorescence levels upon introducing a sufficient delay (typically 120 s) before applying a new series of repetitive scans.

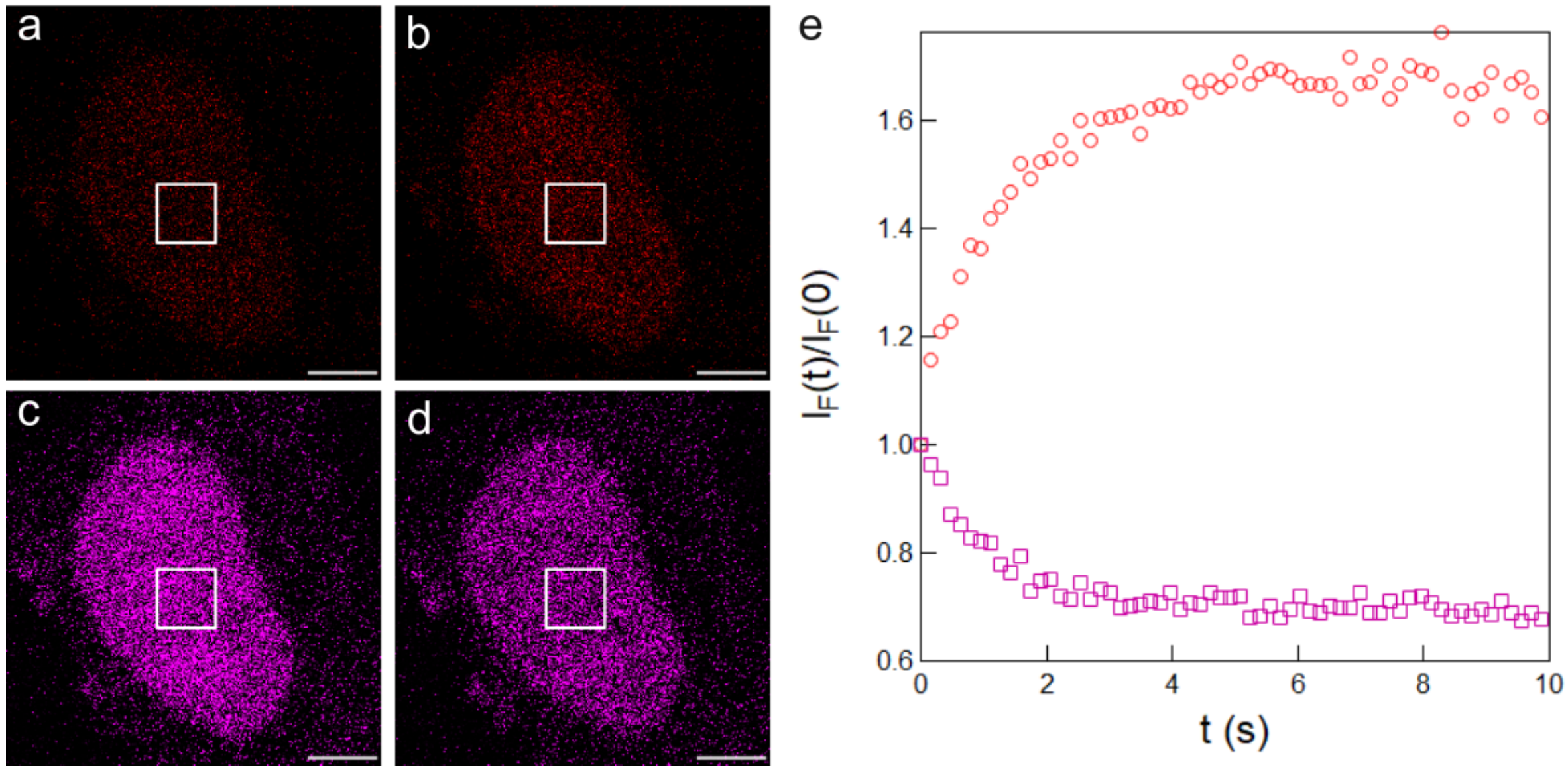

Figure S42: ***FAST** to generate a palette of reversibly photoconvertible fluorescent proteins*. 543 nm light-induced change of fluorescence hue from **HPAR3OM**:**HBR35DOM** in H2B-**nirFAST** labelled living HeLa cells observed in confocal microscopy in the presence of 0.5 μM **HPAR3OM** and 0.25 μM **HBR35DOM** in DMEM pH =7.4. Photoconversion of the fluorescence hue is evidenced from the fluorescence contrast at the nuclei between the initial (**a**,**c**) and photosteady state (**b**,**d**) images quantified at $I = 2.5 \times 10^3$ E·m^{-2}·s^{-1} light intensity; **e**: Time evolution of the NIR (pink markers for **HPAR3OM**) and red (red markers for **HBR35DOM**) fluorescence normalized by its initial value at $I = 2.5 \times 10^3$ E·m^{-2}·s^{-1} light intensity with 1 μs dwell time in the region interest at nuclei. Scale bar: 5 μm. $T = 298$ K. See Table S9 for the conditions of image acquisition.

In order to further investigate potential phototoxicity due to the photoconversion process, we submitted U2OS cells expressing **pFAST** at the mitochondria to repeated photoconversion cycles. As displayed in Figure S46, the fluorescence signals in the green and red wavelength channels at the mitochondria respectively decreased by up to 21 % and increased by up to 308 % upon repetitive scans in the presence of 1 μM of **HBR3Cl** and 0.5 μM of **HBR35DOM**. Photocompetition reversibility was evidenced by essentially recovering the initial fluorescence levels upon introducing a sufficient delay (180 s) before applying new series of repetitive scans. After repeating this illumination-recovery cycle 5 times, the levels of fluorescence of **HBR3Cl** and **HBR35DOM** decreased by only 5 % and 17 % respectively. Furthermore, the morphology of mitochondria was unaffected, suggesting that no phototoxicity occurred.

5.2.2.3 At the membrane As displayed in Figure S47, the fluorescence signals in the green and red wavelength channels at the **Lyn11-pFAST**-labeled nucleus respectively decreased by up to 24 % and increased by up to 162 % upon repetitive scans in the presence of 1 μM of **HBR3Cl** and 0.5 μM of **HBR35DOM**. Photocompetition reversibility was evidenced by essentially recovering the initial fluorescence levels upon introducing a sufficient delay (typically 120 s) before applying a new series of repetitive scans.

5.3 Multiplexed imaging experiments

We here present an alternative of the classification method reported in the Main Text: We use manually identified Regions Of Interest (ROIs) containing pixels only belonging to class **nirFAST** or class **pFAST** to internally train a classifier and apply the classification to the whole image to retrieve the segmentation mask.

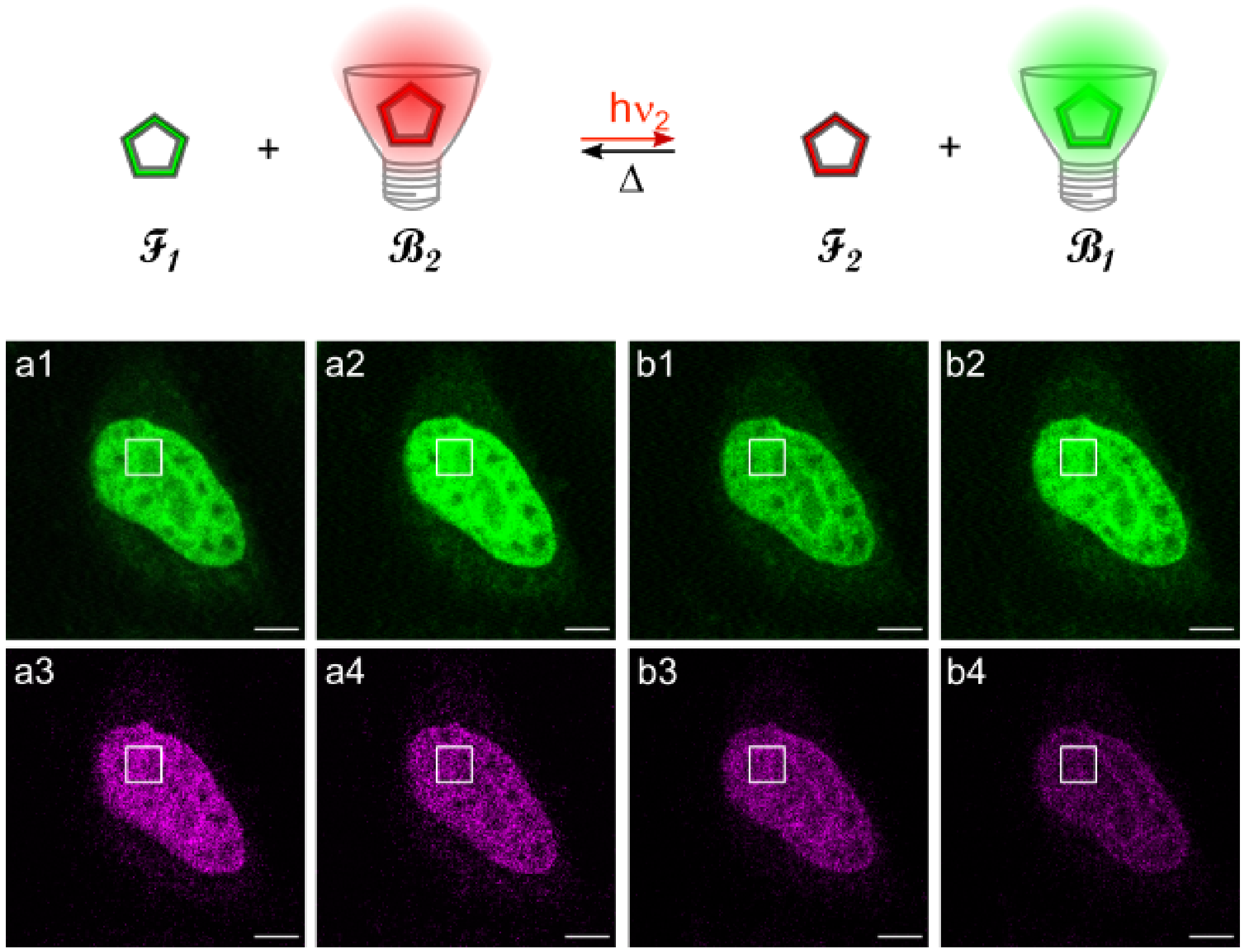

Figure S43: ***FAST** to generate a palette of reversibly photoconvertible fluorescent proteins*. 639 nm light ($I = 1.6 \times 10^4$ E·m^{-2}·s^{-1})-induced change of fluorescence hue from **HBR3F5OM**:**HPAR3OM** in H2B-**nirFAST** labelled living HeLa cells observed in confocal microscopy in the presence of 1 μM **HBR3F5OM** and 1 μM **HPAR3OM** in DMEM pH =7.4. Photoconversion of the fluorescence hue is evidenced from the fluorescence contrast at the nuclei between the initial (**a1**,**a3)** and photosteady state (**a2**,**a4**) images upon exciting at 488 nm at $I = 1.7 \times 10^4$ E·m^{-2}·s^{-1} light intensity. Evidence for reversibility is obtained from the similar changes of the fluorescence hue between the initial (**a1/b1** vs **a3/b3**) and photosteady (**a2/b2** vs **a4/b4**) states for two consecutive series of frame acquisition: the **HBR3F5OM** fluorescence signal increased by 10 and 12 % and the **HPAR3OM** one decreased by 24 and 29 % during the first and second series of frame acquisition respectively. Scale bar: 5 μm. $T = 310$ K. See Table S9 for the conditions of image acquisition.

5.3.1 Mixture of living Hela cells labeled at the nucleus and at mitochondria

We first implemented this strategy by discriminating **pFAST** and **nirFAST** labeling in two different cells. For illustration, we retained a mixture of live U2OS cells expressing **H2B-nirFAST** at the nucleus and **Mito-pFAST** at mitochondria. After conditioning with 1:0.5 μM **HBR3Cl**:**HBR35DOM**, this sample was submitted to acquisition of a sequence of confocal microscopy frames at 488 nm. The images were then processed to retrieve the time evolution of the fluorescence response at each pixel in the green and red spectral channels, mostly reporting on the fluorescence emission from **FAST-HBR3Cl** and **FAST-HBR35DOM** respectively.

The following supervised classification scheme was performed to distinguish the two cells populations. We selected a Region of Interest (ROI) manually corresponding to each class, trained a machine learning algorithm to classify the pixels within the ROIs, then applied the trained model to classify the pixels of the whole image. In details, the videos were downscaled by averaging and a mask was applied to separate the pixels of interest from the background. We selected an ROI inside the nucleus of the **pFAST**-marked cell, and an ROI inside the mitochondria of the **nirFAST**-marked cell (Figure S48a). The red and green time series from those pixels were normalized to their respective first point and

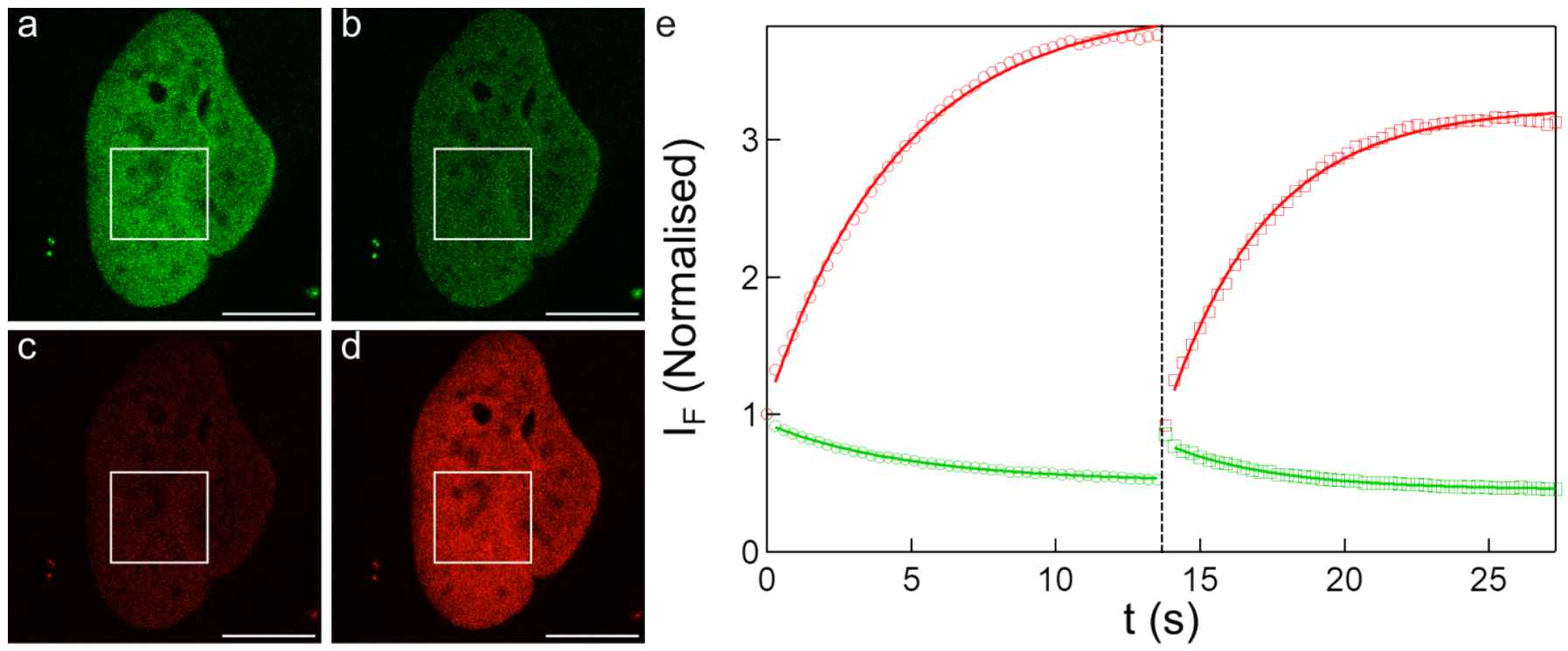

Figure S44: ***FAST** to generate a palette of ncRCFPs.* **a-d**: 488 nm light-induced changes of fluorescence emission in two wavelength channels of confocal microscopy from **H2B-pFAST**-labeled live U2OS cells conditioned with the 1 μM **HBR3Cl**:0.5 μM **HBR35DOM** mixture of fluorogens in DMEM pH 7.4. The fluorescence change is evidenced from the contrast between the initial (**a**,**c**) and photostationary state (**b**,**d**) images of the nuclei; **e**: Time trace of the fluorescence normalized by its initial value in the region of interest at nuclei. Its reversibility is evidenced by the similarity of the time traces after two consecutive series of frame acquisition (circles and squares markers after the 1st and 2nd series respectively) separated by 120 s thermal recovery period in the dark (not included in the time axis for compactness). Green lines: exponential fits of the observed fluorescence drops with Eq.(S33) by further adding a slow linear drift to account for photobleaching. Scale bar: 5 μm. $T = 310$ K. See Table S9 for the conditions of image acquisition.

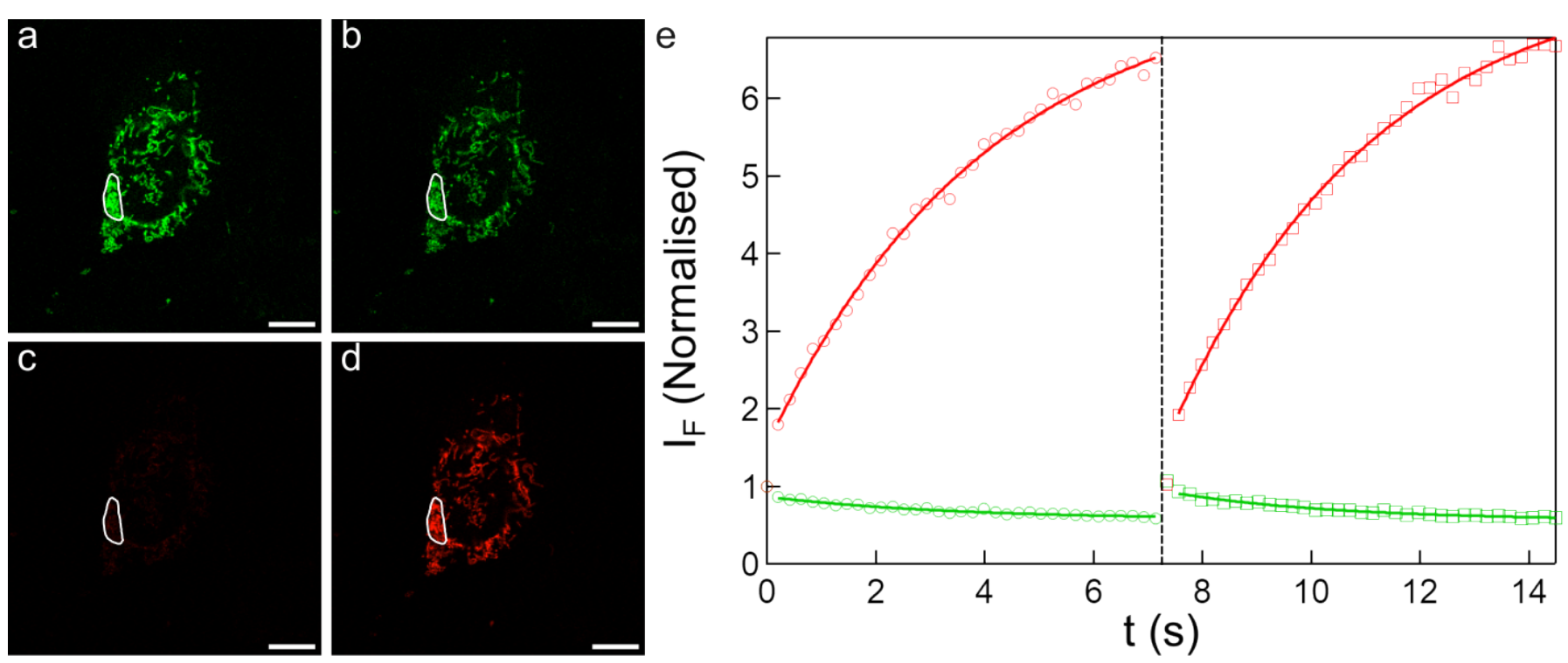

Figure S45: ***FAST** to generate a palette of negative ncRSFPs.* **a-d**: 488 nm light-induced changes of fluorescence emission in two wavelength channels of confocal microscopy from **mito-pFAST**-labeled live U2OS cells conditioned with the 1 μM **HBR3Cl**:0.5 μM **HBR35DOM** mixture of fluorogens in DMEM pH 7.4. The fluorescence change is evidenced from the contrast between the initial (**a**,**c**) and photostationary state (**b**,**d**) images of the nuclei; **e**: Time trace of the fluorescence normalized by its initial value in the region of interest at nuclei. Its reversibility is evidenced by the similarity of the time traces after two consecutive series of frame acquisition (circles and squares markers after the 1st and 2nd series respectively) separated by 120 s thermal recovery period in the dark (not included in the time axis for compactness). Green lines: exponential fits of the observed fluorescence drops with Eq.(S33) by further adding a slow linear drift to account for photobleaching. Scale bar: 5 μm. $T = 310$ K. See Table S9 for the conditions of image acquisition.

concatenated to form a time series of 150 datapoints. We applied a Principal Components Analysis (PCA) to reduce the data dimension to 8. Then, we applied a Linear Discriminant Analysis (LDA) supervised by assigning the label of the corresponding ROI. We subsequently applied the trained model to classify the pixels of the whole image and assigned the predicted class to the pixels of the mask, resulting in a segmentation mask (Figure S48b.). We overlay the segmented mask with the initial image in Figure S48c. The choices of algorithms are justified in the section 5.4.

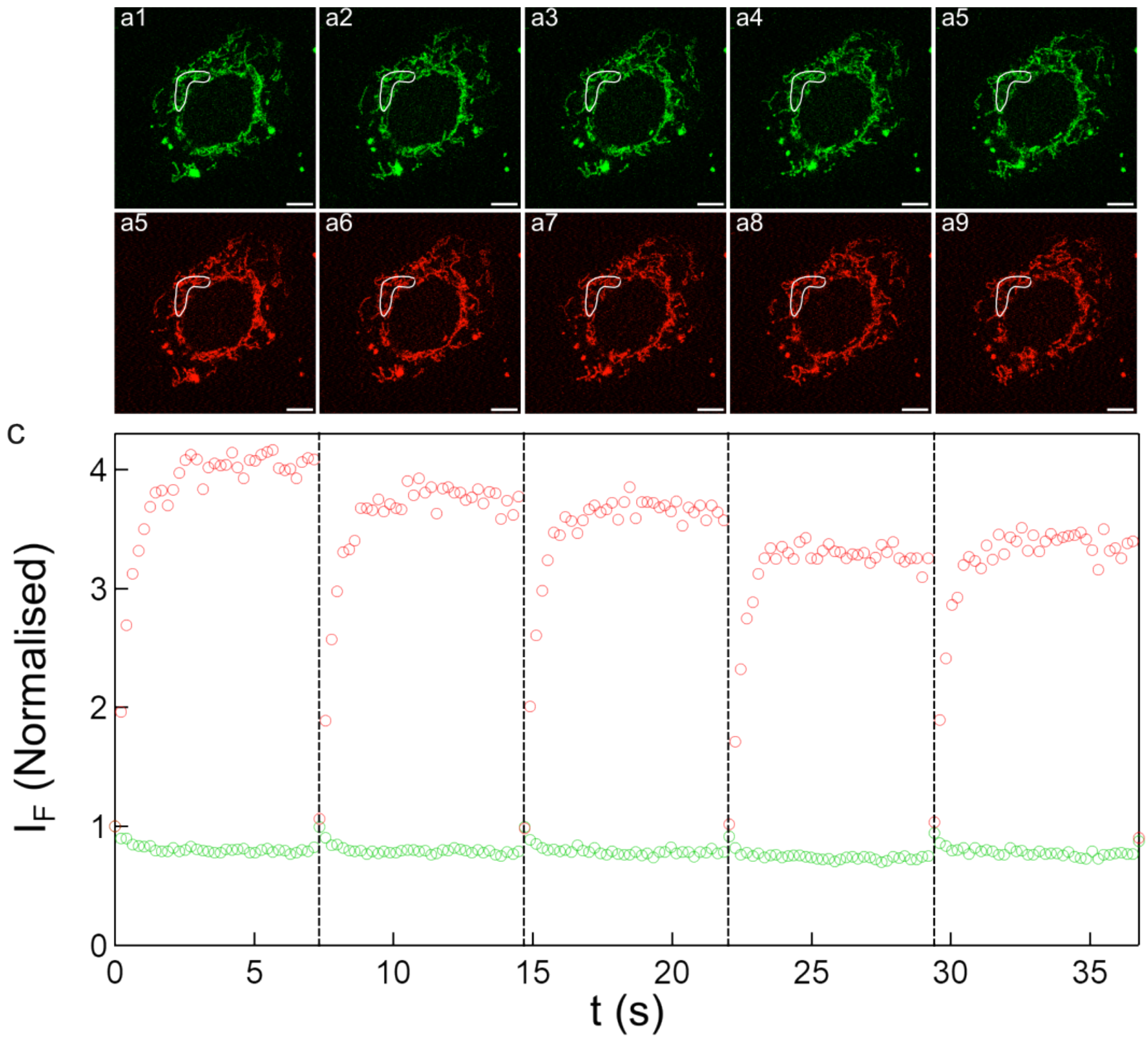

Figure S46: *Evaluation of potential phototoxicity during photoconversion.* **a1-a10**: Fluorescence image of the first frame in the green (**a1-a5**) and last frame in red (**a6-a10**) channels of confocal microscopy from **Mito-pFAST**-labeled live U2OS cells conditioned with 1 μM **HBR3Cl** and 0.5 μM **HBR35DOM** in DMEM pH 7.4 over the course of 5 successive 488 nm light-induced photoejection and thermal recovery cycles; **b**: Evolution of the fluorescence intensity normalized by its initial value in the labeled mitochondria shown in **a** over the course of the 5 cycles. Each vertical dashed line represents a 180 s thermal recovery period in the dark (not included in the time axis for compactness). The duration of each illumination period is 5.9 s. See Table S9 for the conditions of image acquisition.

5.3.2 Living U2OS cells doubly labeled at the nucleus and at mitochondria

We then implemented this strategy by discriminating **pFAST** and **nirFAST** labeling at two different compartments of a same cell. For illustration, we retained live U2OS cells expressing both **H2B-nirFAST** at the nucleus and **Mito-pFAST** at mitochondria. After conditioning with 1:0.5 μM **HBR3Cl**:**HBR35DOM**, this sample was submitted to acquisition of a sequence of confocal microscopy frames at 488 nm. The images were then processed to retrieve the time evolution of the fluorescence response at each pixel in the green and red spectral channels, mostly reporting on the fluorescence emission from **FAST-HBR3Cl** and **FAST-HBR35DOM** respectively. The same supervised classification scheme was performed to distinguish the two compartments (see previous section). The ROIs were selected inside the nucleus and mitochondria of the same cell (Figure S49a) to train the model, then classify the pixels of the whole image and obtain a segmented mask of the illuminated pixels with the predicted class (Figure S49b). We overlay the segmented mask with the initial image in Figure S49c.

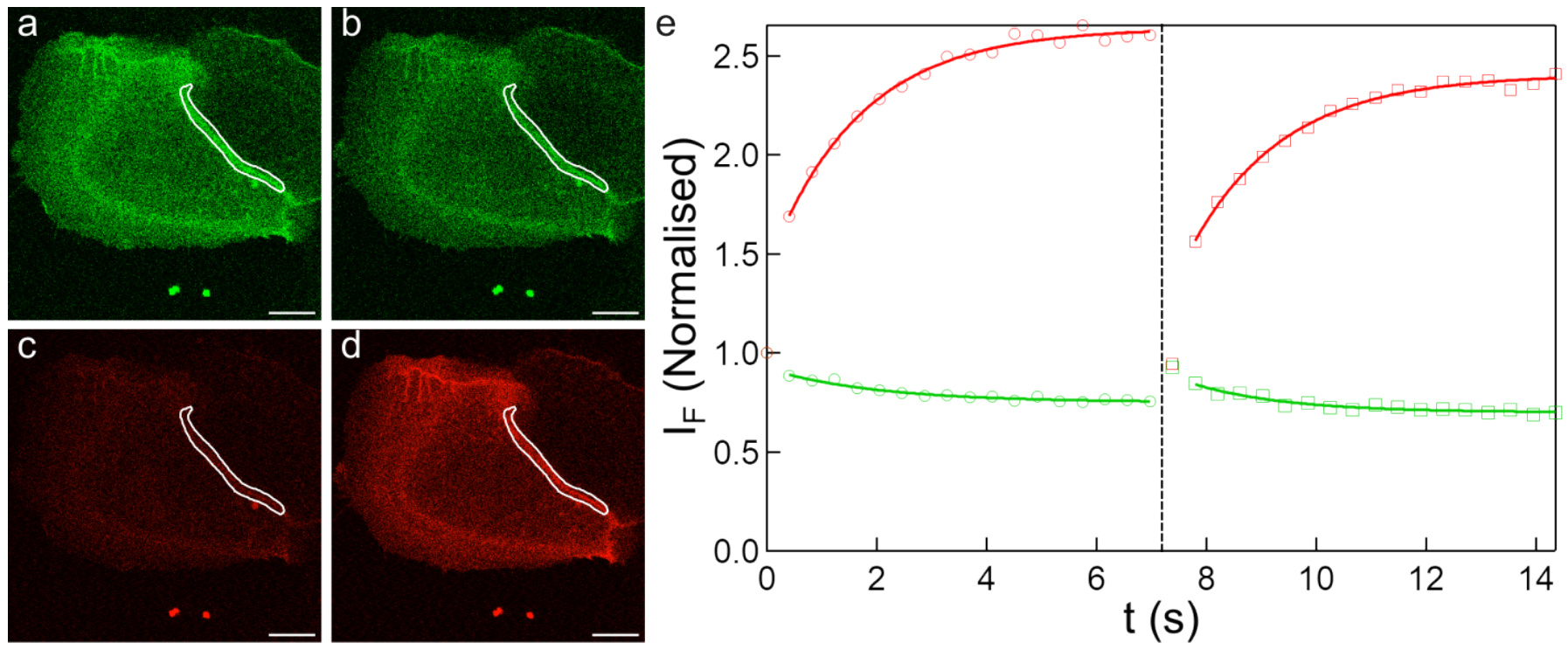

Figure S47: ***FAST** to generate a palette of negative ncRSFPs.* **a-d**: 488 nm light-induced changes of fluorescence emission in two wavelength channels of confocal microscopy from **Lyn11-pFAST**-labeled live U2OS cells conditioned with the 1 μM **HBR3Cl**:0.5 μM **HBR35DOM** mixture of fluorogens in DMEM pH 7.4. The fluorescence change is evidenced from the contrast between the initial (**a**,**c**) and photostationary state (**b**,**d**) images of the nuclei; **e**: Time trace of the fluorescence normalized by its initial value in the region of interest at nuclei. Its reversibility is evidenced by the similarity of the time traces after two consecutive series of frame acquisition (circles and squares markers after the 1st and 2nd series respectively) separated by 120 s thermal recovery period in the dark (not included in the time axis for compactness). Green lines: exponential fits of the observed fluorescence drops with Eq.(S33) by further adding a slow linear drift to account for photobleaching. Scale bar: 5 μm. $T = 310$ K. See Table S9 for the conditions of image acquisition.

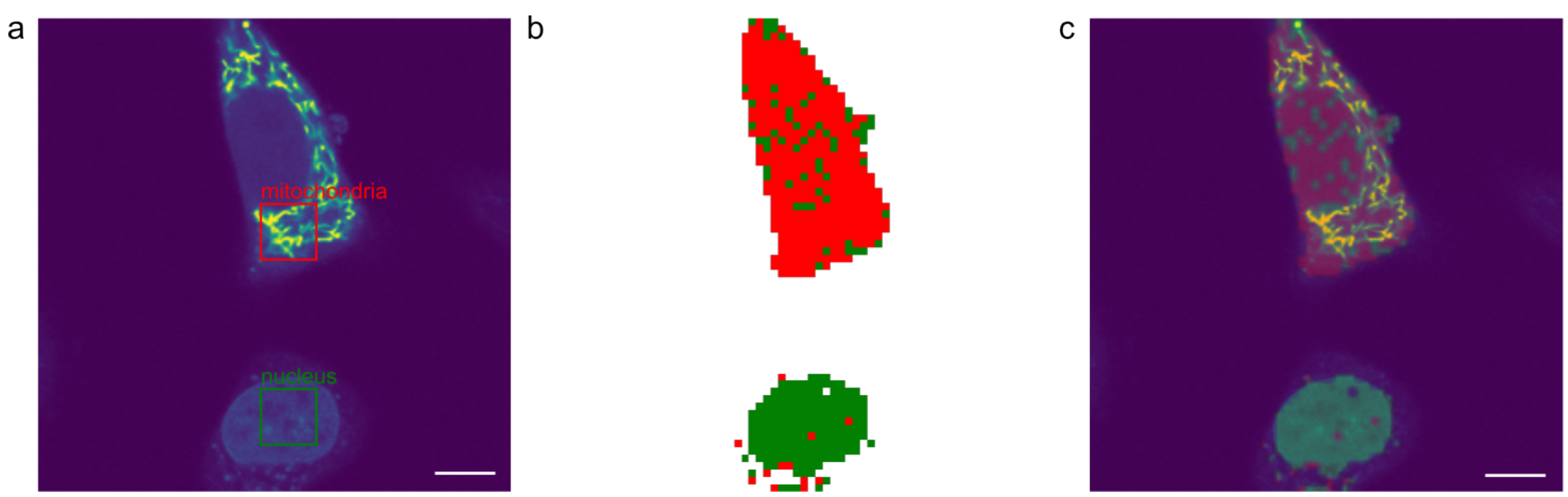

Figure S48: *rcFASTs for dual color dynamic contrast in live U2OS cells conditioned with 1:0.5 μM **HBR3Cl:HBR35DOM** and expressing **H2B-nirFAST** or **Mito-pFAST**.* **a:** Average image of the red and green channel combined (488 nm light in confocal microscopy). The ROIs used for the training of the classifier are displayed, one ROI generates the training data corresponding to one class, with two classes in total: **pFAST** and **nirFAST**. **b:** Segmentation mask obtained after applying the classifier trained on the ROIs to the full image (limited to the unnmasked pixels). Class prediction **pFAST** is displayed in red while **nirFAST** is colored in green. **c:** Overlay of the average confocal image and the predicted mask. Scale bar: 5 μm. $T = 310$ K. See Table S9 for the conditions of image acquisition.

We eventually implemented this strategy by discriminating **pFAST** and **nirFAST** in live U2OS cells expressing both **H2B-pFAST** at the nucleus and **Mito-nirFAST** at mitochondria. After conditioning with 1:0.5 μM **HBR3Cl**:**HBR35DOM**, this sample was submitted to acquisition of a sequence of confocal microscopy frames at 488 nm. The images were then processed to retrieve the time evolution of the fluorescence response at each pixel in the green and red spectral channels, mostly reporting on the fluorescence emission from **FAST-HBR3Cl** and **FAST-HBR35DOM** respectively.The same supervised classification scheme was performed to distinguish the two compartments (see previous section). The ROIs were selected inside the nucleus and mitochondria of the same cell (Figure S50a) to train the model, then classify the pixels of the whole image and obtain a segmented mask of the illuminated pixels with the predicted class (Figure S50b). We overlay the segmented mask with the initial image in Figure S50c.

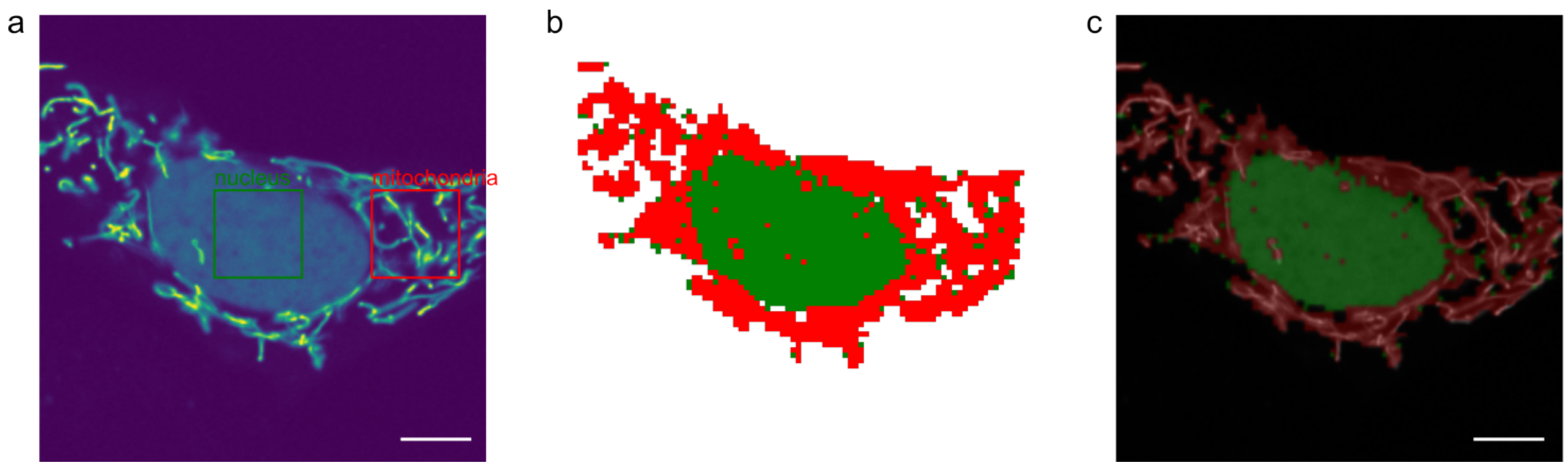

Figure S49: *rcFASTs for dual color dynamic contrast in live U2OS cells conditioned with 1:0.5 μM **HBR3Cl:HBR35DOM** and expressing both **H2B-nirFAST** and **Mito-pFAST**.* **a:** Average image of the red and green channel combined (488 nm light in confocal microscopy). The ROIs used for the training of the classifier are displayed, one ROI generates the training data corresponding to one class, with two classes in total: **pFAST** and **nirFAST**. The time series are retrieved after downscaling by averaging of the videos and applying a mask to remove the background pixels; **b:** Segmentation mask obtained after applying the classifier trained on the ROIs to the full image (limited to the unmasked pixels). Class prediction **pFAST** is displayed in red while **nirFAST** is colored in green; **c:** Overlay of the average confocal image and the predicted mask. Scale bar: 5 μm. $T = 310$ K. See Table S9 for the conditions of image acquisition.

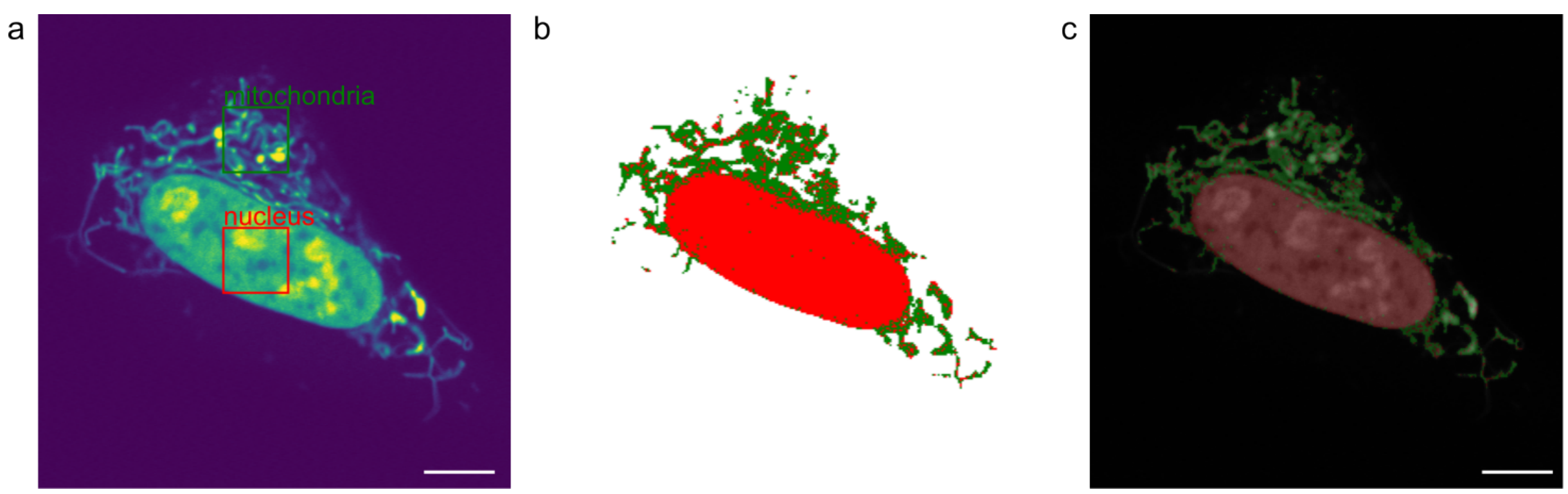

Figure S50: *rcFASTs for dual color dynamic contrast in live U2OS cells conditioned with 1:0.5 μM **HBR3Cl:HBR35DOM** and expressing both **H2B-pFAST** and **Mito-nirFAST**.* **a:** Average image of the red and green channel combined (488 nm light in confocal microscopy). The ROIs used for the training of the classifier are displayed, one ROI generates the training data corresponding to one class, with two classes in total: **pFAST** and **nirFAST**. The time series are retrieved after downscaling by averaging of the videos and applying a mask to remove the background pixels; **b:** Segmentation mask obtained after applying the classifier trained on the ROIs to the full image (limited to the unmasked pixels). Class prediction **pFAST** is displayed in red while **nirFAST** is colored in green; **c:** overlay of the average confocal image and the predicted mask. Scale bar: 5 μm. $T = 310$ K. See Table S9 for the conditions of image acquisition.

5.4 Comparative analyses in relation to multiplexed imaging

In order to evaluate the potential of the light-induced time evolutions of the fluorescence in the green and red channels for multiplexed fluorescence imaging, we examined discrimination among a pool of live HeLa cells labeled at the nucleus with nine distinct H2B-xFAST (x=Y, p, nir, fr, t, green, i, o, Tsi) by using 1:0.5 μM **HBR3Cl**:**HBR35DOM** mixture. Note that we did not match the expression levels nor we did not control the initial distributions of the fluorescence level in the investigated labeled cells, which were imaged. In order to give an order of magnitude of the distribution of the fluorescence level and of the expression level, we introduce relevant information in Table S10.
Refer to Table S8 and Figure S51 for a summary of comparative analyses performed to assess the robustness of the classification pipeline.

- *Six vs. seven vs. eight proteins:* The pipeline was run on the dataset containing six proteins augmented with experimental data corresponding to the protein iFAST and/or oFAST and/or YFAST, using identical segmentation, preprocessing, and training steps. When adding either YFAST or iFAST, the confusion matrix indicates that either additional protein mainly introduces confusion with greenFAST, which exhibits similar kinetic features. When adding oFAST, confusion mainly arises with tFAST and TsiFAST. When adding the three extra proteins, the overall accuracy drops from 98% (six proteins) to 75% (nine proteins) with a gradual performance decay as the proteins are added (see table S8);

- *One vs. two fluorescence channels:* The full analysis on the six proteins was repeated using only the red or green fluorescence channel to assess the gain of dual-channel information. We observed a drop of performance by approximately 20% when using only one channel. To reach performance comparable to the two-channel model (98% accuracy), we had to limit the dataset to 4 proteins (frFAST, greenFAST, nirFAST and tFAST) when using only the green channel (accuracy 97%) and to 3 proteins (pFAST, greenFAST and TsiFAST) when using only the red channel (accuracy 100%). See Figure S52 for the 2D representation of the separability of the proteins when using one channel only;

- *Normalization strategies:* The effect of different normalization strategies on classification performance was evaluated. We compared normalization to the first time point (used in the main analysis) to min-max normalization (0-1 scaling). The results indicate that normalizing to the first point yields better classification accuracy. A confusion between TsiFAST and pFAST appears with this normalisation, signalling that the amplitude change (and not only the kinetic constant) is an important component for the classification.

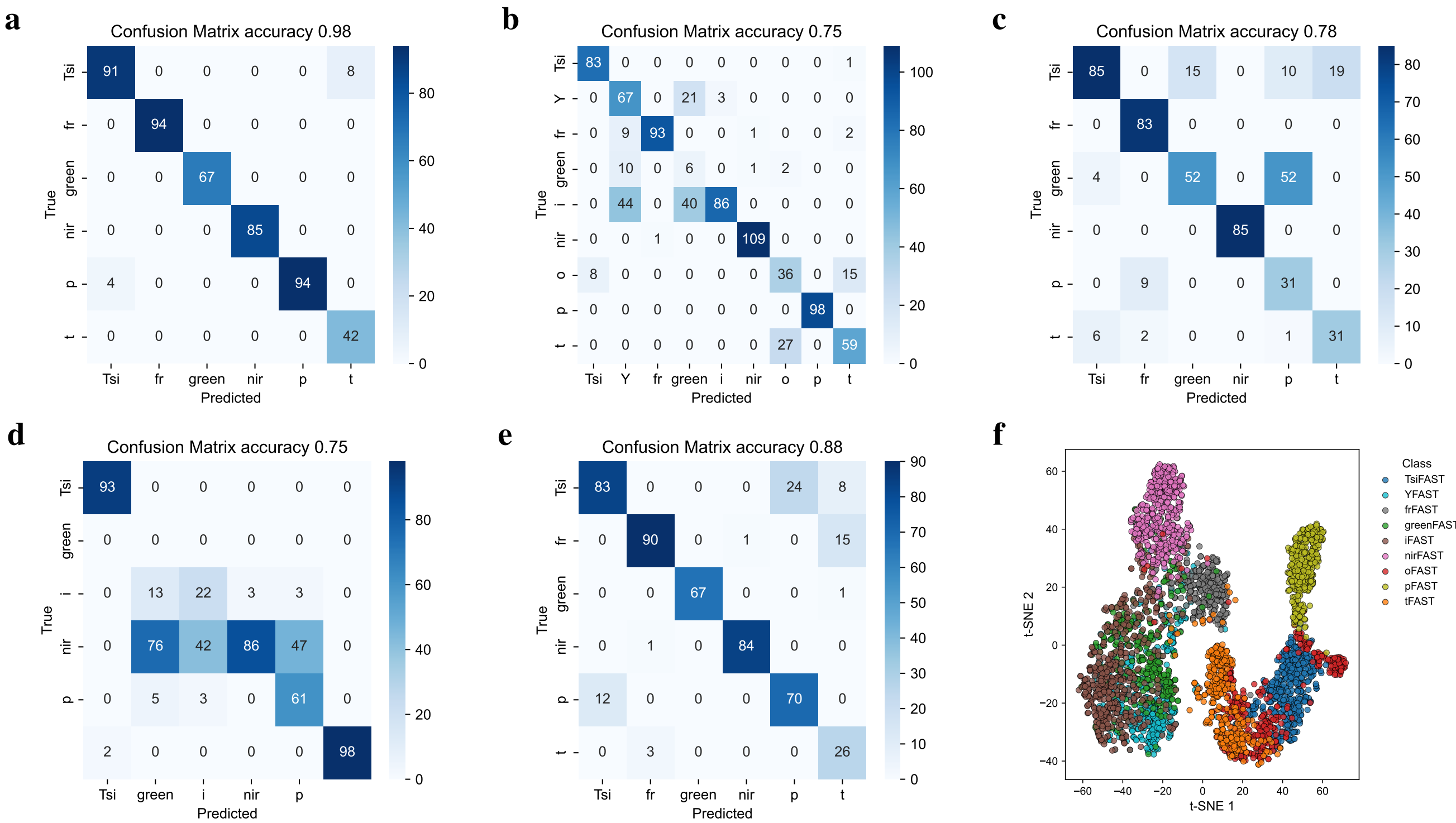

Figure S51: *Study of the parameters affecting the quality of the classification.* **a-e**: Confusion matrices for the classification by Linear Discriminant Analysis model after dimension reduction by PCA to eight components. **f**: A linear supervised reduction of dimension of the red and green traces (Linear Discriminant Analysis, dimension 150 reduced to dimension $n-1$, where n is the number of proteins) is followed by a non-linear dimension reduction technique (t-SNE, dimension $n-1$ reduced to dimension 2) to display the overlap of the signatures of the nine proteins. The different pannels correspond to conditions described in Table S8.

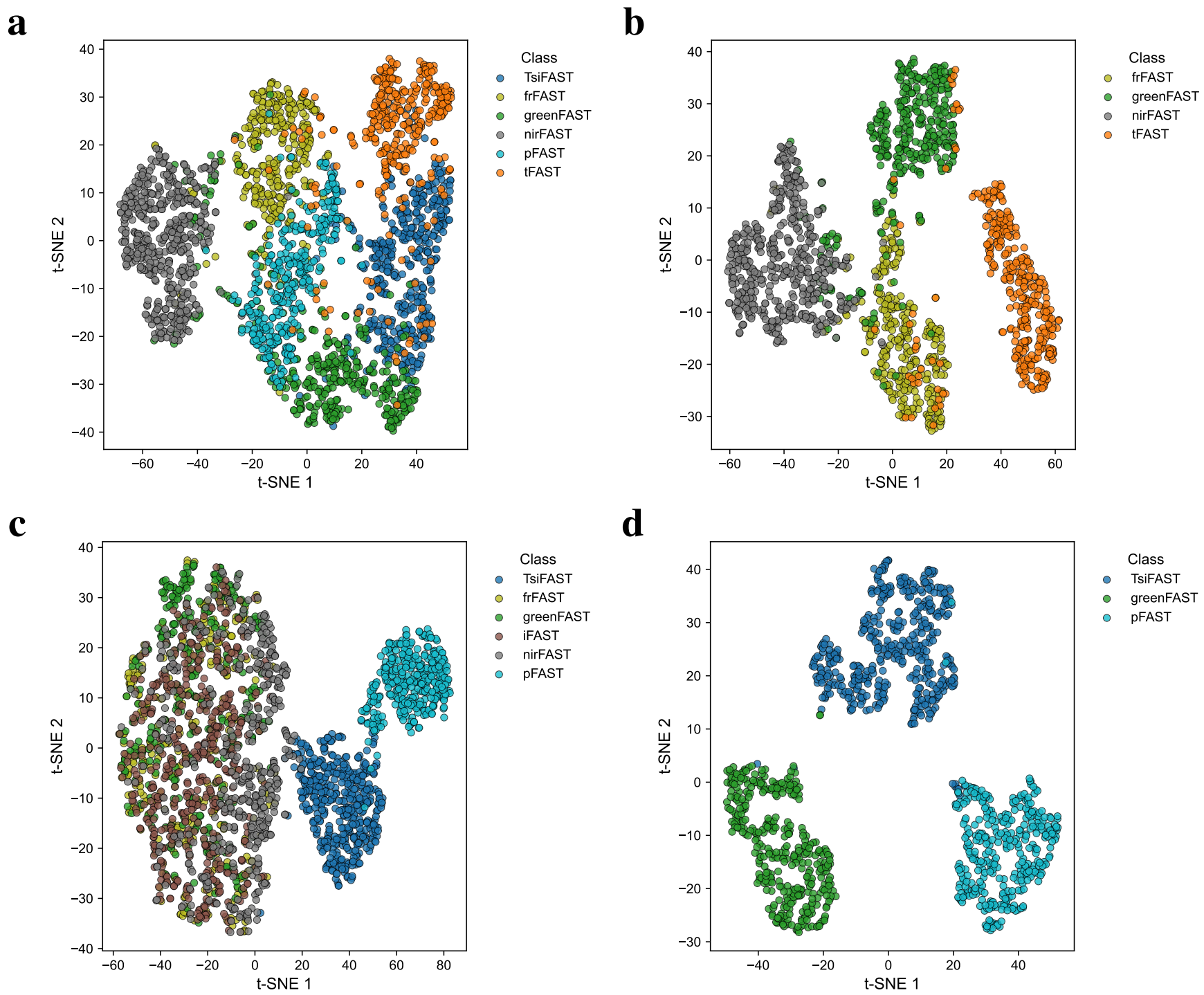

Figure S52: *2D visualisation of the distance between sample points per protein when only one channel is used (green or red)* A linear supervised reduction of dimension (Linear Discriminant Analysis, dimension 75 reduced to dimension $n-1$, where n is the number of proteins) is followed by a non-linear dimension reduction technique (t-SNE, dimension $n-1$ reduced to dimension 2). The reduction is performed using only the green channel (**a** - six proteins, **b** - four proteins) or red channel (**c** - six proteins, **d** - three proteins). The separability of the six proteins is lower when channels are not combined (see Figure 6 of the Main Text for combined channels). The results of the corresponding classification are provided in Table S8.

proteins	green	red	normalisation	accuracy	Related figure
6	■	■	first point	98%	S51a
7 (+ i)	■	■	first point	90%	S51f
7 (+ o)	■	■	first point	92%	S51f
7 (+ Y)	■	■	first point	92%	S51f
8 (no i)	■	■	first point	79%	S51f
8 (no o)	■	■	first point	83%	S51f
8 (no Y)	■	■	first point	83%	S51f
9	■	■	first point	75%	S51b,f
6	■		first point	78%	S51c, S52a
4	■		first point	97%	S52b
6		■	first point	75%	S51d, S52c
3		■	first point	100%	S52d
6	■	■	0-1	88%	S51e

Table S8: *Summary of comparative classification analyses.* Each row corresponds to a different condition tested. The last row correspond to confusion matrices in Figure S51.

5.5 Experimental conditions for acquiring the images of the Main Text and the Supporting Information

All the experiments that led to deliver the images reported in the Main Text and in the Supporting Information have been reproduced at least twice.

Table S9: *Experimental conditions for acquiring the images of the Main Text and the Supporting Information*. T = 310 K.

Figure	Fluorogen 1 ($C_1^{\dagger}$)	Fluorogen 2 ($C_2^{\dagger}$)	$\lambda_{exc,1}$ (nm)	$I_1^{\pm}$ ($E \cdot m^{-2} \cdot s^{-1}$)	$\lambda_{exc,2}$ (nm)	$I_2^{\mp}$ ($E \cdot m^{-2} \cdot s^{-1}$)	δ^* (μs)	$T^{\ddagger}$ (ms)	$Z^{\times}$	$N^{=}$
3a-c	**HBT35DM** (0.1, 1, 10)	–	405	9.1×10^3	–	–	0.49	300	4	3×2
3d-f	**HBR3M** (0.1, 1, 10)	–	488	1.7×10^4	–	–	0.49	300	4	6×2
3g-i	**HPAR3OM** (0.1, 1, 10)	–	639	3.0×10^4	–	–	0.49	300	4	9×2
3j-l	**HPAR35DOM** (0.1, 1, 10)	–	639	3.0×10^4	–	–	0.49	300	4	7×2
4a1-10,4b	**HBR3Cl** (1)	**HBR35DOM** (0.5)	488	4.9×10^3	–	–	0.98	150	4	3×2
4c1-4,4d	**HBR3Cl** (1)	**HBR35DOM** (2)	480	7.5×10^{-2}	–	–	–	100	2×2	
5a1-6	**HBR3Cl** (1)	**HBR35DOM** (0.5)	488	4.9×10^3	–	–	0.98	150	4	7×2
5b1-6	**HBT35DM** (10)	**HBR35DOM** (0.25)	445	6.5×10^3	561	2.6×10^3	0.49	300	4	3×2
5c1-6	**HPAR3F5OM** (0.5)	**HBR35DOM** (0.5)	561	4.8×10^3	–	–	1.00	150	4	2×2
6a1-5	**HBIR3Cl** (1)	**HBR35DOM** (0.25)	514	2.1×10^3	–	–	0.49	300	4	3×2
6b1-4	**HBIR3Cl** (1)	**HPAR35DOM** (0.5)	488	1.7×10^4	639	1.6×10^4	1.00	150	4	2×2
7a-c	**HBR3Cl** (1)	**HBR35DOM** (0.5)	488	1.4×10^4	–	–	1.34	410	2	3×2
7g-i	**HBR3Cl** (1)	**HBR35DOM** (0.5)	488	1.0×10^4	–	–	1.34	210	2.5	3×2
7m-o	**HBR3Cl** (1)	**HBR35DOM** (0.5)	488	1.0×10^4	–	–	1.34	210	2.5	3×2
8	**HBR3Cl** (1)	**HBR35DOM** (0.5)	488	4.9×10^3	–	–	0.98	150	4	10×1
S33	**HBR25DM** (0.1, 1, 10)	–	488	1.7×10^4	–	–	0.49	300	4	6×2
S34	**HPAR3Cl** (0.1, 1, 10)	–	488	1.7×10^4	–	–	0.49	300	4	5×2
S35	**HBR3F5OM** (0.1)	–	488	1.7×10^4	–	–	0.49	300	4	2×2
S36	**HPAR3Cl** (0.1)	–	488	1.7×10^4	–	–	0.49	300	4	2×2
S37	**HBR3F5OM** (0.1)	–	488	1.7×10^4	–	–	0.49	300	4	2×2
S38	**HBR3Cl** (1)	–	488	1.7×10^4	–	–	0.49	300	4	2×2
S39	**HBR3Cl** (1)	–	488	1.7×10^4	–	–	0.49	300	4	2×2
S40	**HBR3Cl** (1)	–	488	1.7×10^4	–	–	0.67	410	2	2×2
S41	**HBR3Cl** (1)	**HBR35DOM** (0.5)	488	4.9×10^3	–	–	0.98	150	4	2×10
S42	**HPAR3OM** (0.5)	**HBR35DOM** (0.25)	543	2.5×10^3	–	–	1.00	160	5	1×1
S43	**HBR3F5OM** (1)	**HPAR3OM** (1)	488	1.7×10^4	639	1.6×10^4	1.00	150	4	2×2
S44	**HBR3Cl** (1)	**HBR35DOM** (0.5)	488	4.9×10^3	–	–	0.49	300	4	2×2
S45	**HBR3Cl** (1)	**HBR35DOM** (0.5)	488	4.9×10^3	–	–	1.34	210	2	2×2
S46	**HBR3Cl** (1)	**HBR35DOM** (0.5)	488	4.9×10^3	–	–	1.34	210	2	2×5
S47	**HBR3Cl** (1)	**HBR35DOM** (0.5)	488	4.9×10^3	–	–	1.34	410	2	2×2

† C_i is the fluorogen concentration in μM.
* δ is the dwell time.
‡ T is the duration of frame acquisition.
$^{\pm}$ I_1 is the irradiance of the first light in the illumination.
$^{\mp}$ I_2 is the irradiance of the second light in the illumination.
$^{\times}$ Z is the zoom factor.
$^{=}$ N is the number of biological $\times$ technical repetitions.

To give comparative information on the fluorescence level of the **RSFASTs**, we complemented Table S9 reporting the experimental conditions of image acquisition and added Table S10 delivering estimates of the background fluorescence levels and fluorescence levels in the region of interest in the recorded images. The reported fluorescence levels have been corrected to account for the sensitivity of the detector in the observed spectral region. For **pFAST** and **nirFAST**, the brightness of the complex with **HBR3Cl** is known, which allowed us to estimate their relative levels of expression at the nucleus by dividing the fluorescence level by the product of applied light intensity and **RSFAST** brightness at 488 nm. In this way, the level of expression of **H2B-pFAST** and **H2B-nirFAST** were 3.0 $\pm$ 0.9 and 1.5 $\pm$ 0.3 (a.u.) respectively (N = 10).

Table S10: *Orders of magnitude of the background ($I_{F,1,min}$ and $I_{F,2,min}$) and region of interest ($I_{F,1,max}$ and $I_{F,2,max}$) values of the fluorescence levels and the contrast ratios $\rho_1 = I_{F,1,max}/I_{F,1,min}$ and $\rho_2 = I_{F,2,max}/I_{F,2,min}$ in for the images of the Main Text and the Supporting Information.* T = 310 K.

Figure	Fluorogen 1	Fluorogen 2	$\lambda_{\mathrm{em},1,\mathrm{min}}$-$\lambda_{\mathrm{em},1,\mathrm{max}}$ (nm)	$I_{F,1,min}$ (a.u.)	$I_{F,1,max}$ (a.u.)	ρ_1	$\lambda_{\mathrm{em},2,\mathrm{min}}$-$\lambda_{\mathrm{em},2,\mathrm{max}}$ (nm)	$I_{F,2,min}$ (a.u.)	$I_{F,2,max}$ (a.u.)	ρ_2
3a	**HBT35DM** (0.1)	–	446-606	112	427	3.8	–	–	–	–
3d	**HBR3M** (0.1)	–	499-597	67	3768	56.6	–	–	–	–
3g	**HPAR3OM** (0.1)	–	650-757	34	9225	274.2	–	–	–	–
3j	**HPAR35DOM** (0.1)	–	650-757	36	6772	190.6	–	–	–	–
4a	**HBR3Cl** (1)	**HBR35DOM** (0.5)	497-544	34	487	14.2	588-694	104	981	9.4
5a	**HBR3Cl** (1)	**HBR35DOM** (0.5)	497-544	12	1277	108.0	588-694	48	3603	74.4
5b	**HBT35DM** (10)	**HBR35DOM** (0.25)	446-526	54	1379	25.8	597-694	107	3991	37.3
5c	**HPAR3F5OM** (0.5)	**HBR35DOM** (0.5)	561-614	57	542	9.6	700-757	134	3317	24.7
6a	**HBIR3Cl** (1)	**HBR35DOM** (0.25)	570-694	108	534	4.9	–	–	–	–
6b	**HBIR3Cl** (1)	**HPAR35DOM** (0.5)	670-757	59	519	8.8	–	–	–	–
7a-c	**HBR3Cl** (1)	**HBR35DOM** (0.5)	501-544	18	642	36.6	588-693	68	1372	20.2
7g-i	**HBR3Cl** (1)	**HBR35DOM** (0.5)	501-544	43	1319	30.9	588-693	116	1556	13.4
7m-o	**HBR3Cl** (1)	**HBR35DOM** (0.5)	501-544	54	1490	27.6	588-693	99	1732	17.5
8 (pFAST)	**HBR3Cl** (1)	**HBR35DOM** (0.5)	497-544	23	668	29.6	588-694	107	2373	22.1
8 (tFAST)	**HBR3Cl** (1)	**HBR35DOM** (0.5)	497-544	21	776	37.6	588-694	86	744	8.7
8 (greenFAST)	**HBR3Cl** (1)	**HBR35DOM** (0.5)	497-544	21	144	6.8	588-694	86	150	1.7
8 (frFAST)	**HBR3Cl** (1)	**HBR35DOM** (0.5)	497-544	20	149	7.4	588-694	85	165	1.9
8 (nirFAST)	**HBR3Cl** (1)	**HBR35DOM** (0.5)	497-544	21	247	12.0	588-694	102	281	2.8
8 (tsiFAST)	**HBR3Cl** (1)	**HBR35DOM** (0.5)	497-544	19	767	40.3	588-694	95	1413	14.9
S33	**HBR25DM** (0.1)	–	499-597	86	4223	49.0	–	–	–	–
S34	**HPAR3Cl** (0.1)	–	570-694	334	2776	8.3	–	–	–	–
S35	**HBR3F5OM** (0.1)	–	499-606	299	7808	26.1	–	–	–	–
S36	**HPAR3Cl** (0.1)	–	570-757	312	2401	7.7	–	–	–	–
S37	**HBR3F5OM** (0.1)	–	499-606	251	5095	20.3	–	–	–	–
S38	**HBR3Cl** (1)	–	501-649	510	11233	22.0	–	–	–	–
S39	**HBR3Cl** (1)	–	501-649	542	4712	8.7	–	–	–	–
S40	**HBR3Cl** (1)	–	501-649	222	2531	11.4	–	–	–	–
S41	**HBR3Cl** (1)	**HBR35DOM** (0.5)	501-544	35	511	14.6	588-693	95	1001	10.5
S44	**HBR3Cl** (1)	**HBR35DOM** (0.5)	501-544	33	987	30.2	588-693	149	3429	23.0
S45	**HBR3Cl** (1)	**HBR35DOM** (0.5)	501-544	20	1113	56.3	588-693	70	3540	50.7
S46	**HBR3Cl** (1)	**HBR35DOM** (0.5)	501-544	17	383	22.8	588-693	67	673	10.1
S47	**HBR3Cl** (1)	**HBR35DOM** (0.5)	501-544	35	515	14.8	588-693	124	1555	12.6

References

[1] A. Stiel, S. Trowitzsch, G. Weber, M. Andresen, C. Eggeling, S. Hell, S. Jakobs, and M. Wahl. 1.8 å bright-state structure of the reversibly switchable fluorescent protein Dronpa guides the generation of fast switching variants. *Biochem. J.*, 402:35–42, 2007.

[2] J. Quérard, R. Zhang, Z. Kelemen, M.-A. Plamont, X. Xie, R. Chouket, I. Roemgens, Y. Korepina, S. Albright, E. Ipendey, M. Volovitch, H. L. Sladitschek, P. Neveu, L. Gissot, A. Gautier, J.-D. Faure, V. Croquette, T. Le Saux, and L. Jullien. Resonant out-of-phase fluorescence microscopy and remote imaging overcome spectral limitations. *Nat. Comm.*, 8:969, 2017.

[3] Marie-Aude Plamont, Emmanuelle Billon-Denis, Sylvie Maurin, Carole Gauron, Frederico M Pimenta, Christian G Specht, Jian Shi, Jérôme Quérard, Buyan Pan, Julien Rossignol, et al. Small fluorescence-activating and absorption-shifting tag for tunable protein imaging in vivo. *Proceedings of the national academy of sciences*, 113(3):497–502, 2016.

[4] Hela Benaissa, Karim Ounoughi, Isabelle Aujard, Evelyne Fischer, Rosette Goïame, Julie Nguyen, Alison G Tebo, Chenge Li, Thomas Le Saux, Giulia Bertolin, et al. Engineering of a fluorescent chemogenetic reporter with tunable color for advanced live-cell imaging. *Nature Communications*, 12(1):6989, 2021.

[5] Lina El Hajji, Benjamin Bunel, Octave Joliot, Chenge Li, Alison G Tebo, Christine Rampon, Michel Volovitch, Evelyne Fischer, Nicolas Pietrancosta, Franck Perez, et al. A tunable and versatile chemogenetic near-infrared fluorescent reporter. *Nature Communications*, 16(1):2594, 2025.

[6] Chenge Li, Alison G Tebo, Marion Thauvin, Marie-Aude Plamont, Michel Volovitch, Xavier Morin, Sophie Vriz, and Arnaud Gautier. A far-red emitting fluorescent chemogenetic reporter for in vivo molecular imaging. *Angewandte Chemie International Edition*, 59(41):17917–17923, 2020.

[7] Alison G Tebo, Benjamien Moeyaert, Marion Thauvin, Irene Carlon-Andres, Dorothea Böken, Michel Volovitch, Sergi Padilla-Parra, Peter Dedecker, Sophie Vriz, and Arnaud Gautier. Orthogonal fluorescent chemogenetic reporters for multicolor imaging. *Nature chemical biology*, 17(1):30–38, 2021.

[8] Alison G Tebo, Frederico M Pimenta, Yu Zhang, and Arnaud Gautier. Improved chemical-genetic fluorescent markers for live cell microscopy. *Biochemistry*, 57(39):5648–5653, 2018.

[9] Louise-Marie Rakotoarison, Alison G Tebo, Dorothea Boken, Stephanie Board, Lina El Hajji, and Arnaud Gautier. Improving split reporters of protein–protein interactions through orthology-based protein engineering. *ACS Chemical Biology*, 19(2):428–441, 2024.

[10] Ian Coghill, Fivos Pham, Mrinal Mandal, Alienor Lahlou, Yuriy Shpinov, Isabelle Aujard, Marie-Aude Plamont, Jacques Fattaccioli, Thomas Le Saux, and Ludovic Jullien. Spatiotemporal light modulation for optical sectioning, diffusion measurement and parallelized experiments with a dual-dmd microscope. 2026.

[11] Aliénor Lahlou, Hessam Sepasi Tehrani, Ian Coghill, Yuriy Shpinov, Mrinal Mandal, Marie-Aude Plamont, Isabelle Aujard, Yuxi Niu, Ladislav Nedbal, Dusan Lazár, et al. Fluorescence to measure light intensity. *Nature Methods*, 20(12):1930–1938, 2023.

[12] Yuriy Shpinov, Ian Coghill, Mrinal Mandal, Philippe Pelupessy, Isabelle Aujard, Thomas Le Saux, and Ludovic Jullien. A modular low-cost 3d uniformly illuminated mini-photoreactor with versatile in-situ analysis. *Advanced Optical Materials*, page 2403466, 2025.

[13] Anne Bourdoncle, A Estevez Torres, Charlie Gosse, Laurent Lacroix, Pierre Vekhoff, Thomas Le Saux, Ludovic Jullien, and J-L Mergny. Quadruplex-based molecular beacons as tunable dna probes. *Journal of the American Chemical Society*, 128(34):11094–11105, 2006.

[14] Harald Gampp, Marcel Maeder, Charles J Meyer, and Andreas D Zuberbühler. Calculation of equilibrium constants from multiwavelength spectroscopic data—i: Mathematical considerations. *Talanta*, 32(2):95–101, 1985.

[15] Harald Gampp, Marcel Maeder, Charles J Meyer, Andreas D Zuberbu, et al. Calculation of equilibrium constants from multiwavelength spectroscopic data—ii32, 95.: Specfit: two user-friendly programs in basic and standard fortran 77. *Talanta*, 32(4):257–264, 1985.

[16] Harald Gampp, Marcel Maeder, Charles J Meyer, and Andreas D Zuberbühler. Calculation of equilibrium constants from multiwavelength spectroscopic data—iii: model-free analysis of spectrophotometric and esr titrations. *Talanta*, 32(12):1133–1139, 1985.

[17] Harald Gampp, Marcel Maeder, Charles J Meyer, and Andreas D Zuberbühler. Calculation of equilibrium constants from multiwavelength spectroscopic data—iv: Model-free least-squares refinement by use of evolving factor analysis. *Talanta*, 33(12):943–951, 1986.

[18] Y. Shpinov et al. Kinetic and mechanistic characterization of rspfast, a non-covalent versatile chemogenetic platform for fluorescence imaging. page In revision to J. Am. Chem. Soc., 2025.

[19] R. Chouket, A. Pellissier-Tanon, A. Lahlou, R. Zhang, D. Kim, M.-A. Plamont, M. Zhang, X. Zhang, P. Xu, N. Desprat, D. Bourgeois, A. Espagne, A. Lemarchand, T. Le Saux, and L. Jullien. Extra kinetic dimensions for label discrimination. *Nature communications*, 13(1):1–8, 2022.
